# Effectiveness and Safety of Enteric-Coated vs Uncoated Aspirin in Patients With Cardiovascular Disease: A Secondary Analysis of the ADAPTABLE Randomized Clinical Trial

**DOI:** 10.1001/jamacardio.2023.3364

**Published:** 2023-10-04

**Authors:** Amber Sleem, Mark B. Effron, Amanda Stebbins, Lisa M. Wruck, Guillaume Marquis-Gravel, Daniel Muñoz, Richard N. Re, Kamal Gupta, Carl J. Pepine, Sandeep K. Jain, Saket Girotra, Jeffrey Whittle, Catherine P. Benziger, Peter M. Farrehi, Kirk U. Knowlton, Tamar S. Polonsky, Matthew T. Roe, Russell L. Rothman, Robert A. Harrington, W. Schuyler Jones, Adrian F. Hernandez

**Affiliations:** 1 Department of Medicine, Ochsner Medical Center, New Orleans, Louisiana; 2 John Ochsner Heart and Vascular Institute, University of Queensland-Ochsner Clinical School, New Orleans, Louisiana; 3 Duke Clinical Research Institute, Duke University, Durham, North Carolina; 4 Department of Biostatistics and Bioinformatics, Duke University School of Medicine, Durham, North Carolina; 5 Montreal Heart Institute, Montreal, Canada; 6 Department of Medicine, Duke University Health System, Durham, North Carolina; 7 Vanderbilt University Medical Center, Nashville, Tennessee; 8 Research Division, Ochsner Medical Center, New Orleans, Louisiana; 9 University of Kansas Medical Center, Kansas City; 10 Department of Medicine, University of Florida, Gainesville; 11 Heart and Vascular Institute, University of Pittsburgh Medical Center, Pittsburgh, Pennsylvania; 12 Division of Cardiology, Department of Medicine, University of Texas Southwestern Medical Center, Dallas; 13 Department of Medicine, Medical College of Wisconsin, Milwaukee; 14 Essentia Health Heart and Vascular Center, Duluth, Minnesota; 15 University of Michigan, Ann Arbor; 16 Intermountain Medical Center Heart Institute, Salt Lake City, Utah; 17 University of Chicago Medicine, Department of Medicine, Chicago, Illinois; 18 Department of Medicine, Stanford University School of Medicine, Stanford, California

## Abstract

**Importance:**

Clinicians recommend enteric-coated aspirin to decrease gastrointestinal bleeding in secondary prevention of coronary artery disease even though studies suggest platelet inhibition is decreased with enteric-coated vs uncoated aspirin formulations.

**Objective:**

To assess whether receipt of enteric-coated vs uncoated aspirin is associated with effectiveness or safety outcomes.

**Design, Setting, and Participants:**

This is a post hoc secondary analysis of ADAPTABLE (Aspirin Dosing: A Patient-Centric Trial Assessing Benefits and Long-term Effectiveness), a pragmatic study of 15 076 patients with atherosclerotic cardiovascular disease having data in the National Patient-Centered Clinical Research Network. Patients were enrolled from April 19, 2016, through June 30, 2020, and randomly assigned to receive high (325 mg) vs low (81 mg) doses of daily aspirin. The present analysis assessed the effectiveness and safety of enteric-coated vs uncoated aspirin among those participants who reported aspirin formulation at baseline. Data were analyzed from November 11, 2019, to July 3, 2023.

**Intervention:**

ADAPTABLE participants were regrouped according to aspirin formulation self-reported at baseline, with a median (IQR) follow-up of 26.2 (19.8-35.4) months.

**Main Outcomes and Measures:**

The primary effectiveness end point was the cumulative incidence of the composite of myocardial infarction, stroke, or death from any cause, and the primary safety end point was major bleeding events (hospitalization for a bleeding event with use of a blood product or intracranial hemorrhage). Cumulative incidence at median follow-up for primary effectiveness and primary safety end points was compared between participants taking enteric-coated or uncoated aspirin using unadjusted and multivariable Cox proportional hazards models. All analyses were conducted for the intention-to-treat population.

**Results:**

Baseline aspirin formulation used in ADAPTABLE was self-reported for 10 678 participants (median [IQR] age, 68.0 [61.3-73.7] years; 7285 men [68.2%]), of whom 7366 (69.0%) took enteric-coated aspirin and 3312 (31.0%) took uncoated aspirin. No significant difference in effectiveness (adjusted hazard ratio [AHR], 0.94; 95% CI, 0.80-1.09; *P* = .40) or safety (AHR, 0.82; 95% CI, 0.49-1.37; *P* = .46) outcomes between the enteric-coated aspirin and uncoated aspirin cohorts was found. Within enteric-coated aspirin and uncoated aspirin, aspirin dose had no association with effectiveness (enteric-coated aspirin AHR, 1.13; 95% CI, 0.88-1.45 and uncoated aspirin AHR, 0.99; 95% CI, 0.83-1.18; interaction *P* = .41) or safety (enteric-coated aspirin AHR, 2.37; 95% CI, 1.02-5.50 and uncoated aspirin AHR, 0.89; 95% CI, 0.49-1.64; interaction *P* = .07).

**Conclusions and Relevance:**

In this post hoc secondary analysis of the ADAPTABLE randomized clinical trial, enteric-coated aspirin was not associated with significantly higher risk of myocardial infarction, stroke, or death or with lower bleeding risk compared with uncoated aspirin, regardless of dose, although a reduction in bleeding with enteric-coated aspirin cannot be excluded. More research is needed to confirm whether enteric-coated aspirin formulations or newer formulations will improve outcomes in this population.

**Trial Registration:**

ClinicalTrials.gov Identifier: NCT02697916

## Key Points

**Question:**

Does enteric coating on aspirin reduce effectiveness or increase safety in patients with cardiovascular disease?

**Findings:**

In this post hoc secondary analysis of 10 678 participants with atherosclerotic cardiovascular disease from the ADAPTABLE randomized clinical trial, there were no significant differences in the primary effectiveness (death, hospitalization for myocardial infarction, or hospitalization for stroke) or safety (major bleeding) end points between enteric-coated aspirin and uncoated aspirin among participants, regardless of which dose of aspirin they were assigned.

**Meaning:**

These findings suggested that enteric coating on aspirin is not associated with changes in the effectiveness or safety of aspirin for secondary prevention of cardiovascular events, allowing patients to determine the aspirin formulation.

## Teaser

This post hoc secondary analysis of a randomized clinical trial compares the effectiveness and safety of enteric-coated aspirin vs uncoated aspirin among patients with atherosclerotic cardiovascular disease.

## Introduction

Aspirin has been one of the most widely used medications since its introduction in the 1890s.^1^ Its irreversible inhibition of both cyclooxygenase 1 synthase reducing production of the eicosanoid thromboxane A2 needed for platelet aggregation makes it ideal for prevention of ischemic cardiovascular events, such as myocardial infarction (MI), stroke, and transient ischemic attack^1,2,3^ but at the price of substantial adverse effects, including gastrointestinal (GI) tract bleeding, intracranial hemorrhage (ICH), and generalized bleeding.^4^ Enteric coating of aspirin delays the breakdown of the tablet until it is in the higher pH of the duodenum and has been shown to reduce gastric erosion^5^ but has not been shown to reduce gastrointestinal bleeding.^6,7,8,9^ Historically among clinicians and advanced practice providers such as nurse practitioners and physician assistants, it has been recommended for patients to use enteric-coated aspirin over the plain pressed uncoated formulations to minimize GI tract ulceration and bleeding,^10^ but to our knowledge, no study has shown that the enteric-coated formulation is safer than uncoated aspirin.

The association of enteric-coated aspirin with secondary prevention of cardiovascular disease is controversial. Several studies have proposed that the enteric coating reduces the bioavailability of aspirin due to reduced dissolution and absorption.^2,11,12,13,14^ In 2021, the ADAPTABLE (Aspirin Dosing: A Patient-Centric Trial Assessing Benefits and Long-term Effectiveness) trial evaluated the safety and effectiveness of high-dose (325 mg) vs low-dose (81 mg) daily aspirin in 15 076 patients with established atherosclerotic cardiovascular disease (ASCVD).^15^ The results of the trial showed no statistical significance between high- and low-dose aspirin on the primary composite end points of all-cause death, hospitalization for MI, or hospitalization for stroke, as well as no significant difference in major bleeding between the 2 doses of aspirin.^15^ The present post hoc secondary analysis of the ADAPTABLE trial was designed to provide insight into the effectiveness and safety of enteric-coated aspirin compared with uncoated aspirin among patients with ASCVD.

## Methods

The ADAPTABLE study design has been previously described in detail.^15,16^ The trial protocol and statistical analysis plan are provided in Supplement 1, and the trial protocol for the enteric-coated aspirin assessment reported here is provided in Supplement 2. In brief, ADAPTABLE was a pragmatic, open-label, multicenter, and patient-centered randomized clinical trial evaluating the effectiveness and safety of 2 daily doses of aspirin (81 mg compared with 325 mg) in patients with established ASCVD. Potential participants from 40 centers and 1 private insurance health plan from the National Patient-Centered Clinical Research Network were identified with the use of electronic health record data at each institution through a cohort identification query (termed *computable phenotype*) and enrolled from April 19, 2016, through June 30, 2020. Data were analyzed from November 11, 2019, to July 3, 2023. Participants were randomly assigned in a 1:1 ratio to receive 81 mg or 325 mg of daily aspirin. Baseline demographic characteristics were participant self-reported, with baseline medical history and comorbidities ascertained from electronic health records queries. Information on race and ethnicity were collected on participants to better understand whether these parameters made a difference in a participants response to aspirin dose or formulation. Race included Black or African American, White, and other (Asian, American Indian or Alaska Native, multiple race, or other). Ethnicity included Hispanic and non-Hispanic. Multiple sources were taken into consideration to compile end points, including queries of the electronic health records harmonized into a National Patient-Centered Clinical Research Network Common Data Model, private health plan partners, US Centers for Medicare & Medicaid Services claims data, online participant portals, and remote phone call study visits from the Duke Clinical Research Institute. This end point adjudication method was validated to capture major bleeding.^17^ This study was approved by the institutional review board of all participating sites. All participants in the trial provided written informed consent. The present study followed the Consolidated Standards of Reporting Trials (CONSORT) reporting guideline.

Overall, 15 076 participants 18 years or older with known ASCVD and at least 1 enrichment factor (age >65 years, serum creatinine >1.5 mg/dL [to convert milligrams per deciliter to micromoles per liter, multiply by 88.4], diabetes type 1 or type 2, 3-vessel coronary artery disease, cerebrovascular disease or peripheral arterial disease, left ventricular ejection fraction lower than 50%, and current cigarette smoker), no history of significant GI bleed within the last 12 months, and not currently treated with an oral anticoagulant or with ticagrelor were enrolled. The primary effectiveness end point was the time to a first occurrence of any event in the composite of death from any cause, hospitalization for MI, or hospitalization for stroke. The secondary effectiveness end points were the individual components of the primary end point, all-cause mortality, revascularization (percutaneous coronary intervention [PCI] or coronary artery bypass graft [CABG]), and transient ischemic attack. The primary safety end point was hospitalization for major bleeding with an associated blood product transfusion. Gastrointestinal tract bleeding was also evaluated as a safety end point. For this analysis, participants were divided into subgroups based on randomized aspirin dose and self-reported aspirin formulation (enteric-coated aspirin or uncoated aspirin) at the time of randomization. Participants who did not answer the question related to aspirin formulation, or reported that they did not know the formulation, were excluded from the analyses. It was assumed that the participants remained on the same aspirin formulation throughout the study, as information on enteric coating was not collected past baseline.

### Statistical Analysis

The statistical analysis plan was prespecified before conducting the analyses but after database lock. Baseline characteristics, medical history, and selected concomitant medications are presented separately for participants who took enteric-coated or uncoated aspirin by randomly assigned aspirin dose. Discrete variables are presented as counts and percentages, and continuous variables as medians and IQRs. The χ^2^ test was used to compared discrete variables between groups, and the Wilcoxon rank sum test was used for continuous variables.

Cumulative incidence at median follow-up for primary and secondary effectiveness end points and the primary safety end point was compared between participants taking enteric-coated or uncoated aspirin using unadjusted and multivariable Cox proportional hazards models. Cumulative incidence was calculated at median follow-up (26.2 months; IQR, 19.8-35.4 months) using the Kalbfleisch and Prentice cumulative incidence function estimator and comparisons reported as hazard ratios (HRs) with 95% CI. The Fine-Gray method was used to account for the competing risk of death for nonlethal secondary effectiveness and primary safety outcomes. Primary effectiveness end point, MI, all-cause mortality, and revascularization adjustment measures were randomly assigned treatment, age, current smoking, randomized follow-up strata, no internet use at randomization, race, ethnicity, history of coronary artery disease (CAD), MI, CABG, PCI, ASCVD, hypertension, hyperlipidemia, atrial fibrillation, congestive heart failure (CHF), peripheral artery disease (PAD), prior aspirin use, body mass index (BMI), diabetes, history of bleeding, and baseline P2Y_12_ inhibitor use. The primary safety end point adjustment measures were randomly assigned treatment, age, sex, hypertension, baseline P2Y_12_ inhibitor use, diabetes, history of bleeding, and BMI. Stroke, transient ischemic attack, and GI tract bleeding adjustment measures were randomly assigned treatment, age, current smoking, ethnicity, race, history of CAD, ASCVD, hypertension, hyperlipidemia, atrial fibrillation, CHF, PAD, diabetes, history of bleeding, prior aspirin use, baseline P2Y_12_ inhibitor use, and BMI.

To assess whether the association of aspirin formulation was consistent across randomly assigned aspirin dose for selected end points (primary effectiveness end point, all-cause mortality, and primary safety end point), we included an interaction term in the multivariable Cox proportional hazards models and report tests of the interactions terms. Counts and cumulative incidence at median follow-up with 95% CI are reported by randomized aspirin dose and aspirin formulation. Comparisons of aspirin formulation group, by randomly assigned aspirin dose, are reported as aspirin dose-specific HRs with 95% CI. Cumulative incidence figures reporting the primary effectiveness end point and the primary safety end point are provided by randomly assigned aspirin dose and aspirin formulation. The interaction models were adjusted for covariates prespecified as potential confounders and included age, sex, ethnicity, randomly assigned follow-up stratum (follow-up every 3 or 6 months), race, prior aspirin use, P2Y_12_ inhibitor use at baseline, smoking status, no internet use at randomization, history of atrial fibrillation, bleeding, CAD, CHF, cardiovascular disease, diabetes, hypertension, hyperlipidemia, prior MI, and PAD.

To account for nonadherence to the randomly assigned aspirin dose, we conducted a sensitivity analysis to assess if aspirin formulation modified the association of time-varying participant-reported aspirin dose with selected study end points (primary effectiveness end point, all-cause death, and primary safety end point). We constructed a Cox proportional hazards model with actual aspirin dose (self-reported at 81 mg, 325 mg, not taking, or other) as a time-varying exposure, an interaction term of time-varying aspirin dose and aspirin formulation reported at baseline, and selected covariates. The HRs were interpreted as the association of current aspirin dose with outcomes. The key assumption for this model was that the decision to switch doses was made for reasons unrelated to the outcome of interest (ie, the primary end point of death, MI, or stroke), beyond the baseline adjustment measures. The adjusted models included age, race, ethnicity, prior aspirin dose, prior MI, prior PCI, history of atrial fibrillation, no internet use at randomization, history of bleeding, and baseline P2Y_12_ inhibitor use.

In an exploratory analysis, we also tested whether there was an interaction with an acid reducing medication (ARM) by formulation of aspirin used. Multivariable Cox proportional hazards models were used to assess whether the association of enteric coating with prespecified end points was modified by the use of ARM. In this analysis, primary end point, nonfatal MI, all-cause mortality, and revascularization adjustment measures were randomly assigned treatment, proton pump inhibitor medication, age, current smoking, randomized follow-up strata (3-month or 6-month intervals), no internet use at randomization, race, ethnicity, history of CAD, MI, CABG, PCI, cardiovascular disease, hypertension, hyperlipidemia, atrial fibrillation, CHF, PAD, prior aspirin use, BMI, and baseline P2Y_12_ inhibitor use. Major bleeding adjustment measures were randomly assigned treatment, proton pump inhibitor medication, age, sex, hypertension, baseline P2Y_12_ inhibitor use, and BMI.

All hypothesis tests were 2-sided, and *P* < .05 was interpreted as statistically significant without adjustment for multiple comparisons in this post hoc subgroup analysis. Modeling assumptions of linearity and proportional hazards were tested, and transformations were included when necessary. Missing covariates were handled using multiple imputation. All analyses were conducted for the intention-to-treat population and were performed at the Duke Clinical Research Institute (Durham, North Carolina) using SAS, version 9.4 (SAS Institute Inc).

## Results

### Demographic Characteristics, Medication Use, and Medical History

**Figure 1.**
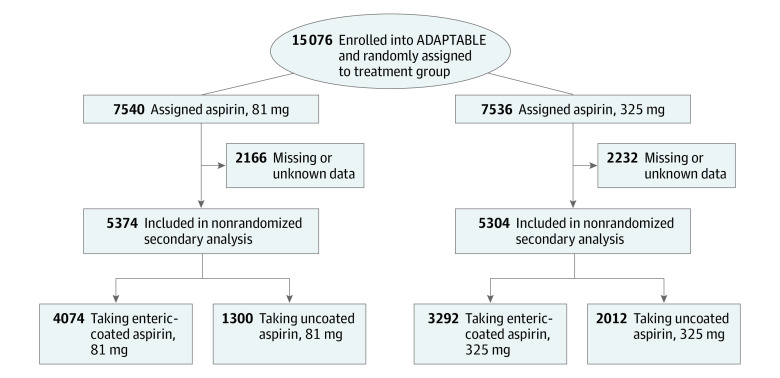
Diagram of Patient Flow in the Analysis Participants were randomly assigned to aspirin dose but not to aspirin formulation, which was at the discretion of the participant. For several analyses, participants were grouped by aspirin formulation regardless of aspirin dose (shown at the bottom of the figure). Regardless of aspirin dose, there were 7366 total participants taking enteric-coated aspirin and 3312 taking uncoated aspirin.

Of 15 076 participants enrolled in the ADAPTABLE study, 10 678 (70.1%) reported the aspirin formulation used (median [IQR] age, 68.0 [61.3-73.7] years; 7285 [68.2%] men and 3393 [31.8%] women; 900 [8.4%] Black or African American, 8965 [84.0%] White, 482 [4.5%] other race (Asian, American Indian or Alaska Native, multiple race, or other), and 331 missing race data or preferred not to say; 270 [2.5%] Hispanic, 10 051 [94.2%] non-Hispanic, and 354 [3.3%] did not respond to ethnicity query) (Table 1). Aspirin formulation was reported by 5374 (71.3%) participants in the 81-mg dose cohort, with enteric-coated aspirin used by 54.0%, and by 5304 (70.4%) participants in the 325-mg dose cohort, with enteric-coated aspirin used by 44.3% (Figure 1). There was a greater predilection for enteric-coated aspirin use over uncoated aspirin (7536 [69.0%] vs 3312 [31.0%]). Almost all participants (96.2%) reported aspirin use prior to enrolling in ADAPTABLE, with more participants in the 81-mg dose cohort taking enteric-coated aspirin than uncoated aspirin (86.0% vs 82.5%) and fewer participants using enteric-coated aspirin than uncoated aspirin in the 325-mg dose cohort (11.4% vs 15.3%) (*P* < .001). At the time of enrollment 22% of the participants were taking a P2Y_12_ inhibitor in both aspirin groups. The percentage of participants taking uncoated aspirin was higher than for participants taking enteric-coated aspirin among current smokers, individuals with diabetes, or with a history of bleeding (eTable 1 in Supplement 3). All other demographic characteristics, medical history, and medication use recorded were not significantly different across the 4 groups, except that more participants who had hyperlipidemia used enteric-coated aspirin (6426 of 7339 [87.6%]) rather than uncoated aspirin (2806 of 3312 [84.7%]; *P* < .001) (Table 1). Overall use of an ARM was also similar across aspirin cohorts, although an ARM was used more frequently by participants taking enteric-coated aspirin compared with uncoated aspirin (38% vs 35%; *P* < .002) (eTable 1 in in Supplement 3).

**Table 1.** Participant Demographic and Presenting Characteristics, Medical History, and Medications

Characteristic	Participants, No. (%)					*P* value
	All (n = 10 678)	Enteric-coated aspirin		Uncoated aspirin		
		81 mg (n = 4074)	325 mg (n = 3292)	81 mg (n = 1300)	325 mg (n = 2012)	
Age, median (IQR), y	68.0 (61.3-73.7)	68.4 (61.8-73.8)	68.2 (61.7-73.8)	67.1 (60.1-73.2)	67.2 (60.6-73.4)	<.001
Sex						
Male	7285 (68.2)	2827 (69.4)	2235 (67.9)	863 (66.4)	1360 (67.6)	.16
Female	3393 (31.8)	1247 (30.6)	1057 (32.1)	437 (33.6)	652 (32.4)	
History of PAD	2436 (23.4)	916 (23.0)	736 (22.9)	298 (23.7)	486 (24.7)	.46
Race						
Black or African American	900 (8.4)	335 (8.2)	257 (7.8)	147 (11.3)	161 (8.0)	.003
White	8965 (84.0)	3432 (84.2)	2786 (84.6)	1040 (80.0)	1707 (84.8)	
Other^a^	482 (4.5)	195 (4.8)	136 (4.1)	68 (5.2)	83 (4.1)	
Prefer not to say/missing	331 (3.1)	112 (2.7)	113 (3.4)	45 (3.5)	61 (3.0)	
Ethnicity						
Hispanic	270 (2.5)	101 (2.5)	80 (2.4)	42 (3.2)	47 (2.3)	.39
Non-Hispanic	10 051 (94.2)	3850 (94.5)	3104 (94.3)	1204 (92.8)	1893 (94.1)	
Did not respond	354 (3.3)	123 (3.0)	108 (3.3)	51 (3.9)	72 (3.6)	
Current smoker	1006 (9.4)	368 (9.0)	272 (8.3)	144 (11.1)	222 (11.0)	.001
Baseline P2Y_12_ inhibitor use	2300 (22.0)	880 (22.0)	707 (22.0)	294 (23.1)	419 (21.3)	.68
EHR and claims information						
Weight						
No.	9313	3572	2879	1125	1737	.02
Median (IQR), kg	90.0 (78.2-103.6)	90.0 (78.6-103.6)	89.5 (77.3-103.6)	90.0 (77.7-103.2)	91.4 (79.5-105.9)	
Height						
No.	9172	3532	2838	1104	1698	.41
Median (IQR), cm	172.7 (165.1-180.3)	172.7 (165.1-180.3)	172.7 (165.1-180.3)	172.7 (165.1-180.3)	172.7 (165.1-180.3)	
BMI						
No.	9074	3486	2807	1097	1684	.01
Median (IQR)	30.0 (26.7-34.2)	30.1 (26.7-34.2)	29.9 (26.5-34.0)	29.9 (26.6-34.2)	30.3 (27.0-34.8)	
Atrial fibrillation or flutter	884 (8.5)	325 (8.2)	286 (8.9)	101 (8.0)	172 (8.7)	.64
History of bleeding^b^	894 (8.6)	290 (7.3)	305 (9.5)	129 (10.3)	170 (8.6)	<.001
Significant GI tract bleed	661 (6.3)	222 (5.6)	224 (7.0)	97 (7.7)	118 (6.0)	.01
History of ICH	155 (1.5)	50 (1.3)	55 (1.7)	16 (1.3)	34 (1.7)	.30
History of bleeding disorder	126 (1.2)	35 (0.9)	38 (1.2)	24 (1.9)	29 (1.5)	.02
Prior CABG	2525 (24.2)	989 (24.9)	774 (24.1)	291 (23.2)	471 (23.9)	.63
Coronary artery disease	9763 (93.7)	3730 (93.7)	3036 (94.5)	1165 (92.8)	1832 (93.0)	.07
CHF	2392 (23.0)	902 (22.7)	744 (23.2)	282 (22.5)	464 (23.6)	.84
Chronic kidney disease	1835 (17.6)	718 (18.0)	547 (17.0)	217 (17.3)	353 (17.9)	.69
COPD	1938 (18.6)	724 (18.2)	605 (18.8)	235 (18.7)	374 (19.0)	.86
CVD	1880 (18.0)	713 (17.9)	566 (17.6)	231 (18.4)	370 (18.8)	.74
Diabetes	3988 (38.3)	1472 (37.0)	1224 (38.1)	517 (41.2)	775 (39.3)	.04
Hypertension	8873 (85.2)	3413 (85.8)	2734 (85.1)	1060 (84.5)	1666 (84.6)	.53
Hyperlipidemia	9232 (88.6)	3547 (89.1)	2879 (89.6)	1088 (86.7)	1718 (87.2)	.005
Prior MI	3739 (35.9)	1403 (35.3)	1144 (35.6)	473 (37.7)	719 (36.5)	.41
Peptic ulcer disease	312 (3.0)	120 (3.0)	91 (2.8)	39 (3.1)	62 (3.1)	.92
PCI or CABG	5648 (54.2)	2183 (54.9)	1728 (53.8)	682 (54.3)	1055 (53.6)	.74
Prior aspirin use	10 275 (96.2)	3932 (96.5)	3195 (97.1)	1231 (94.7)	1917 (95.3)	<.001
Prior aspirin dose						
81 mg	8714 (84.8)	3344 (85.0)	2781 (87.0)	1014 (82.4)	1575 (82.2)	<.001
162 mg	250 (2.4)	102 (2.6)	77 (2.4)	30 (2.4)	41 (2.1)	
325 mg	1295 (12.6)	481 (12.2)	334 (10.5)	184 (14.9)	296 (15.4)	
Other	16 (0.2)	5 (0.1)	3 (0.1)	3 (0.2)	5 (0.3)	
ARM use	3904 (37.3)	1528 (38.2)	1239 (38.5)	440 (34.6)	697 (35.4)	.01

Abbreviations: ARM, acid reducing medication; BMI, body mass index (calculated as weight in kilograms divided by height in meters squared); CABG, coronary artery bypass graft; CHF, congestive heart failure; COPD, chronic obstructive pulmonary disease; CVD, cardiovascular disease; EHR, electronic health record; GI, gastrointestinal; ICH, intracerebral hemorrhage; MI, myocardial infarction; PAD, peripheral artery disease; PCI, percutaneous coronary intervention.

^a^ Other includes Asian, American Indian or Alaska Native, multiple race, or other.

^b^ History of bleeding includes GI tract bleed, ICH, and bleeding disorder.

### Effectiveness Outcomes

**Figure 2.**
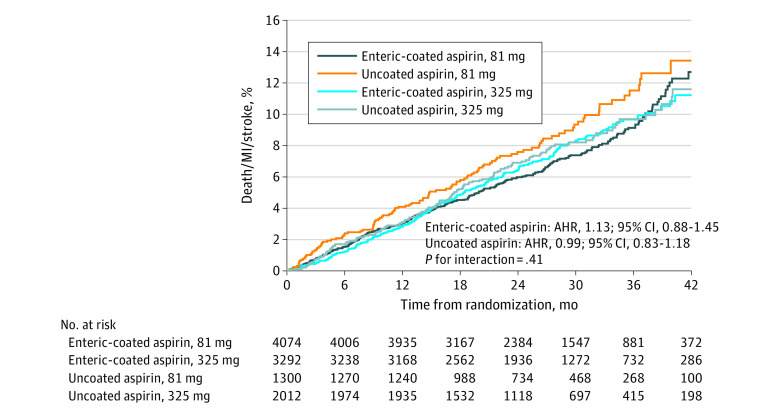
Cumulative Incidence of the Primary End Point (All-Cause Death, Myocardial Infarction [MI], or Stroke) by Randomly Assigned Aspirin Dose (81 or 325 mg) and by Self-Selected Aspirin Formulation (Enteric-Coated or Uncoated) From Participant Self-Reported, Electronic Health Record, and Claims Data AHR indicates adjusted hazard ratio.

Within the enteric-coated aspirin cohort, the primary effectiveness end point occurred in 297 participants (cumulative incidence at median follow-up 6.6%) in the 81-mg dose group and 246 participants (7.2%) in the 325-mg dose group (adjusted hazard ratio [AHR], 1.13; 95% CI, 0.88-1.45) (Table 2, Figure 2). Similar results were observed with uncoated aspirin (AHR, 0.99; 95% CI, 0.83-1.18) with an interaction of *P* = .41 across the groups. All-cause mortality did not significantly differ across the 4 cohorts (AHR, 0.88; 95% CI, 0.63-1.23 for enteric-coated aspirin vs AHR, 0.90; 95% CI, 0.72-1.13 for uncoated aspirin; interaction *P* = .90) (Table 2). Grouping participants by aspirin formulation regardless of dose showed no difference in effectiveness outcomes (AHR, 0.94; 95% CI, 0.80-1.09; *P* = .40) (eTable 2 and eFigure 1 in Supplement 3). There was no significant interaction between enteric coating and the presence of ARM on the effectiveness end points (eTable 3 in Supplement 3).

**Table 2.** Cumulative Incidence of Study End Points by Aspirin Formulation Type and Randomly Assigned Dose of Aspirin

End point	Enteric-coated aspirin			Uncoated aspirin			*P* value for interaction
	Cumulative incidence (%)^a^		Adjusted HR (95% CI)^b^	Cumulative incidence (%)^a^		Adjusted HR (95% CI)^b^	
	81 mg	325 mg		81 mg	325 mg		
Death, MI, or stroke	297 (6.6)	246 (7.1)	1.13 (0.88-1.45)	114 (8.5)	152 (7.6)	0.99 (0.83-1.18)	.41
All-cause mortality	155 (3.6)	146 (3.8)	0.88 (0.63-1.23)	56 (3.9)	99 (4.5)	0.90 (0.72-1.13)	.90
Major bleeding	22 (0.5)	23 (0.7)	2.37 (1.02-5.50)	15 (1.0)	9 (0.4)	0.89 (0.49-1.64)	.07
GI tract bleeding	48 (1.2)	40 (1.2)	1.27 (0.73-2.22)	27 (1.7)	27 (1.4)	1.19 (0.76-1.84)	.85

Abbreviations: GI, gastrointestinal; MI, myocardial infarction.

^a^ Cumulative incidence is reported at median follow-up, 26.2 months from randomization.

^b^ Adjustment variables include age, sex, ethnicity, strata, race, prior aspirin use, P2Y_12_ inhibitor use at baseline, smoking status, no internet use, history of atrial fibrillation, history of bleeding, coronary artery disease, congestive heart failure, cardiovascular disease, diabetes, hypertension, hyperlipidemia, prior MI, and peripheral arterial disease.

### Safety Outcomes

**Figure 3.**
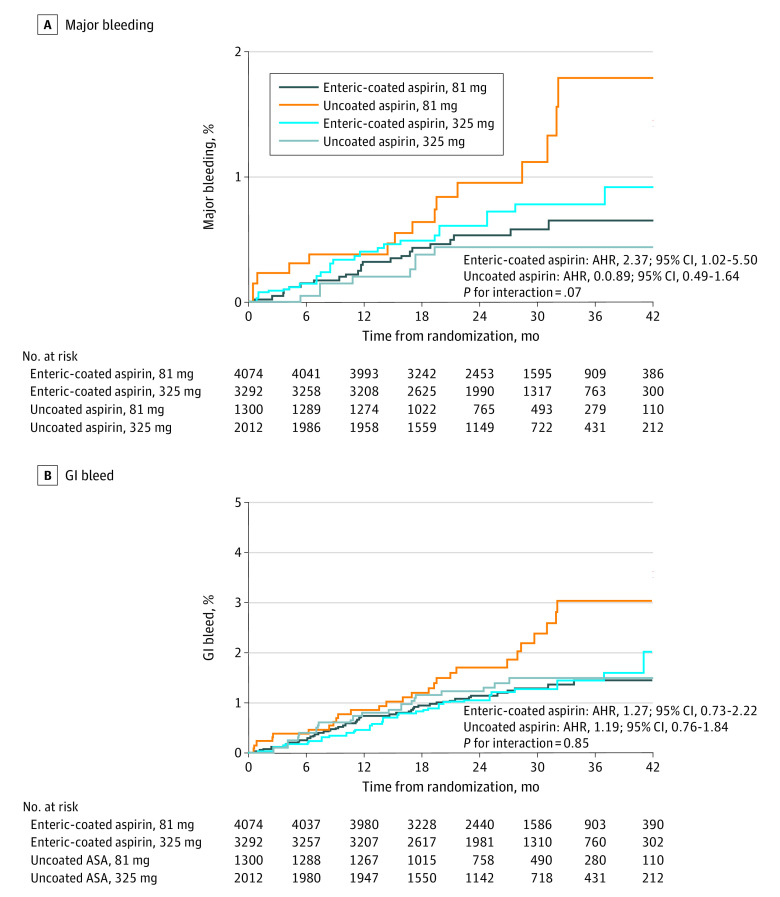
Cumulative Incidence of Bleeding Accounting for the Competing Risk of Death by Randomly Assigned Aspirin Dose (81 or 325 mg) and Self-Selected Aspirin Formulation (Enteric-Coated or Uncoated) From Participant Self-Reported, Electronic Health Record, and Claims Data Major bleeding was defined as bleeding requiring blood product transfusion. AHR indicates adjusted hazard ratio.

Overall bleeding requiring blood product transfusion (major bleeding) was low in ADAPTABLE. Across the 4 cohorts, there was no significant interaction of major bleeding by aspirin dose or formulation (Table 2, Figure 3A). Within the enteric-coated aspirin group, there was a small but significant increase in major bleeding with 325-mg dose aspirin (AHR, 2.37; 95% CI, 1.02-5.50) but no difference in major bleeding in the uncoated aspirin cohort (AHR, 0.89; 95% CI, 0.49-1.64; interaction *P* = .07) (Table 2, Figure 3A). There was also no significant difference in GI tract bleeding across the 4 formulations of aspirin (AHR, 1.27; 95% CI, 0.73-2.22 vs AHR, 1.19; 95% CI, 0.76-1.84; interaction *P* = .85) (Table 2, Figure 3B). Grouping participants by aspirin formulation regardless of dose showed no difference in safety outcomes (AHR, 0.82; 95% CI, 0.49-1.37; *P* = .46) (eTable 2 and eFigure 2 in Supplement 3). There was no significant interaction between enteric-coated aspirin and the presence of ARM on major or GI tract bleeding (eTable 3 in Supplement 3).

### Sensitivity Analyses

In a sensitivity analysis of time-varying self-reported aspirin dose, reflecting the association of aspirin dose at the time of the event, aspirin formulation, with or without covariate adjustment, did not modify the association of self-reported aspirin dose with the primary effectiveness outcome, all cause death, or the primary safety outcome (eTable 4 in Supplement 3).

## Discussion

In this post hoc secondary analysis of ADAPTABLE, a large multicenter, pragmatic, randomized clinical trial, we evaluated the association of aspirin formulation (enteric-coated vs uncoated aspirin) with the effectiveness and safety of aspirin in secondary prevention of ASCVD. The results of this analysis did not show any difference in the effectiveness or safety outcomes analyzed by formulation of aspirin consumed regardless of the dose of aspirin participants were randomly assigned. While prior pharmacodynamic studies^2,18^ have found that enteric coating impedes the temporal dissolution of the aspirin in the small intestine and limits overall drug absorption, the results of the present study suggest no clear differences in clinical outcomes and should promote further discussion about the appropriate formulation and dose for individual patients.

Due to reduced aspirin bioavailability and the possibility of limited cardiovascular protection, the value of enteric-coated aspirin has been called into question during the last few decades. Cox et al^18^ reported on a 2-week patient volunteer study comparing the bioavailability of aspirin and inhibition of thromboxane A2 between enteric-coated aspirin and uncoated aspirin. They found that 100% of patients using uncoated aspirin showed higher than 95% thromboxane A2 inhibition compared with 87% of patients receiving enteric-coated aspirin (*P* < .001). A similar study by Grosser et al^10^ with 400 patients showed that 83% of patients who took a single-dose of enteric-coated aspirin (325 mg) had greater than 60% reduction in cyclooxygenase activity in 8 hours compared with 100% of patients who took a single dose of uncoated aspirin (325 mg). A sensitivity analysis in the present study using enteric coating and aspirin as time dependent variables also showed that the enteric coating did not modify the outcome response to aspirin dose over time. While it is not known what level of aspirin-induced platelet inhibition is needed to decrease cardiovascular events, this finding implies that enteric coating did not limit the effectiveness of aspirin in providing cardiovascular protection in this patient population.

Acid-reducing medications, such as proton pump inhibitors and histamine type 2 receptor antagonists, used to buffer aspirin within the stomach, have been shown to affect dissolution of the enteric formulations of aspirin by altering the pH, composition, and ionic strength in the stomach.^19^ The effect on the ability of enteric-coated aspirin to inhibit platelet aggregation has been mixed, with 1 study^20^ showing no effect with lansoprazole and another study^21^ showing reduced platelet aggregation when pantoprazole was given with enteric-coated aspirin. Although the pharmacodynamics suggest that enteric-coated aspirin may be less effective than uncoated aspirin, there are no studies, to our knowledge, addressing the question of whether combining ARM with enteric-coated aspirin will result in more adverse clinical outcomes. In the present analysis, we found that the primary efficacy end point of death from any cause, hospitalization for MI, or hospitalization for stroke had similar cumulative incidences reported across 26.2 months in both the enteric-coated aspirin and uncoated aspirin cohorts, regardless of whether patients were also taking ARM.

In terms of the safety profile, enteric coating has been postulated to have better protection against GI tract bleeding and other major bleeding events.^22^ A double-blind placebo-controlled crossover trial by Hawthorne et al^23^ showed that enteric coating virtually eliminated gastric mucosa toxic effects compared with nonenteric coating at both high and low doses of aspirin, with similar inhibition of prostaglandin synthesis. Since then, a follow-up study has shown that while this may be true with short-term use, long-term administration of both enteric-coated aspirin or uncoated aspirin causes gastric complications and the development of erosion.^24^ A meta-analysis demonstrated less-than-convincing effects of GI tract protection with enteric-coated aspirin.^25^ However, the point estimate for major bleeding with enteric-coated aspirin in the present study showed an 18% relative risk reduction, although the 95% CI was wide, so that a reduction in bleeding with enteric-coated aspirin cannot be reliably excluded. Similar to the lack of an association of enteric coating with the efficacy of aspirin, no significant difference in safety outcomes was observed with enteric-coated aspirin compared with uncoated aspirin in this analysis. We found that more participants randomly assigned to receive the lower dose aspirin (81 mg) took enteric-coated aspirin, while more participants randomly assigned to receive the 325-mg dose used uncoated aspirin. As these data were based on data at randomization and not on the actual drug taken throughout the study, there is no discernable reason for this difference. The rationale behind this finding could be that more patients were given low-dose aspirin because of the perception that low-dose aspirin is less irritating to the GI tract than high-dose aspirin.

When major bleeding was evaluated within formulation cohorts, there was no association with aspirin dose among participants using uncoated aspirin and a small but significant association among patients using enteric-coated aspirin at 325 mg compared with 81 mg. The lack of bleeding difference in the participants using uncoated aspirin may account for the apparent lack of difference in major bleeding seen in the overall ADAPTABLE trial. The results of the present study for the enteric-coated aspirin cohort differed from those of other trials in which no difference in major bleeding was noted; however, in those trials, formulation of aspirin used was not mentioned.^26,27^

It has been proposed that the coadministration of ARM with enteric-coated aspirin would decrease the frequency of bleeding. However, we were unable to demonstrate a clinical interaction between ARM and the presence of enteric coating associated with either major bleeding or GI tract bleeding in this analysis.

### Limitations

This study has limitations. First, formulation of aspirin was participant determined, and participants reported only at baseline, with only 70.1% of the participants reporting the formulation of the aspirin they were using. The determination of whether a participant took the reported medication or was adherent to it was not captured after baseline. This assumption of which formulation of medication the participants were taking is an act of trust, as patients could have switched during the study on their own or with the advice of their physician. This would lead to a false conclusion if a large number of participants switched formulations of medication during the study. Second, the use of enteric-coated aspirin was not part of the randomization stratification; thus the data must be treated as though from an observational study. Because the participants decided which formulation of aspirin they would use, there may be unknown confounders leading to the choice of aspirin formulation used by the participant. For instance, a well-known leading brand of aspirin has a large number of their aspirin products, particularly cardiovascular products, packaged with the phrase “safety coating” included. However, adjustments were applied to the data to attempt to control for known confounders although unknown confounders may still affect the results. Third, there are no pharmacokinetic or pharmacodynamic data demonstrating whether enteric coating had any association with serum aspirin levels or platelet inhibition. Fourth, we did not collect detailed data on bleeding outcomes. It is unknown whether minor GI tract or other bleeding was reduced by either enteric coating or the dose of aspirin consumed. Finally, despite attempts to enroll a diverse population of patients with ASCVD, the enrollment of women and historically underrepresented minority groups lagged, so generalizability of the results remains questionable.

## Conclusions

In this post hoc secondary analysis of data from the ADAPTABLE randomized clinical trial, there were no significant differences in the primary effectiveness or safety end points between enteric-coated aspirin and uncoated aspirin among participants with established ASCVD although a reduction in bleeding with enteric-coated aspirin cannot be reliably excluded. More research is needed to confirm whether enteric-coated aspirin formulations or newer formulations will improve ischemic and bleeding outcomes among patients with ASCVD.

## Notes

Supplement 1. **Trial Protocol.** ADAPTABLE

Supplement 2. **Trial Protocol.** Enteric-Coated Aspirin Portion of ADAPTABLE

Supplement 3. **eTable 1.** Demographic and Presenting Characteristics, Medical History, and Medications by Aspirin Formulation Regardless of Dose **eTable 2.** Clinical End Points by Aspirin Formulation [Adjusted] Regardless of Dose **eTable 3.** Interaction of Acid Reducing Medication and Aspirin Formulation Type Regardless of Aspirin Dose on the Cumulative Incidence of Study End Points **eTable 4.** Event Rates of Study End Points by Formulation Type and Time-Dependent Reported Aspirin Use **eFigure 1.** Cumulative Incidence of the Primary End Point (All-Cause Death, Myocardial Infarction, or Stroke) by Enteric-Coated Aspirin and Uncoated (Plain Coated) Aspirin Regardless of Aspirin Dose **eFigure 2.** Cumulative Incidence Function of Major Bleeding by Enteric-Coated Aspirin and Uncoated (Plain Coated) Aspirin Regardless of Aspirin Dose

Supplement 4.
Data Sharing Statement
