# Supplementary material for: Effectiveness and Safety of Enteric-Coated vs Uncoated Aspirin in Patients With Cardiovascular Disease: A Secondary Analysis of the ADAPTABLE Randomized Clinical Trial
Source: JAMA Cardiol. 2023 Oct 4;8(11):1061–9. doi: 10.1001/jamacardio.2023.3364 (PMC10551818; doi:10.1001/jamacardio.2023.3364)
Supplement: Supplement 1. — Trial Protocol. ADAPTABLE [file jamacardiol-e233364-s001.pdf]

Aspirin Dosing: A Patient-Centric Trial Assessing Benefits and Long-term Effectiveness (ADAPTABLE) Study Protocol

Version 1.0

The protocol and statistical analysis plan for the overall study was previously published in the New England Journal of Medicine as a supplement to Jones WS, Mulder H, Wruck LM, et al. Comparative Effectiveness of Aspirin Dosing in Cardiovascular Disease. N Engl J Med. 2021 May 27;384(21):1981-1990. doi: 10.1056/NEJMoa2102137. Epub 2021 May 15. PubMed PMID: 33999548.

Table of Contents

Signature Page iv

I. Study Overview & Goals 1

IA. Study Rationale 1

I.A.1. ADAPTABLE Study Design 2

I.B. Study Aims 3

I.B.1. Comparison to DCRI Standard Metrics for Clinical Trials 4

I.B.2. PCORnet as a Network 4

I.B.3. CDRN Experience 4

I.B.4. Patient Experience 4

II. Background and Significance 4

II.A. Significance of Aspirin Dosing: A Global Perspective 4

II.B. Optimal Aspirin Dosing in the Context of PCORnet: A New Model 6

II.C. Potential Impact of Proper Dosing of Aspirin 7

II.C.1. Benefit of Aspirin as Preventive Therapy 7

II.C.2. Aspirin Dose and Clinical Outcomes 7

II.C.3. Aspirin and Platelet $P2Y_{12}$ Inhibitors 9

II.C.4. Systematic Reviews and Mechanistic Insights into Aspirin Dosing 11

II.D. Aspirin: Mechanism, Clinical Benefit, and Adverse Effects 12

II.D.1. Mechanism of Aspirin Effect on ASCVD 12

II.D.2. Aspirin Intolerance and Dose-Related Risks of Aspirin Therapy 13

II.E. Modifiers of Aspirin Dose 15

II.E.1. Aspirin, Minorities, and Other Subgroups 15

II.E.2. Other Issues 15

III. Research Design and Methods 16

III.A. Study Aims 1 to 3: Aspirin Dosing 16

III.A.1. Enrollment and Eligibility 16

III.A.2. Solicitation of Participation 17

III.A.3. Study Design and Procedures 21

III.A.4. Data Security and Back-up Procedures 27

III.A.5. Endpoints and Adverse Events 30

III.A.6. Summary of Statistical Methods 33

III.A.7. Study Coordination and Monitoring 37

IV. Human Subjects 40

IV.A. Protection of Human Subjects 40

IV.A.1. Human Subjects Involvement and Characteristics 40

IV.B. Inclusion of Women 43

IV.C. Inclusion of Minorities 43

IV.D. Inclusion of Children 44

IV.E. Data and Safety Monitoring Plan 44

Appendix: Patient Survey 45

References 47

Signature Page – Not applicable

Version 1.0

I. Study Overview & Goals

IA. Study Rationale

Every year 720,000 Americans have a heart attack, and nearly 380,000 die of atherosclerotic cardiovascular disease (ASCVD).[1] Many of the patients who survive develop heart failure, stroke, and/or other cardiovascular complications. As such, patients with ASCVD and their caregivers suffer from substantial symptomatic, emotional, and functional difficulties. These patients often experience chest pain, shortness of breath, and fatigue, which can lead to significant distress and worsening quality of life. Rates of mental health illness like depression are high among both these patients and their caregivers; rates of depression may approach 66% in post-myocardial infarction (MI) patients.[2-8] Coronary heart disease alone costs the United States $108.9 billion each year.[1] This total includes the cost of health care services, medications, and lost productivity.[1]

Aspirin is a mainstay therapy for patients with ASCVD. Introduced as a medicinal product more than 100 years ago, aspirin significantly reduces ischemic outcomes such as myocardial infarction and stroke in patients with previous cardiovascular events and/or atherosclerosis at a cost of less than a cent per day. However, despite dozens of clinical trials involving more than 200,000 patients, the optimal dose of aspirin—the dose that is most effective in reducing ischemic events in the setting of secondary prevention, balanced by the potential for adverse events such as gastrointestinal bleeding—has not been determined in direct comparative-effectiveness trials. Observational studies and indirect comparisons of different doses of aspirin have yielded conflicting results. Although most studies have found that lower-dose aspirin is associated with less bleeding, these studies have provided contradictory evidence regarding the comparative effectiveness of low vs higher-dose aspirin in reducing ischemic events. Additional evidence raises the possibility that patients with different underlying characteristics may benefit most from different doses of aspirin.

To identify the optimal dose of aspirin for secondary prevention in patients with ASCVD, we propose a pragmatic clinical trial in which 20,000 patients who are at high risk for ischemic events will be randomly assigned in a 1:1 ratio to receive an aspirin dose of 81 mg/day vs. 325 mg/day. Study participants will be enrolled over 24 months. Maximum follow-up will be 30 months. The primary endpoint is a composite of all-cause death, hospitalization for MI, or hospitalization for stroke. The primary safety endpoint is hospitalization for major bleeding with an associated blood product transfusion.

The ADAPTABLE trial study design is shown in the schematic below.

I.A.1. ADAPTABLE Study Design

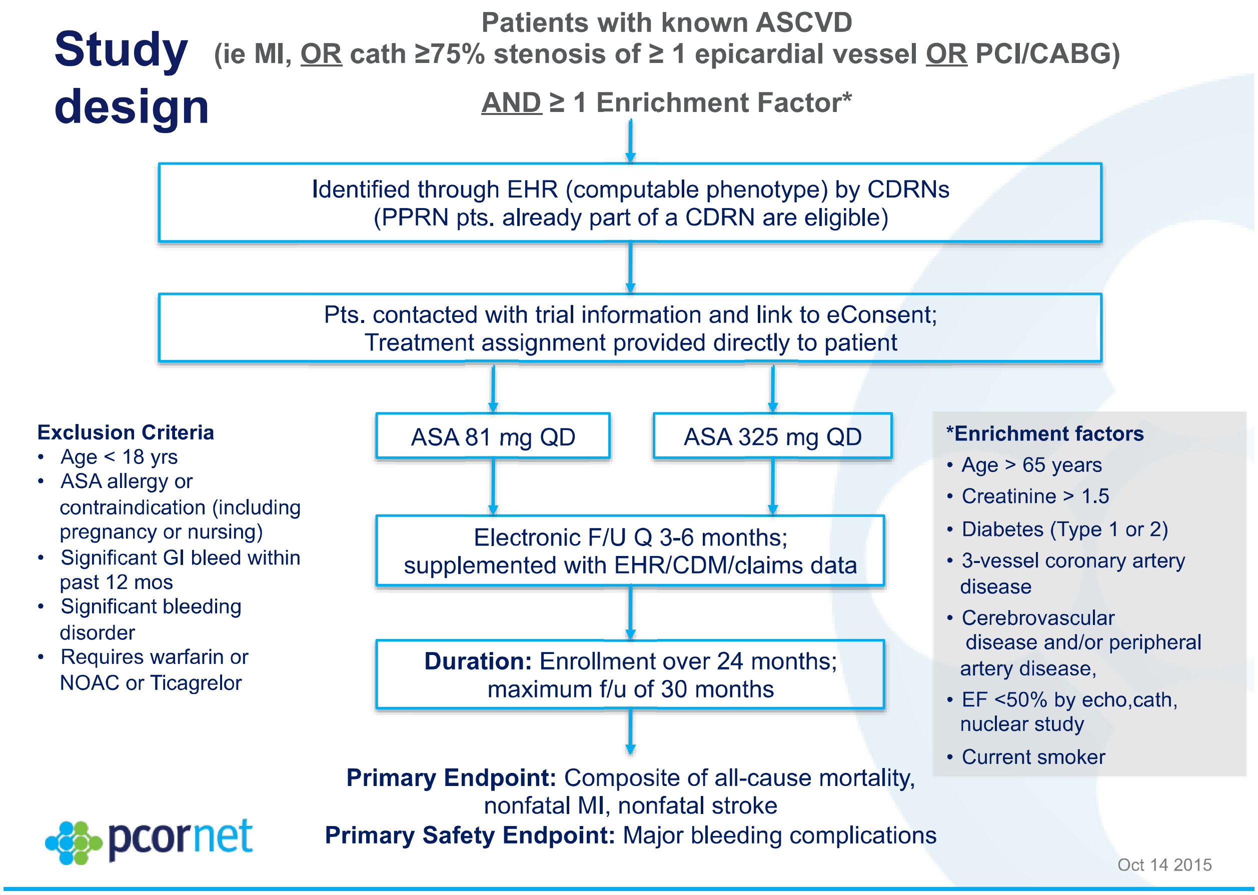

Enrollment and follow-up of study participants will be conducted using highly streamlined methods, with electronic health record (EHR) data organized according to the recently developed PCORnet Common Data Model (CDM) format and stored in a PCORnet DataMart,[*] complemented where possible by existing data sources (Medicare claims data) and patient reported outcomes. Additional information will be collected via streamlined forms to be completed by participants either by Internet if they are able to access the Internet or by the Call Center at the Duke Clinical Research Institute (DCRI). This project constitutes the initial randomized comparative-effectiveness trial conducted by the National Patient-Centered Clinical Research Network (PCORnet; http://www.pcornet.org/).[9]

---

[*] Please note: throughout this protocol, the term “Common Data Model” (CDM) is used to refer both to the format used for standardizing and organizing information, and also as shorthand for EHR data that are extracted and stored using the CDM format within the PCORnet DataMart.

This trial will incorporate several essential aspects of the new genre of patient-centered comparative effectiveness trials:

1. By using existing data sources to gather baseline characteristics and a combination of existing data and patient-reported outcomes during follow-up, the trial will answer this critical question at a relatively low cost.
2. An Internet portal will enable the trial to collect and monitor data and enable mutual learning by both patients and clinicians, capitalizing on the frequent use of the Internet by the American public and clinicians.
3. The trial will not have a placebo control, but instead will provide all patients with active treatment at different doses, with monitoring to balance benefit and risk.
4. Patient-reported outcomes will be collected.
5. The evolving PCORnet infrastructure will be used to streamline administrative aspects of the trial, including centralization of institutional review board (IRB) functions and contracts, electronic consent and use of EHR data standardized into the CDM format.

I.B. Study Aims

We have defined the following specific aims for this study:

- **Aim 1:** To compare the effectiveness of two daily doses of aspirin (81 mg and 325 mg) in reducing a composite endpoint of all-cause death, hospitalization for nonfatal MI, or hospitalization for nonfatal stroke in high-risk patients with a history of MI or documented atherosclerotic cardiovascular disease (ASCVD). Secondary endpoints will be the components of the composite primary endpoint as well as coronary revascularization procedures (percutaneous coronary intervention [PCI] or coronary artery bypass grafting [CABG] performed during study follow-up. The primary safety endpoint will be hospitalization for major bleeding complications with an associated blood product transfusion.
- **Aim 2:** To compare the effects of aspirin in selected subgroups of patients, including women vs men, older vs younger patients, racial minority patients vs white patients, patients with vs. without diabetes, and patients with vs. without chronic kidney disease (CKD)
- **Aim 3:** To develop, refine, and evaluate the infrastructure for PCORnet to conduct multiple comparative-effectiveness trials in the future. This aim will be accomplished with a “phased-in” approach (as previously described) that will allow for an initial testing of the PCORnet infrastructure followed by adjustments to the trial operational plan to most efficiently accomplish Aims 1 and 2. Also, during the first year of the trial, we will be carefully monitoring the recruitment and enrollment patterns within and across CDRN’s and will be providing regular feedback reports to each CDRN to promote consistency in the recruitment practices. Potential metrics to evaluate the success of ADAPTABLE are listed below and will be finalized with the PCORnet leadership in the context of other performance measures in development PCORnet-wide:

I.B.1. Comparison to DCRI Standard Metrics for Clinical Trials

- Time to IRB approval
- Time to contract approval
- Time to first site activation
- Time to first patient enrolled
- Recruitment rate
- Retention
- Withdrawn consents
- Drug discontinuation
- Lost to follow-up
- Missed study contacts
- Data quality

I.B.2. PCORnet as a Network

- Ability to support widespread screening, contact, enrollment, and follow-up of patients across the networks

I.B.3. CDRN Experience

- Administrative simplicity (i.e. IRB share model, contracts)
- Participation, engagement and leadership

I.B.4. Patient Experience

- Assess electronic consent process and patient experience
- Evaluate experiences of participating patients

II. Background and Significance

II.A. Significance of Aspirin Dosing: A Global Perspective

ASCVD that leads to ischemic events represents the leading cause of death, morbidity, and disability.[10] Despite remarkable progress in prevention and treatment for atherosclerosis, ASCVD is expected to be an even more prominent cause of death and disability over the next 30 years.[11] In high-income countries, the major factors contributing to this expansion are the aging of the population coupled with increases in incidence of obesity, diabetes, and sedentary lifestyle. Despite declining age-specific disease rates, the total disease burden increases as ASCVD eventually affects a larger population of older adults. In economically developing countries, a major epidemic of atherosclerosis is occurring, concentrated in younger age groups and presumably due to increasing tobacco use as well as obesity and diabetes arising from Westernization of diets and lack of exercise.[12]

The development of new biological and technological approaches to treating ASCVD is exciting, but maximizing the use of an inexpensive yet effective therapy shows more promise for reducing death and disability on a global scale. Numerous clinical trials have shown the clinical benefit of aspirin vs placebo in reducing vascular events in patients with a history of ASCVD or a specific cardiovascular event, but the best dose of aspirin for the general population with ischemic heart disease has not been determined. Considering the burden of ASCVD and that the population affected by it is growing rapidly, identifying the optimal aspirin dose will save lives and prevent ischemic and bleeding events at a global scale.

For example, based on recent evidence suggesting a reduction in ischemic events with lower doses of aspirin, the odds ratio for an event with an aspirin dose of 81 mg/day vs 325/day would be 0.84 (95% confidence interval [CI], 0.64–1.1).[13-15] If the rate of death, MI, or stroke in a prospective clinical trial over ~18 months of treatment was 8% with 325 mg of aspirin (based on contemporary trials of aspirin use in patients with ischemic heart disease),[13,16,17] then the expected event rate with 81 mg would be 6.8% (95% CI, 5.3–8.7), or ~12 events prevented for every 1000 patients treated. Given the magnitude of the global burden of ischemic heart disease, a 1.2% absolute reduction in events achieved simply through optimal aspirin dosing would be of tremendous importance to public health.

Until recently, aspirin dosing patterns after acute MI in the United States were uncertain. A 2014 analysis of the National Cardiovascular Data Registry's (NCDR's) Acute Coronary Treatment and Intervention Outcomes Network (ACTION) Registry-Get with the Guidelines (GWTG) examined aspirin dosing in 221,199 patients with acute MI (both ST-elevation MI and non-ST-elevation MI) from 525 US hospitals.[18] Between January 2007 and March 2011, 61% of patients were discharged on 325 mg of aspirin, 36% on 81 mg, and 4% on other doses. The rate of use of 325 mg of aspirin at discharge was 73% in patients who underwent PCI vs. 45% in patients managed medically (i.e., without invasive revascularization). When aspirin was used concomitantly with thienopyridine and warfarin, a 325-mg dose was used in 44% of patients. Even among patients who experienced major in-hospital bleeding, 57% received the 325-mg dose. The relatively high rate of use of this dose, even in patients at high risk of bleeding, and the 25-fold variation in the rate of use of the 325-mg aspirin dose across participating centers are surprising and likely reflect uncertainty regarding appropriate aspirin dosing.[18]

Further details on aspirin dosing patterns and the impact of high- vs. low-dose aspirin among patients with acute MI undergoing PCI in the United States from 2010-2012 were recently published from the TRANSLATE ACS registry.[19] Among 10,213 patients, 6,387 (62.6%) received high-dose (325 mg) aspirin at hospital discharge with substantial variability across the 228 hospitals in the analysis (median hospital-level frequency of high-dose aspirin use was 70%). The adjusted risks of ischemic outcomes (death, MI, stroke, or unplanned revascularization) and bleeding requiring hospitalization through 6 months were similar with high- vs. low-dose (81 mg) aspirin. However, approximately 35% of patients discharged on high-dose aspirin were switched to low-dose aspirin within 6 months. These non-randomized findings, coupled with the findings from the ACTION Registry-GWTG analysis, highlight the substantial variability in aspirin dosing patterns in the United States for patients with ASCVD who have experienced a recent acute MI event and directly point to the need to do an adequately powered, large-scale trial of low- vs.

high-dose aspirin to determine the most effective dose of aspirin for the secondary prevention of ASCVD.

In the United States, the 2010 death rates attributable to coronary heart disease, stroke, and other cardiovascular diseases were 113.6, 39.1, and 82.7 per 100,000, respectively.[10] Globally, given the rapidly increasing burden of ASCVD and limited healthcare resources, particularly in lower-income countries, a similar benefit from identifying the best dose of aspirin for treating the general population with ischemic heart disease could translate to as many as 88,800 fewer deaths from ASCVD annually and would prevent ~145,000 deaths in 2020.[20] In the United States alone, this would mean ~19,000 fewer deaths and MIs each year without employing new treatments or technology and with no additional healthcare expenditures.[10]

In addition to defining the best dose of aspirin from the population perspective, the subgroup analyses and model-based analyses of heterogeneity of treatment effect planned for this proposed trial will allow further insights into refinement of aspirin dosing at the patient level. Such knowledge could further enhance the benefit derived from aspirin treatment.

II.B. Optimal Aspirin Dosing in the Context of PCORnet: A New Model

Although the primary aim of this study is to determine the optimal dose of aspirin for secondary prevention of ASCVD, it also represents the initial use of a transformative approach to developing a new and efficient interactive model for designing and implementing clinical trials that aim to compare the effectiveness of therapies already in use in clinical practice (comparative-effectiveness research, or CER) using methods centered on the needs and experiences of patients. Because we live in an era in which the number of effective (or potentially effective) therapies far exceeds our ability to evaluate them in prospective clinical trials using current methods, we face an urgent need to develop an approach to CER trials that can greatly reduce the cost of trials while maintaining the quality, reproducibility, and generalizability of the research. By using existing data from EHRs organized into the CDM developed by PCORnet and derived from the FDA's Sentinel project,[21] the trial will develop initial experience with the use of the CDM to supplant costly and time-consuming data collection approaches that are used with traditional clinical trials. By following the majority of patients on the Internet and collecting minimal data directly from them, we can avoid the costs incurred by non-clinically indicated research visits, lengthy case report forms (CRFs), and extensive site management operational approaches.

We believe that a more efficient and less expensive model for trials can be developed that could be extended to more experimental comparisons. However, working through the issues of informed consent, data validation, events ascertainment, and compliance assessment will require acceptance of novel approaches to statistical sampling and "quality by design" principles[22] as well as communication with patients. Amid increasing concerns about patient privacy and research integrity, such an approach to trial efficiency would be difficult to pilot with untested therapies. Because this trial will test only doses of aspirin that are considered relatively safe and are widely used in current clinical practice, it presents a critically and globally important clinical issue with which to develop these new methods.

A trial designed to use existing data resources almost exclusively (supplemented by Internet interaction with research participants and telephone contact from the DCRI Call Center for those without internet access) offers many potential benefits. For example, clinicians will not be burdened with extensive data collection forms and cumbersome consent and contracting procedures. In addition, the patient portal will serve as the primary mechanism for follow-up, with routine data entry by the patients themselves providing a concise set of patient-reported outcomes (PROs).

For physicians and other clinicians, application of the PCORnet CDM format and the patient portal to clinical trials could broaden awareness and participation and, at the same time, aid in conduct of the study. For example, trial enrollment and follow-up could be automated with use of the CDM and patient portal at any time, eliminating or greatly reducing the need for traditional methods such as telephone or postal mail contact, or extended in-person clinic visits. Instead, valuable clinician-patient interaction time can be focused on clinical care and answering questions that arise.

Finally, the platform created by this trial will unite a broad and diverse community of patients and their physicians around the common goal of refining the evidence underlying an existing therapy (aspirin) to maximize its benefit relative to risk. By integrating direct physician and patient participation in the examination of the relationships among clinical outcomes in response to various aspirin doses, this platform will also produce far greater global benefit than the introduction of many other "high-tech" approaches.

II.C. Potential Impact of Proper Dosing of Aspirin

II.C.1. Benefit of Aspirin as Preventive Therapy

Aspirin has a significant impact on the risk of vascular events in patients with known atherosclerosis. The Antiplatelet Trialists' Collaboration reported a 20%–40% reduction in the risk of death, MI, or stroke for study participants taking aspirin.[13] This benefit was clear for patients with coronary artery disease or cerebrovascular disease and for those with MI, unstable angina, transient ischemic attack (TIA), or stroke. More recently, aspirin therapy was associated with reduced long-term mortality among 6,174 patients undergoing stress echocardiography to evaluate known or suspected ASCVD (hazard ratio [HR] over ~3 years, 0.67; 95% CI, 0.51–0.87; *P*=0.002).[23] After adjustment for the propensity to use aspirin and other possible confounding variables, aspirin use remained associated with a lower risk of death (HR, 0.56; 95% CI, 0.40–0.78; *P*<0.001). Patient characteristics associated with the greatest reductions in mortality included advanced age, known ASCVD, and impaired exercise capacity. These results were recently replicated in a broad assessment of aspirin effectiveness across racial, ethnic, and sex subgroups.[24]

II.C.2. Aspirin Dose and Clinical Outcomes

Despite uncertainties about optimal aspirin dosing, consensus has developed regarding recommendations for aspirin dosing between 75–325 mg daily in patients with ASCVD. This practice has been driven primarily by commercial availability, physician preference, and concerns about adverse effects such as abdominal discomfort and gastrointestinal bleeding associated with higher doses.

Although this dose range has developed empirically over time, attempts to balance clinical benefit with adverse side effects largely reflect indirect comparisons performed in various clinical settings.

Few direct comparisons of different doses of aspirin have been performed, and their results have been inconclusive. The only large, direct prospective study performed to date (OASIS 7-CURRENT)[25] evaluated outcomes among patients with Acute Coronary Syndromes (ACS) over the first 30 days only. The results were complex because the trial used a factorial design of high- and low-dose aspirin with high- and low-dose clopidogrel starting at the time of the index ACS event.[25,26] Although the factorial analysis showed the most favorable outcomes for the combination of high-dose aspirin and high-dose clopidogrel, the results were not definitive, and a variety of different interpretations have been offered by experts.[27] Thus, while indirect evidence exists for dose-dependent efficacy of aspirin in preventing vascular events, it is equally clear that no adequately sized randomized trials have addressed this issue, particularly in patients with established ASCVD who are receiving long-term treatment for secondary prevention. The suggestive (but not definitive) data supporting lower aspirin dose emphasize a clear need for larger randomized studies of aspirin dosing in ASCVD.

Various aspirin doses, even as low as 30 mg/day, have been shown to be effective in preventing vascular events.[28] A trial of unstable angina patients found a dose of 75 mg/day to be effective in reducing recurrent vascular events,[29] and the European Stroke Prevention Trial found a benefit of 25 mg twice daily in preventing stroke or death in high-risk patients.[30]

Varied trial results have underscored the uncertainty about aspirin dosing in secondary prevention. A study of secondary prevention after TIA compared 300 mg/day with 1200 mg/day and found no difference in efficacy.[31] Similarly, the Dutch TIA prevention study found no difference in efficacy between 30 and 283 mg of aspirin per day.[28] A trial comparing aspirin doses after carotid endarterectomy found a lower risk of the composite of death, MI, or stroke with daily doses of 81 or 325 mg/day versus 650 or 1300 mg/day.[32] This study contradicted earlier findings of a lower event rate with doses of ≥650 mg versus ≤325 mg per day for prevention of perioperative stroke.[33,34]

In what had been the largest experience for many years, the Antiplatelet Trialists' Collaboration's systematic review of 11 trials of antiplatelet therapy, there was no apparent dose-response relationship of aspirin in secondary prevention of cardiovascular outcomes.[13] When the investigators expanded their meta-analysis to include subsequent trials of antiplatelet therapies,[14] they found that compared with no aspirin therapy, high doses of aspirin (500–1500 mg/day) were not clearly more effective in reducing ischemic vascular events than doses of 75–150 mg/day. Specifically, the proportional reduction in vascular events was 19% with 500–1500 mg/day, 26% with 160–325 mg/day, and 32% with 75–150 mg/day **(Table 1).**

Table 1. Aspirin Dosing and Ischemic Events: ATC

		Ischemic Event Rate		
Aspirin Dose	Trials (n)	Aspirin	Control	Relative Reduction
500–1500 mg	34	14.5%	17.2%	19%
160–325 mg	19	11.5%	14.8%	26%
75–150 mg	12	10.9%	15.2%	32%
<75 mg	3	17.3%	19.4%	13%
Any dose	65	12.9%	16.0%	23%

II.C.3. Aspirin and Platelet $P2Y_{12}$ Inhibitors

Platelet $P2Y_{12}$ inhibitors (ticlopidine, clopidogrel, prasugrel, ticagrelor) reduce the risk of major adverse cardiovascular events (MACE) when added to aspirin (termed dual anti-platelet therapy – DAPT) in patients with ST-segment-elevation myocardial infarction (STEMI), acute coronary syndromes (ACS), and percutaneous revascularization procedures. The recommended duration of DAPT for these indications has been approximately 1 year, but the recently completed DAPT and PEGASUS Trials[24,35] suggest that extended durations of dual antiplatelet therapy for up to 3 years after PCI with coronary stent placement or prior MI provide long-term benefit. Observational comparisons indicate that lower-dose aspirin may be associated with better outcomes when DAPT is used. However, no sizable randomized comparisons of aspirin dosing are available wherein aspirin was used in combination with $P2Y_{12}$ inhibitors except for the CURRENT-OASIS-7 trial, as previously mentioned.[25]

Thienopyridine platelet inhibitors have been in clinical use for more than 2 decades. Ticlopidine, the prototype, was shown to reduce ischemic events compared with placebo in a series of clinical trials,[36,37] to be marginally superior to aspirin in one trial in cerebrovascular disease,[38] and to provide additive benefit to aspirin after percutaneous coronary intervention (PCI).[39] Clopidogrel is structurally similar to ticlopidine but is associated with substantially fewer serious adverse events. Most notably, ticlopidine has been associated with the development of neutropenia, thrombocytopenia, and thrombotic thrombocytopenic purpura;[40] such events have not been associated with clopidogrel use in large clinical trials.

The first major study of clopidogrel was the CAPRIE trial, which showed a modest but measurable benefit over aspirin (RR reduction, 8.7%; *P*=0.043) in the secondary prevention of ischemic stroke, MI, or vascular death among patients with vascular disease.[17]

The CURE trial compared the effectiveness of DAPT with aspirin and clopidogrel versus aspirin alone in patients with acute coronary syndromes (ACS).[41] At a mean follow-up of 9 months, there was a 22% relative reduction in the composite endpoint of death, MI, or stroke with combination therapy (9.3% with clopidogrel + aspirin vs 11.4% with aspirin alone; *P*<0.001). Although major bleeding occurred significantly more often with combined therapy (3.7% with aspirin + clopidogrel; 2.7% with aspirin alone; *P*=0.001), there was no excess in major bleeding in the clopidogrel group after the first 30 days, suggesting that most of the risk was related to early revascularization procedures. Although CURE was not a formal study of secondary prevention (follow-up period was 1 year) and aspirin therapy ranged from 75–325 mg/day, the trial found benefit during 1 year of follow-up. Of note, aspirin dosing in the CURE trial was left to the treating physician's discretion and was not part of the randomized treatment assignment. With increasing aspirin doses, however, trends toward higher rates of ischemic and bleeding events were observed for patients in both study arms (**Table 2**), with the lowest rates of bleeding and ischemic events observed in patients taking an aspirin dose of <100 mg/day.[41]

Table 2. Effect of Aspirin Dosing on Ischemic Events and Bleeding in the CURE Trial

	Death, MI, Stroke				
Aspirin Dose	Clopidogrel +ASA	Aspirin	HR (95% CI)	Life-threatening Bleeding	Any Bleeding
<100 mg (n=1927)	80 (8.5%)	96 (9.7%)	0.86 (0.64–1.16)	1.2%	1.9%
110–161 mg (n=7428)	345 (9.2%)	402 (10.9%)	0.84 (0.73–0.97)	1.7%	2.3%
>200 mg (n=3201)	157 (9.9%)	221 (13.7%)	0.71 (0.58–0.87)	2.5%	3.9%

Conflicting results have now been reported for prasugrel and ticagrelor. The TRITON trial compared clopidogrel vs. prasugrel added to aspirin therapy for patients with ACS undergoing PCI and found no evidence for an interaction between aspirin dose and treatment effect of prasugrel relative to clopidogrel for key outcomes, nor did it find a difference in outcomes as a primary function of aspirin dose.[42] In contrast, an observational analysis of the PLATO trial[43] found that lower doses of aspirin were associated with less bleeding and fewer ischemic events in patients receiving ticagrelor. Further, there was a significant interaction between aspirin dose and the treatment benefit of ticagrelor, which overall reduced total mortality compared with clopidogrel. In the PLATO trial, patients receiving low-dose aspirin had a significant reduction in death and MACE if they were randomized to ticagrelor, whereas patients on high-dose aspirin fared equally well with clopidogrel and ticagrelor. However, it should be noted that this comparison of the impact of concomitant aspirin doses with the treatment effect of ticagrelor was non-randomized and was subject to a significant amount of bias based upon regional differences in the concomitant aspirin dose across the multiple countries that participated in the PLATO trial. Nonetheless, the approval of ticagrelor for the treatment of ACS by the Food and Drug Administration (FDA) in the United States incorporated a "black box" warning for avoiding aspirin doses

>100 mg together with ticagrelor (http://www.fda.gov/NewsEvents/Newsroom/PressAnnouncements/ucm263964.htm).

In summary, the appropriate dose of aspirin in conjunction with a $P2Y_{12}$ inhibitor is unknown. The ADAPTABLE Trial will investigate this issue by conducting a subgroup analysis according to the concomitant use of a $P2Y_{12}$ inhibitor and will allow patients to be included in the study who are treated with either clopidogrel or prasugrel, but patients treated with ticagrelor will be excluded given the FDA "black box" warning for the use of high-dose aspirin with ticagrelor.

II.C.4. Systematic Reviews and Mechanistic Insights into Aspirin Dosing

To evaluate the influence of aspirin dosing, a random effects model was used for combining data from 11 randomized, placebo-controlled trials of aspirin in the Antiplatelet Trialists' Collaboration's dataset of 14,810 patients.[15] This modeling technique allows adjustments for patient populations and time trends, two limitations inherent in indirect comparisons. Aspirin doses evaluated in this meta-analysis, which examined endpoints including death, acute MI, and stroke (but not bleeding), ranged from 50 mg to 1.5 g daily. Overall, compared with placebo, aspirin resulted in significant reductions in death and death or MI, yet there was a significant decrease in estimated effectiveness as aspirin dose increased (OR for an event: 1.14 with each doubling of aspirin dose; P=0.007). The analysis also suggested that the study population influenced the effect of aspirin, with a greater benefit observed among patients with unstable angina vs MI. For the post-infarction subgroup, the OR was 0.76 (95% CI, 0.66–0.88); for the unstable angina subgroup, the OR was 0.55 (0.43–0.71). Such findings also imply a need for larger randomized comparisons of aspirin dosing in patients with ASCVD.

Although recent observational studies using indirect or nonrandomized comparisons of outcome by aspirin dose suggest that lower doses of aspirin are associated with fewer ischemic events, the possibility remains that in a randomized comparison, higher doses of aspirin may be more favorable. For example, if we consider that aspirin resistance, as defined by elevated urinary thromboxane B_2 levels, is associated with worse outcomes and that aspirin 81 mg/day provides less effective thromboxane suppression than aspirin 325 mg/day,[44] we might expect that the latter dose would be associated with better clinical outcomes.

Further, if we postulate that, given its relatively weak antiplatelet effects, an important mechanism of aspirin in prevention of ischemic events is via its anti-inflammatory properties resulting from cyclooxygenase (COX)-2 suppression of platelets or inflammatory cells, similar questions arise about the expected optimal dose. Because 1) aspirin is ~50–100 times more potent in inhibiting platelet COX-1 than monocyte-derived COX-2, and 2) inhibition of COX-2–dependent processes (e.g., inflammation) require larger doses of aspirin (because nucleated cells rapidly resynthesize the enzyme), higher aspirin doses may be required for efficacy.[45] Therefore, if an anti-inflammatory effect of aspirin is important in its ability to prevent ischemic events, then we might expect daily aspirin doses of 162 or 325 mg to be more effective than 81 mg.

In addition, recent research has found alternative pathways for aspirin's effect on platelet aggregation.[46] These pathways may account for the need for different doses in various individuals and subgroups of patients.

These theoretical and mechanistic arguments highlight the need for a randomized comparison of the effect of aspirin dosing on clinical outcomes that avoids any potential for selection bias toward lower-risk patients receiving lower aspirin doses from their physicians. It also highlights the possible value of mechanistic substudies as a part of this effort to advance our understanding of the factors that identify and modify aspirin response and their relationships with clinical outcomes.

II.D. Aspirin: Mechanism, Clinical Benefit, and Adverse Effects

II.D.1. Mechanism of Aspirin Effect on ASCVD

Aspirin's putative mechanism of action for preventing cardiovascular events is through irreversible inhibition of platelet COX-1, which prevents the conversion of arachidonic acid to prostaglandin H2—the immediate precursor to thromboxane A2 (TXA2), a potent platelet agonist. Until recently, most experts assumed that the benefit of aspirin solely reflected the inhibition of platelet aggregation. Low-dose aspirin (~80 mg/day) is sufficient to maximally inhibit platelet COX-1 and COX-1-dependent measures of platelet aggregation; higher aspirin doses do not produce additional COX-1 inhibition.[47] Consistent with this finding, selective TXA2 receptor inhibitors have shown no benefit compared with either aspirin or placebo.[48,49]

With 325 mg/day aspirin dosing, there appear to be additional effects on inhibiting measures of platelet function, particularly from the perspective of non-COX-1 dependent assays (e.g., collagen- and high-dose ADP-induced platelet aggregation).[47] The consequences of this additional, apparently non-COX mediated platelet inhibition with 325 mg/day dosing on clinical outcomes are unknown. However, residual platelet aggregation of non-COX1 dependent platelet aggregation in aspirin treated patients is associated with future cardiovascular events.[50] Further it is clear that additional platelet inhibition in excess of that produced by low-dose aspirin either through inhibition of platelet P2Y12[41,51] or PAR1[52,53] receptors can lower the risk of cardiovascular events in high-risk patient populations. In conclusion, aspirin has dose-dependent effects on platelet inhibition that go beyond platelet COX-1 inhibition and which have unknown clinical consequences.

Aspirin is also a weak inhibitor of COX-2, which is expressed in immune function and endothelial cells. Full suppression of COX-2 activity by aspirin has been estimated to require aspirin doses >500 mg/day. Beyond COX1/2 inhibition, it is increasingly clear that aspirin possesses several additional properties that do not appear to depend on COX inhibition. For example, aspirin (and its stable metabolite salicylate) are well-known to produce dose-dependent inhibition of elements of the proinflammatory NFkB pathway: NFKB,[54,55] IkB phosphorylation,[56] IkB kinase,[57] as well as other cellular kinases.[58,59] Aspirin also influences erythrocytes,[60] endothelial cells,[61] endothelial progenitor cells,[62] and inflammatory markers (CRP, interleukin-6, and macrophage colony stimulating factor (MCSF).[63] In addition, omics-based assays are now illuminating the full suite of pathways affected by aspirin, many of which are not predicted

based on aspirin's putative mechanisms of action: acetylation of proteins beyond COX,[64] alterations in fatty acid and amino acid metabolism,[65] and changes in platelet RNA/protein content.[66] Although it is unknown to what extent these apparent non-COX effects of aspirin are dose-dependent, it is clear that aspirin interacts with a wide array of diverse biologic pathways that may underlie its beneficial effects on cardiovascular disease.

In contrast, aspirin may also interact with prothrombotic pathways. In addition to serving as a precursor to TXA2, PGH2 produced by COX1/2 can be metabolized by vascular endothelial cells to produce prostacyclin (PGI_2). Although TXA_2 promotes platelet aggregation and vasoconstriction, PGI_2 inhibits platelet aggregation and induces vasodilation. Aspirin causes dose-dependent reductions in TXA2 and PGI2 production,[67] thus producing a blend of anti- and prothrombotic effects. No studies to date have directly established that more profound suppression of PGI_2 formation by higher doses of aspirin is sufficient to initiate or to predispose to thrombosis. However, recent studies in healthy volunteers exposed to aspirin 325 mg/day demonstrate that in certain individuals such dosing can lead to a paradoxical *increase* in platelet aggregation despite complete suppression of platelet COX-1.[68] Further, recent studies have associated selective COX-2 inhibitors with unopposed platelet activation and a greater likelihood for thrombotic events.[69] These findings provide mechanistic support for the efficacy of lower aspirin doses in preventing COX-1-mediated TXA_2 production while preserving the antiaggregatory and vasodilatory effects of PGI2. Whether higher aspirin doses will achieve a more beneficial (or potentially harmful) balance of TXA2 vs PGI2 in preventing cardiovascular events is unknown.

II.D.2. Aspirin Intolerance and Dose-Related Risks of Aspirin Therapy

In patients with established ASCVD, the risk associated with aspirin use is quite low compared with the benefit. Only a very small number of patients develop a serious anaphylactic reaction or bronchoconstriction with nasal polyps when they take aspirin.[70,71] The risk of intracranial hemorrhage with chronic aspirin treatment is estimated to be <0.04% per year.[72] A modest number of patients develop serious gastrointestinal bleeding with chronic aspirin therapy, mostly related to the loss of the protective effect of prostaglandins on the gastric mucosa. A larger number of patients have gastrointestinal intolerance with aspirin, but this is often transient and can be overcome in many patients. The absolute risk varies as a function of the trial entry criteria, but none of these major risks exceeds 5 events per 100 patients treated per year.[73,74] Except for patients with pre-existing asthma, no studies have described the risk factors for these complications of aspirin use.

The Clopidogrel versus Aspirin in Patients at Risk of Ischaemic Events (CAPRIE),[17] Coumadin Aspirin Reinfarction Study (CARS),[75] and Sibrafiban versus aspirin to Yield Maximum Protection from ischemic Heart events post acute cOroNary sYndromes (SYMPHONY)[76] trials have provided excellent opportunities to understand the incidence of aspirin intolerance and toxicity in patients with vascular disease. Among patients randomized to aspirin therapy alone in CARS, 2.7% discontinued the drug permanently and 7.6% had a dose reduction. In SYMPHONY, which compared aspirin 80 mg twice daily with high and low doses of the oral glycoprotein IIb/IIIa inhibitor sibrafiban, the rate of early aspirin discontinuation was 19.2%, although over half of these patients later reported open-label use. In CAPRIE, 11% of patients randomized to aspirin therapy had to stop the drug because of rash, diarrhea,

indigestion, minor or major bleeding, or abnormal liver-function tests (**Table 3**).[17] Much higher proportions of patients had minor adverse events that did not lead to discontinuation.

Table 3. Adverse Events with Antiplatelet Therapy in the CAPRIE Trial[17]

	Patients Ever Reporting		Severe		Requiring Discontinuation	
	Clopidogrel	Aspirin	Clopidogrel	Aspirin	Clopidogrel	Aspirin
Rash	578 (6.0)	442 (4.6)*	25 (0.26)	10 (0.1)*	86 (0.90)	39 (0.41)*
Diarrhea	428 (4.5)	322 (3.4)*	22 (0.23)	11 (0.1)	40 (0.42)	26 (0.27)
Indigestion/N/V	1441 (15)	1686 (18)*	93 (0.97)	118 (1.2)	182 (1.9)	231 (2.4)*
Any bleeding disorder	890 (9.3)	890 (9.3)	132 (1.38)	149 (1.6)	115 (1.20)	131 (1.4)
Intracranial hemorrhage	34 (0.35)	47 (0.49)	30 (0.31)	41 (0.43)	20 (0.21)	32 (0.33)
GI hemorrhage	191 (2.0)	255 (2.7)*	47 (0.49)	68 (0.71)*	50 (0.52)	89 (0.93)*
Abnormal liver function	285 (3.0)	302 (3.2)*	11 (0.11)	9 (0.09)	22 (0.23)	28 (0.29)

* *P*<0.05. Data are number (%) of patients. N/V = nausea/vomiting; GI = gastrointestinal.

Although the dose-dependent COX inhibition by aspirin suggests that gastrointestinal bleeding can be reduced with lower aspirin doses, no large, prospective randomized study has examined the relationship between lower doses of aspirin and bleeding. The available evidence is equivocal, and much of the available evidence is from an era in which positive reporting bias was common, especially in observational studies.

Despite the limitations of the available literature, a dose-response relationship has been suggested with regard to GI side effects, including discomfort and bleeding. [73,74] The lower risk associated with lower doses is believed to reflect both the differential inhibition of COX-1 on platelets and the differential inhibition of COX-2 in the gastric mucosa. In a case-control study, the odds ratio for hospitalization for a bleeding peptic ulcer was 2.3 with 75 mg of aspirin, 3.2 with 150 mg of aspirin, and 3.9 with 300 mg of aspirin.[73] In another study, higher doses (300–1200 mg/day) showed a consistent dose-response relationship between gastrointestinal bleeding and a need for hospitalization.[77] Conversely, a systematic review that included data from 66,000 patients reported no difference in the incidence of gastrointestinal hemorrhage with doses <163 mg/day vs higher doses.[78] This study did not examine the risk of bleeding with doses <100 mg/day relative to higher doses, which may be important, because prostaglandin synthesis is inhibited with doses >100 mg.

There is no reliable estimate of the risk of fatal gastrointestinal bleeding as a function of aspirin doses below 162 mg/day. A retrospective analysis of antiplatelet therapy in the CURE trial implies lower risks of both life-threatening bleeding and overall bleeding with lower aspirin doses **(see Section II.C.3).** The risk of death, in the presence of clinically detectable bleeding from a gastric ulcer, is estimated to be 0.5%–10%.[79] Considering the available evidence, aspirin doses <100 mg/day may well be associated with less gastrointestinal bleeding, which may in turn translate into a decrease in fatal events.

A large systematic review in 2007[80] found no evidence that doses higher than 81–100 mg/day were better for ischemic events and lower doses seemed to be associated with less bleeding. However, despite the millions of people taking aspirin daily, fewer than 5,000 participants in randomized trials could be aggregated, leaving the analysis dependent on extrapolations and observational studies.

In summary, more than 16 million Americans have ASCVD, contributing to substantial morbidity, mortality, and costs.[10] Based on the side-effect profile reported in trials of aspirin therapy,[17,75] 3% of these patients would be expected to have a total of 319,000 episodes of gastrointestinal bleeding at an aspirin dose of 325 mg/day. Even assuming a conservative 10% reduction in gastrointestinal bleeding with optimal dosing of aspirin, about 32,000 such bleeding events and 3,200 deaths from gastrointestinal hemorrhage[79] would be prevented annually in the United States alone. Accordingly, there is a clear need for an adequately powered randomized controlled trial to define the optimal dose of aspirin in order to minimize bleeding risks while preventing ischemic events.

II.E. Modifiers of Aspirin Dose

II.E.1. Aspirin, Minorities, and Other Subgroups

Early trials with aspirin were performed almost exclusively in men, leading to the empirical observation that the significant reductions in events were limited to men. Further study showed this simply was a problem of inadequate power to detect an effect in women, because very few women had been enrolled in trials. In a large prospective cohort study of 28,678 nurses taking one to six aspirins (dosage unknown) per week, the age-adjusted relative risk (RR) of a first MI was 0.68 (P=0.005) with a trend toward fewer cardiovascular deaths (RR, 0.89; P=0.56).[81] Still, without randomized trials enrolling representative numbers of men and women, it remains unknown whether aspirin has the same effect in men and women or if it shows a similar relation between dose and outcome.

In addition, little is known about how the effect of aspirin is modulated by age, race and ethnic background, the presence of diabetes or renal function. Because ASCVD is the leading cause of death in women, racial minorities, diabetic patients, and patients with CKD in the United States,[10] and because its prevalence and associated adverse events increase proportionately with age, it is critically important to perform randomized trials of aspirin use in these populations. Patients with diabetes constitute a particularly important subgroup, given the considerable evidence that they may exhibit resistance to the antiplatelet effects of aspirin.

II.E.2. Other Issues

II.E.2.1. Enteric Coating

Enteric coating of aspirin became popular as a proposed approach to reducing aspirin's gastrointestinal toxicity. While some small studies have shown promising results regarding the benefit of enteric coating, no large outcomes trials have evaluated the benefit-risk balance of enteric-coated aspirin versus aspirin without enteric coating. Additionally, pharmacokinetic/pharmacodynamic studies have raised questions about the reliability of absorption of aspirin when given in enteric-coated form.[82,83] Given these uncertainties, we will collect details of the actual dose and type (enteric-coated vs. non-

enteric-coated) of aspirin taken by trial participants at regular intervals, but the randomization to low vs. high-dose aspirin will not specify whether the patient should take enteric-coated aspirin.

III. Research Design and Methods

III.A. Study Aims 1 to 3: Aspirin Dosing

We plan to compare the effectiveness of two once-daily doses of aspirin (81 mg and 325 mg) in a secondary-prevention trial in patients with ASCVD, using a novel format that exploits EHR data that have been standardized according to a common format and primarily web-based systems of communication among enrolled patients and trial investigators with the support of health systems interested in the best care for their patients. Every aspect of the trial is designed to not only answer the research question, but also to build an infrastructure for future pragmatic trials in which a community of patients, clinicians, researchers and administrators work together to improve patient care and clinical outcomes.

III.A.1. Enrollment and Eligibility

Importantly, these criteria are intended to reflect the best judgment of clinicians in practice and to reflect the general "uncertainty principle." For patients whom aspirin is indicated to reduce recurrent events, the clinician is uncertain about the best dose of aspirin to prescribe. These enrollment criteria have been selected to achieve the most generalizable sample possible to address the main study hypothesis. Furthermore, the protocol was posted for public review and comment during July, 2015. Specific questions regarding key eligibility criteria were voted upon and the responses are described herein. Approximately 57% of the survey respondents voted to not limit the inclusion criteria to only include patients already taking aspirin at the time of randomization (with 7% expressing no opinion), 49% voted to allow patients taking ticagrelor at the time of screening to be included in the trial (with 14% expressing no opinion), and 49% voted to exclude patients with a clear indication for an oral anticoagulant even if they are not current taking an oral anti-coagulant (with 6% expressing no opinion). The final inclusion/exclusion criteria listed below were adapted based upon these survey responses, but based upon feedback from the CDRNs regarding their local Institutional Review Boards' perspective on the "minimal risk" of the informed consent process, it was decided to exclude patients treated with ticagrelor given the "black box" warning in the ticagrelor label in the United States for avoiding high-dose aspirin with concomitant ticagrelor use.

1. Known atherosclerotic cardiovascular disease (ASCVD), defined by a history of prior myocardial infarction, prior coronary angiography showing ≥75% stenosis of at least one epicardial coronary vessel, or prior coronary revascularization procedures (either PCI or CABG)
2. Age ≥ 18 years
3. No known safety concerns or side effects considered to be related to aspirin, including
    a. No history of significant allergy to aspirin such as anaphylaxis, urticaria, or significant gastrointestinal intolerances
    b. No history of significant GI bleed within the past 12 months

   c. Significant bleeding disorders that preclude the use of aspirin

4. Access to the Internet. In the event that the CDRNs are notified that a cohort of patients without internet access can be included, then patient agreement will be obtained during the consent process to provide follow-up information by telephone contact with the DCRI Call Center.
5. Not currently treated with an oral anticoagulant – either warfarin or a novel anticoagulant (dabigatran, rivaroxaban, apixaban, edoxaban) – and not planned to be treated in the future with an oral anticoagulant for existing indications such as atrial fibrillation, deep venous thrombosis, or pulmonary embolism.
6. Not currently treated with ticagrelor and not planned to be treated in the future with ticagrelor.
7. Female patients who are not pregnant or nursing an infant
8. Estimated risk of a major cardiovascular event (MACE) > 8% over next 3 years as defined by the presence of at least one or more of the following enrichment factors:
   a. Age > 65 years
   b. Serum creatinine > 1.5 mg/dL
   c. Diabetes mellitus (Type 1 or Type 2)
   d. 3-vessel coronary artery disease
   e. Cerebrovascular disease and/or peripheral arterial disease
   f. Left ventricular ejection fraction (LVEF) < 50%
   g. Current cigarette smoker

There will be no exclusions for any upper age limit, comorbid conditions, or concomitant medications other than oral anticoagulants and ticagrelor that are used at the time of randomization, or are planned to be used during the study follow-up.

Simple, inclusive eligibility criteria will make enrollment easier, and will render study results more generalizable to a broader population of patients. We will exclude pregnant or lactating women (because of concern for the fetus or child), patients taking oral anticoagulants or likely to require an oral anticoagulant during trial follow-up (because of complex drug interactions and a projected excessive risk of bleeding), and patients at relatively low risk for cardiovascular events (ie, no enrichment factor because of the large number of outcomes needed to detect a clinically meaningful difference with the available sample size).

III.A.2. Solicitation of Participation

The majority of the PCORnet's Clinical Data Research Networks (CDRNs) and 1 Patient-Powered Research Network (PPRN) have agreed to participate in this trial. After obtaining IRB approval of the trial and with agreement of the clinicians and their health systems, the EHRs at the participating health systems in the CDRNs will be queried for eligible study participants. This will be supplemented with local engagement of health systems, clinicians, and patients as well as direct recruitment in clinics and hospitals. The trial is designed not to interfere with routine clinical practice and is expected to impose a minimal burden on clinicians, clinics, health systems, and patients.

III.A.2.a. Women, Elderly, and Minority Subjects

The inclusion of women and minorities in clinical trials is desirable for scientific, ethical, and social reasons. This trial's investigators feel strongly that the inclusion of women, the elderly, and racial minorities is imperative to meet the goal of developing a therapy that is ultimately generalizable to the global ASCVD population. The increasing number of men and women ≥65 years of age with ischemic heart disease also mandates that they be represented in a dosing trial evaluating the safety and effectiveness of aspirin. Although octogenarians represent only 5% of the U.S. population, they represent 20% of all patients hospitalized for MI and 30% of all MI-related hospital deaths.[84]

PCORnet is committed to promoting equity in research and PCORI has made concerted efforts to include underrepresented populations in research. Because this trial is intended to identify an optimal aspirin dose that is applicable to a large population of ASCVD patients, our aim is to enroll a study population whose demographic profile is similar to the representation of women, elderly persons, and minorities in the overall population of individuals with ASCVD in the United States.[20,85] Estimates of the incidence of ASCVD in American women have ranged from 30%[86] to 51%[85] and vary considerably relative to age group.[87] Although the elderly (>75 years) account for <6% of the population,[58] the incidence of ASCVD among elderly patients) is proportionally higher compared with younger age groups (169 vs. 60 per 1,000 persons).[88] Based on these demographics, we will aim for 35%-40% of our enrolled patients to be women and at least 10% to be elderly (≥ 75 years). Because the incidence of ASCVD is similar among ethnic groups in the United States (~7% for each group of non-Hispanic whites, non-Hispanic blacks, and Hispanics),[85] we have selected to strive for enrollment target of 20-25% for minorities to reflect their representation in the general population (e.g., 12.1% non-Hispanic blacks, 12.3% Hispanics).[58]

The CDRNs will develop local recruitment strategies specifically designed to facilitate the inclusion of a broadly representative population of patients that incorporates women, the elderly, and racial/ethnic minorities. For example, many participating sites are regional centers located in areas with higher numbers of minorities; this will enhance minority recruitment.

III.A.2.b. Sources of Potential Bias

Because this trial emphasizes the use of the Internet and standardized EHR data, we acknowledge potential barriers to participation including older age, low socioeconomic status, and low literacy. However, Internet use will facilitate the conduct of this pragmatic trial by reducing potential limitations to enrollment, including the costs associated with frequent follow-up visits at healthcare facilities. We will encourage enrollment of patients with personal Internet access as well as patients with public Internet access only, such as those available through a library, enrolling physician's office, or workplace. Additionally, the ADAPTABLE patient portal will be accessible via tablet computers and smart phones so patients with access to the internet only through cellular service will be able to participate in this manner.

No studies have formally evaluated the association of Internet use with general health status and outcomes. Internet users may derive indirect benefits that improve their health status (e.g., disease recognition, symptom awareness, risk-factor modification), and/or they may be better educated, more

motivated in personal health maintenance, and wealthier, all of which may be associated with better health status and outcomes in secondary prevention. Although the investigators acknowledge these possibilities, we believe the results of this trial remain applicable to all patients with ASCVD for three reasons.

First, since the occurrence of cardiovascular events is multifactorial, standard risk factors (e.g., smoking, diabetes, and age) are more likely to directly influence cardiovascular event rates than Internet use. Second, aspirin use has substantial consequences, effecting a ~25% reduction in adverse cardiac events. Rather than modify the benefit of aspirin therapy, Internet use would more likely be associated with increased awareness of the general need for aspirin therapy, which also is an educational objective of the trial patient portal. Third, the aim of this trial is to define the optimal dose of aspirin in patients with ASCVD. Even if Internet use were associated with improved health, this effect would be a general reduction in adverse events across all treatment groups. Although attributing a global improvement in outcomes in this trial to Internet use may be hypothesis-generating, such a finding still would not influence the primary objective of the trial.

Another source of bias may exist with respect to aspirin therapy prior to randomizations. We expect that most patients recruited into the ADAPTABLE trial will already be treated with long-term aspirin therapy, given the nature of the inclusion criteria and the known high utilization of aspirin for the secondary prevention of coronary artery disease in the United States. However, we will add a sensitivity analysis with a 10-day landmark to the statistical analysis plan (SAP) that excludes all events occurring in the first 10 days following randomization in order to formally test for a legacy effect of the aspirin dose taken before randomization.

III.A.2.d. Patient Engagement

The ADAPTABLE trial will address several key questions mentioned in PCORI's definition of patient-centered outcomes research. This trial is designed to help individuals answer the following questions: "Given my personal characteristics, conditions, and preferences, what should I expect will happen to me?" "What are my options and what are the potential benefits and harms of those options?" "What can I do to improve the outcomes that are most important to me?"

The study seeks to make more relevant, detailed information regarding aspirin therapy as secondary prevention for ASCVD available to patients and their caregivers by answering question such as "What is the most effective dose of aspirin in secondary prevention of ASCVD?" and "What are the bleeding risks associated with taking 81mg or 325mg of aspirin?" Of the forty topics suggested for the first PCORnet study, six were selected for consideration through the PCORI Advisory Panel Prioritization process by a body consisting of patients, scientists, and other stakeholders charged with prioritizing CER topics for PCORI. ADAPTABLE was chosen to be the first PCORnet study based on its importance to patients, clinicians and scientific communities.

Patient and stakeholder engagement has been a priority to the study team since the inception of ADAPTABLE. A partnership was formed with the Health eHeart Alliance Patient-powered Research

Network to support ADAPTABLE by leading the CDRN patient representative team called the Adaptors. Additionally, two seasoned cardiology patient advocates served on the protocol design committee to assist with defining essential characteristics of the study, participants, and outcomes. These advocates also worked actively with the study team and Health eHeart to develop the patient engagement plan.

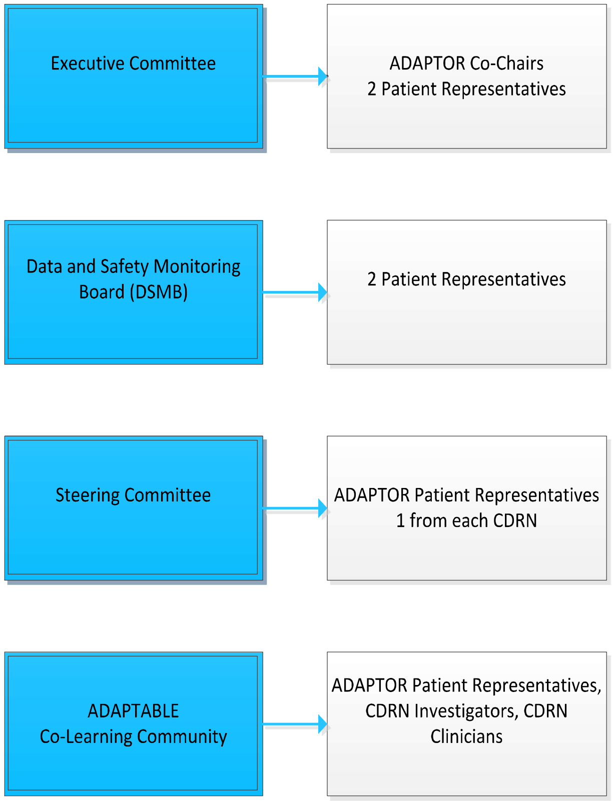

The DCRI Coordinating Center for ADAPTABLE will convene the Executive Committee, Steering Committee, and Data and Safety Monitoring Board (DSMB) – each of which will have at least 2 patient representatives, while Health eHeart will support and facilitate the Patient Group herein referred to as "Adaptors." The Adaptors group will include one patient representative from each participating CDRN. CDRNs will seek to identify patients who represent the trial's population (see **Appendix: Patient Survey**), and they may select skilled cardiology patient representatives who use aspirin if they are unable to locate a patient representative with appropriately matched characteristics and skills. Diversity and inclusiveness are highly valued and encouraged. We will ensure that the patient representatives will be recruited from the population they are serving by using the Adaptors Screening Tool (see attachment provided with this document). The screening tool will be circulated by Health eHeart and partnering CDRNs to identify potential patient representatives for the Adaptors group. The Adaptors will serve a dual role as designers and advisors; they will work in concert with the study team to help design study materials, the trial consent form, and recruitment plans through their work with Health eHeart. They will also be members of the Steering Committee where they will monitor study conduct and progress. The Co-Chairs of the Adaptors group, who will be selected by the patient representatives and confirmed by Health eHeart and the DCRI Coordinating Center to be able to carry out the associated duties, will also serve as the patient representatives on the Executive Committee to provide study oversight, promote cross-pollination of ideas and sharing of information between the Executive Committee, Steering Committee and the Adaptors Group. Also, two cardiology patient advocates (not a part of the Adaptors) will serve on the DMSB to participate in review of study data sets and adverse event monitoring and reporting.

The ADAPTABLE Co-Learning Community (ACLC) will be a discussion forum supported by Health eHeart and composed of Adaptor patient representatives, investigators, clinicians, study team members, and support staff from participating networks, and associated professional society and disease advocacy organization representatives. The forum will promote information sharing from diverse perspectives with anticipated discussion of issues related to physician-patient communications, adverse events potentially related to aspirin, common patient questions, adherence, and patient participation in

research. The forum will be co-managed by team members from Health eHeart and the DCRI Coordinating Center to ensure that study-related issues are properly addressed through appropriate channels such as committee meetings. However, a discussion forum is available to promote co-learning, sharing of information and community building in a less formal manner than structured, time-constrained committee meetings. The diagram at right shows how patients will be engaged in all operations of the trial.

III.A.3. Study Design and Procedures

III.A.3.a. General Enrollment Plan and Timeline

To meet the goal of 20,000 patients, we plan to enlist the CDRNs and patient advocates to mobilize the requisite number of clinics and health system based populations. Enrollment of at least 2,500 patients per CDRN over an approximate 24-month period is expected. Informed consent will be required from all participants The anticipated study duration is 3 years; 3 months for planning, 24 months for enrollment, a maximum of 30 months for follow-up, and 3 months for data analysis.

III.A.3.b. Cohort Identification

Local site investigators within the CDRNs will be asked to endorse the protocol. They will then be asked to give their permission for the CDRN, through its integrated health system members, to identify and contact potentially eligible patients. In the latter case, patients who meet criteria for secondary prevention after a cardiovascular event will be identified using search algorithms developed by the DCRI Coordinating Center (based on the trial inclusion criteria) and customized by the CDRN for their own EHR systems. Broad agreement from both cardiovascular specialists and primary care physicians will be sought. In this trial, we believe that most systems will agree that prior approval of the relevant clinician will be needed and useful since these patients will be at high-risk for death or a major disabling event. Although it is unlikely that a medical reason for ineligibility will be found, most of these patients are close to their clinicians, whose confidence in and support of the trial will be important for patient engagement, both in terms of participation as well as promoting adherence to the study medication and treatment of the inevitable clinical events.

III.A.3.c. Enrollment, Randomization, and Drug Allocation

Patients who are identified as candidates for the trial will be directed to the ADAPTABLE patient portal for the eConsent as well as an abbreviated eligibility confirmation prior to randomization. The ADAPTABLE patient portal will include the electronic patient consent (eConsent) that satisfies the local Institutional Review Board (IRB) and state law requirements of the participating CDRNs. CDRN personnel will be available, if needed, to give a more local perspective on protocol-related consultations.

Patients will be randomized via the patient portal in a 1:1 ratio to receive 81 mg vs. 325 mg of aspirin. Patients will be asked to obtain their randomized aspirin dose at their local pharmacies. The randomization scheme will be established before the inception of enrollment. In a trial of this size, stratified randomization has no significant advantages.

After randomization, patients will be asked to answer questions related to current aspirin use and dose, and other specific concomitant medications. They will also be asked to provide contact information such as their name, email and home address, phone numbers and contact information for a family member or friend not living with them. This data will be used to contact patients that miss multiple visits and to identify events such as hospitalizations that occur out of network.

Neither patients nor health care providers will be blinded to their treatment assignment as blinding would add substantial complexity and cost without commensurate incremental benefit. The "hard" outcomes, large sample size and equipoise in the clinical community should enable valid results to be obtained, and we have no reason to believe that investigator or clinician bias will play a role in ascertainment or classification of events.

III.A.3.c. Concomitant Therapy

Key concomitant medications will be self-reported by the participant, and over time will also be harvested from the PCORnet DataMart in CDM version 3.0 as it is deployed. Pre-randomization aspirin dose for aspirin users will be recorded. Participants will report key concomitant medications annually, either on the patient portal or during phone follow-up calls from the DCRI Call Center.

Details on the use of aspirin and important concomitant medications (P2Y12 inhibitors) and other over-the-counter medications of interest (non-steroidal anti-inflammatory agents and proton-pump inhibitors) will be collected from patients at baseline and annually during study follow-up. Details on other common secondary prevention medications prescribed (beta-blockers, ACE inhibitors/angiotensin receptor blockers, and statins) will be collected from the CDM. The large trial population and randomization will minimize potential biases and confounding that could result from differential use of concomitant medications by randomized treatment assignment. A sensitivity analysis that accounts for concomitant medications as time-dependent covariates will be done to account for post-randomization treatments.

III.A.3.d. Schedule of Assessments

	Baseline All participants	1-3 Week Follow-up Post-Randomization	Every 3 or 6 Month Follow-up	Annual Follow-up (12 & 24 month)	End of Study
			Assessments collected every three or six months based on randomization for up to 30 months	Replaces the 3/6 Month Follow -up	
Video describing trial expectations	X				
Check key eligibility review	X				
Sign e- Consent	X				
Randomization and dose assignment	X				
Collect current aspirin dose	X				
Collect demographic/ contact information	X				
Collect key concomitant (Rx and OTC) medications		X		X	X
Collect current aspirin dose/reason for DC		X	X	X	X
Collect hospitalizations			X	X	X
Collect Patient Reported Outcome (PRO)	X		Q6 months	X	X
Insurance Info/Confirmation of Contact Info		X		X	X

III.A.3.e. Data Collection and Follow-up

For all participants and during follow-up, demographics and key medical history, cardiovascular risk factors, and certain current medications will be obtained from the CDM. Patients will be randomly assigned to follow-up contact every 3 vs. 6 months via the ADAPTABLE patient portal. During follow-up contacts, data on aspirin dose and use, and hospitalizations will be collected. By embedding a randomization for follow-up every 3 months vs. every 6 months for patients via the ADAPTABLE patient portal, we will be able to investigate the best method for optimizing patient follow-up and data collection in this pragmatic trial. In the event that the CDRNs are notified that a cohort of patients without internet access can be included, then these patients will be contacted every 6 months by the DCRI call center via telephone for the follow-up contacts.

As envisioned by PCORnet, the PCORnet distributed research network (DRN) is to be a "…functional distributed research network that facilitates multi-site patient–centered research across the CDRNs, the PPRN, and other interested contributors. The distributed network enables the conduct of observational research and clinical trials while allowing each participating organization to maintain physical and operational control over its data."[91]

In PCORnet's distributed data environment, code is developed centrally and distributed to each partner to execute against data that are stored in a common format. Code ("queries") are distributed and results are returned via PopMedNet, a networking software application that manages the creation, operation, and governance of distributed health data networks.

The PCORnet CDM is the foundation of the PCORnet DRN. The PCORnet CDM is being implemented in phases to allow for the incorporation of new data domains and fields based on PCORnet needs, lessons learned from use, and data availability.

The CDM contains some of the 18 elements that define PHI under HIPAA, including encounter dates and date of birth. The necessary "cross-walks" between the arbitrary identifiers included in the CDM and their originating data are not specified in the scope of the CDM, but are expected to be maintained by each CDRN.

- PATID is a pseudoidentifier with a consistent crosswalk to the true identifier retained by the source site. For analytical data sets requiring patient-level data, only the pseudoidentifier is used to link across all information belonging to a patient.
- Locally maintained "mapping tables" are tables necessary to implement so that each CDRN/partner has the ability to map arbitrary identifiers back to the originating data and patient.

The PCORnet CDM Version 3.0 includes ten tables that represent specific data domains that are available in EHRs and directly from patients. **Table 4** describes data categories that are applicable to the ADAPTABLE case report form (CRF):

Table 4. Data Categories Applicable to the ADAPTABLE CRF		
	**Description**	**Applicability for ADAPTABLE Data Collection**
Demographic	Contains 1 record per patient.	Birth date, sex, Hispanic ethnicity (y/n) & race captured.
Encounter	Contains 1 record for each time a patient sees a provider in ambulatory setting or is hospitalized; multiple encounters/day possible if they occur with different providers or in different care settings.	Encounter type will be used to identify hospitalizations during follow-up period. Deaths occurring during a hospital stay will be captured in the discharge status variable.
Diagnosis	Contains all uniquely recorded diagnoses for all encounters. Each diagnosis is associated with a specific patient & encounter.	Diagnosis codes and associated encounter dates will be used to establish medical history prior to randomization.
Procedure	Contains all uniquely recorded procedures for all encounters. Each procedure is associated with a specific patient & encounter.	Procedure codes and associated encounter dates will be used to establish cath, PCI, and CABG prior to randomization.
Vital	Contains one record per result/entry. Multiple measurements per encounter are recorded as separate measures.	Height and weight are captured; tobacco status has been added to v2.0.
Lab Result	Contains 1 record per result/entry.	Creatinine, hemoglobin, and LDL-C will be included in v2.0.

In essence, except for aspirin dose and use of over the counter NSAIDS, H2 Blockers and PPIs, all of the essential data for the trial are included in CDM 3.0. A unique attribute of this trial is the continuous improvement expected in the extent and complexity of the available data as the trial goes on.

III.A.3.d.i. Minimizing Cross-Overs

Because this is an open-label trial in which participants and their healthcare providers will know their randomized dose of aspirin, the CDRN will be asked to engage regularly with the healthcare providers in their network regarding the rationale for the trial and the importance of compliance with the randomized treatment assignment to minimize patient cross-over. Health care providers will be asked not to change a patient's aspirin dose during the trial unless it is absolutely necessary to ensure patient safety. On the ADAPTABLE patient portal, patients will be reminded of the importance of adhering to the aspirin dose they were randomly assigned to unless their physician changes their dose for a major safety concern. Patients who cross-over will be analyzed as they were originally randomized according to the intention-to-treat principle.

III.A.3.d.ii. Drug Discontinuation – Monitoring and Recommendations

Aspirin use will be collected via the responses to the patient questions during follow-up. During each follow-up contact, compliance with the randomized aspirin dose will be assessed. The occurrence of and reasons for discontinuing aspirin or changing the dose of aspirin will be ascertained. In the event of aspirin intolerance or bleeding, the decision to continue the randomized aspirin dose, convert to a lower or higher aspirin dose, or to discontinue aspirin therapy will be left to the treating physician's discretion. Patients who discontinue aspirin therapy altogether will remain in the trial for clinical outcomes follow-up according to the intention-to-treat principle.

If a female participant becomes pregnant or plans to attempt to become pregnant during study follow-up, then aspirin should be permanently discontinued. Furthermore, if a participant requires an oral anti-coagulant during study follow-up, the treating physician should carefully consider whether to continue

aspirin based upon the estimated bleeding risks of aspirin used together with an oral anti-coagulant based on the clinical context. Finally, participants who develop an intracranial hemorrhage or a life-threatening bleeding event during study follow-up should be considered for permanent discontinuation of aspirin, at the discretion of their treating physician.

III.A.3.d.iii. Delinquent or Missing Follow-up

Data from patients who fail to respond to questionnaires via the ADAPTABLE patient portal or via telephone contact with the DCRI Call Center after at least two separate contact time points will be collected using all available approaches including but not limited to the DCRI call center, patient finder companies, the Internet, EHR data stored in the CDM format in the PCORnet DataMart, the Social Security Death Index, and other additional search methods. Additionally, if these methods fail, the site will be contacted to determine whether they have been in contact with the patient.

III.A.3.e. Developing and Refining the PCORnet Infrastructure

Based on previous experience with clinical trials and patient follow-up (see **Section III.A.3.d**), there is broad familiarity with the potential limitations of EHR data and patient-reported clinical outcomes. As a result, PCORnet is in a position to identify and resolve inaccuracies to improve the validity of such data. Because this trial will investigate a clinically relevant issue in a new format, the study will also examine the potential for conducting trials through health systems with secondary data use and Internet-based follow-up with both participants and clinician-investigators. Because this trial will rely in large part on patient-reported data gathered via the Internet, it will enable us to show the feasibility of participant enrollment and reporting via the Internet. As previously stated, Aim 3 of this project will focus upon using defined metrics to evaluate the PCORnet infrastructure that will be developed across PCORnet studies.

III.A.3.f. ADAPTABLE Patient Portal

Patient data will be captured via the ADAPTABLE patient portal that will be designed and maintained by Mytrus. Screens will be user-friendly and will contain questions that are easy to understand and answer. Content for the Patient Portal will be in English and Spanish to facilitate bi-lingual interactions. Based on previous studies of Internet usability for people >65 years,[92] the study patient portal will be designed to accommodate issues such as impaired vision (e.g., font size and "readability"), memory (e.g., focused error messages and hypertext links), and motor control and precision of movement (e.g., "clickability"). Screens, checkboxes or pull-down menus will be used when possible for data entry, to minimize the need to enter free text.

Queries will be programmed directly into the study patient portal and will prompt patients for missing or discrepant data. For example, if a patient skips a question, a program warning will occur that directs the patient to complete all questions before continuing. Similarly, program warnings will alert patients if out of range data have been detected. This method of on-the-spot querying will ensure cleaner data.

The patient portal will contain a general information section that allows access by study participants and general and medical lay communities. There will also be restricted sections on the patient portal that are

accessible only to the trial's investigators. The restricted section for investigators will contain more specific information about their local participation. Visibility of the link to the restricted section will be determined by the access rights.

Below is a partial list of items that could be shared:

- *Training materials,* to include resources for training in ethics of human research, recordings of investigator meetings/conference calls, and other appropriate training materials.
- *Educational links,* to provide additional education materials for patients (*e.g.*, the American Heart Association website) and providers (*e.g.*, theheart.org; the American College of Cardiology website).
- A *protocol* section, to include the current version of the protocol. Eligibility criteria will be listed, as will instructions for completing follow-up questionnaires through the patient portal.
- Regular communications, to include enrollment data, frequently asked questions (FAQs), and other trial information.
- *Enrollment information* to be posted on the general area of the site.

III.A.4. Data Security and Back-up Procedures

III.A.4.a. Mytrus Data Security for the Patient Portal

The system is deployed using a LAMP (Linux, Apache, MySQL, PHP) technology stack running on a virtual machine (VM) in a private cloud. By using a virtualized environment, additional resources (disk space, processing power) can be added to the VM as necessary to support increased load. Data centers used to host the system hold current SSAE16 SOC certification and have Tier 1 internet access from multiple major Internet Service Providers (ISPs) for redundancy and 100% network uptime.

Access to the system's web-based user interface (UI) is provided using HTTPS (web protocol that is secured using 128-bit [or stronger] encryption). Each user must have a unique username and secret password to authenticate into the system's UI using a browser. All passwords, whether used to access the web UI or to access a server directly via the backend, are required to meet minimum requirements for strength, complexity, and aging. Subsequent responses by the server to requests sent from the user's browser are restricted and processed according to the user's assigned role (i.e., using role-based access control that is strictly enforced by the application logic).

Direct access to the VM which hosts the system is only possible via a virtual private network (VPN) and then only by senior technology staff who are documented on an access control list that is reviewed for accuracy at least quarterly. Direct access to the MySQL database is also only possible by using the VPN, and the MySQL database server is configured to disallow network connections and only to accept connections that originate from the same server ("localhost"). That is, it is not possible for a MySQL client to connect to the MySQL database over the network. The client must be on the same VM as the database server.

The firewalls that implement the VPN only allow connections to ports that are required for the system to function (i.e., to port 443 for HTTPS connections). All other attempted connections are rejected. This configuration ensures that an end-user can only access data stored in the database by first authenticating with and sending requests through the web server, which processes each request and returns results that are determined by the user's role.

The system leverages industry-standard techniques to maintain high-levels of data security and integrity. User credentials are stored as salted hashes, and the study team does not have access to or the ability to recreate user passwords. Direct access to the study database is tightly controlled. Moreover, all changes to the database (create, modify, delete) are tracked using a 21 CFR § 11-compliant audit trail.

Security is further ensured by the use of an Alert Logic Intrusion Detection System (IDS) and an Alert Logic Log Manager, both of which monitor all servers on the network. The Alert Logic IDS inspects all inbound and outbound network traffic (i.e., data packets) that passes through the network (both from the Internet and within the private network). Moreover, the IDS generates regular reports of possible exploits, thereby ensuring that outdated server software is promptly detected and upgraded to more secure patch levels.

To ensure high-availability, the system is configured with a primary server and an identical failover ("disaster recovery") server that is located at a geographically remote data center. If the primary site becomes unavailable, the failover site can be activated well below the 60-minute Recovery Time Objective. The primary and failover database servers are also configured for replication, thereby ensuring that any transaction on the primary database also occurs on the failover database seconds later. As a further safeguard against data loss, the failover server exports the database to file every thirty minutes, and retains exports for 72 hours. In addition to the database exports, each server itself is backed up in-full daily and weekly.

To ensure that the protected health information (PHI) of research participants remains confidential, any personally identifying information (PII) that is stored in the database is encrypted using 128-bit AES encryption algorithm. Moreover, the technology staff who maintain the systems are required to use personal computers that use password protected access, screen locking, hard drive encryption, VPNs, and virus scanning utilities to ensure that their access to study's servers cannot be compromised or misappropriated by unauthorized parties.

The data centers are monitored by certified network technicians and security personnel 24/7/365, and provide physical and logical security through a combination of keycard protocols, biometric scanning protocols, and around-the-clock interior and exterior surveillance. Access to the facilities is limited to authorized data center personnel—no one can enter the production area without prior clearance and appropriate escort. Every data center employee undergoes thorough background security checks. Conditioned power provides 100% availability through UPS (Uninterruptible Power Supply) for all servers; N+1 redundant UPS power subsystem, with instantaneous failover if the primary UPS fails. If an

extended utility power outage occurs, routinely tested, onsite diesel generators can run indefinitely. The facilities also offer a precision environment. Using an N+1 redundant HVAC (Heating Ventilation Air Conditioning) system ensures a duplicate system immediately comes online in the event of an HVAC system failure; Every 90 seconds, all air is circulated and filtered to remove dust and contaminants; the facilities also use advanced fire suppression systems appropriate for a data hosting environment. The facilities network topology and configuration was co-developed with Cisco and guards against single points of failure at the shared network level. Cisco and Arbor Networks work with the data centers to continually improve monitoring and security.

III.A.4.b. PopMedNet Data Security

Data will be transferred from the sites to the DCRI using PopMedNet.[97] PopMedNet consists of two layers: a security layer where access controls and permissions are established and an exchange layer through which questions and responses are passed. Each of the current implementations of PopMedNet™ is hosted in a Federal Information Security Management Act (FISMA)-compliant private cloud tier III data center. All communications from the portal are encrypted. Selected current and in-process security features include:

- Strong passwords expire every 6 months and may not be reused
- Automatic logoff after inactivity
- Automated query results deletion
- Audit of all system activity
- DataMart administrators notified of new users
- Encrypted password storage
- Use of cryptographically secure random values for session IDs
- Secure distribution of DataMart Client software

III.A.4.c. Duke Clinical Research Institute Data Security

One major risk in patient-centered research is introduced by the necessity to use patient identifiers (e.g., a patient's name and phone number is needed in order to call them for follow-up) in data systems outside the enrolling site without compromising the confidentiality and security of data. The principles of minimum-necessary and compartmentalization of identifiable data will be adopted. Primary identifiers will be retained only within the data systems where required to conduct the study; these will be omitted as data travel downstream and only study IDs will be in the analysis datasets. Further de-identification and anonymization methods will be used when making data available for secondary analysis.

The DCRI, as part of the Duke University Health System HIPAA-covered entity, routinely operates at the intersection of handling PHI and the requirements of large-scale, multicenter research programs and data-sharing initiatives. The primary computing platforms for enterprise systems are Sun Servers running the Solaris 8 operating system, a Unix-based operating system. All Oracle databases run on the Sun Servers, which are additionally attached to the HP Storage Works Storage Area Network (SAN). Client-server applications require network authentication. In addition, client-server applications may have their own internal security system and/or Relational Database Management System (RDBMS)

security. The DCRI's core back-end tools used on this project will be SAS Analytics (SAS Institute Inc.) for data integration and data analysis. As a HIPAA-covered entity and experienced research organization, we employ a variety of technical and SOP-driven approaches to ensure the security and confidentiality of all data.

Data will be transferred from the sites to the DCRI using PopMedNet. The DCRI will be responsible for maintaining the confidentiality and security of transferred data. The control of access to databases will be managed centrally by the DCRI through user passwords linked to appropriate access privileges. This protects data from unauthorized view and modifications as well as inadvertent loss or damage. The DCRI, and our technology partner Lincoln Peak Partners, have an extensive data security infrastructure. Database servers are secured by a firewall as well as through controlled physical access. Oracle has many security protection features that ensure that each person accessing the database has the proper authority to perform the functions he or she requests of the data management system. Within the secondary SAS databases, UNIX group access control will be used for maintaining similar security. The Sun workstation log-in will be secured by extensive user password facilities under UNIX.

DCRI is part of the Duke HIPAA covered entity and has experience working with PHI in the research context; it is extremely prudent in keeping patient data secure and confidential.

III.A.5. Endpoints and Adverse Events

III.A.5.a. Primary Endpoint

The primary effectiveness analysis will be performed on the entire randomized (intention-to-treat) population. The primary endpoint of this study is the composite rate of all-cause mortality, hospitalization for nonfatal MI, or hospitalization for nonfatal stroke. Traditional reporting of potential endpoints by study sites with independent adjudication by a Clinical Events Committee will not be done in this trial given the pragmatic nature of the ADAPTABLE study and the relatively low-cost of the trial budget. We are planning to implement an endpoint validation plan to ensure the accuracy of endpoint classification compared with clinical endpoint adjudication processes used in traditional clinical trials. Once approved, the description of the validation plan will be provided in a separate document. Validated coding algorithms will be applied to a variety of EHR data sources to comprehensively ascertain potential endpoints related to hospitalizations for the non-fatal component primary endpoints (MI and stroke) and the secondary endpoints. A recently published study demonstrated that events ascertainment and classification using coding algorithms with administrative claims data yielded similar results compared with the standard adjudication of events done in traditional clinical trials.[98] The informed consent form will cover access to the data needed to support the validation plan.

III.A.5.b. Secondary Endpoints

Secondary endpoints include the components of the primary endpoint, coronary revascularization procedures (PCI and CABG), and quality of life and functional status.

III.A.5.c. Safety

The major safety endpoint is hospitalization for major bleeding with an associated blood product transfusion.

III.A.5.d. Identifying Endpoints

Endpoints will be ascertained by applying algorithms designed to ensure comprehensive surveillance for all potential endpoints.

- Routine queries will be applied to the PCORnet CDM to capture and classify endpoints using validated coding algorithms. Because the EHR and (by extension) the PCORnet CDM is the source of truth for the trial, no patient confirmation or other additional confirmation will be required.
- Hospitalization reported by patients captured and classified by the CDM will be evaluated in the following manner:
  - Queries of the PCORnet CDM will be used to classify in-network hospitalizations (nonfatal MI, nonfatal stroke, or major bleeding).
  - It may be possible for Out-of-network hospitalizations not captured in the CDM to be investigated by executing queries against the following:
    1. Near real-time Medicare fee-for-service claims that are updated quarterly
    2. Data held by large national health plans participating in the FDA's Mini-Sentinel initiative
    3. In the event that it is not possible to identify the patient reported event in either of the above 2 datasets, then hospitalization records will be obtained by the DCRI Call Center and used to classify the patient reported endpoints
- Death events are often not well represented in the EHR, especially out-of-hospital deaths. When participants do not respond to regular attempts at contact and no death has been reported in the PCORnet CDM, patient finder services will be utilized to find the patient. Additionally a series of cross-checks will be done to comprehensively capture all deaths that occur during trial follow-up. These cross checks for death ascertainment could be done via the Medicare Beneficiary Summary File (which includes death dates provided by the Social Security Administration) and the National Death Index.

III.A.5.d.i. Death

This endpoint includes death from any cause (all-cause mortality).

III.A.5.d.ii. Hospitalization for Nonfatal MI

The endpoint of hospitalization for nonfatal MI will be ascertained using ICD-9-CM diagnosis codes 410.x0-410.x1 in the principal or primary position. The algorithm was developed for use in FDA's Mini-Sentinel program[99] and has been shown to have a positive predictive value (PPV) of 86%. If these diagnosis codes are not found for a particular patient, then self-reported data regarding occurrence of nonfatal MI will be used.

III.A.5.d.iii. Hospitalization for Stroke

The endpoint of hospitalization for nonfatal ischemic stroke will be ascertained using ICD-9-CM diagnosis codes 430.x, 431.x, 433.x1, 434.x1, 435.x, 436, and 362.3 (PPV=90%).[100] The endpoint of nonfatal hemorrhagic stroke will be ascertained using ICD-9 diagnosis codes 431 and 432 (PPV=91%).[101] Both ischemic and hemorrhagic stroke hospitalizations will be incorporated into a combined endpoint of all stroke events. If these are not found for a particular patient, then self-reported regarding occurrence of nonfatal stroke data will be used.

III.A.5.d.iv. Coronary Revascularization

Coronary revascularization includes all coronary revascularization procedures (PCI/CABG) performed during the study. These will be identified using ICD-9-CM procedure codes (00.66, 36.06, 36.07, 00.40-00.48, 36.10-36.19) and CPT procedure codes (92920, 92921, 92924, 92925, 92928, 92929, 92933, 92934, 92937, 92938, 92941, 92943, 92944).

III.A.5.d.v. Hospitalization for Major Bleeding

Major bleeding at any location will be ascertained using previously validated ICD-9-CM diagnosis codes for a) intracranial bleeding, b) gastrointestinal bleeding, c) bleeding at another location or physician service code for GI hemorrhage (CPT code 43255 or ICD-9 procedure code 44.4x), together with a CPT code 36430 for any blood product transfusion during the same hospitalization.[102]

III.A.5.d.vi. ICD-10 Coding Algorithms

Given the recent implementation of ICD-10 in routine clinical practice in the United States, the aforementioned coding algorithms will be updated and adapted to ICD-10 specifications.

III.A.5.d.vii. Quality of Life and Functional Status

In addition to the effectiveness and safety endpoints listed above, we will collect data on quality of life and functional status as shown in **Table 5**:

Table 5. PCORI Patient-Reported Outcomes Common Measures – Core and Recommended (LOINC Panel 75418-4)

Domain	Item text	Answer list
General Health	In general, would you say your health is	5=Excellent; 4=Very good; 3=Good; 2=Fair; 1=Poor
Quality of life	In general, would you say your quality of life is	5=Excellent; 4=Very good; 3=Good; 2=Fair; 1=Poor
Physical Function	Are you able to run errands and shop?	5=Without any difficulty; 4=With a little difficulty; 3=With some difficulty; 2=With much difficulty; 1=Unable to do
Depression	In the past 7 days…I felt depressed	1=Never; 2=Rarely; 3=Sometimes; 4=Often; 5=Always
Fatigue	During the past 7 days…I feel fatigued	1=Not at all; 2=A little bit; 3=Somewhat; 4=Quite a bit; 5=Very much
Sleep Disturbance	In the past 7 days…I had a problem with my sleep	1=Not at all; 2=A little bit; 3=Somewhat; 4=Quite a bit; 5=Very much
Social Roles & Activities	I have trouble doing all of my regular leisure activities with others	5=Never; 4=Rarely; 3=Sometimes; 2=Usually; 1=Always

III.A.5.e. Events Collection

(Refer to section III.A.5.d. Identifying Endpoints.)

These data will be analyzed, and the trial statistician will provide them to the DSMB regularly. The DSMB will review the data every 6 months and recommend any necessary changes to the conduct of the trial. Given that aspirin has been used for more than a century and most serious adverse events have been reported in conjunction with its use, the rate of unsuspected serious adverse events that the sites will/can submit through MedWatch to the FDA is expected to be very low.

III.A.6. Summary of Statistical Methods

III.A.6.a. Baseline Demographic, Clinical, Functional, and Procedural Characteristics

Descriptive summaries of baseline demographic and clinical variables will be generated for each randomized treatment arm of the study. Continuous baseline variables will be presented as medians with 25th and 75th percentiles, and discrete variables will be summarized using frequencies and percentages.

III.A.6.b. Populations for Analysis

The Intent-To-Treat (ITT) Population will consist of all patients randomized to a treatment group in the study regardless of their compliance with the study medication. For all analyzed using the ITT population, subjects will be analyzed as randomized.

The per-protocol population is a subset of the ITT population excluding subjects who complied with the randomized treatment for less than 50% of the follow-up or had major protocol deviations expected to affect the primary effectiveness or safety endpoint.

III.A.6.c. Primary Effectiveness Comparison

The primary endpoint of this study will be survival free from the first event of a composite of all-cause death, hospitalization for nonfatal myocardial infarction, or hospitalization for nonfatal stroke. Specifications for the identification of these endpoints are provided in **Section III.A.5.** The primary effectiveness analysis will be performed by the ITT principle based on randomized treatment assignment. Event-free survival rates will be compared using Cox proportional-hazards models.[103] In the absence of any other covariates, this is the same as the log-rank test.[104] The test will be two-tailed and will be performed at an overall α of 0.05.

III.A.6.d. Primary Safety Comparison

The primary safety endpoint of this study will be the first occurrence of hospitalization for major bleeding as defined in **Section III.A.5.** The primary safety analysis will be performed by the ITT principle based on randomized treatment assignment. Event-free survival rates will be compared using Cox proportional-hazards models, equivalent to the log-rank test. The test will be two-tailed and will be performed at an overall α of 0.05.

III.A.6.e. Power

The following calculations were performed using PASS software.[105] For the primary effectiveness endpoint, power calculations were based on an estimated primary event rate of 5% per year (in the higher dose arm), annualized rate of loss to follow-up of 5%, two-sided significance level α of 0.05, 10,000 patients in each treatment arm, enrollment of 24 months and a maximum follow-up period of 30 months. The power of the chosen testing strategy to detect a statistically significant difference under these assumptions is 85% if the relative risk reduction is 15%, corresponding to a total of 1308 primary effectiveness events. The power levels for other combinations of event rates and relative risk reductions, keeping all other assumptions the same, are presented in **Table 6**.

Table 6

Annualized event rate in higher-dose arm	Relative risk reduction	No. of events	Power
4%	20%	1022	95%
	15%	1050	76%
	10%	1078	42%
4.5%	20%	1148	97%
	15%	1179	81%
	10%	1211	46%
5%	20%	1273	98%
	**15%**	**1308**	**85%**
	10%	1343	51%
5.5%	20%	1398	99%
	15%	1437	88%
	10%	1475	55%

For the primary safety endpoint, power calculations were based on an estimated primary event rate of 2.5% per year (in the higher dose arm), annualized rate of loss to follow-up of 5%, two-sided significance level α of 0.05, 10,000 patients in each treatment arm, enrollment of 24 months and a maximum follow-up period of 30 months. The power of the chosen testing strategy to detect a statistically significant difference under these assumptions is 81% if the relative risk reduction is 20%, corresponding to a total of 642 primary safety events.

The power levels for other combinations of event rates and relative risk reductions, keeping all other assumptions the same, are presented in **Table 7**.

Table 7

Annualized event rate in higher-dose arm	Relative risk reduction	No. of events	Power
2%	25%	500	90%
	20%	514	72%
	15%	529	47%
2.5%	25%	624	95%
	**20%**	**642**	**81%**
	15%	660	56%
3%	25%	748	98%
	20%	769	88%
	15%	790	64%

III.A.6.f. Secondary Endpoints

Secondary endpoints include the components of the primary endpoint, coronary revascularization procedures, quality of life and functional status (**Section III.A.5.a**). Time-to-event outcomes will be analyzed using the same approach as the one used for the primary endpoint. Variables collected on numerical scales will be analyzed as continuous variables. Linear repeated measures mixed model will be employed to compare the two treatments on the changes from baseline.

III.A.6.g. Prior Treatment Effect

We expect that most patients recruited into the ADAPTABLE trial will already be treated with pre-randomization aspirin therapy, given the nature of the inclusion criteria and the known high utilization of aspirin for the secondary prevention of coronary artery disease in the United States. Therefore, a sensitivity analysis with a 10-day landmark will be performed that excludes all events occurring in the first 10 days following randomization in order to formally test for a legacy effect of aspirin.

III.A.6.h. Handling of Missing Data

Concerted effort will be made to eliminate or minimize the occurrence of missing data. Participants will grant access to their electronic medical records at enrollment and during study follow-up as well as to have their information searched in national databases. If, despite these efforts, missing data occur, we will employ the following statistical techniques to address them.

First, reasons for missing data will be collected and described. All patients will be accounted for in all analyses and presentations. For the primary analysis based on event-free survival, subjects discontinuing the study prematurely will be censored at the time of discontinuation. However, this approach might lead to biased results if discontinuation does not occur at random. Thus, two “sensitivity analyses” will be undertaken:

1. **Inverse probability weighting.** In this approach, the contribution of each subject to the risk set calculated at time *t* will be inversely weighted by the estimated probability of remaining uncensored up to time *t*. This probability will be estimated using a Cox proportional hazards model fitted to time to censoring with variables potentially prognostic of both, failure and censoring, with baseline and time-dependent (such as most frequent major protocol deviations, certain AEs etc.), entered as covariates. In order to reduce the potentially high variability of the resulting treatment effect estimators due to sampling variability in weights, the weights will be “stabilized” by multiplication of probabilities of remaining uncensored up to time *t* estimates using baseline covariates only.
2. **Pattern-mixture approach.** Following Little et al.[106,107] we will assume that for participants who drop out, the hazard of an outcome deviates from that of participants who do not drop out by an offset, denoted by *d1* for the higher dose and by *d0* for the lower dose. We will then explore the effect of this deviation on the findings for various choices of the offsets in the two study groups. If the treatment effect is qualitatively maintained for the range of offsets that are considered to be clinically plausible, then the findings will be considered to be robust.

In other analyses, missing data will be handled using multiple imputations. Ten imputed data sets will be generated with imputation methods based on the regression or Monte Carlo framework. Final results will be based on averages from the 10 imputed data sets with appropriate estimator employed of the variance.[107]

III.A.6.i. Subgroup Analyses (Heterogeneity of Treatment Effect)

Subgroup analyses for the primary effectiveness and safety endpoints will be performed on the ITT population in order to explore whether the treatment effect is consistent across subgroups. Subgroup analyses to evaluate variation in treatment effect will be performed on the basis of tests for interaction using the Cox proportional hazards model with terms for treatment group, the subgroup variable and treatment by subgroup variable interaction. Additionally, treatment effects within each categorical subgroup will be examined separately using Cox proportional hazards models. Event rates by treatment and HRs with 95% confidence intervals will be reported for each subgroup. Forest plots will be generated displaying the estimated hazard ratios and 95% confidence intervals for each subgroup will be presented. For subgroups defined using continuous variables, the analysis based on the continuous form will be considered primary but for display purposes these variables can also be categorized.

The following subgroups determined at baseline will be examined:

- Age ≥ 65 years
- Race categories (White, Black, and Asian; Hispanic ethnicity)
- Diabetes mellitus
- Chronic kidney disease (serum creatinine > 1.5 mg/dL)
- Current P2Y12 inhibitor use
- Female sex

Although the importance of understanding the effects of aspirin in these subgroups is critical, we recognize that despite enrolling over 20,000 participants, our power to detect statistically significant and clinically meaningful interactions will be limited. However, this effort represents the largest such undertaking and is of vital importance. Heterogeneity of treatment effect will be established based on the interaction test specified above. Testing for differences between treatment arms within subgroups will be considered exploratory and no claims of heterogeneity will be made based on tests within subgroups. By their nature, these tests have low power unless the effect sizes are large, as illustrated in **Table 8.**

Table 8

Subgroup size as % of total	Relative risk reduction	Power
10%	25%	37%
	20%	25%
	15%	16%
30%	25%	81%
	20%	60%
	15%	38%
50%	25%	95%
	20%	82%
	15%	57%
70%	25%	99%
	20%	93%
	15%	71%
90%	25%	99%
	20%	97%
	15%	81%

Patients without internet access will not be analyzed as a pre-specified sub-group, but sensitivity analyses for the main trial endpoints will be incorporated into the statistical analysis plan to analyze the populations with vs. without Internet access to assess the consistency of the treatment results and potential treatment interactions, given expected demographic and socioeconomic differences between those with vs. without internet access.

III.A.6.j. Interim Blinded Trial Monitoring

During the conduct of the trial, we will employ state of the art statistical monitoring techniques. These will be conducted in a blinded fashion. We will observe study discontinuation patterns as well as reasons for the missing data. We will monitor the event rate as it accumulates and develop event rate projections which will help us determine if and when we are likely to achieve sufficient power. We will

also review the baseline characteristics of enrolled participants to make sure we are enrolling the population defined in the protocol. We will also monitor the primary safety events as they accumulate.

III.A.7. Study Coordination and Monitoring

III.A.7.a. Site Management and Quality Assurance

This trial will be monitored using quality-by-design principles[108] and will not require on-site monitoring.

III.A.7.a.i. ADAPTABLE Quality-by-Design

Final success of any clinical trial in answering the question of interest depends in large part on its design. The term “quality by design”[108] refers to steps taken at the trial design stage to foresee and limit problems that might occur during the trial conduct. Adherence to the following five “guiding principles” greatly increases the chances of final success:

1. Have we enrolled the right participants according to the protocol with adequate consent (Right Patient)?
2. Did participants receive the assigned treatment and did they stay on the treatment (Right Treatment)?
3. Was there complete ascertainment of primary and secondary outcome data (Right Outcomes)?
4. Was there complete ascertainment of primary and secondary safety data (Right Outcomes)?
5. Were there any major Good Clinical Practice (GCP)-related issues?

This trial was designed to maximize the likelihood that the five principles noted above will be followed. In particular, patient enrollment and consent is facilitated by the carefully selected and operationalized CDRNs that are the cornerstone of the PCORnet initiative. Patient selection facilitated by electronic health records (EHRs) should lead to efficient identification and enrollment of patients who satisfy study eligibility and enrichment criteria. These criteria have been chosen in a manner that corresponds to what is routinely available in EHRs, reducing the risk of any ambiguity and enrollment of patients who do not meet the protocol-specified criteria.

Although it is not possible to guarantee that all study participants will remain on the trial until its end and fully adhere to the study drug, the existence of the ADAPTABLE patient portal and potential follow-up from the DCRI Call Center are expected to keep participants engaged and help them maintain their assigned treatment by regularly asking them about the treatment they take. At the same time, because this trial is intended to describe what happens in a “real-world” population (intent-to-treat principle), variability in treatment adherence may actually contribute to better generalizability of study results.

Similarly to the eligibility criteria, primary efficacy and safety endpoints have been defined in a way that is well synchronized with the endpoint collection tools that will be employed. The focus on “hard” endpoints (that is, endpoints that can be unambiguously determined), cause-specific hospitalizations for MI, stroke, and bleeding, greatly reduces the potential of recall bias by study participants during self-report. At the same time, it facilitates improved ascertainment through the corresponding EHRs. Accordingly, the case report forms are short and questions are phrased in a manner that does not overwhelm study participants. Access to EHRs and consent to search death records should also reduce the amount of missing primary endpoint data. Minimal levels of intervention and a focus on observing

rather than influencing the study participants greatly increases the likelihood that Good Clinical Practices will be followed.

III.A.7.b. Study Network

The Study Network is composed of most of the CDRNs and 1 PPRN within PCORnet. Each of these CDRNs have developed a network specific plan to engage clinicians, identify and enroll participants and be available as patients are followed. Each CDRN will strive to be activated to begin enrollment during the first year of the study. The Coordinating Center will perform specific centralized functions; this will be advantageous to meet study budget limitations.

III.8.b.i. ADAPTABLE Leadership Structure

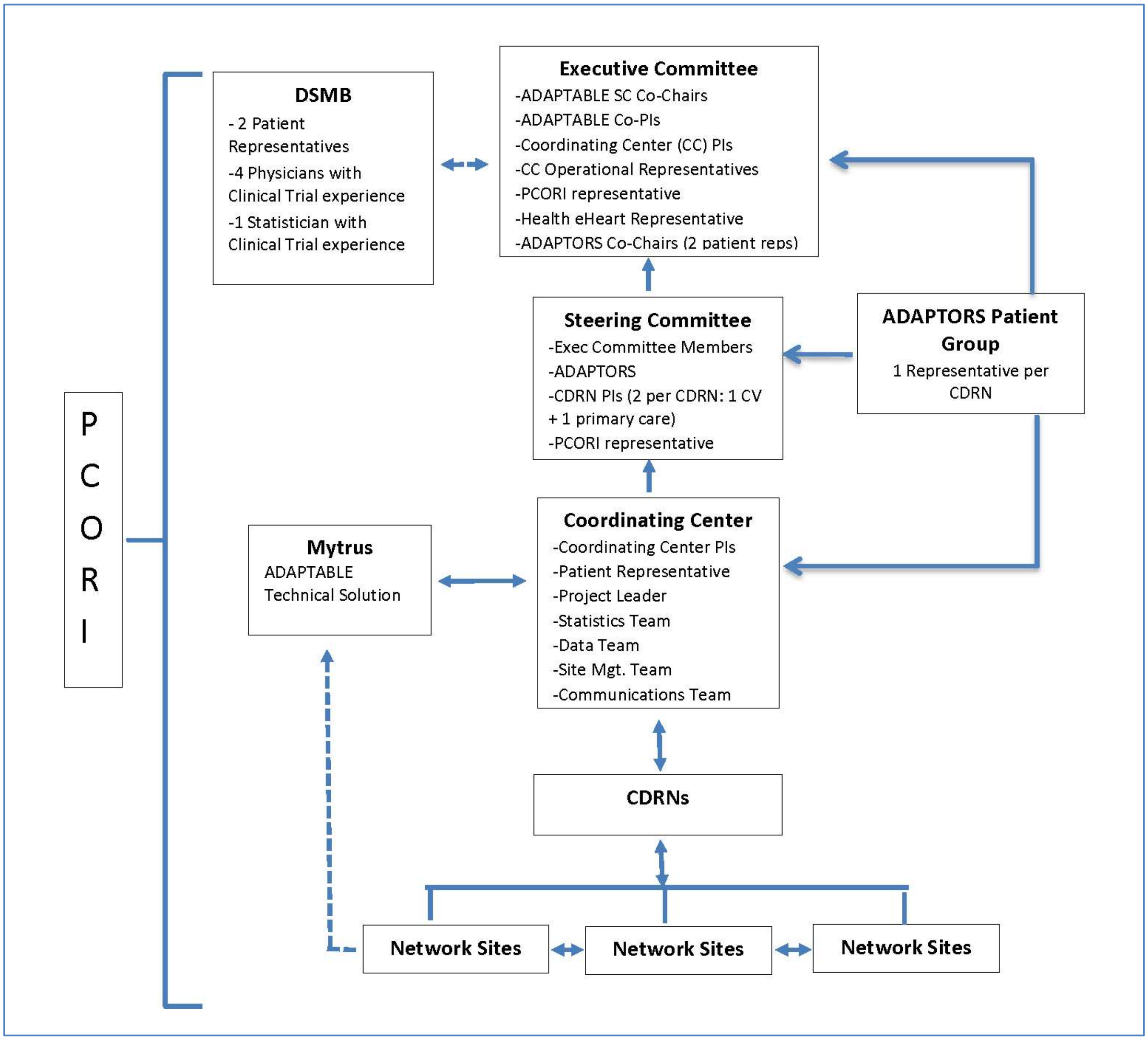

III.A.7.c. Executive Committee

The ADAPTABLE Executive Committee will provide oversight and guidance for overall trial activities. It will also serve as a communication nexus to promote sharing of information and cross-pollination of ideas among the Executive Committee, Steering Committee, study co-PIs, Coordinating Center PIs and staff, PCORI representatives, and the Co-Chairs of the Patient Representatives/Adaptors group, all of whom will have representation on the Committee. The decision-making structure and process for the Executive Committee will be described in a separate Executive Committee charter.

III.A.7.d. Steering Committee

The trial's Steering Committee will be responsible for the protocol and the scientific conduct of the study. The Committee will include patient and physician representatives from each participating CDRN and PPRN, along with selected network members with expertise in clinical research in antiplatelet therapies, cardiovascular disease, and analytical methods. Additionally, representatives from the American College of Cardiology (ACC), the American Heart Association (AHA), and PCORI will be on the Steering Committee. The Steering Committee will make decisions about any changes to the protocol and will review all proposed substudies. The decision-making structure and process for the Steering Committee will be described in a separate Steering Committee charter.

III.A.7.e. Data and Safety Monitoring Board (DSMB)

The DSMB will consist of independent members who are not participating investigators in this trial. The committee will include a Chair who is a cardiovascular specialist, a statistician, 2 patient representatives, and 3 cardiovascular specialists. The DSMB will review data regularly and recommend necessary changes to the conduct of the trial. For example, if significantly large and important treatment differences are observed during any of the interim analyses, the DSMB may recommend that randomization of patients be stopped, or that the design and conduct of the trial be appropriately modified.

We anticipate that the DSMB will meet at approximately 6-month intervals to review the accumulating data. Before each DSMB meeting, the Data Coordinating Center will conduct the desired statistical analyses and prepare a summary report for review by the DSMB. The extracted data files and analysis programs for each DSMB report will be maintained at the Data Coordinating Center for the life of the study. Each report will describe the progress in enrollment, the rates of compliance with therapy.

The DSMB may recommend stopping the trial for any safety-related concern at any time. Particular attention will be paid to all-cause mortality. For effectiveness, the trial will compare doses of aspirin that have been proven effective in previous trials and thus it is unlikely that any dramatic differences will appear early. However, the DSMB will monitor these differences about every 6 months over the 2.5 years of the trial. Thus, we anticipate four interim analyses and one final analysis. Because there are multiple tests, each test must be made at an adjusted α level using group sequential-testing methods. To minimize the chance of stopping early due to a spurious result, the endpoints will be tested at the planned analyses using a specific method known as the Haybittle-Peto rule.[109] This rule tests the endpoints at the 0.001 level ($z = 3.29$, two-sided) at each interim review and then makes the final test at the 0.0499 level ($z = 1.9605$). Thus, there is only minimal penalty for interim analyses.

The decision-making structure and process for the DSMB will be described in a separate DSMB charter.

III.A.7.f. Data Auditing Conventions

Data will be collected via the study patient portal (**Section III.A.3.d**).Data will be queried at the time of data entry through the study patient portal for missingness and inconsistencies.

IV. Human Subjects

IV.A. Protection of Human Subjects

IV.A.1. Human Subjects Involvement and Characteristics

IV.A.1.a. Inclusion Criteria

1. Known atherosclerotic cardiovascular disease (ASCVD), defined by a history of prior myocardial infarction, prior coronary angiography showing ≥75% stenosis of at least one epicardial coronary vessel, or prior coronary revascularization procedures (either PCI or CABG)
2. Age ≥ 18 years
3. No known safety concerns or side effects considered to be related to aspirin, including
    a. No history of significant allergy to aspirin such as anaphylaxis, urticaria, or significant gastrointestinal intolerances
    b. No history of significant GI bleed within the past 12 months
    c. Significant bleeding disorders that preclude the use of aspirin
4. Access to the Internet. In the event that the CDRNs are notified that a cohort of patients without internet access can be included, then patient agreement will be obtained during the consent process to provide follow-up information by telephone contact with the DCRI Call Center.
5. Not currently treated with an oral anticoagulant – either warfarin or a novel anticoagulant (dabigatran, rivaroxaban, apixaban, edoxaban) – and not planned to be treated in the future with an oral anticoagulant for existing indications such as atrial fibrillation, deep venous thrombosis, or pulmonary embolism.
6. Not currently treated with ticagrelor and not planned to be treated in the future with ticagrelor.
7. Female patients who are not pregnant or nursing an infant
8. Estimated risk of a major cardiovascular event (MACE) > 8% over next 3 years as defined by the presence of at least one or more of the following enrichment factors:
    a. Age > 65 years
    b. Serum creatinine > 1.5 mg/dL
    c. Diabetes mellitus (Type 1 or Type 2)
    d. 3-vessel coronary artery disease
    e. Cerebrovascular disease and/or peripheral arterial disease
    f. Left ventricular ejection fraction (LVEF) < 50%

g. Current cigarette smoker.

There will be no exclusions for any upper age limit, comorbid conditions, or concomitant medications other than oral anticoagulants and ticagrelor that are used at the time of randomization, or are planned to be used during the study follow-up.

Simple, inclusive eligibility criteria will make enrollment easier, and will render study results more generalizable to a broader population of patients. We will exclude pregnant or lactating women (because of concern for the fetus or child), patients taking oral anticoagulants or likely to require an oral anticoagulant during trial follow-up (because of complex drug interactions and a projected excessive risk of bleeding), and patients at relatively low risk for cardiovascular events (ie, no enrichment factor because of the large number of outcomes needed to detect a clinically meaningful difference with the available sample size).

IV.A.1.b. Sources of Material

Data obtained from the study patient portal and from the CDM will be transferred to the DCRI along with unique patient identifiers. Data contained with the CDM will be obtained at each of the sites and transferred to the DCRI. The control of access to databases at DCRI will be managed centrally by the DCRI through user passwords linked to appropriate access privileges. This protects data from unauthorized view and modifications as well as inadvertent loss or damage. Within the secondary SAS databases, UNIX group access control will be used for maintaining similar security. The Sun workstation login will be secured by extensive user password facilities under UNIX.

IV.A.1.c. Potential Risks

Aspirin is approved by the United States Food and Drug Administration (FDA) for the secondary prevention of ischemic events associated with ASCVD. In patients with established atherosclerosis, the risk of aspirin is quite low compared with the benefit. A very small number of patients have a serious anaphylactic reaction or bronchoconstriction with nasal polyps when they use aspirin. The risk of intracranial hemorrhage is <0.04% per year. A modest number of patients develop serious gastrointestinal bleeding as a result of loss of the protective effect of prostaglandins on the gastric mucosa. A larger number of patients have gastrointestinal intolerance, but this is often transient and can be overcome. The absolute risk varies as a function of the trial entry criteria, but none of these major risks exceeds 5 events per 100 patients treated per year. Except for patients with preexisting asthma, no studies have described risk factors for these complications of aspirin use. However, because patients will be selected based on the absence of such known major intolerances to a dose of 325 mg of aspirin, the expected risk of these major events in this trial will be low.

IV.A.1.d. Recruitment and Informed Consent

Each CDRN will develop a recruitment process that will work best within their organization, utilizing the tools they have available and their local infrastructure. Prior to cohort identification, site investigators will be asked to endorse the protocol, and depending upon their preference, they will either determine each patient’s eligibility or will give permission for the CDRN, through its integrated health system members, to identify and contact potentially eligible patients. In the latter case, patients who meet

criteria for secondary prevention after a cardiovascular event will be identified using customized search algorithms unique to each network from their aggregated EHR systems. Broad agreement from both cardiovascular specialists and primary care physicians will be sought. Patients will be enrolled after providing electronic informed consent.

IV.A.1.e. Protection Against Risk

Please see Sources of Material above and Data and Safety Monitoring Plan below.

IV.A.1.f. Potential Benefits of the Proposed Research to the Research Participants

As discussed in **Section I.A.**, atherosclerosis leading to thrombotic events, and in particular ASCVD, represents the leading cause of death, morbidity, and disability and affects more than 150 million people worldwide. Despite remarkable progress in preventive and interventional approaches to atherosclerosis, ASCVD is expected to be an even more prominent cause of death and disability for the next 30 years. In technologically-developed countries, the major factor that contributes to this expansion is the aging of the population, such that, despite declining age-specific disease rates, the total disease burden increases because ASCVD eventually strikes a larger population of older adults. In developing countries, a major epidemic of atherosclerosis is occurring, concentrated at younger ages and presumably due to the spread of tobacco use, Westernization of diet, and a sedentary life-style.

The development of new biological and technological approaches to treating ASCVD is exciting, but maximizing the use of an inexpensive yet effective therapy is far more promising in reducing death and disability on a global scale. Numerous clinical trials have shown the clinical benefit of aspirin versus placebo in reducing vascular events, but the best dose of aspirin for the general population with ischemic heart disease has not been determined. Considering the global burden of ASCVD and that the affected population is growing rapidly, identifying the optimal dose of aspirin would save lives and prevent ischemic and bleeding events globally. For example, based on recent evidence suggesting a reduction in ischemic events with lower doses of aspirin, the odds ratio for an event with a dose of 81 mg versus 325 mg per day would be 0.84 (95% confidence interval [CI], 0.64–1.1). If borne out in a prospective clinical trial, and if the rate of death, MI, or stroke over ~18 months of treatment was 8% with 325 mg of aspirin (based on contemporary trials of aspirin use in patients with ischemic heart disease), then the expected event rate with 81 mg would be 6.8% (95% CI, 5.3–8.7), or ~12 events prevented for every 1000 patients treated. Considering the increasing global burden of ischemic heart disease, a 1.2% absolute reduction in events simply through optimal aspirin dosing would be of tremendous importance to public health.

IV.A.1.g. Importance of the Knowledge to Be Gained

The primary aim of this study is to determine the optimal dose of aspirin, the universally available effective therapy for ASCVD. Despite dozens of clinical trials involving over 100,000 patients, the most effective aspirin dose has not been identified. Nonrandomized studies have suggested that lower doses of aspirin may be associated with not only lower rates of bleeding but also reduced ischemic outcomes. Despite guideline recommendations for lower dose aspirin, over half of post-ACS patients in the US are discharged on a dose of 325 mg. Considering the worldwide epidemic of ASCVD and the potential

benefits of aspirin, identifying the optimal dose of aspirin would have substantial global health and economic effects similar to few other medical therapies.

Although the primary aim of this study is to determine the optimal dose of aspirin, this study also represents a new and efficient interactive model for designing and implementing clinical trials that aim to refine therapies already in use in contemporary clinical practice. Because we live in an era in which the number of effective (or potentially effective) therapies far exceeds our ability to evaluate them in prospective clinical trials using current methods, there is an urgent need to develop an approach to outcome-based trials that can greatly reduce the cost per patient. By following patients directly on the Internet and thereby avoiding the costs of clinic visits, lengthy case record forms and extensive site management, we believe that a new, more efficient, and less expensive model for trials can be developed that could be extended to more experimental comparisons. However, working through the issues of informed consent, data validation, events ascertainment, and compliance assessment will require acceptance of novel approaches to statistical sampling and an intense focus on communication with both patients and their physicians. In this era of increasing concern about both patient privacy and research integrity, such an approach to trial efficiency would be exceedingly difficult to pilot with untested therapies. Because this trial will test only doses of aspirin that are considered relatively safe and are widely used in current clinical practice, it provides a critically and globally important clinical issue with which to develop such new clinical trials methods.

This trial also provides an opportunity to explore the use of integrated health systems, EHRs, and patient reported outcomes as tools for performing clinical trials. A trial conducted almost exclusively over the Internet offers many potential benefits. First, patients would have access to a custom-built, patient centered patient portal that offers current information about symptom awareness, risk factor modification, and disease management and prevention. The patient portal would also serve as the primary mechanism for follow-up, with routine data entry by the patients themselves. Not only would this method examine the practicability of patient self-reporting, but it would potentially enable substantial savings in cost and resources (*e.g.*, costs associated with physician reporting). For physicians, use of the Internet in clinical trials could further broaden awareness and participation and, at the same time, facilitate the conduct of the study. For example, trial enrollment and follow-up could be immediate with use of the Internet, at any time, eliminating a need for traditional methods (e.g., face to face visits). Finally, the platform created by this trial will unite a far-reaching community of patients and their physicians with a common goal of refining an existing therapy to maximize its benefit relative to risk. It is likely that such knowledge will produce far greater global benefit than the introduction of many other "high-tech" approaches.

IV.B. Inclusion of Women

(see following section)

IV.C. Inclusion of Minorities

This Study will seek to enroll a diverse population representative of the broad population of patients with ASCVD. The CDRN medical practices are from communities representing a wide range of sex, age,

racial, and ethnic backgrounds. We will make every attempt to explain the project in easy (non-clinical) terms to all patients to make sure they understand the importance of the research and the need to have good representation of key subgroups and they appreciate the potential benefit they could derive from participating in it. A particular advantage for this trial is the relative lack of barriers to participation. Because clinic visits are not needed for follow-up, additional transportation is not required, and there is minimal additional time commitment for patients. With regard to the specific enrollment of women, this is discussed in detail in **Section III.A.2.a-b**. Strategies to enhance the enrollment of ethnic minorities have been described in detail in **Section III.A.2.a-b.** We will strive to enroll a proportion of minority patients similar to that in the overall ASCVD population. Content on the ADAPTABLE patient portal will be available in English and Spanish.

IV.D. Inclusion of Children

Because participation in this trial is restricted to patients aged ≥18 years, inclusion of children in this trial is prohibited. Since the incidence of coronary artery disease among young (i.e., <18 years) individuals is very low, children do not represent a study population relevant to this trial.

IV.E. Data and Safety Monitoring Plan

This has been described in detail in **Section III.A.7** (Study Coordination and Monitoring).

Appendix: Patient Survey

Aspirin Trial Questions

Have you had a history of MI (myocardial infarction?)

- ○ Yes
- ○ No
- ○ Don't know

Have you had a history of CAD (Coronary artery disease?)

- ○ Yes
- ○ No
- ○ Don't know

Do you take any of these medications on a regular basis?

Mark all that apply

- ☐ Aspirin 81mg "baby aspirin"
- ☐ Aspirin 325mg "regular aspirin"
- ☐ Aspirin at another dose
- ☐ Apixaban (Eliquis)
- ☐ Clopidogrel (Plavix)
- ☐ Dabigatran (Pradaxa)
- ☐ Prasugrel (Effient)
- ☐ Rivaroxaban (Xarelto)
- ☐ Ticagrelor (Brilinta)
- ☐ Warfarin (Coumadin)

What dose of aspirin do you take?

Answer only if applicable

If you take Aspirin: How often do you usually take it?

Answer only if applicable

- ○ Every day, 2 times per day
- ○ Every day, 1 time per day
- ○ Every other day
- ○ Less often

Have you ever had to stop taking aspirin because of side effects like a rash, upset stomach or bleeding?

Answer only if applicable

- ○ Yes
- ○ No

If you had side effects from taking aspirin please list them

Answer only if applicable

Would you be willing to be a patient advisor to this study?

This may include: helping with study design, identifying ways to recruit study participants, helping develop a patient-friendly consent form, helping identify study outcomes important to patients, participating in the study's data safety monitoring process and helping interpret and disseminate results.

- ○ Yes
- ○ No

Submit

Never submit passwords through Google Forms.

References

1. Heart Disease Facts: America's Heart Disease Burden. Centers for Disease Control and Prevention, 2014. (Accessed February 5, 2015, at http://www.cdc.gov/heartdisease/facts.htm.)

2. Forrester AW, Lipsey JR, Teitelbaum ML, DePaulo JR, Andrzejewski PL. Depression following myocardial infarction. Int J Psychiatry Med 1992;22:33-46.

3. Schleifer SJ, Macari-Hinson MM, Coyle DA, et al. The nature and course of depression following myocardial infarction. Arch Intern Med 1989;149:1785-79.

4. Havik OE, Maeland JG. Patterns of emotional reactions after a myocardial infarction. . J Psychosom Res 1990;34:271-85.

5. Freedland KE, Carney RM, Lustman PJ, Rich MW, Jaffe AS. Major depression in coronary artery disease patients with vs. without a prior history of depression. Psychosom Med 1992;4:416-21.

6. Carney RM, Rich MW, Tevelde MA, Saini J, Clark K, Jaffe AS. Major depressive disorder in coronary artery disease. Am J Cardiol 1987;60:1273-5.

7. Hance M, Carney RM, Freedland KE, Skala J. Depression in patients with coronary artery disease: a 12-month follow up. Gen Hosp Psychiatry 1996;1996:61-5.

8. McKhann GM, Borowicz LM, Goldsborough MA, Enger C, Selnes OA. Depression and cognitive decline after coronary artery bypass grafting. Lancet 1997;349:1282-4.

9. PCORI. Limited PCORI funding announcement: Optimal maintenance aspirin dose for patients with coronary artery disease. 2014. (Accessed February 10, 2015, at http://www.pcori.org/announcement/limited-pcori-funding-announcement-optimal-maintenance-aspirin-dose-patients-coronary.)

10. Go AS, Mozaffarian D, Roger VL, et al. Heart Disease and Stroke Statistics—2014 Update A Report From the American Heart Association. Circulation 2014;129:e28-e292.

11. Global, regional, and national age–sex specific all-cause and cause-specific mortality for 240 causes of death, 1990–2013: a systematic analysis for the Global Burden of Disease Study 2013. Lancet 2015;385:117-71.

12. WHO Atlas of Heart Disease and Stroke. World Health Organization. (Accessed January 23, 2015, at http://www.who.int/cardiovascular_diseases/resources/atlas/en/.)

13. Collaborative overview of randomised trials of antiplatelet therapy--I: Prevention of death, myocardial infarction, and stroke by prolonged antiplatelet therapy in various categories of patients. Antiplatelet Trialists' Collaboration. BMJ 1994;308:81-106.

14. Antithrombotic Trialists' Collaboration. Collaborative meta-analysis of randomised trials of antiplatelet therapy for prevention of death, myocardial infarction, and stroke in high risk patients. BMJ 2002;324:71-86.

15. Kong DF, Hasselblad V, Kandzari DE, Newby LK, Califf RM. Seeking the optimal aspirin dose in acute coronary syndromes. Am J Cardiol 2002;90:622-5.

16. Yusuf S, Zhao F, Mehta SR, et al. Effects of clopidogrel in addition to aspirin in patients with acute coronary syndromes without ST-segment elevation. N Engl J Med 2001;345:494-502.

17. A randomised, blinded, trial of clopidogrel versus aspirin in patients at risk of ischaemic events (CAPRIE). CAPRIE Steering Committee. Lancet 1996;348:1329-39.

18. Hall HM, de Lemos JA, Enriquez JR, et al. Contemporary patterns of discharge aspirin dosing after acute myocardial infarction in the United States: results from the National Cardiovascular Data Registry (NCDR). Circ Cardiovasc Qual Outcomes 2014;7:701-7.

19. Xian Y, Wang TY, McCoy LA, et al. Dose with outcomes after acute myocardial infarction: Insights from the TRANSLATE-ACS Study. Circulation 2015;132:174-181.

20. Yusuf S, Reddy S, Ounpuu S, Anand S. Global burden of cardiovascular diseases: part I: general considerations, the epidemiologic transition, risk factors, and impact of urbanization. Circulation 2001;104:2746-53.

21. U.S. Food and Drug Administration. FDA's Sentinel Initiative. 2014. (Accessed February 10. 2015, 2015, at http://www.fda.gov/Safety/FDAsSentinelInitiative/ucm2007250.htm.)

22. U.S. Food and Drug Administration. Guidance for Industry: Q8(R2) Pharmaceutical Development. Center for Drug Evaluation and Research. 2009.

23. Gum PA, Thamilarasan M, Watanabe J, Blackstone EH, Lauer MS. Aspirin use and all-cause mortality among patients being evaluated for known or suspected coronary artery disease: A propensity analysis. JAMA 2001;286:1187-94.

24. Mauri L, Kereiakes DJ, Yeh RW, et al. Twelve or 30 months of dual antiplatelet therapy after drug-eluting stents. N Engl J Med 2014;371:2155-66.

25. Mehta SR, Bassand J-P, Chrolavicius S, et al. Dose comparisons of clopidogrel and aspirin in acute coronary syndromes. CURRENT-OASIS 7 Investigators. N Engl J Med 2010;363:930-42.

26. Mehta SR, Tanguay J-F, Eikelboom JW, et al. Double-dose versus standard-dose clopidogrel and high-dose versus low-dose aspirin in individuals undergoing percutaneous coronary intervention for acute coronary syndromes (CURRENT-OASIS 7): a randomised factorial trial. Lancet 2010;376:1233-43.

27. DiNicolantonio JJ, Serebruany VL. Exploring PLATO-USA Paradox and CURRENT-OASIS 7 Trials Reveals the Benefit of Higher Aspirin Dose. Am J Ther 2015;22:85-6.

28. A comparison of two doses of aspirin (30 mg vs. 283 mg a day) in patients after a transient ischemic attack or minor ischemic stroke. The Dutch TIA Trial Study Group. N Engl J Med 1991;325:1261-6.

29. Juul-Möller S, Edvardsson N, Jahnmatz B, Rosén A, Sørensen S, Omblus R. Double-blind trial of aspirin in primary prevention of myocardial infarction in patients with stable chronic angina pectoris. The Swedish Angina Pectoris Aspirin Trial (SAPAT) Group. Lancet 1992;340:1421-5.

30. Diener HC, Cunha L, Forbes C, Sivenius J, Smets P, Lowenthal A. European Stroke Prevention Study. 2. Dipyridamole and acetylsalicylic acid in the secondary prevention of stroke. J Neurol Sci 1996;143:1-13.

31. Farrell B, Godwin J, Richards S, Warlow C. The United Kingdom transient ischaemic attack (UK-TIA) aspirin trial: final results. J Neurol Neurosurg Psychiatry 1991;54:1044-54.

32. Taylor DW, Barnett HJ, Haynes RB, et al. Low-dose and high-dose acetylsalicylic acid for patients undergoing carotid endarterectomy: a randomised controlled trial. ASA and Carotid Endarterectomy (ACE) Trial Collaborators. Lancet 1999;353:2179-84.

33. Beneficial effect of carotid endarterectomy in symptomatic patients with high-grade carotid stenosis. North American Symptomatic Carotid Endarterectomy Trial Collaborators. N Engl J Med 1991;325:445-53.

34. Barnett HJ, Kaste M, Meldrum H, Eliasziw M. Aspirin dose in stroke prevention: beautiful hypotheses slain by ugly facts. Stroke 1996;27:588-92.

35. Bonaca MP, Bhatt DL, Cohen M, et al. Long-term use of ticagrelor in patients with prior myocardial infarction. N Engl J Med 2015;372:1791-800.

36. Gent M, Blakely JA, Easton JD, et al. The Canadian American Ticlopidine Study (CATS) in thromboembolic stroke. Lancet 1989;1:1215-20.

37. Balsano F, Rizzon P, Violi F, et al. Antiplatelet treatment with ticlopidine in unstable angina. A controlled multicenter clinical trial. The Studio della Ticlopidina nell'Angina Instabile Group. Circulation 1990;82:17-26.

38. Hass WK, Easton JD, Adams HP, et al. A randomized trial comparing ticlopidine hydrochloride with aspirin for the prevention of stroke in high-risk patients. Ticlopidine Aspirin Stroke Study Group. N Engl J Med 1989;321:501-7.

39. Leon MB, Baim DS, Popma JJ, et al. A clinical trial comparing three antithrombotic-drug regimens after coronary-artery stenting. Stent Anticoagulation Restenosis Study Investigators. N Engl J Med 1998;339:1665-71.

40. Quinn MJ, Fitzgerald DJ. Ticlopidine and clopidogrel. Circulation 1999;100:1667-72.

41. Yusuf S, Zhao F, Mehta SR, Chrolavicius S, Tognoni G, Fox KK. Effects of clopidogrel in addition to aspirin in patients with acute coronary syndromes without ST-segment elevation. N Engl J Med 2001;345:494-502.

42. Wiviott SD, Braunwald E, Angiolillo DJ, et al. Greater clinical benefit of more intensive oral antiplatelet therapy with prasugrel in patients with diabetes mellitus in the trial to assess improvement

in therapeutic outcomes by optimizing platelet inhibition with prasugrel–Thrombolysis in Myocardial Infarction 38. Circulation 2008;118:1626-36.

43. Becker RC, Bassand JP, Budaj A, et al. Bleeding complications with the P2Y12 receptor antagonists clopidogrel and ticagrelor in the PLATelet inhibition and patient Outcomes (PLATO) trial. Eur Heart J 2011;32:2933-44.

44. Tohgi H, Konno S, Tamura K, Kimura B, Kawano K. Effects of low-to-high doses of aspirin on platelet aggregability and metabolites of thromboxane A2 and prostacyclin. Stroke 1992;23:1400-3.

45. Patrono C, Coller B, Dalen JE, et al. Platelet-active drugs : the relationships among dose, effectiveness, and side effects. Chest 2001;119:39S-63S.

46. Voora D, Cyr D, Lucas J, et al. Aspirin exposure reveals novel genes associated with platelet function and cardiovascular events. J Am Coll Cardiol 2013;62:1267-76.

47. Gurbel PA, Bliden KP, DiChiara J, et al. Evaluation of dose-related effects of aspirin on platelet function: results from the Aspirin-Induced Platelet Effect (ASPECT) study. Circulation 2007;115:3156-64.

48. Savage MP, Goldberg S, Bove AA, et al. Effect of thromboxane A2 blockade on clinical outcome and restenosis after successful coronary angioplasty. Multi-Hospital Eastern Atlantic Restenosis Trial (M-HEART II). Circulation 1995;92:3194-200.

49. Serruys PW, Rutsch W, Heyndrickx GR, et al. Prevention of restenosis after percutaneous transluminal coronary angioplasty with thromboxane A2-receptor blockade. A randomized, double-blind, placebo-controlled trial. Coronary Artery Restenosis Prevention on Repeated Thromboxane-Antagonism Study (CARPORT). Circulation 1991;84:1568-80.

50. Frelinger AL, III, Li Y, Linden MD, et al. Association of cyclooxygenase-1-dependent and -independent platelet function assays with adverse clinical outcomes in aspirin-treated patients presenting for cardiac catheterization. Circulation 2009;120:2586-96.

51. Bhatt DL, Flather MD, Hacke W, et al. Patients with prior myocardial infarction, stroke, or symptomatic peripheral arterial disease in the CHARISMA Trial. J Am Coll Cardiol 2007;49:1982-8.

52. Tricoci P, Huang Z, Held C, et al. Thrombin-receptor antagonist vorapaxar in acute coronary syndromes. N Engl J Med 2012;366:20-33.

53. Morrow DA, Braunwald E, Bonaca MP, et al. Vorapaxar in the secondary prevention of atherothrombotic events. N Engl J Med 2012;366:1404-13.

54. Kopp E, Ghosh S. Inhibition of NF-kappa B by sodium salicylate and aspirin. Science 1994;265:956-9.

55. Frantz B, O'Neill EA, Ghosh S, Kopp E. The Effect of Sodium Salicylate and Aspirin on NF-κB. Science 1995;270:2017-9.

56. Pierce JW, Read MA, Ding H, Luscinskas FW, Collins T. Salicylates inhibit I kappa B-alpha phosphorylation, endothelial-leukocyte adhesion molecule expression, and neutrophil transmigration. J Immunol 1996;156:3961-9.

57. Yin M-J, Yamamoto Y, Gaynor RB. The anti-inflammatory agents aspirin and salicylate inhibit the activity of I[kappa]B kinase-[beta]. Nature 1998;396:77-80.

58. Stevenson MA, Zhao M-J, Asea A, Coleman CN, Calderwood SK. Salicylic acid and aspirin inhibit the activity of RSK2 kinase and repress RSK2-dependent transcription of cyclic AMP response element binding protein- and NF-κB-responsive genes. J Immunol 1999;163:5608-16.

59. Schwenger P, Bellosta P, Vietor I, Basilico C, Skolnik EY, Vilček J. Sodium salicylate induces apoptosis via p38 mitogen-activated protein kinase but inhibits tumor necrosis factor-induced c-Jun N-terminal kinase/stress-activated protein kinase activation. Proc Natl Acad Sci U S A 1997;94:2869-73.

60. Santos MT, Valles J, Aznar J, Marcus AJ, Broekman MJ, Safier LB. Prothrombotic effects of erythrocytes on platelet reactivity. Reduction by aspirin. Circulation 1997;95:63-8.

61. Weber C, Erl W, Pietsch A, Weber PC. Aspirin inhibits nuclear factor–κB mobilization and monocyte adhesion in stimulated human endothelial cells. Circulation 1995;91:1914-7.

62. Lou J, Povsic TJ, Allen JD, et al. The effect of aspirin on endothelial progenitor cell biology: preliminary investigation of novel properties. Thromb Res 2010;126:e175-9.

63. Steinhubl SR, Badimon JJ, Bhatt DL, Herbert J-M, Lüscher TF. Clinical evidence for anti-inflammatory effects of antiplatelet therapy in patients with atherothrombotic disease. Vasc Med 2007;12:113-22.

64. Marimuthu S, Chivukula RS, Alfonso LF, Moridani M, Hagen FK, Bhat GJ. Aspirin acetylates multiple cellular proteins in HCT-116 colon cancer cells: Identification of novel targets. International journal of oncology 2011;39:1273-83.

65. Lewis JP, Yerges-Armstrong LM, Ellero-Simatos S, Georgiades A, Kaddurah-Daouk R, Hankemeier T. Integration of pharmacometabolomic and pharmacogenomic approaches reveals novel insights into antiplatelet therapy. Clinical pharmacology and therapeutics 2013.

66. Suchindran S, Himmel T, Ortel TL, et al. Abstract 16260: Aspirin-Responsive Platelet Genes Are Associated With Platelet Function and Cardiovascular Events. Circulation 2014;130:A16260.

67. FitzGerald GA, Oates JA, Hawiger J, et al. Endogenous biosynthesis of prostacyclin and thromboxane and platelet function during chronic administration of aspirin in man. J Clin Invest 1983;71:676-88.

68. Voora D, Ortel T, Lucas J, Chi J-T, Becker R, Ginsburg G. Time-dependent changes in non-COX-1-dependent platelet function with daily aspirin therapy. J Thromb Thrombolysis 2012:1-12.

69. Mukherjee D, Nissen SE, Topol EJ. Risk of cardiovascular events associated with selective COX-2 inhibitors. JAMA 2001;286:954-9.

70. Szczeklik A. Adverse reactions to aspirin and nonsteroidal anti-inflammatory drugs. Ann Allergy 1987;59:113-8.

71. Allen LaPointe NM, Kramer JM, DeLong ER, et al. Patient-reported frequency of taking aspirin in a population with coronary artery disease. Am J Cardiol 2002;89:1042-6.

72. Final report on the aspirin component of the ongoing Physicians' Health Study. Steering Committee of the Physicians' Health Study Research Group. N Engl J Med 1989;321:129-35.

73. Garcia-Rodriguez LA, Cattaruzzi C, Troncon MG, Agostinis L. Risk of hospitalization for upper gastrointestinal tract bleeding associated with ketorolac, other nonsteroidal anti-inflammatory drugs, calcium antagonists, and other antihypertensive drugs. Arch Intern Med 1998;158:33-9.

74. Roderick PJ, Wilkes HC, Meade TW. The gastrointestinal toxicity of aspirin: an overview of randomised controlled trials. Br J Clin Pharmacol 1993;35:219-26.

75. Randomised double-blind trial of fixed low-dose warfarin with aspirin after myocardial infarction. Coumadin Aspirin Reinfarction Study (CARS) Investigators. Lancet 1997;350:389-96.

76. Comparison of sibrafiban with aspirin for prevention of cardiovascular events after acute coronary syndromes: a randomised trial. The SYMPHONY Investigators. Sibrafiban versus Aspirin to Yield Maximum Protection from Ischemic Heart Events Post-acute Coronary Syndromes. Lancet 2000;355:337-45.

77. Slattery J, Warlow CP, Shorrock CJ, Langman MJ. Risks of gastrointestinal bleeding during secondary prevention of vascular events with aspirin--analysis of gastrointestinal bleeding during the UK-TIA trial. Gut 1995;37:509-11.

78. Derry S, Loke YK. Risk of gastrointestinal haemorrhage with long term use of aspirin: meta-analysis. BMJ 2000;321:1183-7.

79. Laine L, Peterson WL. Bleeding peptic ulcer. N Engl J Med 1994;331:717-27.

80. Campbell CL, Smyth S, Montalescot G, Steinhubl SR. Aspirin dose for the prevention of cardiovascular disease: a systematic review. JAMA 2007;297:2018-24.

81. Manson JE, Stampfer MJ, Colditz GA, et al. A prospective study of aspirin use and primary prevention of cardiovascular disease in women. JAMA 1991;266:521-7.

82. Grosser T, Fries S, Lawson JA, Kapoor SC, Grant GR, FitzGerald GA. Drug resistance and pseudoresistance: an unintended consequence of enteric coating aspirin. Circulation 2013;127:377-85.

83. Peace A, McCall M, Tedesco T, et al. The role of weight and enteric coating on aspirin response in cardiovascular patients. J Thromb Haemost 2010;8:2323-5.

84. Williams MA, Fleg JL, Ades PA, et al. Secondary prevention of coronary heart disease in the elderly (with emphasis on patients > or =75 years of age): an American Heart Association scientific statement

from the Council on Clinical Cardiology Subcommittee on Exercise, Cardiac Rehabilitation, and Prevention. Circulation 2002;105:1735-43.

85. American Heart Association. 2001 Heart and Stroke Statistical Update. Dallas, Texas.

86. Roger VL, Go AS, Lloyd-Jones DM, et al. Heart disease and stroke statistics—2012 update: a report from the American Heart Association. Circulation 2012;125:e2-e200.

87. Berry JD, Dyer A, Cai X, et al. Lifetime risks of cardiovascular disease. N Engl J Med 2012;366:321-9.

88. Prevalence of Selected Chronic Conditions: United States, 1990-92. National Center for Health Statistics. Hyattsville, MD1993.

89. Street L, NW, Washington S, Inquiries DMFM. Internet User Demographics. Pew Research Center's Internet & American Life Project.

90. Street L, NW, Washington S, Inquiries DMFM. Offline Adults. Pew Research Center's Internet & American Life Project.

91. PCORnet. Data Standards. Security, and Network Infrastructure. 2014. (Accessed February 10, 2015, at http://www.pcornet.org/task-forces/data-standards-security-and-network-infrastructure/.)

92. Seniors as Web Users. 2013. (Accessed January 23, 2015, 2015, at http://www.nngroup.com/articles/usability-for-senior-citizens/.)

93. Wikipedia. Multitier architecture. 2015. (Accessed February 10, 2015, at http://en.wikipedia.org/wiki/Multitier_architecture.)

94. Wikipedia. Salt (cryptography). 2015. (Accessed February 10, 2015, at http://en.wikipedia.org/wiki/Salt_(cryptography)#Web_application_implementations.)

95. Wikipedia. Public-key cryptography. 2015. (Accessed February 10, 2015, at http://en.wikipedia.org/wiki/Public-key_cryptography.)

96. OAuth.net. OAuth 2.0 authorization framework. 2015. (Accessed February 10, 2015, at http://oauth.net/.)

97. PopMedNet - Homepage. (Accessed February 10, 2015, at http://www.popmednet.org/.)

98. Hlatky MA, Ray RM, Burwen DR, et al. Use of Medicare data to identify coronary heart disease outcomes in the Women's Health Initiative. Circ Cardiovasc Qual Outcomes 2014;7:157-62.

99. U.S. Food and Drug Administration. Welcome to Mini-Sentinel. 2014. (Accessed February 10, 2015, at http://mini-sentinel.org/.)

100. Kokotailo RA, Hill MD. Coding of stroke and stroke risk factors using international classification of diseases, revisions 9 and 10. . Stroke 2005;36:1776-81.

101. Iribarren C, Jacobs DR, Sadler M, Claxton AJ, Sidney S. Low total serum cholesterol and intracerebral hemorrhagic stroke: Is the association confined to elderly men? The Kaiser Permanente Medical Care Program. Stroke 1996;27:1993-8.

102. Arnason T, Wells PS, van Walraven C, Forster AJ. Accuracy of coding for possible warfarin complications in hospital discharge abstracts. Thromb Res 2006;118:253-62.

103. Sasieni P. Cox Regression Model. Encyclopedia of Biostatistics: John Wiley & Sons, Ltd; 2005.

104. Keiding N. Logrank Test. Encyclopedia of Biostatistics: John Wiley & Sons, Ltd; 2005.

105. Hintze JL. PASS User's Guide II. In: JL H, ed.2008.

106. Little RJ, D'Agostino R, Cohen ML, et al. The prevention and treatment of missing data in clinical trials. N Engl J Med 2012;367:1355-60.

107. Little RJA, Rubin DB. Statistical Analysis with Missing Data. 2 edition ed. Hoboken, N.J: Wiley-Interscience; 2002.

108. Juran JM. Juran on Quality by Design: The New Steps for Planning Quality into Goods and Services. Revised edition ed. New York : Toronto : New York: Free Press; 1992.

109. Haybittle JL. Repeated assessment of results in clinical trials of cancer treatment. Br J Radiol 1971;44:793-7.

110. PCORnet. PCORnet: A committment to patient privacy and data security. 2014. (Accessed February 10, 2015, at http://pcornet.org/wp-content/uploads/2014/12/PCORnet_PrivacyStatement_Final.pdf.)

Aspirin Dosing: A Patient-Centric Trial Assessing Benefits and Long-term Effectiveness (ADAPTABLE) Study Protocol

Version 4.0

Table of Contents

Abbreviations 6

I. Study Overview & Goals 8

I.A. Study Rationale 8

I.B. Study Aims 10

I.B.1. Comparison to DCRI Standard Metrics for Clinical Trials 11

I.B.2. PCORnet as a Network 11

I.B.3. CDRN and HPRN Experience 11

I.B.4. Patient Experience 11

II. Background and Significance 11

II.A. Significance of Aspirin Dosing: A Global Perspective 11

II.B. Optimal Aspirin Dosing in the Context of PCORnet: A New Model 13

II.C. Potential Impact of Proper Dosing of Aspirin 14

II.C.1. Benefit of Aspirin as Preventive Therapy 14

II.C.2. Aspirin Dose and Clinical Outcomes 14

II.C.3. Aspirin and Platelet $P2Y_{12}$ Inhibitors 16

II.C.4. Systematic Reviews and Mechanistic Insights into Aspirin Dosing 18

II.D. Aspirin: Mechanism, Clinical Benefit, and Adverse Events 19

II.D.1. Mechanism of Aspirin Effect on ASCVD 19

II.D.2. Aspirin Intolerance and Dose-Related Risks of Aspirin Therapy 20

II.E. Modifiers of Aspirin Dose 22

II.E.1. Aspirin, Minorities, and Other Subgroups 22

II.E.2. Enteric Coating 22

III. Research Design and Methods 23

III.A. Recruitment 23

III.A.1. Cohort Identification 23

III.A.2. Women, Elderly, and Minority Participants 23

III.A.3. Sources of Potential Bias 24

III.A.4. Patient Engagement 25

III.B. Enrollment and Eligibility 26

III.C. Study Design and Procedures 28

III.C.1. General Timeline 28

III.C.2. Consent, Randomization, and Drug Allocation 28

III.C.3. Concomitant Therapy 29

III.C.4. Schedule of Assessments 30

III.C.5. Data Collection and Follow-up 31

III.C.6. Developing and Refining the PCORnet Infrastructure 34

III.C.7. ADAPTABLE Patient Portal 34

III.D. Data Security and Back-up Procedures 35

III.D.1. Medidata Data Security for the Participant Portal 35

III.D.2. PopMedNet Data Security 36

III.D.3. Duke Clinical Research Institute Data Security 37

III.E. Endpoints and Adverse Events 38

III.E.1. Primary Endpoint 38

III.E.2. Secondary Endpoints 38

III.E.3. Safety Endpoints 38

III.E.4. Identifying Endpoints 38

III.E.5. Events Collection 41

III.F. Summary of Statistical Methods 42

III.F.1. Baseline Demographic, Clinical, Functional, and Procedural Characteristics 42

III.F.2. Populations for Analysis 42
III.F.3. Primary Effectiveness Comparison 42
III.F.4. Primary Safety Comparison 42
III.F.5. Power 42
III.F.6. Secondary Endpoints 44
III.F.7. Prior Treatment Effect 44
III.F.8. Handling of Missing Data 44
III.F.9. Subgroup Analyses (Heterogeneity of Treatment Effect) 45
III.F.10. Interim Blinded Trial Monitoring 46
III.G. Study Coordination and Monitoring 46
III. G.1. Site Management and Quality Assurance 46
III.G.2. Study Network 47
III.G.3. Executive Committee 49
III.G.4. Steering Committee 49
III.G.5. Data and Safety Monitoring Board (DSMB) 49
III.G.6. Data Auditing Conventions 50
IV. Human Subjects 50
IV.A. Protection of Human Subjects 50
IV.A.1. Sources of Data 50
IV.A.2. Potential Risks 50
IV.B. Diminution of Barriers 52
IV.C. Inclusion of Children 53
IV.D. Data and Safety Monitoring Plan 53
Appendix: Patient Survey for ADAPTOR Participants 54
References 56

Abbreviations

ACS	acute coronary syndrome
ACTION	Acute Coronary Treatment and Intervention Outcomes Network
AE	adverse event
ASCVD	atherosclerotic cardiovascular disease
CABG	coronary artery bypass graft
CAPRIE	Clopidogrel versus Aspirin in Patients at Risk of Ischaemic Events
CARS	Coumadin Aspirin Reinfarction Study
CDM	Common Data Model (PCORnet)
CDRN	Clinical Data Research Network
CER	comparative-effectiveness research
CKD	chronic kidney disease
CRF	case report form
CURE	Clopidogrel in Unstable Angina to Prevent Recurrent Events
CURRENT-OASIS	Clopidogrel optimal loading dose Usage to Reduce Recurrent EveNTs-Organization to Assess Strategies in Ischemic Syndromes
DAPT	dual antiplatelet therapy
DCRI	Duke Clinical Research Institute
DRN	distributed research network
DSMB	Data and Safety Monitoring Board
EHR	electronic health record
FDA	Food and Drug Administration (US)
GCP	Good Clinical Practice
GI	gastrointestinal
GWTG	Get with the Guidelines
HPRN	Health Plan Research Network
HR	hazard ratio
HVAC	heating, ventilation, air conditioning
IDS	intrusion detection system
IRB	institutional review board
ITT	intention to treat
MACE	major adverse cardiovascular events

MI	myocardial infarction
NFκB	nuclear factor kappa B
NSAID	nonsteroidal antiinflammatory drugs
PCI	percutaneous coronary intervention
PCORnet	National Patient-Centered Clinical Research Network
PHI	protected health information
PLATO	Platelet Inhibition and Patient Outcomes
PPI	proton-pump inhibitor
PPV	positive predictive value
RR	relative risk
SAE	serious adverse event
SAP	statistical analysis plan
SYMPHONY	Sibrafiban versus aspirin to Yield Maximum Protection from ischemic Heart events post acute cOroNary sYndromes
TIA	transient ischemic attack
UI	user interface
UPS	uninterruptable power service
VM	virtual machine
VPN	virtual private network

I. Study Overview & Goals

I.A. Study Rationale

Every year 720,000 Americans have a heart attack, and nearly 380,000 die of atherosclerotic cardiovascular disease (ASCVD).[1] Many of the patients who survive develop heart failure, stroke, and/or other cardiovascular complications. As such, patients with ASCVD and their caregivers suffer from substantial symptomatic, emotional, and functional difficulties. These patients often experience chest pain, shortness of breath, and fatigue, which can lead to significant distress and worsening quality of life. Rates of mental health illnesses such as depression are high among both these patients and their caregivers; rates of depression may approach 66% in post-myocardial infarction (MI) patients.[2-8] Coronary heart disease alone costs the US $108.9 billion each year.[1] This includes the cost of healthcare services, medications, and lost productivity.[1]

Aspirin is a mainstay therapy for patients with ASCVD. Introduced as a medicinal product more than 100 years ago, aspirin significantly reduces ischemic outcomes such as MI and stroke in patients with previous cardiovascular events and/or atherosclerosis at a cost of less than a cent per day. However, despite dozens of clinical trials involving more than 200,000 patients, the optimal dose of aspirin—the dose that is most effective in reducing ischemic events in the setting of secondary prevention, balanced by the potential for adverse events (AEs) such as gastrointestinal (GI) bleeding—has not been determined in direct comparative-effectiveness trials. Observational studies and indirect comparisons of different doses of aspirin have yielded conflicting results. Although most studies have found that lower-dose aspirin is associated with less bleeding, these studies have provided contradictory evidence regarding the comparative effectiveness of low- vs higher-dose aspirin in reducing ischemic events. Additional evidence raises the possibility that patients with different underlying characteristics may benefit most from different doses of aspirin.

To identify the optimal dose of aspirin for secondary prevention in patients with ASCVD, we propose a pragmatic clinical trial in which 15,000 patients who are at high risk for ischemic events will be randomly assigned in a 1:1 ratio to receive an aspirin dose of 81 mg/day vs 325 mg/day. Study participants will be enrolled over 38 months. Maximum follow-up will be 50 months. The primary endpoint is a composite of all-cause death, hospitalization for MI, or hospitalization for stroke. The primary safety endpoint is hospitalization for major bleeding with an associated blood product transfusion.

The ADAPTABLE trial study design is shown in the schematic below.

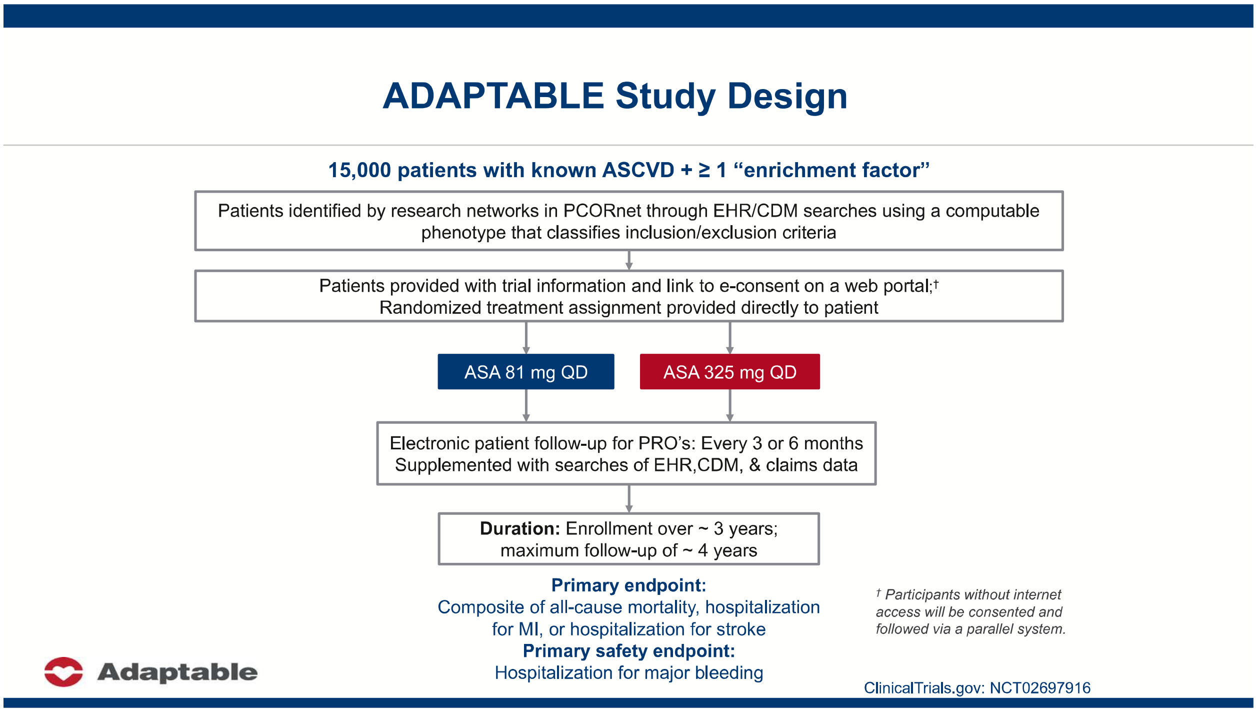

ASA, acetylsalicylic acid; ASCVD, atherosclerotic cardiovascular disease; CDM, Common Data Model; CDRN, Clinical Data Research Network; EHR, electronic health record; MI, myocardial infarction; PPRN, Patient-Powered Research Network; QD, once daily

Enrollment and follow-up of study participants will be conducted using highly streamlined methods, with electronic-health-record (EHR) data organized according to the recently developed PCORnet (National Patient-Centered Clinical Research Network) Common Data Model (CDM) format and stored in a PCORnet DataMart,* complemented where possible by existing data sources (Medicare and private health plan claims data) and patient-reported outcomes. Additional information will be collected via streamlined forms to be completed by participants either by internet if they are able to access the internet or by the Call Center at the Duke Clinical Research Institute (DCRI). This project constitutes the initial randomized comparative-effectiveness trial conducted by PCORnet; http://www.pcornet.org/.[9]

This trial will incorporate several essential aspects of the new genre of patient-centered comparative-effectiveness trials:

---

* Please note: throughout this protocol, the term "Common Data Model" (CDM) is used to refer both to the format used for standardizing and organizing information, and also as shorthand for electronic-health-record data that are extracted and stored using the CDM format within the PCORnet DataMart.

1. By using existing data sources to gather baseline characteristics and a combination of existing data and patient-reported outcomes during follow-up, the trial will answer this critical question at a relatively low cost.
2. An internet portal will enable the trial to collect and monitor data and enable mutual learning by both patients and clinicians, capitalizing on the frequent use of the internet by the US public and clinicians.
3. The trial will not have a placebo control, but instead will provide all patients with active treatment at different doses, with monitoring to balance benefit and risk.
4. Patient-reported outcomes will be collected.
5. The evolving PCORnet infrastructure will be used to attempt to streamline administrative aspects of the trial, including the use of a web-based, electronic informed consent process and use of EHR data standardized into the CDM format for baseline data collection and centralized patient follow-up.

I.B. Study Aims

We have defined the following specific aims for this study:

- **Aim 1:** To compare the effectiveness of 2 daily doses of aspirin (81 mg and 325 mg) in reducing a composite endpoint of all-cause death, hospitalization for nonfatal MI, or hospitalization for nonfatal stroke in high-risk patients with a history of MI or documented ASCVD. Secondary endpoints will be the components of the composite primary endpoint as well as coronary revascularization procedures (percutaneous coronary intervention [PCI] or coronary artery bypass grafting [CABG]) performed during study follow-up. The primary safety endpoint will be hospitalization for major bleeding complications with an associated blood product transfusion.
- **Aim 2:** To compare the effects of aspirin in selected subgroups of patients, including women vs men, older vs younger patients, racial minority patients vs white patients, patients with vs without diabetes, patients with vs without chronic kidney disease (CKD), and patients treated with a platelet $P2Y_{12}$ inhibitor vs those not treated with a platelet $P2Y_{12}$ inhibitor.
- **Aim 3:** To develop, refine, and evaluate the infrastructure for PCORnet to conduct multiple comparative-effectiveness trials in the future. This aim will be accomplished with a “phased-in” approach that will allow for an initial testing of the PCORnet infrastructure followed by adjustments to the trial operational plan to most efficiently accomplish Aims 1 and 2. Also, we will carefully monitor the recruitment and enrollment patterns within and across participating Clinical Data Research Networks (CDRNs) and a Health Plan Research Network (HPRN) and will provide regular feedback reports to each CDRN and HPRN to promote consistent recruitment practices. Potential metrics to evaluate the success of ADAPTABLE are listed below and will be finalized with the PCORnet leadership in the context of other performance measures in development PCORnet-wide:

I.B.1. Comparison to DCRI Standard Metrics for Clinical Trials

- Time to institutional review board (IRB) approval
- Time to contract approval
- Time to first site activation
- Time to first patient enrolled
- Recruitment rate
- Retention
- Withdrawn consents
- Drug discontinuation
- Lost to follow-up
- Missed study contacts
- Data quality

I.B.2. PCORnet as a Network

- Ability to support widespread screening, contact, enrollment, and follow-up of patients across the networks

I.B.3. CDRN and HPRN Experience

- Administrative simplicity (i.e., IRB share model; contracts)
- Participation, engagement, and leadership

I.B.4. Patient Experience

- Assess electronic consent process and patient experience
- Evaluate experiences of participating patients

II. Background and Significance

II.A. Significance of Aspirin Dosing: A Global Perspective

ASCVD that leads to ischemic events represents the leading cause of death, morbidity, and disability.[10] Despite remarkable progress in prevention and treatment for atherosclerosis, ASCVD is expected to be an even more prominent cause of death and disability over the next 30 years.[11] In high-income countries, the major factors contributing to this expansion are the aging of the population coupled with increases in incidence of obesity, diabetes, and sedentary lifestyle. Despite declining age-specific disease rates, the total disease burden increases as ASCVD eventually affects a larger population of older adults. In economically developing countries, a major epidemic of atherosclerosis is occurring, concentrated in younger age groups and presumably due to increasing tobacco use as well as obesity and diabetes arising from Westernization of diets and lack of exercise.[12]

The development of new biological and technological approaches to treating ASCVD is exciting, but maximizing the use of an inexpensive yet effective therapy shows more promise for reducing death and disability on a global scale. Considering the burden of ASCVD and that the population affected by it is growing rapidly, identifying the optimal aspirin dose will save lives and prevent ischemic and bleeding events globally.

For example, based on recent evidence suggesting a reduction in ischemic events with lower doses of aspirin, the odds ratio (OR) for an event with an aspirin dose of 81 mg/day vs 325 mg/day would be 0.84 (95% CI, 0.64–1.1).[13,14] If the rate of death, MI, or stroke in a prospective clinical trial over approximately 18 months of treatment was 8% with 325 mg of aspirin (based on contemporary trials of aspirin use in patients with ischemic heart disease),[13,15,16] then the expected event rate with 81 mg would be 6.8% (95% CI, 5.3–8.7), or approximately 12 events prevented for every 1,000 patients treated. Given the magnitude of the global burden of ischemic heart disease, a 1.2% absolute reduction in events achieved simply through optimal aspirin dosing would be of tremendous importance to public health.

Until recently, aspirin dosing patterns after acute MI in the US were uncertain. A 2014 analysis of the National Cardiovascular Data Registry's Acute Coronary Treatment and Intervention Outcomes Network (ACTION) Registry-Get with the Guidelines (GWTG) examined aspirin dosing in 221,199 patients with acute MI (both ST-segment-elevation MI [STEMI]and non-STEMI) from 525 US hospitals.[17] Between January 2007 and March 2011, 61% of patients were discharged on 325 mg of aspirin, 36% on 81 mg, and 4% on other doses. The rate of use of 325 mg of aspirin at discharge was 73% in patients who underwent PCI vs 45% in patients managed medically (i.e., without invasive revascularization). When aspirin was used concomitantly with a thienopyridine and warfarin, a 325-mg dose was used in 44% of patients. Even among patients who experienced major in-hospital bleeding, 57% received the 325-mg dose. The relatively high rate of use of this dose, even in patients at high risk of bleeding, and the 25-fold variation in the rate of use of the 325-mg aspirin dose across participating centers, are surprising and likely reflect uncertainty regarding appropriate aspirin dosing.[17]

Further details on aspirin dosing patterns and the impact of high- vs low-dose aspirin among patients with acute MI undergoing PCI in the US from 2010 through 2012 were recently published from the TReatment with ADP receptor iNhibitorS: Longitudinal Assessment of Treatment Patterns and Events after Acute Coronary Syndrome (TRANSLATE ACS) registry.[18] Among 10,213 patients, 6,387 (62.6%) received high-dose (325 mg) aspirin at hospital discharge with substantial variability across the 228 hospitals in the analysis (median hospital-level frequency of high-dose aspirin use was 70%). The adjusted risks of ischemic outcomes (death, MI, stroke, or unplanned revascularization) and bleeding requiring hospitalization through 6 months were similar with high- vs low-dose (81 mg) aspirin. However, approximately 35% of patients discharged on high-dose aspirin were switched to low-dose aspirin within 6 months. These non-randomized findings, coupled with the findings from the ACTION Registry-GWTG analysis, highlight the substantial variability in aspirin dosing patterns in the US for patients with ASCVD who have experienced a recent acute MI event and directly point to the need to do an adequately powered, large-scale trial of low- vs high-dose aspirin to determine the most effective dose of aspirin for the secondary prevention of ASCVD.

In the US, the 2010 death rates attributable to coronary heart disease, stroke, and other cardiovascular diseases were 113.6, 39.1, and 82.7 per 100,000, respectively.[10] Globally, given the rapidly increasing burden of ASCVD and limited healthcare resources, particularly in lower-income countries, a similar benefit from identifying the best dose of aspirin for treating the general population with ischemic heart disease could translate to as many as 88,800 fewer deaths from ASCVD annually and would prevent approximately 145,000 deaths in 2020.[19] In the US alone, this would mean approximately 19,000 fewer deaths and MIs each year without employing new treatments or technology and with no additional healthcare expenditures.[10]

In addition to defining the best dose of aspirin from the population perspective, the subgroup analyses and model-based analyses of heterogeneity of treatment effect planned for this proposed trial will allow further insights into refinement of aspirin dosing at the patient level. Such knowledge could further enhance the benefit derived from aspirin treatment.

II.B. Optimal Aspirin Dosing in the Context of PCORnet: A New Model

Although the primary aim of this study is to determine the optimal dose of aspirin for secondary prevention of ASCVD, it also represents the initial use of a transformative approach to developing a new and efficient interactive model for designing and implementing clinical trials that aim to compare the effectiveness of therapies already in use in clinical practice (comparative-effectiveness research [CER]) using methods centered on the needs and experiences of patients. Because we live in an era in which the number of effective (or potentially effective) therapies far exceeds our ability to evaluate them in prospective clinical trials using current methods, we face an urgent need to develop an approach to CER trials that can greatly reduce the cost of trials while maintaining the quality, reproducibility, and generalizability of the research. By using existing data from EHRs organized into the CDM developed by PCORnet and derived from the Food and Drug Administration's (FDA's) Sentinel project,[20] the trial will develop initial experience with the use of the CDM to supplant costly and time-consuming data-collection approaches that are used with traditional clinical trials. By following the majority of participants on the internet and collecting minimal data directly from them, we can avoid the costs incurred by non-clinically indicated research visits, lengthy case report forms (CRFs), and extensive site management operational approaches.

We believe that a more efficient and less expensive model for trials can be developed that could be extended to more experimental comparisons. However, working through the issues of informed consent, data validation, events ascertainment, and compliance assessment will require acceptance of novel approaches to statistical sampling and "quality-by-design" principles[21] as well as communication with participants. Amid increasing concerns about participant privacy and research integrity, such an approach to trial efficiency would be difficult to pilot with untested therapies. Because this trial will test only doses of aspirin that are considered relatively safe and are widely used in current clinical practice, it presents a critically and globally important clinical issue with which to develop these new methods.

A trial designed to use existing data resources almost exclusively (supplemented by internet interaction with research participants and telephone contact from the DCRI Call Center for those without internet

access) offers many potential benefits. For example, clinicians will not be burdened with extensive data-collection forms and cumbersome consent and contracting procedures. In addition, the participant portal will serve as the primary mechanism for follow-up, with routine data entry by the participants themselves providing a concise set of patient-reported outcomes.

For physicians and other clinicians, application of the PCORnet CDM format and the participant portal to clinical trials could broaden awareness and participation and, at the same time, aid in conduct of the study. For example, trial enrollment and follow-up could be automated with use of the CDM and participant portal at any time, eliminating or greatly reducing the need for traditional methods such as telephone or postal mail contact, or extended in-person clinic visits. Instead, valuable clinician-participant interaction time can be focused on clinical care and answering questions that arise.

Finally, the platform created by this trial will unite a broad and diverse community of patients and their physicians around the common goal of refining the evidence underlying an existing therapy (aspirin) to maximize its benefit relative to risk. By integrating direct physician and patient participation in the examination of the relationships among clinical outcomes in response to various aspirin doses, this platform will also produce far greater global benefit than the introduction of many other “high-tech” approaches.

II.C. Potential Impact of Proper Dosing of Aspirin

II.C.1. Benefit of Aspirin as Preventive Therapy

Aspirin has a significant impact on the risk of vascular events in patients with known atherosclerosis. The Antiplatelet Trialists’ Collaboration reported a 20% to 40% reduction in the risk of death, MI, or stroke for study participants taking aspirin.[13] This benefit was clear for patients with coronary artery disease or cerebrovascular disease and for those with MI, unstable angina, transient ischemic attack (TIA), or stroke. More recently, aspirin therapy was associated with reduced long-term mortality among 6,174 patients undergoing stress echocardiography to evaluate known or suspected ASCVD (hazard ratio [HR] over approximately 3 years, 0.67; 95% CI, 0.51–0.87; *P*=0.002).[22] After adjustment for the propensity to use aspirin and other possible confounding variables, aspirin use remained associated with a lower risk of death (HR, 0.56; 95% CI, 0.40–0.78; *P*<0.001). Patient characteristics associated with the greatest reductions in mortality included advanced age, known ASCVD, and impaired exercise capacity. These results were recently replicated in a broad assessment of aspirin effectiveness across racial, ethnic, and sex subgroups.[23]

II.C.2. Aspirin Dose and Clinical Outcomes

Despite uncertainties about optimal aspirin dosing, consensus has developed regarding recommendations for aspirin dosing between 75 and 325 mg daily in patients with ASCVD. This practice has been driven primarily by commercial availability, physician preference, and concerns about AEs such as abdominal discomfort and GI bleeding associated with higher doses. Although this dose range has developed empirically over time, attempts to balance clinical benefit with adverse side effects largely reflect indirect comparisons performed in various clinical settings.

Few direct comparisons of different doses of aspirin have been performed, and their results have been inconclusive. The only large, direct prospective study performed to date, Clopidogrel optimal loading dose Usage to Reduce Recurrent EveNTs-Organization to Assess Strategies in Ischemic Syndromes (CURRENT-OASIS 7),[24] evaluated outcomes among patients with acute coronary syndromes (ACSs) over the first 30 days only. The results were complex because the trial used a factorial design of high- and low-dose aspirin with high- and low-dose clopidogrel starting at the time of the index ACS event.[24,25] Although the factorial analysis showed the most favorable outcomes for the combination of high-dose aspirin and high-dose clopidogrel, the results were not definitive, and a variety of different interpretations have been offered by experts.[26] Thus, while indirect evidence exists for dose-dependent efficacy of aspirin in preventing vascular events, it is equally clear that no adequately sized randomized trials have addressed this issue, particularly in patients with established ASCVD who are receiving long-term treatment for secondary prevention. The suggestive (but not definitive) data supporting lower aspirin dose emphasize a clear need for larger randomized studies of aspirin dosing in ASCVD.

Various aspirin doses, even as low as 30 mg/day, have been shown to be effective in preventing vascular events.[27] A trial of unstable angina patients found a dose of 75 mg/day to be effective in reducing recurrent vascular events,[28] and the European Stroke Prevention Trial found a benefit with 25 mg twice daily in preventing stroke or death in high-risk patients.[29]

Varied trial results have underscored the uncertainty about aspirin dosing in secondary prevention. A study of secondary prevention after TIA compared 300 mg/day with 1200 mg/day and found no difference in efficacy.[30] Similarly, the Dutch TIA prevention study found no difference in efficacy between 30 and 283 mg of aspirin per day.[27] A trial comparing aspirin doses after carotid endarterectomy found a lower risk of the composite of death, MI, or stroke with daily doses of 81 or 325 mg/day vs. 650 or 1300 mg/day.[31] This study contradicted earlier findings of a lower event rate with doses of 650 mg or more vs. 325 mg or less per day for prevention of perioperative stroke.[32,33]

In what had been the largest experience for many years, the Antiplatelet Trialists' Collaboration's systematic review of 11 trials of antiplatelet therapy, there was no apparent dose-response relationship of aspirin in secondary prevention of cardiovascular outcomes.[13] When the investigators expanded their meta-analysis to include subsequent trials of antiplatelet therapies,[14] they found that compared with no aspirin therapy, high doses of aspirin (500–1500 mg/day) were not clearly more effective in reducing ischemic vascular events than doses of 75 to 150 mg/day. Specifically, the proportional reduction in vascular events was 19% with 500 to 1500 mg/day, 26% with 160 to 325 mg/day,

and 32% with 75 to 150 mg/day **(Table 1).**

II.C.3. Aspirin and Platelet P2Y$_{12}$ Inhibitors

Platelet P2Y$_{12}$ inhibitors (ticlopidine, clopidogrel, prasugrel, ticagrelor) reduce the risk of major adverse cardiovascular events (MACE) when added to aspirin (termed dual antiplatelet therapy, or DAPT) in patients with STEMI, ACS, and PCI. The recommended duration of DAPT for these indications has been approximately 1 year, but the recently completed DAPT and Prevention of Cardiovascular Events in Patients With Prior Heart Attack Using Ticagrelor Compared to Placebo on a Background of Aspirin (PEGASUS) trials[23,34] suggest that extended durations of DAPT for an additional 18 to 36 months (beyond the standard 12-month treatment period) after PCI with coronary stent placement or after a prior MI may provide longer-term benefit. Observational, non-randomized comparisons indicate that lower-dose aspirin may be associated with better outcomes when DAPT is used. However, no sizable randomized comparisons of aspirin dosing are available wherein aspirin was used in combination with P2Y$_{12}$ inhibitors except for the CURRENT-OASIS-7 trial, which only evaluated patients for 30 days after the index ACS event, as previously mentioned.[24]

Table 1. Aspirin Dosing and Ischemic Events: ATC

		Ischemic Event Rate		
Aspirin Dose	Trials (n)	Aspirin	Control	Relative Reduction
500–1500 mg	34	14.5%	17.2%	19%
160–325 mg	19	11.5%	14.8%	26%
75–150 mg	12	10.9%	15.2%	32%
<75 mg	3	17.3%	19.4%	13%
Any dose	65	12.9%	16.0%	23%

Thienopyridine platelet inhibitors have been in clinical use for more than 2 decades. Ticlopidine, the prototype, was shown to reduce ischemic events compared with placebo in a series of clinical trials,[35,36] to be marginally superior to aspirin in 1 trial of cerebrovascular disease,[37] and to provide additive benefit to aspirin after PCI.[38] Clopidogrel is structurally similar to ticlopidine but is associated with substantially fewer serious AEs (SAEs). Most notably, ticlopidine has been associated with the development of neutropenia, thrombocytopenia, and thrombotic thrombocytopenic purpura;[39] such events have not been associated with clopidogrel use in large clinical trials.

The first major study of clopidogrel was the Clopidogrel versus Aspirin in Patients at Risk of Ischaemic Events (CAPRIE) trial, which showed a modest but measurable benefit over aspirin (relative risk [RR] reduction, 8.7%; P=0.043) in the secondary prevention of ischemic stroke, MI, or vascular death among patients with vascular disease.[16]

The Clopidogrel in Unstable Angina to Prevent Recurrent Events (CURE) trial compared the effectiveness of DAPT with aspirin and clopidogrel vs. aspirin alone in patients with ACS.[40] At a mean follow-up of 9 months, there was a 22% relative reduction in the composite endpoint of death, MI, or stroke with combination therapy (9.3% with clopidogrel + aspirin vs 11.4% with aspirin alone; $P < 0.001$). Although major bleeding occurred significantly more often with combined therapy (3.7% with aspirin + clopidogrel; 2.7% with aspirin alone; $P = 0.001$), there was no excess in major bleeding in the clopidogrel group after the first 30 days, suggesting that most of the risk was related to early revascularization procedures. Although CURE was not a formal study of secondary prevention (follow-up period was 1 year) and aspirin therapy ranged from 75 to 325 mg/day, the trial found benefit during 1 year of follow-up. Of note, aspirin dosing in the CURE trial was left to the treating physician's discretion and was

not part of the randomized treatment assignment. With increasing aspirin doses, however, trends toward higher rates of ischemic and bleeding events were observed for patients in both study arms (**Table 2**), with the lowest rates of bleeding and ischemic events observed in patients taking an aspirin dose of less than 100 mg/day.[40]

Table 2. Effect of Aspirin Dosing on Ischemic Events and Bleeding in the CURE Trial

Aspirin Dose	Death, MI, Stroke			Life-threatening Bleeding	Any Bleeding
	Clopidogrel+ Aspirin	Aspirin	HR (95% CI)		
<100 mg (n=1927)	80 (8.5%)	96 (9.7%)	0.86 (0.64–1.16)	1.2%	1.9%
110–161 mg (n=7428)	345 (9.2%)	402 (10.9%)	0.84 (0.73–0.97)	1.7%	2.3%

Conflicting results have now been reported for prasugrel and ticagrelor. The Efficacy and Safety of Initial Triple Versus Initial Dual Oral Combination Therapy in Patients With Newly Diagnosed Pulmonary Arterial Hypertension (TRITON) trial compared clopidogrel vs prasugrel added to aspirin therapy for patients with ACS undergoing PCI and found no evidence for an interaction between aspirin dose and treatment effect of prasugrel relative to clopidogrel for key outcomes, nor did it find a difference in outcomes as a primary function of aspirin dose.[41] In contrast, an observational analysis of the Platelet Inhibition and Patient Outcomes (PLATO) trial[42] found that lower doses of aspirin were associated with less bleeding and fewer ischemic events in patients receiving ticagrelor. Further, there was a significant interaction between aspirin dose and the treatment benefit of ticagrelor, which overall reduced total mortality compared with clopidogrel. In the PLATO trial, patients receiving low-dose aspirin had a significant reduction in death and MACE if they were randomized to ticagrelor, whereas patients on high-dose aspirin fared equally well with clopidogrel and ticagrelor. However, it should be noted that this comparison of the impact of concomitant aspirin doses with the treatment effect of ticagrelor was nonrandomized and was subject to a significant amount of bias based upon regional differences in the concomitant aspirin dose across the multiple countries that participated in the PLATO trial. Nonetheless, the approval of ticagrelor for the treatment of ACS by the FDA in the US incorporated a "black box" warning for avoiding aspirin doses greater than 100 mg together with ticagrelor.[43]

Notwithstanding the relative lack of randomized evidence regarding the optimal dose of aspirin when it is used together with a $P2Y_{12}$ inhibitor during DAPT treatment, recently published US guidelines for the duration of DAPT treatment give a Class IB-NR recommendation for the use of low-dose aspirin (< 100 mg/day) when aspirin is used together with any $P2Y_{12}$ inhibitor.[44] The data supplement within the guidelines document underlying this specific recommendation cites numerous nonrandomized, post-hoc analyses that have investigated different aspirin doses both with and without concomitant use of $P2Y_{12}$ inhibitors, except for the aforementioned CURRENT-OASIS 7 trial[24] that evaluated patients for only 30 days after the index ACS event. However, the guidelines committee specifically endorsed the goals and objectives of the ADAPTABLE trial to determine the optimal dose of aspirin with concomitant DAPT treatment.

In summary, the appropriate dose of aspirin in conjunction with a $P2Y_{12}$ inhibitor is unknown despite the recent guidelines recommendation. The ADAPTABLE trial investigators recognize the equipoise and uncertainty that exist regarding this important clinical question and will investigate this issue by conducting a subgroup analysis according to the concomitant use of a $P2Y_{12}$ inhibitor. Patients who are treated with either clopidogrel or prasugrel will be eligible to be included in the study, but patients treated with ticagrelor will be excluded, given the FDA “black box” warning for the use of high-dose aspirin with ticagrelor.[43]

II.C.4. Systematic Reviews and Mechanistic Insights into Aspirin Dosing

To evaluate the influence of aspirin dosing, a random effects model was used for combining data from 11 randomized, placebo-controlled trials of aspirin in the Antiplatelet Trialists’ Collaboration’s dataset of 14,810 patients.[14] This modeling technique allows adjustments for patient populations and time trends, 2 limitations inherent in indirect comparisons. Aspirin doses evaluated in this meta-analysis, which examined endpoints including death, acute MI, and stroke (but not bleeding), ranged from 50 mg to 1.5 g daily. Overall, compared with placebo, aspirin resulted in significant reductions in death and death or MI, yet there was a significant decrease in estimated effectiveness as aspirin dose increased (OR for an event: 1.14 with each doubling of aspirin dose; P=0.007). The analysis also suggested that the study population influenced the effect of aspirin, with a greater benefit observed among patients with unstable angina vs MI. For the post-infarction subgroup, the OR was 0.76 (95% CI, 0.66–0.88); for the unstable angina subgroup, the OR was 0.55 (0.43–0.71). Such findings also imply a need for larger randomized comparisons of aspirin dosing in patients with ASCVD.

Although recent observational studies using indirect or nonrandomized comparisons of outcome by aspirin dose suggest that lower doses of aspirin are associated with fewer ischemic events, the possibility remains that in a randomized comparison, higher doses of aspirin may be more favorable. For example, if we consider that aspirin resistance as defined by elevated urinary thromboxane B_2 levels is associated with worse outcomes and that aspirin 81 mg/day provides less effective thromboxane suppression than aspirin 325 mg/day,[45] we might expect that the latter dose would be associated with better clinical outcomes.

Further, if we postulate that, given its relatively weak antiplatelet effects, an important mechanism of aspirin in prevention of ischemic events is via its antiinflammatory properties resulting from cyclooxygenase (COX)-2 suppression of platelets or inflammatory cells, similar questions arise about the expected optimal dose. Because 1) aspirin is approximately 50 to 100 times more potent in inhibiting platelet COX-1 than monocyte-derived COX-2; and 2) inhibition of COX-2–dependent processes (e.g., inflammation) require larger doses of aspirin (because nucleated cells rapidly resynthesize the enzyme), higher aspirin doses may be required for efficacy.[46] Therefore, if an antiinflammatory effect of aspirin is important in its ability to prevent ischemic events, then we might expect daily aspirin doses of 162 or 325 mg to be more effective than 81 mg.

In addition, recent research has found alternative pathways for aspirin's effect on platelet aggregation.[47] These pathways may account for the need for different doses in various individuals and subgroups of patients.

These theoretical and mechanistic arguments highlight the need for a randomized comparison of the effect of aspirin dosing on clinical outcomes that avoids any potential for selection bias toward lower-risk patients receiving lower aspirin doses from their physicians. It also highlights the possible value of mechanistic substudies as a part of this effort to advance our understanding of the factors that identify and modify aspirin response and their relationships with clinical outcomes.

II.D. Aspirin: Mechanism, Clinical Benefit, and Adverse Events

II.D.1. Mechanism of Aspirin Effect on ASCVD

Aspirin's putative mechanism of action for preventing cardiovascular events is through irreversible inhibition of platelet COX-1, which prevents the conversion of arachidonic acid to prostaglandin H2—the immediate precursor to thromboxane A2 (TXA2), a potent platelet agonist. Until recently, most experts assumed that the benefit of aspirin solely reflected the inhibition of platelet aggregation. Low-dose aspirin (~80 mg/day) is sufficient to maximally inhibit platelet COX-1 and COX-1–dependent measures of platelet aggregation; higher aspirin doses do not produce additional COX-1 inhibition.[48] Consistent with this finding, selective TXA2 receptor inhibitors have shown no benefit compared with either aspirin or placebo.[49,50]

With 325 mg/day aspirin dosing, there appear to be additional effects on inhibiting measures of platelet function, particularly from the perspective of non–COX-1 dependent assays (e.g., collagen- and high-dose ADP-induced platelet aggregation).[48] The consequences of this additional, apparently non-COX mediated platelet inhibition with 325 mg/day dosing on clinical outcomes are unknown. However, residual platelet aggregation of non-COX-1–dependent platelet aggregation in aspirin-treated patients is associated with future cardiovascular events.[51] Further, it is clear that additional platelet inhibition in excess of that produced by low-dose aspirin either through inhibition of platelet $P2Y_{12}$[40,52] or PAR1[53,54] receptors, can lower the risk of cardiovascular events in high-risk patient populations. In conclusion, aspirin has dose-dependent effects on platelet inhibition that go beyond platelet COX-1 inhibition and that have unknown clinical consequences.

Aspirin is also a weak inhibitor of COX-2, which is expressed in immune function and endothelial cells. Full suppression of COX-2 activity by aspirin has been estimated to require aspirin doses greater than 500 mg/day. Beyond COX-1/2 inhibition, it is increasingly clear that aspirin possesses several additional properties that do not appear to depend on COX inhibition. For example, aspirin (and its stable metabolite salicylate) are well-known to produce dose-dependent inhibition of elements of the proinflammatory nuclear factor κB (NFκB) pathway: NFκB,[55,56] IκB phosphorylation,[57] IκB kinase,[58] as well as other cellular kinases.[59,60] Aspirin also influences erythrocytes,[61] endothelial cells,[62] endothelial progenitor cells,[63] and inflammatory markers (c—reactive protein, interleukin-6, and macrophage colony stimulating factor).[64] In addition, omics-based assays are now illuminating the full suite of

pathways affected by aspirin, many of which are not predicted based on aspirin's putative mechanisms of action: acetylation of proteins beyond COX,[65] alterations in fatty acid and amino acid metabolism,[66] and changes in platelet RNA/protein content.[67] Although it is unknown to what extent these apparent non-COX effects of aspirin are dose dependent, it is clear that aspirin interacts with a wide array of diverse biologic pathways that may underlie its beneficial effects on cardiovascular disease.

In contrast, aspirin may also interact with prothrombotic pathways. In addition to serving as a precursor to TXA2, PGH2 produced by COX1/2 can be metabolized by vascular endothelial cells to produce prostacyclin (PGI_2). Although TXA_2 promotes platelet aggregation and vasoconstriction, PGI_2 inhibits platelet aggregation and induces vasodilation. Aspirin causes dose-dependent reductions in TXA2 and PGI2 production,[68] thus producing a blend of anti- and prothrombotic effects. No studies to date have directly established that more profound suppression of PGI_2 formation by higher doses of aspirin is sufficient to initiate or to predispose to thrombosis. However, recent studies in healthy volunteers exposed to aspirin 325 mg/day demonstrate that in certain individuals such dosing can lead to a paradoxical *increase* in platelet aggregation despite complete suppression of platelet COX-1.[69] Further, recent studies have associated selective COX-2 inhibitors with unopposed platelet activation and a greater likelihood for thrombotic events.[70] These findings provide mechanistic support for the efficacy of lower aspirin doses in preventing COX-1-mediated TXA_2 production while preserving the antiaggregatory and vasodilatory effects of PGI2. Whether higher aspirin doses will achieve a more beneficial (or potentially harmful) balance of TXA2 vs PGI2 in preventing cardiovascular events is unknown.

II.D.2. Aspirin Intolerance and Dose-Related Risks of Aspirin Therapy

In patients with established ASCVD, the risk associated with aspirin use is quite low compared with the benefit. Only a very small number of patients develop a serious anaphylactic reaction or bronchoconstriction with nasal polyps when they take aspirin.[71,72] The risk of intracranial hemorrhage with chronic aspirin treatment is estimated to be less than 0.04% per year.[73] A modest number of patients develop serious GI bleeding with chronic aspirin therapy, mostly related to the loss of the protective effect of prostaglandins on the gastric mucosa. A larger number of patients have GI intolerance with aspirin, but this is often transient and can be overcome in many patients. The absolute risk varies as a function of the trial entry criteria, but none of these major risks exceeds 5 events per 100 patients treated per year.[74,75] Except for patients with pre-existing asthma, no studies have described the risk factors for these complications of aspirin use.

The CAPRIE,[16] Coumadin Aspirin Reinfarction Study (CARS),[76] and Sibrafiban versus aspirin to Yield Maximum Protection from ischemic Heart events post acute cOroNary sYndromes (SYMPHONY)[77] trials have provided excellent opportunities to understand the incidence of aspirin intolerance and toxicity in patients with vascular disease. Among patients randomized to aspirin therapy alone in CARS, 2.7% discontinued the drug permanently, and 7.6% had a dose reduction. In SYMPHONY, which compared aspirin 80 mg twice daily with high and low doses of the oral glycoprotein IIb/IIIa inhibitor sibrafiban, the rate of early aspirin discontinuation was 19.2%, although over half of these patients later reported open-label use. In CAPRIE, 11% of patients randomized to aspirin therapy had to stop the drug because of

rash, diarrhea, indigestion, minor or major bleeding, or abnormal liver-function tests (**Table 3**).[16] Much higher proportions of patients had minor AEs that did not lead to discontinuation.

Table 3. Adverse Events with Antiplatelet Therapy in the CAPRIE Trial[16]

	Patients Ever Reporting		Severe		Requiring Discontinuation	
	Clopidogrel	Aspirin	Clopidogrel	Aspirin	Clopidogrel	Aspirin
Rash	578 (6.0)	442 (4.6)*	25 (0.26)	10 (0.1)*	86 (0.90)	39 (0.41)*
Diarrhea	428 (4.5)	322 (3.4)*	22 (0.23)	11 (0.1)	40 (0.42)	26 (0.27)
Indigestion/N/V	1441 (15)	1686 (18)*	93 (0.97)	118 (1.2)	182 (1.9)	231 (2.4)*
Any bleeding disorder	890 (9.3)	890 (9.3)	132 (1.38)	149 (1.6)	115 (1.20)	131 (1.4)
Intracranial hemorrhage	34 (0.35)	47 (0.49)	30 (0.31)	41 (0.43)	20 (0.21)	32 (0.33)
GI hemorrhage	191 (2.0)	255 (2.7)*	47 (0.49)	68 (0.71)*	50 (0.52)	89 (0.93)*
Abnormal liver function	285 (3.0)	302 (3.2)*	11 (0.11)	9 (0.09)	22 (0.23)	28 (0.29)

GI, gastrointestinal; N/V = nausea/vomiting
* $P < 0.05$. Data are number (%) of patients..

Although the dose-dependent COX inhibition by aspirin suggests that GI bleeding can be reduced with lower aspirin doses, no large, prospective randomized study has examined the relationship between lower doses of aspirin and bleeding. The available evidence is equivocal, and much of the available evidence is from an era in which positive reporting bias was common, especially in observational studies.

Despite the limitations of the available literature, a dose-response relationship has been suggested with regard to GI side effects, including discomfort and bleeding.[74,75] The lower risk associated with lower doses is believed to reflect both the differential inhibition of COX-1 on platelets and the differential inhibition of COX-2 in the gastric mucosa. In a case-control study, the OR for hospitalization for a bleeding peptic ulcer was 2.3 with 75 mg of aspirin, 3.2 with 150 mg of aspirin, and 3.9 with 300 mg of aspirin.[74] In another study, higher doses (300–1200 mg/day) showed a consistent dose-response relationship between GI bleeding and a need for hospitalization.[78] Conversely, a systematic review that included data from 66,000 patients reported no difference in the incidence of GI hemorrhage with doses less than 163 mg/day vs higher doses.[79] This study did not examine the risk of bleeding with doses less than 100 mg/day relative to higher doses, which may be important, because prostaglandin synthesis is inhibited with doses greater than 100 mg.

There is no reliable estimate of the risk of fatal GI bleeding as a function of aspirin doses below 162mg/day. A retrospective analysis of antiplatelet therapy in the CURE trial implies lower risks of both life-threatening bleeding and overall bleeding with lower aspirin doses **(see Section II.C.3).** The risk of death, in the presence of clinically detectable bleeding from a gastric ulcer, is estimated to be 0.5% to 10%.[80] Considering the available evidence, aspirin doses less than 100 mg/day may well be associated with less GI bleeding, which may in turn translate into a decrease in fatal events.

A large systematic review in 2007[81] found no evidence that doses higher than 81 to 100 mg/day were better for ischemic events and lower doses seemed to be associated with less bleeding. However, despite the millions of people taking aspirin daily, fewer than 5,000 participants in randomized trials could be aggregated, leaving the analysis dependent on extrapolations and observational studies.

In summary, more than 16 million Americans have ASCVD, contributing to substantial morbidity, mortality, and costs.[10] Based on the side-effect profile reported in trials of aspirin therapy,[16,76] 3% of these patients would be expected to have a total of 319,000 episodes of GI bleeding at an aspirin dose of 325 mg/day. Even assuming a conservative 10% reduction in GI bleeding with optimal dosing of aspirin, about 32,000 such bleeding events and 3,200 deaths from GI hemorrhage[80] would be prevented annually in the US alone. Accordingly, there is a clear need for an adequately powered randomized controlled trial to define the optimal dose of aspirin in order to minimize bleeding risks while preventing ischemic events.

II.E. Modifiers of Aspirin Dose

II.E.1. Aspirin, Minorities, and Other Subgroups

Early trials with aspirin were performed almost exclusively in men, leading to the empirical observation that the significant reductions in events were limited to men. Further study showed this simply was a problem of inadequate power to detect an effect in women, because very few women had been enrolled in trials. In a large prospective cohort study of 28,678 nurses taking 1 to 6 aspirins (dosage unknown) per week, the age-adjusted RR of a first MI was 0.68 (P = 0.005) with a trend toward fewer cardiovascular deaths (RR, 0.89; P = 0.56).[82] Still, without randomized trials enrolling representative numbers of men and women, it remains unknown whether aspirin has the same effect in men and women or if it shows a similar relation between dose and outcome.

In addition, little is known about how the effect of aspirin is modulated by age, race and ethnic background, the presence of diabetes, or renal function. Because ASCVD is the leading cause of death in women, racial minorities, diabetic patients, and patients with CKD in the US,[10] and because its prevalence and associated AEs increase proportionately with age, it is critically important to perform randomized trials of aspirin use in these populations. Patients with diabetes constitute a particularly important subgroup, given the considerable evidence that they may exhibit resistance to the antiplatelet effects of aspirin.

II.E.2. Enteric Coating

Enteric coating of aspirin became popular as a proposed approach to reducing aspirin's GI toxicity. While some small studies have shown promising results regarding the benefit of enteric coating, no large outcomes trials have evaluated the benefit-risk balance of enteric-coated aspirin vs aspirin without enteric coating. Additionally, pharmacokinetic/pharmacodynamic studies have raised questions about the reliability of absorption of aspirin when given in enteric-coated form.[83,84] Given these uncertainties, we will collect details of the actual dose and type (enteric-coated vs nonenteric-coated) of aspirin taken

by trial participants at regular study intervals, but the randomization to low- vs high-dose aspirin will not specify whether the patient should take enteric-coated aspirin.

III. Research Design and Methods

In this secondary-prevention trial in patients with ASCVD, the effectiveness of 2 once-daily doses of aspirin (81 mg and 325 mg) will be compared. The trial will use a novel format that exploits EHR data that have been standardized according to a common format and primarily web-based systems of communication among enrolled patients and trial investigators with the support of health systems interested in the best care for their patients. Every aspect of the trial is designed to not only answer the research question, but also to build an infrastructure for future pragmatic trials in which a community of patients, clinicians, researchers, and administrators work together to improve patient care and clinical outcomes.

III.A. Recruitment

The majority of the PCORnet's CDRNs, an HPRN, and 1 PPRN have agreed to participate in this trial. After obtaining IRB approval of the trial and with agreement of the clinicians and their health systems, the EHRs at the participating health systems in the CDRNs and the HPRN will be queried for eligible study participants. This will be supplemented with local engagement of health systems, clinicians, and patients, as well as direct recruitment in clinics and hospitals. The trial is designed not to interfere with routine clinical practice and is expected to impose a minimal burden on clinicians, clinics, health systems, and patients.

III.A.1. Cohort Identification

Local site investigators within the CDRNs will be asked to endorse the protocol. They will then be asked to give their permission for the CDRN, through its integrated health system members, to identify and contact potentially eligible patients. In the latter case, patients who meet criteria for trial enrollment will be identified using search algorithms developed by the DCRI Coordinating Center (based on the trial inclusion criteria) and customized by the CDRN and HPRN for their own EHR systems. Broad agreement from both cardiovascular specialists and primary care physicians will be sought to ensure widespread clinician engagement. In this trial, it is believed that most systems will agree that prior approval of the relevant clinician will be needed and useful since these patients will be at high risk for death or a major disabling event. Although it is unlikely that a medical reason for ineligibility will be found, most of these patients are close to their clinicians, whose confidence in and support of the trial will be important for patient engagement, both in terms of participation as well as promoting adherence to the study medication and treatment of the inevitable clinical events.

III.A.2. Women, Elderly, and Minority Participants

The inclusion of women and minorities in clinical trials is desirable for scientific, ethical, and social reasons. This trial's investigators feel strongly that the inclusion of women, the elderly, and racial

minorities is imperative to meet the goal of developing a therapy that is ultimately generalizable to the global ASCVD population. The increasing number of men and women 65 years of age or greater with ischemic heart disease also mandates that they be represented in a dosing trial evaluating the safety and effectiveness of aspirin. Although octogenarians represent only 5% of the US population, they represent 20% of all patients hospitalized for MI and 30% of all MI-related hospital deaths.[85]

PCORnet is committed to promoting equity in research, and PCORI has made concerted efforts to include underrepresented populations in research. Because this trial is intended to identify an optimal aspirin dose that is applicable to a large population of ASCVD patients, our aim is to enroll a study population whose demographic profile is similar to the representation of women, elderly persons, and minorities in the overall population of individuals with ASCVD in the US.[19,85] Estimates of the incidence of ASCVD in American women have ranged from 30%[86] to 51%[85] and vary considerably relative to age group.[86] Although the elderly (>75 years) account for less than 6% of the population,[58] the incidence of ASCVD among elderly patients is proportionally higher compared with younger age groups (169 vs 60 per 1,000 persons).[87] Based on these demographics, we will aim for 30% to 40% of our enrolled patients to be women and at least 10% to be elderly (≥ 75 years). Because the incidence of ASCVD is similar among ethnic groups in the US (~7% for each group of non-Hispanic whites, non-Hispanic blacks, and Hispanics),[85] we have selected to strive for enrollment target of 20% to 25% for minorities, to reflect their representation in the general population (e.g., 12.1% non-Hispanic blacks; 12.3% Hispanics).[58]

The CDRNs will develop local recruitment strategies specifically designed to facilitate the inclusion of a broadly representative population of patients that incorporates women, the elderly, and racial/ethnic minorities. For example, many participating sites are regional centers located in areas with higher numbers of minorities; this will enhance minority recruitment.

III.A.3. Sources of Potential Bias

Because this trial emphasizes the use of the internet and standardized EHR data, we acknowledge potential barriers to participation, including older age, low socioeconomic status, and low literacy. However, internet use will facilitate the conduct of this pragmatic trial by reducing potential limitations to enrollment, including the costs associated with frequent follow-up visits at healthcare facilities. We will encourage enrollment of patients with personal internet access as well as patients with public internet access only, such as those available through a library, enrolling physician's office, or workplace. Additionally, the ADAPTABLE patient portal will be accessible via tablet computers and smart phones so patients with access to the internet only through cellular service will be able to participate in this manner.

No studies have formally evaluated the association of internet use with general health status and outcomes. Internet users may derive indirect benefits that improve their health status (e.g., disease recognition, symptom awareness, risk-factor modification), and/or they may be better educated, more motivated in personal health maintenance, and wealthier, all of which may be associated with better health status and outcomes in secondary prevention. Although the investigators acknowledge these

possibilities, we believe the results of this trial remain applicable to all patients with ASCVD for 3 reasons.

First, since the occurrence of cardiovascular events is multifactorial, standard risk factors (e.g., smoking, diabetes, and age) are more likely to directly influence cardiovascular event rates than internet use. Second, aspirin use has substantial consequences, effecting an approximately 25% reduction in adverse cardiac events. Rather than modify the benefit of aspirin therapy, internet use would more likely be associated with increased awareness of the general need for aspirin therapy, which also is an educational objective of the trial patient portal. Third, the aim of this trial is to define the optimal dose of aspirin in patients with ASCVD. Even if internet use were associated with improved health, this effect would be a general reduction in AEs across all treatment groups. Although attributing a global improvement in outcomes in this trial to internet use may be hypothesis generating, such a finding still would not influence the primary objective of the trial.

Another source of bias may exist with respect to aspirin therapy prior to randomizations. We expect that most patients recruited into the ADAPTABLE trial will already be treated with long-term aspirin therapy, given the nature of the inclusion criteria and the known high utilization of aspirin for the secondary prevention of coronary artery disease in the US. However, we will add a sensitivity analysis with a 10-day landmark to the statistical analysis plan (SAP) that excludes all events occurring in the first 10 days following randomization, in order to formally test for a legacy effect of the aspirin dose taken before randomization.

III.A.4. Patient Engagement

The ADAPTABLE trial will address several key questions mentioned in PCORI's definition of patient-centered outcomes research. This trial is designed to help individuals answer the following questions: "Given my personal characteristics, conditions, and preferences, what should I expect will happen to me?" "What are my options and what are the potential benefits and harms of those options?" and "What can I do to improve the outcomes that are most important to me?”

The study seeks to make more relevant, detailed information regarding aspirin therapy as secondary prevention for ASCVD available to patients and their caregivers by answering question such as “What is the most effective dose of aspirin in secondary prevention of ASCVD?” and “What are the bleeding risks associated with taking 81 mg or 325 mg of aspirin?” Of the 40 topics suggested for the first PCORnet study, 6 were selected for consideration through the PCORI Advisory Panel Prioritization process by a body consisting of patients, scientists, and other stakeholders charged with prioritizing CER topics for PCORI. ADAPTABLE was chosen to be the first PCORnet study based on its importance to patients, clinicians, and scientific communities.

Patient and stakeholder engagement has been a priority to the study team since the inception of ADAPTABLE. A partnership was formed with the Health eHeart Alliance Patient-Powered Research Network to support ADAPTABLE by leading the CDRN patient representative team called the Adaptors. Additionally, 2 seasoned cardiology patient advocates served on the protocol design committee to assist

with defining essential characteristics of the study, participants, and outcomes. These advocates also worked actively with the study team and Health eHeart to develop the patient-engagement plan.

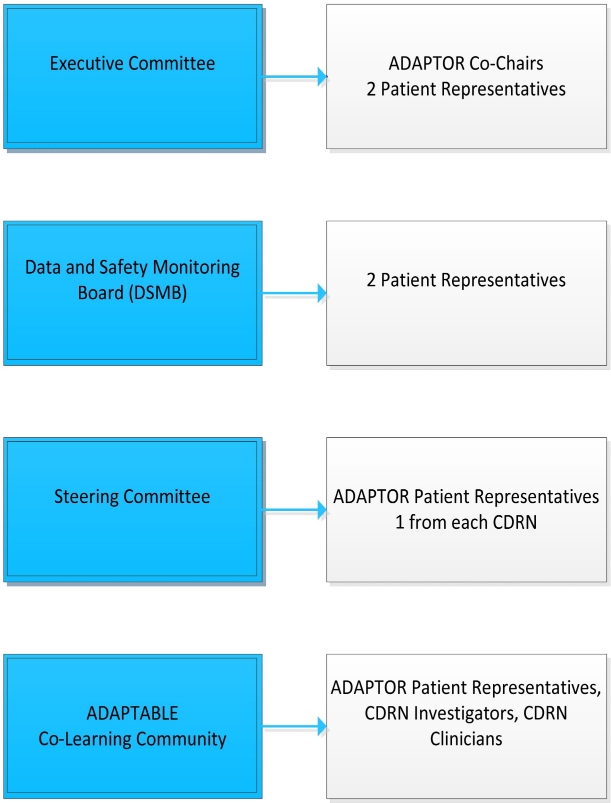

The DCRI Coordinating Center for ADAPTABLE will convene the Executive Committee, Steering Committee, and Data and Safety Monitoring Board (DSMB)—each of which will have at least 2 patient representatives, while Health eHeart will support and facilitate the Patient Group, herein referred to as "Adaptors." The Adaptors group will include 1 patient representative from each participating CDRN. CDRNs will seek to identify patients who represent the trial's population (see **Appendix: Patient Survey for ADAPTOR Participants**), and they may select skilled cardiology patient representatives who use aspirin if they are unable to locate a patient representative with appropriately matched characteristics and skills. Diversity and inclusiveness are highly valued and encouraged. We will ensure that the patient representatives will be recruited from the population they are serving by using the Adaptors Screening Tool (see attachment provided with this document). The screening tool will be circulated by Health eHeart and partnering CDRNs to identify potential patient representatives for the Adaptors group. The Adaptors will serve a dual role as designers and advisors; they will work in concert with the study team to help design study materials, the trial consent form, and recruitment plans through their work with Health eHeart. They will also be members of the Steering Committee, where they will monitor study conduct and progress. The co-chairs of the Adaptors group, who will be selected by the patient representatives and confirmed by Health eHeart and the DCRI Coordinating Center to be able to carry out the associated duties, will also serve as the patient representatives on the Executive Committee to provide study oversight and promote cross-pollination of ideas and sharing of information between the Executive Committee, Steering Committee, and the Adaptors Group. Also, 2 cardiology patient advocates (not a part of the Adaptors) will serve on the DMSB to participate in review of study data sets and AE monitoring and reporting

III.B. Enrollment and Eligibility

Pragmatic enrollment criteria have been selected to achieve the most generalizable sample possible to address the main study hypothesis and to reflect how patients with ASCVD are characterized in routine clinical practice. The protocol was posted for public review and comment in July, 2015, and specific questions regarding key eligibility criteria were voted upon. Approximately 57% of the survey respondents (with 7% expressing no opinion) voted to not limit the inclusion criteria to only include patients already taking aspirin at the time of randomization, 49% (with 14% expressing no opinion) voted to allow patients taking ticagrelor at the time of screening to be included in the trial, and 49%

(with 6% expressing no opinion) voted to exclude patients with a clear indication for an oral anticoagulant even if they are not current taking an oral anticoagulant. The final inclusion/exclusion criteria listed below were adapted based upon these survey responses. However, based upon feedback from the CDRNs regarding their local IRB's perspective on the "minimal risk" of the informed consent process, it was decided to exclude patients treated with ticagrelor, given the "black box" warning in the ticagrelor label in the US for avoiding high-dose aspirin with concomitant ticagrelor use.

1. Known ASCVD, defined as ANY of the following:
    a. Prior MI
    b. Prior coronary revascularization procedures (either prior PCI or prior CABG)
    c. Prior coronary angiography showing 75% or greater stenosis of at least 1 epicardial coronary vessel
    d. History of chronic ischemic heart disease, coronary artery disease, or atherosclerotic cardiovascular disease
2. Age 18 years or greater
3. No known safety concerns or side effects considered to be related to aspirin, including
    a. No history of significant allergy to aspirin, such as anaphylaxis, urticaria, or significant GI intolerances
    b. No history of significant GI bleed within the past 12 months
    c. Significant bleeding disorders that preclude the use of aspirin
4. Not currently treated with an oral anticoagulant—either warfarin or a novel anticoagulant (dabigatran, rivaroxaban, apixaban, edoxaban)—and not planned to be treated in the future with an oral anticoagulant for existing indications such as atrial fibrillation, deep venous thrombosis, or pulmonary embolism.
5. Not currently treated with ticagrelor and not planned to be treated in the future with ticagrelor.
6. Female patients who are not pregnant or nursing an infant.
7. Estimated increased risk of MACE over next 3 years, as defined by the presence of at least 1 or more of the following enrichment factors:
    a. Age 65 years or greater
    b. Serum creatinine 1.5 mg/dL or greater
    c. Diabetes mellitus (type 1 or type 2)
    d. Current cigarette smoking
    e. Cerebrovascular disease
    f. Peripheral arterial disease
    g. 3-vessel coronary artery disease
    h. Systolic or diastolic heart failure
    i. Left ventricular ejection fraction less than 50%
    j. Systolic blood pressure 140 mm Hg or greater, documented within the prior 12 months

k. Low-density lipoprotein cholesterol 130 mg/dL or greater, documented within the prior 12 months

There will be no exclusions for any upper age limit, comorbid conditions, or concomitant medications other than oral anticoagulants and ticagrelor that are used at the time of randomization or are planned to be used during the study follow-up.

Simple, inclusive, pragmatic eligibility criteria will make enrollment easier and will render study results more generalizable to a broader population of patients. We will exclude pregnant or lactating women (because of concern for the fetus or child), patients taking oral anticoagulants or likely to require an oral anticoagulant during trial follow-up (because of complex drug interactions and a projected excessive risk of bleeding), and patients at relatively low risk for cardiovascular events (i.e., no enrichment factor because of the large number of outcomes needed to detect a clinically meaningful difference with the available sample size).

III.C. Study Design and Procedures

III.C.1. General Timeline

To meet the goal of enrolling 15,000 patients, we have enlisted the recruiting CDRNs and HPRN (HealthCore/Anthem Research Network) and patient advocates to mobilize the requisite number of clinics, health-system based populations, and private health plan populations to meet the trial-recruitment goals. The anticipated study duration is approximately 4 years.

III.C.2. Consent, Randomization, and Drug Allocation

Patients who are identified as candidates for the trial will be directed to the ADAPTABLE patient portal for the eConsent as well as an abbreviated eligibility confirmation prior to randomization. The ADAPTABLE patient portal will include the electronic patient consent (eConsent) that satisfies the local IRB and state law requirements of the participating CDRNs and HRPN. CDRN personnel will be available, if needed, to give a more local perspective on protocol-related consultations. For patients without internet access, patient consent via the ADAPTABLE patient portal will be obtained during a process facilitated by site research staff during clinic encounters, and follow-up will be done via telephone contact with the DCRI Call Center.

Patients will be randomized via the patient portal in a 1:1 ratio to receive 81 mg vs 325 mg of aspirin. Patients will be asked to obtain their randomized aspirin dose at their local pharmacies. The randomization scheme will be established before the inception of enrollment. In a trial of this size, stratified randomization has no significant advantages.

After randomization, during the early first web portal contact or telephone contact (for patients without internet access) from 1 to 3 weeks after randomization, patients will be asked to answer questions related to current aspirin use and dose and other specific concomitant medications. They will also be asked to provide contact information such as their name, email and home address, phone numbers, and

contact information for a family member or friend not living with them. These data will be used to contact patients who miss multiple visits and to identify events such as hospitalizations that occur out of network. The enrolling sites will be responsible for ensuring that randomized patients complete their early first contact visit (1-3 weeks after randomization).

Neither patients nor healthcare providers will be blinded to their treatment assignment, as blinding would add substantial complexity and cost without commensurate incremental benefit. The “hard” outcomes, large sample size, and equipoise in the clinical community should enable valid results to be obtained, and we have no reason to believe that investigator or clinician bias will play a role in ascertainment or classification of events.

III.C.3. Concomitant Therapy

Key concomitant medications will be self-reported by the participant and over time will also be harvested from the PCORnet DataMart in CDM version 3.0 as it is deployed. Pre-randomization aspirin dose for aspirin users will be recorded. Participants will report key concomitant medications annually, either on the participant portal or during phone follow-up calls from the DCRI Call Center.

Details on the use of aspirin and important concomitant medications ($P2Y_{12}$ inhibitors) and other over-the-counter medications of interest (nonsteroidal antiinflammatory drugs [NSAIDS] and proton-pump inhibitors [PPIs]) will be collected from participants at baseline and annually during study follow-up. Details on other common secondary prevention medications prescribed (beta-blockers, angiotensin-converting enzyme inhibitors/angiotensin receptor blockers, and statins) will be collected from the CDM. The large trial population and randomization will minimize potential biases and confounding that could result from differential use of concomitant medications by randomized treatment assignment. A sensitivity analysis that accounts for concomitant medications as time-dependent covariates will be done to account for post-randomization treatments.

III.C.4. Schedule of Assessments

ADAPTABLE SCHEDULE OF ASSESSMENTS*

	Baseline	1-3 Week Follow-up Post-Randomization	Every 3 or 6 Month Follow-up	Annual Collection	End of Study
			*Assessments collected every three or six months based on randomization	Replaces 3M /6M follow up	
Video describing trial expectations	X				
Check Key Eligibility Review	X				
Sign e-Consent	X				
Randomization and dose assignment	X				
Collect pre-randomization aspirin dose		X			
Collect demographic/contact information	X	X			
Collect key concomitant (Rx and OTC) medications		X		X	X
Collect current aspirin dose/reason for DC		X	X	X	X
Collect hospitalizations		X	X	X	X
Collect Patient Reported Outcomes (PRO)	X		**Q6 months**		X
Insurance info/Confirmation at Contact info		X			

*All Baseline and 1-3 Week Follow-up Post Randomization visit data will be collected at the Baseline visit for all non-internet patients with the exception of the current aspirin dose. The current aspirin dose will be collected 1-3 weeks following randomization.

III.C.5. Data Collection and Follow-up

For all participants and during follow-up, demographics and key medical history, cardiovascular risk factors, and certain current medications will be obtained from the CDM. An early check-in web portal or telephone (for participants without internet access) contact (1-3 weeks after randomization) will be done for all participants to collect key information, as detailed in the preceding Schedule of Assessments. The enrolling site will be responsible for ensuring that randomized participants complete their early first web portal or telephone contact (1-3 weeks after randomization). Thereafter, participants will be randomly assigned to follow-up electronic or telephone contacts every 3 vs 6 months. During follow-up contacts, data on aspirin dose and use and hospitalizations will be collected. By embedding a randomization for follow-up every 3 months vs every 6 months for participants, the best method for optimizing participant follow-up and data collection in this pragmatic trial will be investigated. For the cohort of participants without internet access included, they will be contacted by the DCRI call center via telephone for the early check-in visit and follow-up contacts based upon their randomized frequency of follow-up, just as the participants with internet access will be followed with electronic contacts.

As envisioned by PCORnet, the PCORnet distributed research network (DRN) is to be a "…functional distributed research network that facilitates multi-site patient–centered research across the CDRNs, the PPRN, the HPRN, and other interested contributors. The distributed network enables the conduct of observational research and clinical trials while allowing each participating organization to maintain physical and operational control over its data."[88]

In PCORnet's distributed data environment, code is developed centrally and distributed to each partner to execute against data that are stored in a common format. Code ("queries") are distributed and results are returned via PopMedNet, a networking software application that manages the creation, operation, and governance of distributed health data networks.

The PCORnet CDM is the foundation of the PCORnet DRN. The PCORnet CDM is being implemented in phases to allow for the incorporation of new data domains and fields based on PCORnet needs, lessons learned from use, and data availability.

The PCORnet CDM Versions 3.0 and 3.1 include 10 tables that represent specific data domains that are available in EHRs and directly from patients. **Table 4** describes data categories that are applicable to the ADAPTABLE CRF.

Table 4. Data Categories Applicable to the ADAPTABLE CRF		
	Description	Applicability for ADAPTABLE Data Collection
Demographic	Contains 1 record per participant.	Birth date, sex, Hispanic ethnicity (y/n) & race captured.
Encounter	Contains 1 record for each time a participant sees a provider in ambulatory setting or is hospitalized; multiple encounters/day possible if they occur with different providers or in different care settings.	Encounter type will be used to identify hospitalizations during follow-up period. Deaths occurring during a hospital stay will be captured in the discharge disposition variable.
Diagnosis	Contains all uniquely recorded diagnoses for all encounters. Each diagnosis is associated with a specific participant & encounter.	Diagnosis codes and associated encounter dates will be used to establish medical history prior to randomization.
Procedure	Contains all uniquely recorded procedures for all encounters. Each procedure is associated with a specific participant & encounter.	Procedure codes and associated encounter dates will be used to establish catheterization, PCI, and CABG prior to randomization.
Vital	Contains 1 record per result/entry. Multiple measurements per encounter are recorded as separate measures.	Height and weight are captured; tobacco status has been added to v2.0.
Lab Result	Contains 1 record per result/entry.	Creatinine, hemoglobin, and LDL-C will be included in v2.0.

LDL-C, low-density-lipoprotein cholesterol

In essence, except for aspirin dose and use of over-the-counter NSAIDS, H_2 blockers and PPIs, all of the essential data for the trial are included in CDM 3.0. A unique attribute of this trial is the continuous improvement expected in the extent and complexity of the available data as the trial goes on.

The CDM contains some of the 18 elements that define protected health information (PHI)under HIPAA (Health Insurance Portability and Accountability Act), including encounter dates and date of birth. The necessary "cross-walks" between the arbitrary identifiers included in the CDM and their originating data are not specified in the scope of the CDM, but are expected to be maintained by each CDRN.

- PATID is a pseudoidentifier with a consistent crosswalk to the true identifier retained by the source site. For analytical data sets requiring participant-level data, only the pseudoidentifier is used to link across all information belonging to a participant.
- Locally maintained "mapping tables" are tables necessary to implement so that each CDRN/partner has the ability to map arbitrary identifiers back to the originating data and participant.

The CDM will be supplemented by the study participant portal datasets, as well as Medicare fee-for-service claims, datasets held by large national health plans (with appropriate authorizations from enrolled participants and agreements from local datamarts to transmit participant PHI to the health plans for future data queries from the coordinating center) and the Medicare Beneficiary Summary File (with death dates provided by the Social Security Administration). Further details on the process and authorizations for Medicare and health plan data linkages are contained in a separate Linkage Plan document. In order to link to these data sources, participants will be asked to voluntarily provide a portion of their social security number and all of their health plan identification numbers via the secure participant portal when they enroll in the trial. If the data are not provided by the participant, sites may be asked to provide these data, if available. Provision of this PHI is entirely voluntary; the data are not

required to participate in the trial. The principles of minimum necessary and compartmentalization of identifiable data will be adopted; data will be stored via highly secure practices as described below.

III.C.5.a. Minimizing Cross-Overs

Because this is an open-label trial in which participants and their healthcare providers will know their randomized dose of aspirin, the CDRN will be asked to engage regularly with the healthcare providers in their network regarding the rationale for the trial and the importance of compliance with the randomized treatment assignment to minimize patient cross-over. These clinical engagement approaches will be designed to facilitate discussions with healthcare providers who will be asked not to change a participant's aspirin dose during the trial unless it is absolutely necessary to ensure participant safety. On the ADAPTABLE participant portal, participant will be reminded of the importance of adhering to the aspirin dose they were randomly assigned to unless their physician changes their dose for a major safety concern. Participant who cross over will be analyzed as they were originally randomized according to the intention-to-treat (ITT) principle.

III.C.5.b. Drug Discontinuation – Monitoring and Recommendations

Aspirin use will be collected via the responses to the participant questions during follow-up. During each follow-up contact, compliance with the randomized aspirin dose will be assessed. The occurrence of and reasons for discontinuing aspirin or changing the dose of aspirin will be ascertained. In the event of aspirin intolerance or bleeding, the decision to continue the randomized aspirin dose, convert to a lower or higher aspirin dose, or to discontinue aspirin therapy will be left to the treating physician's discretion. Participants who discontinue aspirin therapy altogether will remain in the trial for clinical outcomes follow-up according to the ITT principle.

If a female participant becomes pregnant or plans to attempt to become pregnant during study follow-up, then aspirin should be permanently discontinued. Furthermore, if a participant requires an oral anticoagulant during study follow-up, the treating physician should carefully consider whether to continue aspirin based upon the estimated bleeding risks of aspirin used together with an oral anticoagulant, based on the clinical context. Finally, participants who develop an intracranial hemorrhage or a life-threatening bleeding event during study follow-up should be considered for permanent discontinuation of aspirin, at the discretion of their treating physician.

III.C.5.c. Delinquent or Missing Follow-up

Data from participants who fail to respond to questionnaires via the ADAPTABLE participant portal or via telephone contact with the DCRI Call Center after at least 2 separate contact time points will be collected using all available approaches, including but not limited to the DCRI Call Center, participant finder companies, the internet, EHR data stored in the CDM format in the PCORnet DataMart, and other additional search methods. Additionally, if these methods fail, the site will be contacted to determine whether they have been in contact with the participant.

III.C.6. Developing and Refining the PCORnet Infrastructure

Based on previous experience with clinical trials and participant follow-up (see **Section III.C.5.**), there is broad familiarity with the potential limitations of EHR data and patient-reported clinical outcomes. As a result, PCORnet is in a position to identify and resolve inaccuracies to improve the validity of such data. Because this trial will investigate a clinically relevant issue in a new format, the study will also examine the potential for conducting trials through health systems with secondary data use and internet-based follow-up with both participants and clinician-investigators. Because this trial will rely partly on patient-reported data gathered via the internet, it will determine the feasibility of participant enrollment and reporting via the internet. As previously stated, Aim 3 of this project will focus upon using defined metrics to evaluate the PCORnet infrastructure that will be developed across PCORnet studies.

III.C.7. ADAPTABLE Patient Portal

Participant data will be captured via the ADAPTABLE participant portal that will be designed and maintained by Medidata. Screens will be user friendly and will contain questions that are easy to understand and answer. Content for the participant portal will be in English and Spanish to facilitate bilingual interactions. Based on previous studies of internet usability for people over 65 years,[89] the study participant portal will be designed to accommodate issues such as impaired vision (e.g., font size and "readability"), memory (e.g., focused error messages and hypertext links), and motor control and precision of movement (e.g., "clickability"). Screens, checkboxes, or pull-down menus will be used when possible for data entry to minimize the need to enter free text.

Queries will be programmed directly into the study participant portal and will prompt participants for missing or discrepant data. For example, if a participant skips a question, a program warning will occur that directs the participant to complete key questions before continuing. Similarly, program warnings will alert participants if out-of-range data have been detected. This method of on-the-spot querying will ensure cleaner data.

The participant portal will contain a general information section that allows access by study participants and general and medical lay communities. There will also be restricted sections on the participant portal that are accessible only to the trial's investigators. The restricted section for investigators will contain more specific information about their local participation. Visibility of the link to the restricted section will be determined by the access rights.

Below is a partial list of items that could be shared:

- *Training materials,* to include resources for training in ethics of human research, recordings of investigator meetings/conference calls, and other appropriate training materials;
- *Educational links,* to provide additional education materials for participants (e.g., the American Heart Association website) and providers (e.g., theheart.org; the American College of Cardiology website);
- A *protocol* section, to include the current version of the protocol. Eligibility criteria will be listed, as will instructions for completing follow-up questionnaires through the participant portal;

- Regular communications, to include enrollment data, frequently asked questions), and other trial information; and/or
- *Enrollment information* to be posted on the general area of the site.

III.D. Data Security and Back-up Procedures

III.D.1. Medidata Data Security for the **Participant** Portal

The system is deployed using a LAMP (Linux, Apache, MySQL, PHP) technology stack running on a virtual machine (VM) in a private cloud. By using a virtualized environment, additional resources (disk space, processing power) can be added to the VM as necessary to support increased load. Data centers used to host the system hold current SSAE16 SOC certification and have Tier 1 internet access from multiple major internet service providers for redundancy and 100% network uptime.

Access to the system's web-based user interface (UI) is provided using HTTPS (web protocol that is secured using 128-bit [or stronger] encryption). Each user must have a unique username and secret password to authenticate into the system's UI using a browser. All passwords, whether used to access the web UI or to access a server directly via the back end, are required to meet minimum requirements for strength, complexity, and aging. Subsequent responses by the server to requests sent from the user's browser are restricted and processed according to the user's assigned role (i.e., using role-based access control that is strictly enforced by the application logic).

Direct access to the VM that hosts the system is only possible via a virtual private network (VPN) and then only by senior technology staff who are documented on an access control list that is reviewed for accuracy at least quarterly. Direct access to the MySQL database is also only possible by using the VPN, and the MySQL database server is configured to disallow network connections and only to accept connections that originate from the same server ("localhost"). That is, it is not possible for a MySQL client to connect to the MySQL database over the network. The client must be on the same VM as the database server.

The firewalls that implement the VPN only allow connections to ports that are required for the system to function (i.e., to port 443 for HTTPS connections). All other attempted connections are rejected. This configuration ensures that an end-user can access only data stored in the database by first authenticating with and sending requests through the web server, which processes each request and returns results that are determined by the user's role.

The system leverages industry-standard techniques to maintain high levels of data security and integrity. User credentials are stored as salted hashes, and the study team does not have access to or the ability to recreate user passwords. Direct access to the study database is tightly controlled. Moreover, all changes to the database (create, modify, delete) are tracked using a 21 CFR § 11-compliant audit trail.

Security is further ensured by the use of an Alert Logic Intrusion Detection System (IDS) and an Alert Logic Log Manager, both of which monitor all servers on the network. The Alert Logic IDS inspects all inbound and outbound network traffic (i.e., data packets) that passes through the network (both from

the internet and within the private network). Moreover, the IDS generates regular reports of possible exploits, thereby ensuring that outdated server software is promptly detected and upgraded to more secure patch levels.

To ensure high availability, the system is configured with a primary server and an identical failover ("disaster recovery") server that is located at a geographically remote data center. If the primary site becomes unavailable, the failover site can be activated well below the 60-minute recovery time objective. The primary and failover database servers are also configured for replication, thereby ensuring that any transaction on the primary database also occurs on the failover database seconds later. As a further safeguard against data loss, the failover server exports the database to file every 30 minutes, and retains exports for 72 hours. In addition to the database exports, each server itself is backed up in full daily and weekly.

To ensure that the PHI of research participants remains confidential, any personally identifying information that is stored in the database is encrypted using 128-bit AES encryption algorithm. Moreover, the technology staff who maintain the systems are required to use personal computers that use password-protected access, screen locking, hard drive encryption, VPNs, and virus scanning utilities to ensure that their access to the study's servers cannot be compromised or misappropriated by unauthorized parties.

The data centers are monitored by certified network technicians and security personnel 24/7/365, and provide physical and logical security through a combination of keycard protocols, biometric scanning protocols, and around-the-clock interior and exterior surveillance. Access to the facilities is limited to authorized data center personnel—no one can enter the production area without prior clearance and appropriate escort. Every data center employee undergoes thorough background security checks. Conditioned power provides 100% availability through UPS (uninterruptible power supply) for all servers and N+1 redundant UPS power subsystem, with instantaneous failover if the primary UPS fails. If an extended utility power outage occurs, routinely tested, on-site diesel generators can run indefinitely. The facilities also offer a precision environment. Using an N+1 redundant HVAC (heating, ventilation, air conditioning) system ensures a duplicate system immediately comes online in the event of an HVAC system failure. Every 90 seconds, all air is circulated and filtered to remove dust and contaminants; in addition, the facilities also use advanced fire-suppression systems appropriate for a data-hosting environment. The facilities network topology and configuration was co-developed with Cisco and guards against single points of failure at the shared network level. Cisco and Arbor Networks work with the data centers to continually improve monitoring and security.

III.D.2. PopMedNet Data Security

Data will be transferred from the sites to the DCRI using PopMedNet.[90] PopMedNet consists of 2 layers: a security layer where access controls and permissions are established and an exchange layer through which questions and responses are passed. Each of the current implementations of PopMedNet™ is hosted in a Federal Information Security Management Act-compliant private cloud tier III data center. All

communications from the portal are encrypted. Selected current and in-process security features include:

- Strong passwords that expire every 6 months and may not be reused
- Automatic logoff after inactivity
- Automated query-results deletion
- Audit of all system activity
- DataMart administrators notified of new users
- Encrypted password storage
- Use of cryptographically secure random values for session IDs
- Secure distribution of DataMart Client software

III.D.3. Duke Clinical Research Institute Data Security

One major risk in participant-centered research is introduced by the necessity to use participant identifiers (e.g., a participant's name and phone number is needed in order to call them for follow-up) in data systems outside the enrolling site without compromising the confidentiality and security of data. The principles of minimum-necessary and compartmentalization of identifiable data will be adopted. Primary identifiers will be retained only within the data systems where required to conduct the study; these will be omitted as data travel downstream and only study IDs will be in the analysis datasets. Further de-identification and anonymization methods will be used when making data available for secondary analysis.

The DCRI, as part of the Duke Health HIPAA-covered entity, routinely operates at the intersection of handling PHI and the requirements of large-scale, multicenter research programs and data-sharing initiatives and is extremely prudent in keeping participant data secure and confidential. The primary computing platforms for enterprise systems are Sun Servers running the Solaris 8 operating system, a Unix-based operating system. All Oracle databases run on the Sun Servers, which are additionally attached to the HP Storage Works Storage Area Network. Client-server applications require network authentication. In addition, client-server applications may have their own internal security system and/or Relational Database Management System security. The DCRI's core back-end tools used on this project will be SAS Analytics (SAS Institute Inc.) for data integration and data analysis. As a HIPAA-covered entity and experienced research organization, we employ a variety of technical and standard-operating-system–driven approaches to ensure the security and confidentiality of all data.

Data will be transferred from the sites to the DCRI using PopMedNet. The DCRI will be responsible for maintaining the confidentiality and security of transferred data. The control of access to databases will be managed centrally by the Duke Health Technology Solutions (DHTS) through user passwords linked to appropriate access privileges. This protects data from unauthorized view and modifications as well as inadvertent loss or damage. Duke has an extensive data security infrastructure. Database servers are secured by a firewall as well as through controlled physical access. Oracle has many security protection features that ensure that each person accessing the database has the proper authority to perform the functions he or she requests of the data management system. Within the secondary SAS datasets, UNIX

group access control will be used for maintaining similar security. The Sun workstation log-in will be secured by extensive user password facilities under UNIX.

III.E. Endpoints and Adverse Events

III.E.1. Primary Endpoint

The primary effectiveness analysis will be performed on the entire randomized (ITT) population. The primary endpoint of this study is the composite rate of all-cause mortality, hospitalization for nonfatal MI, or hospitalization for nonfatal stroke. Traditional reporting of potential endpoints by study sites with independent adjudication by a Clinical Events Committee will not be done in this trial given the pragmatic nature of the ADAPTABLE study and the low-cost trial budget. We are planning to implement an endpoint validation plan to ensure the accuracy of endpoint classification compared with clinical endpoint adjudication processes used in traditional clinical trials. Once approved, the description of the validation plan will be provided in a separate document. Validated coding algorithms will be applied to a variety of EHR data sources to comprehensively ascertain potential endpoints related to hospitalizations for the nonfatal component primary endpoints (MI and stroke) and the secondary endpoints. A recently published study demonstrated that events ascertainment and classification using coding algorithms with administrative claims data yielded similar results compared with the standard adjudication of events done in traditional clinical trials.[91] The informed consent form will cover access to the data needed to support the validation plan.

III.E.2. Secondary Endpoints

Secondary endpoints include the components of the primary endpoint, coronary revascularization procedures (PCI and CABG), and quality of life and functional status.

III.E.3. Safety Endpoints

The major safety endpoint is hospitalization for major bleeding with an associated blood product transfusion.

III.E.4. Identifying Endpoints

Endpoints will be ascertained by applying algorithms designed to ensure comprehensive surveillance for all potential endpoints. To supplement the PCORnet CDM data, Medicare data and health plan data will be obtained at regular intervals during the course of the study, for a portion of the study participants. Further details on the process and authorizations for Medicare and health plan data linkages are contained in a separate Linkage Plan document.

- Routine queries will be applied to the PCORnet CDM, Medicare data, and health plan data to capture and classify nonfatal endpoints (hospitalization for MI, hospitalization for stroke, or hospitalization for major bleeding) using validated coding algorithms that will be applied to hospitalizations for each randomized participant. Because these electronic health data sources are the source of truth for the trial, no patient confirmation or other additional confirmation will be required for hospitalizations verified by the coding algorithms applied to these data sources (see figure below). Medicare data and health plan data are being queried to help identify endpoints that occur outside of the recruiting sites (“out-of-network” endpoints). Because we do not expect these other data sources to fully capture all out-of-network endpoints, we are

also asking patients to report all hospitalizations that occur during the follow-up period via the ADAPTABLE participant web portal.

- Medical record data for hospitalizations reported by participant on the ADAPTABLE participant web portal, but not captured by queries applied to the PCORnet CDM, Medicare data, or health plan data, will be obtained by the DCRI Call Center and used to classify the specified participant-reported hospitalizations using the same endpoint coding algorithms that will be applied to the EHR data sources (PCORnet CDM, Medicare data, and health plan data—see figure below. The role of the DCRI Call Center will be especially important for the final months of the follow-up period when, due to inherent data latency associated with these data sources, Medicare data and health plan data will not yet be available for confirming out-of-network hospitalizations reported on the ADAPTABLE participant web portal. Participant-reported hospitalizations that cannot be classified by applying coding algorithms will be clinically reviewed using available medical record data obtained by the DCRI Call Center and confirmed events will be included as trial endpoints.
- Deaths occurring in-hospital at participating network hospitals will be captured with CDM queries, but deaths are often not well represented in EHR data sources, especially out-of-hospital deaths, that are expected to be the majority of the accrued death events during the trial. Medicare data do include information about death events regardless of the location of death, but only for patients enrolled in and eligible for Medicare (Medicare gathers this information from claims, reports from family members, and benefit information collected from the Social Security Administration). The DCRI Call Center will often identify death events from participant-designated alternative contacts during ascertainment of vital status when 2 or more consecutive visits are not completed in the ADAPTABLE participant web portal and/or when final contact with the participant cannot be confirmed. Additionally, the DCRI Call Center will confirm and enter death information in cases where sites notify the study team of out-of-hospital deaths. A participant-locator service may be used during the course of the study to find participants who may have been lost to follow-up. This service will be used to ensure that contact at the end of the trial is made with as many participant as possible who do not complete their final visit to the web portal or complete their final visit via telephone contact with the DCRI Call Center.

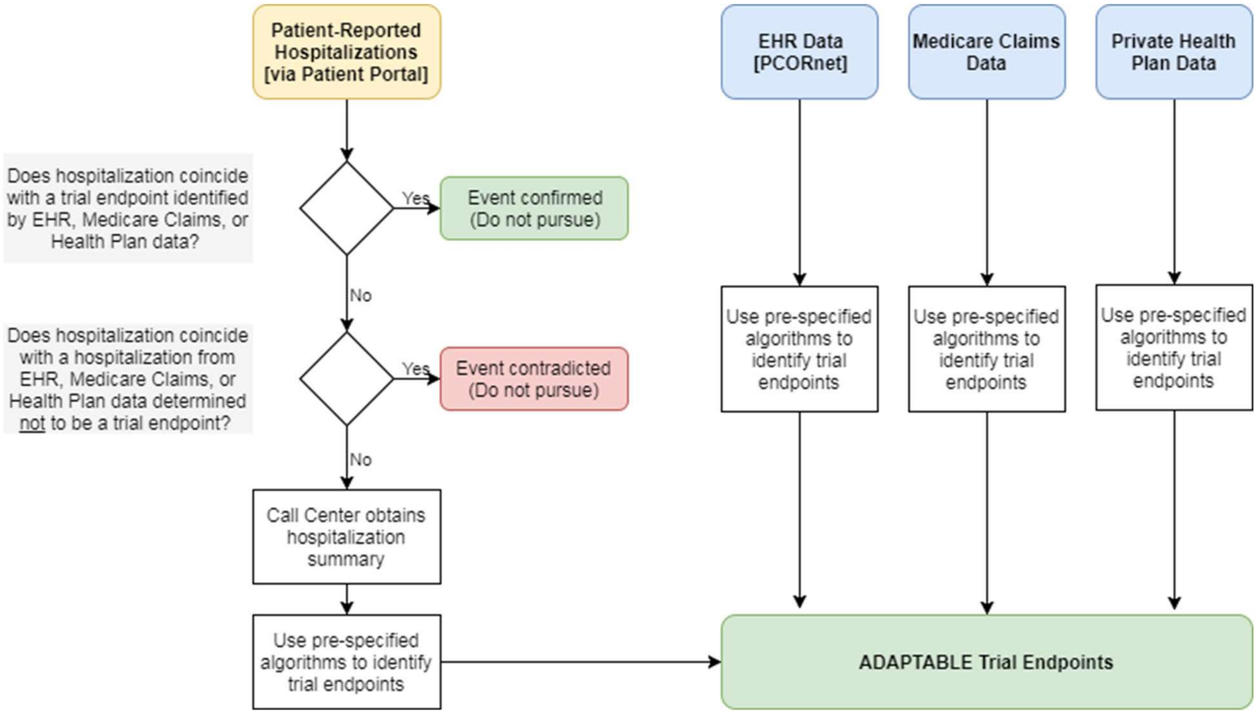

III.E.4.a. Death

This endpoint includes death from any cause (all-cause mortality).

III.E.4.b. Hospitalization for Nonfatal MI

The endpoint of hospitalization for nonfatal MI will be ascertained using ICD-10-CM diagnosis codes (to be listed in the SAP) associated with an encounter in a hospital setting. Priority will be given to diagnosis codes in the principal or primary position. Diagnosis codes in secondary or unknown positions also will be searched for the identification of potential endpoints. The extent to which events identified by diagnosis codes in secondary or unknown positions will or will not be used in the final analysis will be detailed in the SAP. The MI coding algorithm that will guide these efforts was validated for use in FDA's Mini-Sentinel program[92] and has been shown to have a positive predictive value (PPV) of 86%.[93]

III.E.4.c Hospitalization for Stroke

The endpoints of hospitalization for hemorrhagic stroke or ischemic stroke will be ascertained using ICD-10-CM diagnosis codes (to be listed in the SAP) associated with an encounter in a hospital setting. Priority will be given to diagnosis codes in the principal or primary position. Diagnosis codes in secondary or unknown positions also will be searched for the identification of potential endpoints. The extent to which events identified by diagnosis codes in secondary or unknown positions will or will not be used in the final analysis will be detailed in the SAP. The stroke coding algorithm that will guide these efforts has been shown to have a PPV 85% or greater.[94] Ischemic stroke hospitalizations and hemorrhagic stroke hospitalizations will be incorporated into a combined endpoint of all stroke events.

III.E.4.d. Coronary Revascularization

Coronary revascularization includes all coronary revascularization procedures (PCI/CABG) performed during the study. These will be identified using ICD-10-PCS procedure codes and CPT procedure codes (to be listed in the SAP).

III.E.4.e. Hospitalization for Major Bleeding

The endpoints of hospitalization for intracranial bleeding, GI bleeding, and bleeding at another location will be ascertained using ICD-10-CM diagnosis codes (to be listed in the SAP) in a hospital setting. Priority will be given to diagnosis codes in the principal or primary position. Diagnosis codes in secondary or unknown positions also will be searched for the identification of potential endpoints. The extent to which events identified by diagnosis codes in secondary or unknown positions will or will not be used in the final analysis will be detailed in the SAP. The bleeding coding algorithm that will guide these efforts has been shown to have a PPV of 92%.[95] Additionally, procedure codes will be searched for control of bleeding and blood product transfusions and may be used in combination with the diagnosis codes for the definition of the composite major bleeding outcome. [95]

III.E.4.f. Quality of Life and Functional Status

In addition to the effectiveness and safety endpoints listed above, we will collect data on quality of life and functional status as shown in **Table 5**:

Table 5. PCORI Patient-Reported Outcomes Common Measures – Core and Recommended (LOINC Panel 75418-4)

Domain	Item text	Answer list
General Health	In general, would you say your health is	5=Excellent; 4=Very good; 3=Good; 2=Fair; 1=Poor
Quality of life	In general, would you say your quality of life is	5=Excellent; 4=Very good; 3=Good; 2=Fair; 1=Poor
Physical Function	Are you able to run errands and shop?	5=Without any difficulty; 4=With a little difficulty; 3=With some difficulty; 2=With much difficulty; 1=Unable to do
Depression	In the past 7 days…I felt depressed	1=Never; 2=Rarely; 3=Sometimes; 4=Often; 5=Always
Fatigue	During the past 7 days…I feel fatigued	1=Not at all; 2=A little bit; 3=Somewhat; 4=Quite a bit; 5=Very much
Sleep Disturbance	In the past 7 days…I had a problem with my sleep	1=Not at all; 2=A little bit; 3=Somewhat; 4=Quite a bit; 5=Very much
Social Roles & Activities	I have trouble doing all of my regular leisure activities with others	5=Never; 4=Rarely; 3=Sometimes; 2=Usually; 1=Always

III.E.5. Events Collection

(Refer to **Section III.A.5.d. Identifying Endpoints**.)

These data will be analyzed, and the trial statistician will provide them to the DSMB regularly. The DSMB will review the data every 6 months and recommend any necessary changes to the conduct of the trial. Given that aspirin has been used for more than a century and most SAEs have been reported in conjunction with its use, the rate of unsuspected SAEs that the sites will/can submit through MedWatch to the FDA is expected to be very low.

III.F. Summary of Statistical Methods

III.F.1. Baseline Demographic, Clinical, Functional, and Procedural Characteristics

Descriptive summaries of baseline demographic and clinical variables will be generated for each randomized treatment arm of the study. Continuous baseline variables will be presented as medians with 25th and 75th percentiles, and discrete variables will be summarized using frequencies and percentages.

III.F.2. Populations for Analysis

The ITT population will consist of all patients randomized to a treatment group in the study regardless of their compliance with the study medication. For all analyzed using the ITT population, participants will be analyzed as randomized.

The per-protocol population is a subset of the ITT population, excluding participants who complied with the randomized treatment for less than 50% of the follow-up or had major protocol deviations expected to affect the primary effectiveness or safety endpoint.

III.F.3. Primary Effectiveness Comparison

The primary endpoint of this study will be survival free from the first event of a composite of all-cause death, hospitalization for nonfatal MI, or hospitalization for nonfatal stroke. Specifications for the identification of these endpoints are provided in **Section III.A.5.** The primary effectiveness analysis will be performed by the ITT principle based on randomized treatment assignment. Event-free survival rates will be compared using Cox proportional-hazards models.[96] In the absence of any other covariates, this is the same as the log-rank test.[90] The test will be 2-tailed and will be performed at an overall alpha of 0.05.

III.F.4. Primary Safety Comparison

The primary safety endpoint of this study will be the first occurrence of hospitalization for major bleeding as defined in **Section III.A.5.** The primary safety analysis will be performed by the ITT principle based on randomized treatment assignment. Event-free survival rates will be compared using Cox proportional-hazards models, equivalent to the log-rank test. The test will be 2-tailed and will be performed at an overall alpha of 0.05.

III.F.5. Power

The original trial sample size of 20,000 participants was chosen to provide 85% power to detect a 15% RR reduction assuming a primary effectiveness endpoint rate of 5% per year in the higher risk arm. Uniform accrual of 24 months and a maximum follow-up of 30 months were assumed and so was a 5% annualized rate of loss to follow-up.

The time required to bring enrollment into a steady-state uniform accrual proved to be longer by 14 months, leading to a currently estimated 38-month anticipated total accrual period. Furthermore, maximum follow-up will be increased to a maximum of 50 months. Finally, the original sample size calculations conservatively assumed a 5% event rate in the higher risk arm, leading to an overall event

rate below 5%. Thus, it was necessary to update the original power calculations with these new assumptions, incorporating actual accrual patterns observed to date and extrapolating the steady state enrollment numbers observed currently.

With more available data at the time of the DSMB meeting in November 2019, we considered primary effectiveness endpoint rates of 4.6%, 4.8%, 5.0% and 5.2% per year in the higher risk arm, the annualized rate of loss to follow-up of 5%, 2-sided significance level alpha of 0.05, total enrollment duration of 38 months, and an extended maximum follow-up period of 50 months. We considered clinically meaningful RR reduction for the treatment effect between aspirin doses for primary effectiveness endpoint of 20%, 17.5%, and 15%. With these data, taking into account enrollment feasibility and the expected event rate and the necessary meaningful risk reduction, we selected a target sample size of 15,000 participants to be enrolled and a maximum follow up duration of 50 months. Table 6 presents calculations performed for a sample size of 15,000 using PASS software.[97]

Assuming an annualized event rate of 4.6% in the higher risk arm and a 15% RR reduction (annualized event rate 3.8% in the lower risk arm) leads to 88% power and requires 1322 primary outcome events.

Table 6

Overall annualized event rate (%)	Relative Risk	Annualized event rate in low risk group (%)	Annualized event rate in high risk group (%)	Number of Events	Power
4.2	0.800	3.7	4.7	1321	0.99
4.2	0.825	3.8	4.6	1320	0.96
4.2	0.850	3.9	4.5	1322	0.88
4.4	0.800	3.9	4.9	1381	0.99
4.4	0.825	4.0	4.8	1381	0.97
4.4	0.850	4.1	4.8	1383	0.89
4.6	0.800	4.1	5.1	1441	0.99
4.6	0.825	4.2	5.0	1442	0.97
4.6	0.850	4.2	5.0	1439	0.90
4.8	0.800	4.3	5.3	1501	1.00
4.8	0.825	4.3	5.3	1502	0.98
4.8	0.850	4.4	5.2	1503	0.92

For the primary safety endpoint of hospitalization for major bleeding, power calculations were based on estimated primary event rates of 2%, 2.5%, and 3% per year (in the higher dose arm); annualized rate of loss to follow-up of 5%; 2-sided significance level alpha of 0.05; 7,500 participants in each treatment arm; total enrollment of 38 months; and a maximum follow-up period of 50 months. The power of the chosen testing strategy to detect a statistically significant difference in hospitalization for bleeding under these assumptions is shown in **Table 7**. Under all event rate scenarios, there will be power greater than 85% to detect an RR reduction of 25%, and for event rates of 2.5% or greater, power will be close to 80% to detect an RR reduction of 20%.

III.F.6. Secondary Endpoints

Secondary endpoints include the components of the primary endpoint, coronary revascularization procedures, quality of life, and functional status (**Section III.A.5.a**). Time-to-event outcomes will be analyzed using the same approach as the one used for the primary endpoint. Variables collected on numerical scales will be analyzed as continuous variables. Linear repeated measures mixed model will be employed to compare the 2 treatments on the changes from baseline.

Table 7

Annualized event rate in higher-dose arm	Relative risk reduction	No. of events	Power
2%	25%	436	86%
	20%	449	66%
	15%	461	42%
2.5%	25%	544	92%
	20%	559	76%
	15%	575	50%
3%	25%	651	96%
	20%	669	83%
	15%	688	58%

III.F.7. Prior Treatment Effect

We expect that most participants recruited into the ADAPTABLE trial will already be treated with pre-randomization aspirin therapy, given the nature of the inclusion criteria and the known high utilization of aspirin for the secondary prevention of coronary artery disease in the US. Therefore, a sensitivity analysis with a 10-day landmark will be performed that excludes all events occurring in the first 10 days following randomization in order to formally test for a legacy effect of aspirin.

III.F.8. Handling of Missing Data

Concerted effort will be made to eliminate or minimize the occurrence of missing data. Participants will grant access to their electronic medical records at enrollment and during study follow-up as well as permission to have their information searched in national databases. If missing data occur despite these efforts, the following statistical techniques will be employed to address them.

First, all patients will be accounted for in all analyses and presentations. For the primary analysis based on event-free survival, participants discontinuing the study prematurely will be censored at the time of discontinuation. However, this approach might lead to biased results if discontinuation does not occur at random. Thus, 2 “sensitivity analyses” will be undertaken:

1. **Inverse probability weighting.** In this approach, the contribution of each participant to the risk set calculated at time t will be inversely weighted by the estimated probability of remaining uncensored up to time t. This probability will be estimated using a Cox proportional hazards model fitted to time to censoring with variables potentially prognostic of both failure and censoring, with baseline and time-dependent (such as most frequent major protocol deviations, certain AEs, etc.), entered as covariates. In order to reduce the potentially high variability of the resulting treatment-effect estimators due to sampling variability in weights, the weights will be “stabilized” by multiplication of probabilities of remaining uncensored up to time t estimates using baseline covariates only.

2. **Pattern-mixture approach.** Following Little et al.,[98,99] we will assume that for participants who drop out, the hazard of an outcome deviates from that of participants who do not drop out by an offset, denoted by *d1* for the higher dose and by *d0* for the lower dose. We will then explore the effect of this deviation on the findings for various choices of the offsets in the 2 study groups. If the treatment effect is qualitatively maintained for the range of offsets that are considered to be clinically plausible, then the findings will be considered to be robust.

In other analyses, missing data will be handled using multiple imputations. Ten imputed data sets will be generated with imputation methods based on the regression or Monte Carlo framework. Final results will be based on averages from the 10 imputed data sets with appropriate estimator employed of the variance.[99]

III.F.9. Subgroup Analyses (Heterogeneity of Treatment Effect)

Subgroup analyses for the primary effectiveness and safety endpoints will be performed on the ITT population to explore whether the treatment effect is consistent across subgroups. Subgroup analyses to evaluate variation in treatment effect will be performed on the basis of tests for interaction using the Cox proportional hazards model with terms for treatment group, the subgroup variable, and treatment by subgroup variable interaction. Additionally, treatment effects within each categorical subgroup will be examined separately using Cox proportional hazards models. Event rates by treatment and HRs with 95% CIs will be reported for each subgroup. Forest plots will be generated displaying the estimated HRs, and 95% CIs for each subgroup will be presented. For subgroups defined using continuous variables, the analysis based on the continuous form will be considered primary, but for display purposes these variables can also be categorized.

The following subgroups determined at baseline will be examined:

- Age 65 years or older
- Race and ethnicity categories (white, Black, and Asian race; Hispanic ethnicity)
- Diabetes mellitus
- Chronic kidney disease (serum creatinine 1.5 mg/dL or greater)
- Current $P2Y_{12}$ inhibitor use
- Female sex

Table 8

Subgroup size as % of total	Relative risk reduction	Power
10%	25%	37%
	20%	25%
	15%	16%
30%	25%	81%
	20%	60%
	15%	38%
50%	25%	95%
	20%	82%
	15%	57%
70%	25%	99%
	20%	93%
	15%	71%
90%	25%	99%
	20%	97%
	15%	81%

Although the importance of understanding the effects of aspirin in these subgroups is critical, we recognize that despite enrolling 15,000 participants, our power to detect statistically significant and clinically meaningful interactions will be limited. However, this effort represents the largest such undertaking and is of vital importance. Heterogeneity of treatment effect will be established based on the interaction test specified above. Testing for differences between treatment arms within subgroups will be considered exploratory, and no claims of heterogeneity will be made based on tests

within subgroups. By their nature, these tests have low power unless the effect sizes are large, as illustrated in **Table 8.**

Participants without internet access will not be analyzed as a pre-specified sub-group, but sensitivity analyses for the main trial endpoints will be incorporated into the SAP to analyze the populations with vs without internet access to assess the consistency of the treatment results and potential treatment interactions, given expected demographic and socioeconomic differences between the groups.

III.F.10. Interim Blinded Trial Monitoring

During the conduct of the trial, state of the art statistical monitoring techniques will be employed. These will be conducted in a blinded fashion. Study discontinuation patterns will be observed, as well as reasons for the missing data. The event rate will be monitored as it accumulates, and event rate projections will be developed to help us determine if and when we are likely to achieve sufficient power. Baseline characteristics of enrolled participants will also be reviewed to ensure that the population defined in the protocol is enrolled. The primary safety events will be monitored as they accumulate.

III.G. Study Coordination and Monitoring

III. G.1. Site Management and Quality Assurance

This trial will be monitored using quality-by-design principles[100] and will not require on-site monitoring.

Final success of any clinical trial in answering the question of interest depends in large part on its design. The term "quality by design"[100] refers to steps taken at the trial-design stage to foresee and limit problems that might occur during the trial conduct. Adherence to the following 5 "guiding principles" greatly increases the chances of final success:

1. Have we enrolled the right participants according to the protocol with adequate consent (Right Patient)?
2. Did participants receive the assigned treatment and did they stay on the treatment (Right Treatment)?
3. Was there complete ascertainment of primary and secondary outcome data (Right Outcomes)?
4. Was there complete ascertainment of primary and secondary safety data (Right Outcomes)?
5. Were there any major Good Clinical Practice (GCP)-related issues?

This trial was designed to maximize the likelihood that the 5 principles noted above will be followed. In particular, participant enrollment and consent is facilitated by the carefully selected and operationalized CDRNs that are the cornerstone of the PCORnet initiative. Participant selection facilitated by EHRs should lead to efficient identification and enrollment of patients who satisfy study eligibility and enrichment criteria. These criteria have been chosen in a manner that corresponds to what is routinely available in EHRs, reducing the risk of any ambiguity and enrollment of patients who do not meet the protocol-specified criteria.

Although it is not possible to guarantee that all study participants will remain on the trial until its end and fully adhere to the study drug, the existence of the ADAPTABLE patient portal and potential follow-

up from the DCRI Call Center are expected to keep participants engaged and help them maintain their assigned treatment by regularly asking them about the treatment they take. At the same time, because this trial is intended to describe what happens in a "real-world" population (ITT principle), variability in treatment adherence may actually contribute to better generalizability of study results.

Similarly to the eligibility criteria, primary efficacy and safety endpoints have been defined in a way that is well synchronized with the endpoint collection tools that will be employed. The focus on "hard" endpoints (that is, endpoints that can be unambiguously determined), cause-specific hospitalizations for MI, stroke, and bleeding, greatly reduces the potential of recall bias by study participants during self-report. At the same time, it facilitates improved ascertainment through the corresponding EHRs. Accordingly, the CRFs are short and questions are phrased in a manner that does not overwhelm study participants. Access to EHRs and consent to search death records should also reduce the amount of missing primary endpoint data. Minimal levels of intervention and a focus on observing rather than influencing the study participants greatly increases the likelihood that GCPs will be followed.

III.G.2. Study Network

The Study Network of recruiting sites is composed of CDRNs and HealthCore/Anthem Research Network (HCARN) ,an HPRN, within PCORnet. Each of these CDRNs and the HPRN have developed a specific plan to engage clinicians, identify and recruit eligible participants, and facilitate participant follow-up in the trial. The Coordinating Center will perform specific centralized functions; this will be advantageous to meet study budget limitations.

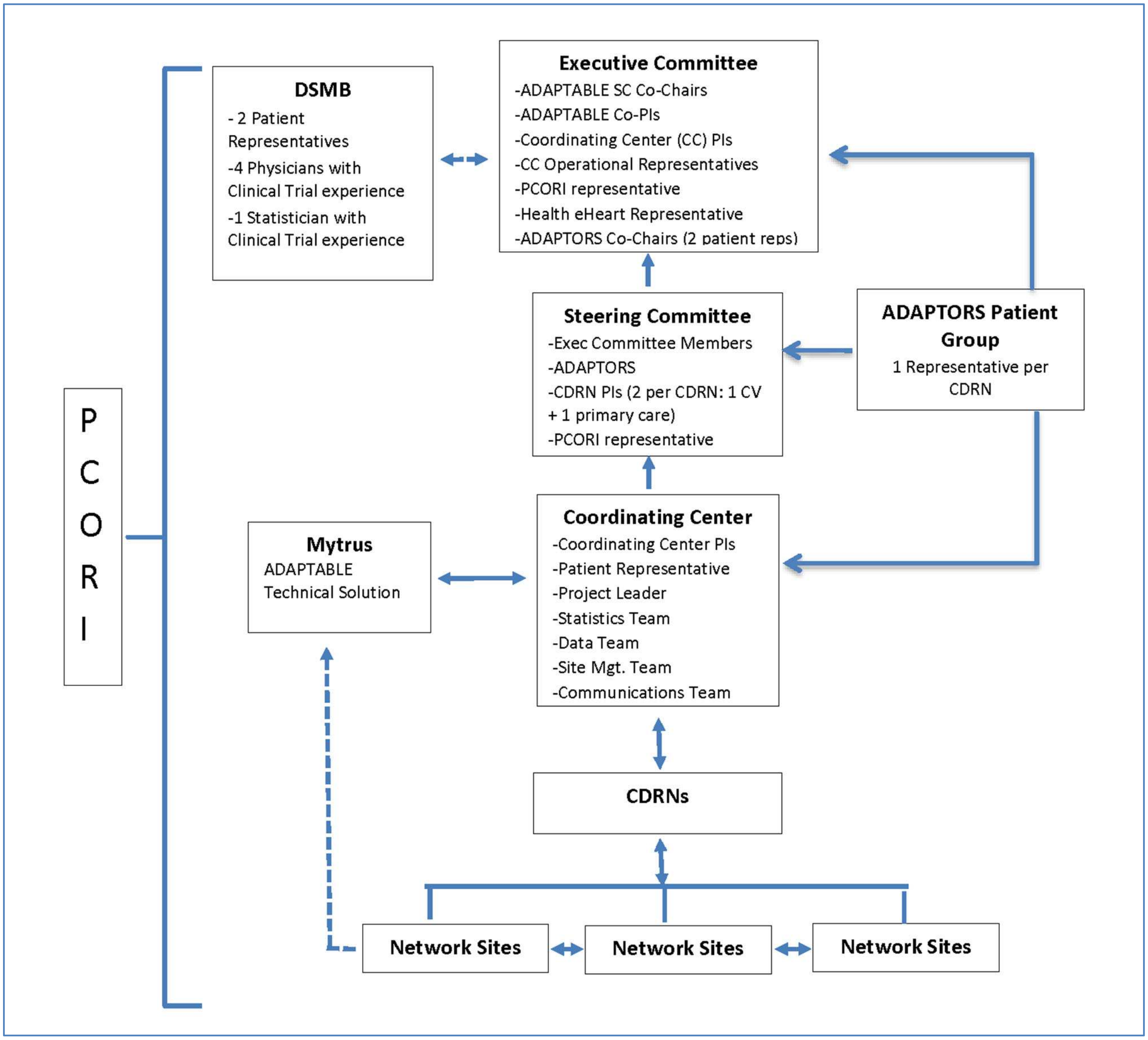

Figure 1. ADAPTABLE Leadership Structure

III.G.3. Executive Committee

The ADAPTABLE Executive Committee will provide oversight and guidance for overall trial activities. It will also serve as a communication nexus to promote sharing of information and cross-pollination of ideas among the Executive Committee, Steering Committee, study co-principal investigators (PIs), Coordinating Center PIs and staff, PCORI representatives, and the co-chairs of the Patient Representatives/Adaptors group, all of whom will have representation on the Committee. The decision-making structure and process for the Executive Committee will be described in a study leadership charter.

III.G.4. Steering Committee

The trial's Steering Committee will be responsible for the protocol and the scientific conduct of the study. The Committee will include patient and clinical representatives from each participating CDRN, HPRN, and PPRN, along with selected network members with expertise in clinical research in antiplatelet therapies, cardiovascular disease, and analytical methods. Additionally, representatives from the American College of Cardiology, the American Heart Association, and PCORI will be on the Steering Committee. The Steering Committee will make decisions about any changes to the protocol and will review all proposed substudies. The decision-making structure and process for the Steering Committee will be described in a study leadership charter.

III.G.5. Data and Safety Monitoring Board (DSMB)

The DSMB will consist of independent members who are not participating investigators in this trial. The committee will include a chair who is a cardiovascular specialist, a statistician, 2 patient representatives, and 3 cardiovascular specialists. The DSMB will review data regularly and recommend necessary changes to the conduct of the trial. For example, if significantly large and important treatment differences are observed during any of the interim analyses, the DSMB may recommend that randomization of patients be stopped, or that the design and conduct of the trial be appropriately modified.

We anticipate that the DSMB will meet at approximately 6-month intervals to review the accumulating data. Before each DSMB meeting, the Data Coordinating Center will conduct the desired statistical analyses and prepare a summary report for review by the DSMB. The extracted data files and analysis programs for each DSMB report will be maintained at the Data Coordinating Center for the life of the study. Each report will describe the progress in enrollment and the rates of compliance with therapy.

The DSMB may recommend stopping the trial for any safety-related concern at any time. Particular attention will be paid to all-cause mortality. For effectiveness, the trial will compare doses of aspirin that have been proven effective in previous trials, and thus it is unlikely that any dramatic differences will appear early. However, the DSMB will monitor these differences about every 6 months over the 3.5 years of the trial. Thus, we anticipate 6 interim analyses and 1 final analysis. Because there are multiple tests, each test must be made at an adjusted alpha level using group sequential-testing methods. To minimize the chance of stopping early due to a spurious result, the endpoints will be tested at the planned analyses using a specific method known as the Haybittle-Peto rule.[101] This rule tests the

endpoints at the 0.001 level (z = 3.29, two-sided) at each interim review and then makes the final test at the 0.0499 level (z = 1.9605). Thus, there is only minimal penalty for interim analyses.

The decision-making structure and process for the DSMB will be described in a separate DSMB charter.

III.G.6. Data Auditing Conventions

Data will be collected via the study patient portal (**Sections III.A.3.c** and **III.A.3.h**).

IV. Human Subjects

IV.A. Protection of Human Subjects

IV.A.1. Sources of Data

Data obtained from the study participant web portal, from the CDM, from Centers for Medicare Services, and from HealthCore/Anthem Research Network, an HPRN, will be transferred to the DCRI along with unique participant identifiers. Details for the process and authorization for Medicare and health plan data linkages are contained in a separate Linkage Plan document. Data contained within the CDM will be obtained from each of the recruiting sites and the HPRN and will be transferred to the DCRI. The control of access to databases at DCRI will be managed centrally by the DCRI through user passwords linked to appropriate access privileges. This protects data from unauthorized view and modifications as well as inadvertent loss or damage. Within the secondary SAS databases, UNIX group access control will be used for maintaining similar security. The Sun workstation login will be secured by extensive user password facilities under UNIX.

IV.A.2. Potential Risks

Aspirin is approved by the US FDA for the secondary prevention of ischemic events associated with ASCVD. In patients with established atherosclerosis, the risk of aspirin is quite low compared with the benefit. A very small number of patients have a serious anaphylactic reaction or bronchoconstriction with nasal polyps when they use aspirin. The risk of intracranial hemorrhage is less than 0.04% per year. A modest number of patients develop serious GI bleeding as a result of loss of the protective effect of prostaglandins on the gastric mucosa. A larger number of patients have GI intolerance, but this is often transient and can be overcome. The absolute risk varies as a function of the trial entry criteria, but none of these major risks exceeds 5 events per 100 patients treated per year. Except for patients with preexisting asthma, no studies have described risk factors for these complications of aspirin use. However, because participants will be selected based on the absence of such known major intolerances to a dose of 325 mg of aspirin, the expected risk of these major events in this trial will be low.

IV.A.2.a. Recruitment and Informed Consent

Each CDRN and the HPRN will develop a specific recruitment process that will work best within their organization, utilizing the tools they have available and their local infrastructure. Prior to cohort identification, site investigators and clinicians will be asked to endorse the protocol, and depending upon their preference, they will either determine each patient's eligibility or will give permission for the

CDRN, through its integrated health system members, to identify and contact potentially eligible patients. Patients who meet trial inclusion criteria will be identified using electronic search algorithms adapted to each network from their aggregated EHR systems. Broad agreement from both cardiovascular specialists and primary care physicians will be sought to enroll eligible participants. Participants will be enrolled after providing electronic informed consent via the study participant portal either directly for those participant s with internet access or via a process facilitated by site research staff during clinic encounters for patients without internet access.

IV.A.2.b. Protection Against Risk

See Sect. IV.A.1. (Sources of Data) above and Section IV.D. (Data and Safety Monitoring Plan) below.

IV.A.2.c. Potential Benefits of the Proposed Research to the Research Participants

As discussed in **Section I.A.**, atherosclerosis leading to thrombotic events, and in particular ASCVD, represents the leading cause of death, morbidity, and disability and affects more than 150 million people worldwide. Despite remarkable progress in preventive and interventional approaches to atherosclerosis, ASCVD is expected to be an even more prominent cause of death and disability for the next 30 years. In technologically developed countries, the major factor that contributes to this expansion is the aging of the population, such that, despite declining age-specific disease rates, the total disease burden increases because ASCVD eventually strikes a larger population of older adults. In developing countries, a major epidemic of atherosclerosis is occurring, concentrated at younger ages and presumably due to the spread of tobacco use, Westernization of diet, and a sedentary lifestyle.

The development of new biological and technological approaches to treating ASCVD is exciting, but maximizing the use of an inexpensive yet effective therapy is far more promising in reducing death and disability on a global scale. Numerous clinical trials have shown the clinical benefit of aspirin vs placebo in reducing vascular events, but the best dose of aspirin for the general population with ischemic heart disease has not been determined. Considering the global burden of ASCVD and that the affected population is growing rapidly, identifying the optimal dose of aspirin would save lives and prevent ischemic and bleeding events globally.

IV.A.2.d. Importance of the Knowledge to Be Gained

The primary aim of this study is to determine the optimal dose of aspirin, the universally available effective therapy for ASCVD. Despite dozens of clinical trials involving over 100,000 patients, the most effective aspirin dose has not been identified. Nonrandomized studies have suggested that lower doses of aspirin may be associated with not only lower rates of bleeding but also reduced ischemic outcomes. Despite guideline recommendations for lower-dose aspirin, over half of post-ACS patients in the US are discharged on a dose of 325 mg. Considering the worldwide epidemic of ASCVD and the potential benefits of aspirin, identifying the optimal dose of aspirin would have substantial global health and economic effects similar to few other medical therapies.

Although the primary aim of this study is to determine the optimal dose of aspirin, this study also represents a new and efficient interactive model for designing and implementing clinical trials that aim

to refine therapies already in use in contemporary clinical practice. Because we live in an era in which the number of effective (or potentially effective) therapies far exceeds our ability to evaluate them in prospective clinical trials using current methods, there is an urgent need to develop an approach to outcome-based trials that can greatly reduce the cost per participant. By following participants directly on the internet and thereby avoiding the costs of clinic visits, lengthy case record forms and extensive site management, we believe that a new, more efficient, and less expensive model for trials can be developed that could be extended to more experimental comparisons. However, working through the issues of informed consent, data validation, events ascertainment, and compliance assessment will require acceptance of novel approaches to statistical sampling and an intense focus on communication with both patients and their physicians. In this era of increasing concern about both patient privacy and research integrity, such an approach to trial efficiency would be exceedingly difficult to pilot with untested therapies. Because this trial will test only doses of aspirin that are considered relatively safe and are widely used in current clinical practice, it provides a critically and globally important clinical issue with which to develop such new clinical trials methods.

This trial also provides an opportunity to explore the use of integrated health systems, EHRs, and patient-reported outcomes as tools for performing clinical trials. A trial conducted almost exclusively over the internet offers many potential benefits. First, participants would have access to a custom-built, patient-centered participant portal that offers current information about symptom awareness, risk factor modification, and disease management and prevention. The participant portal would also serve as the primary mechanism for follow-up, with routine data entry by the participant themselves. Not only would this method examine the practicability of participant self-reporting, but it would potentially enable substantial savings in cost and resources (*e.g.*, costs associated with physician reporting). For physicians, use of the internet in clinical trials could further broaden awareness and participation and, at the same time, facilitate the conduct of the study. For example, trial enrollment and follow-up could be immediate with use of the internet, at any time, eliminating a need for traditional methods (e.g., face-to-face visits). Finally, the platform created by this trial will unite a far-reaching community of patients and their physicians with a common goal of refining an existing therapy to maximize its benefit relative to risk. It is likely that such knowledge will produce far greater global benefit than the introduction of many other “high-tech” approaches.

IV.B. Diminution of Barriers

This study will seek to enroll a diverse population representative of the broad population of patients with ASCVD. The CDRN medical practices are from communities representing both sexes, a wide range of ages, and varied racial and ethnic backgrounds. We will make every attempt to explain the project in easy (non-clinical) terms to all participants to make sure they understand the importance of the research and the need to have good representation of key subgroups and they appreciate the potential benefit they could derive from participating in it. A particular advantage for this trial is the relative lack of barriers to participation. Because clinic visits are not needed for follow-up, additional transportation is not required, and there is minimal additional time commitment for participants. With regard to the specific enrollment of women, this is discussed in detail in **Section III.A.2**. Strategies to enhance the

enrollment of ethnic minorities are also described in detail in **Section III.A.2.** We will strive to enroll a proportion of minority patients similar to that in the overall ASCVD population. Content on the ADAPTABLE patient portal will be available in English and Spanish.

IV.C. Inclusion of Children

Because participation in this trial is restricted to patients aged 18 years and over, inclusion of children in this trial is prohibited. Since the incidence of coronary artery disease among young (i.e., less than 18 years) individuals is very low, children do not represent a study population relevant to this trial.

IV.D. Data and Safety Monitoring Plan

This has been described in detail in **Section III.G.5** (Data and Safety Monitoring Board).

Appendix: Patient Survey for ADAPTOR Participants

Aspirin Trial Questions

The Health eHeart Alliance

Have you had a history of MI (myocardial infarction?)

- ○ Yes
- ○ No
- ○ Don't know

Have you had a history of CAD (Coronary artery disease?)

- ○ Yes
- ○ No
- ○ Don't know

Do you take any of these medications on a regular basis?

Mark all that apply

- ☐ Aspirin 81mg "baby aspirin"
- ☐ Aspirin 325mg "regular aspirin"
- ☐ Aspirin at another dose
- ☐ Apixaban (Eliquis)
- ☐ Clopidogrel (Plavix)
- ☐ Dabigatran (Pradaxa)
- ☐ Prasugrel (Effient)
- ☐ Rivaroxaban (Xarelto)
- ☐ Ticagrelor (Brilinta)
- ☐ Warfarin (Coumadin)

What dose of aspirin do you take?

Answer only if applicable

If you take Aspirin: How often do you usually take it?

Answer only if applicable

- Every day, 2 times per day
- Every day, 1 time per day
- Every other day
- Less often

Have you ever had to stop taking aspirin because of side effects like a rash, upset stomach or bleeding?

Answer only if applicable

- Yes
- No

If you had side effects from taking aspirin please list them

Answer only if applicable

Would you be willing to be a patient advisor to this study?

This may include: helping with study design, identifying ways to recruit study participants, helping develop a patient-friendly consent form, helping identify study outcomes important to patients, participating in the study's data safety monitoring process and helping interpret and disseminate results.

- Yes
- No

Submit

Never submit passwords through Google Forms.

References

1. Heart Disease Facts: America's Heart Disease Burden. Centers for Disease Control and Prevention, 2014. (Accessed February 5, 2015, at http://www.cdc.gov/heartdisease/facts.htm.)

2. Forrester AW, Lipsey JR, Teitelbaum ML, DePaulo JR, Andrzejewski PL. Depression following myocardial infarction. Int J Psychiatry Med 1992;22:33-46.

3. Schleifer SJ, Macari-Hinson MM, Coyle DA, et al. The nature and course of depression following myocardial infarction. Arch Intern Med 1989;149:1785-79.

4. Havik OE, Maeland JG. Patterns of emotional reactions after a myocardial infarction. J Psychosom Res 1990;34:271-85.

5. Freedland KE, Carney RM, Lustman PJ, Rich MW, Jaffe AS. Major depression in coronary artery disease patients with vs without a prior history of depression. Psychosom Med 1992;4:416-21.

6. Carney RM, Rich MW, Tevelde MA, Saini J, Clark K, Jaffe AS. Major depressive disorder in coronary artery disease. Am J Cardiol 1987;60:1273-5.

7. Hance M, Carney RM, Freedland KE, Skala J. Depression in patients with coronary artery disease: a 12-month follow up. Gen Hosp Psychiatry 1996;1996:61-5.

8. McKhann GM, Borowicz LM, Goldsborough MA, Enger C, Selnes OA. Depression and cognitive decline after coronary artery bypass grafting. Lancet 1997;349:1282-4.

9. Patient-Centered Outcomes Research Institute (PCORI). Limited PCORI funding announcement: Optimal maintenance aspirin dose for patients with coronary artery disease. 2014. (Accessed February 10, 2015, at http://www.pcori.org/announcement/limited-pcori-funding-announcement-optimal-maintenance-aspirin-dose-patients-coronary.)

10. Go AS, Mozaffarian D, Roger VL, et al. Heart Disease and Stroke Statistics—2014 Update A Report From the American Heart Association. Circulation 2014;129:e28-e292.

11. Global, regional, and national age–sex specific all-cause and cause-specific mortality for 240 causes of death, 1990–2013: a systematic analysis for the Global Burden of Disease Study 2013. Lancet 2015;385:117-71.

12. WHO Atlas of Heart Disease and Stroke. World Health Organization. (Accessed January 23, 2015, at http://www.who.int/cardiovascular_diseases/resources/atlas/en/.)

13. Collaborative overview of randomised trials of antiplatelet therapy--I: Prevention of death, myocardial infarction, and stroke by prolonged antiplatelet therapy in various categories of patients. Antiplatelet Trialists' Collaboration. BMJ 1994;308:81-106.

14. Kong DF, Hasselblad V, Kandzari DE, Newby LK, Califf RM. Seeking the optimal aspirin dose in acute coronary syndromes. Am J Cardiol 2002;90:622-5.

15. Yusuf S, Zhao F, Mehta SR, et al. Effects of clopidogrel in addition to aspirin in patients with acute coronary syndromes without ST-segment elevation. N Engl J Med 2001;345:494-502.

16. A randomised, blinded, trial of clopidogrel versus aspirin in patients at risk of ischaemic events (CAPRIE). CAPRIE Steering Committee. Lancet 1996;348:1329-39.

17. Hall HM, de Lemos JA, Enriquez JR, et al. Contemporary patterns of discharge aspirin dosing after acute myocardial infarction in the United States: results from the National Cardiovascular Data Registry (NCDR). Circ Cardiovasc Qual Outcomes 2014;7:701-7.

18. Xian Y, Wang TY, McCoy LA, et al. Dose with outcomes after acute myocardial infarction: Insights from the TRANSLATE-ACS Study. Circulation 2015; pii: CIRCULATIONAHA.114.014992.

19. Yusuf S, Reddy S, Ounpuu S, Anand S. Global burden of cardiovascular diseases: part I: general considerations, the epidemiologic transition, risk factors, and impact of urbanization. Circulation 2001;104:2746-53.

20. U.S. Food and Drug Administration. FDA's Sentinel Initiative. 2014. (Accessed February 10, 2015, 2015, at http://www.fda.gov/Safety/FDAsSentinelInitiative/ucm2007250.htm.)

21. U.S. Food and Drug Administration. Guidance for Industry: Q8(R2) Pharmaceutical Development. Center for Drug Evaluation and Research. 2009.

22. Gum PA, Thamilarasan M, Watanabe J, Blackstone EH, Lauer MS. Aspirin use and all-cause mortality among patients being evaluated for known or suspected coronary artery disease: A propensity analysis. JAMA 2001;286:1187-94.

23. Mauri L, Kereiakes DJ, Yeh RW, et al. Twelve or 30 months of dual antiplatelet therapy after drug-eluting stents. N Engl J Med 2014;371:2155-66.

24. Mehta SR, Bassand J-P, Chrolavicius S, et al. Dose comparisons of clopidogrel and aspirin in acute coronary syndromes. CURRENT-OASIS 7 Investigators. N Engl J Med 2010;363:930-42.

25. Mehta SR, Tanguay J-F, Eikelboom JW, et al. Double-dose versus standard-dose clopidogrel and high-dose versus low-dose aspirin in individuals undergoing percutaneous coronary intervention for acute coronary syndromes (CURRENT-OASIS 7): a randomised factorial trial. Lancet 2010;376:1233-43.

26. DiNicolantonio JJ, Serebruany VL. Exploring PLATO-USA paradox and CURRENT-OASIS 7 trials reveals the benefit of higher aspirin dose. Am J Ther 2015;22:85-6.

27. A comparison of two doses of aspirin (30 mg vs 283 mg a day) in patients after a transient ischemic attack or minor ischemic stroke. The Dutch TIA Trial Study Group. N Engl J Med 1991;325:1261-6.

28. Juul-Möller S, Edvardsson N, Jahnmatz B, Rosén A, Sørensen S, Omblus R. Double-blind trial of aspirin in primary prevention of myocardial infarction in patients with stable chronic angina pectoris. The Swedish Angina Pectoris Aspirin Trial (SAPAT) Group. Lancet 1992;340:1421-5.

29. Diener HC, Cunha L, Forbes C, Sivenius J, Smets P, Lowenthal A. European Stroke Prevention Study. 2. Dipyridamole and acetylsalicylic acid in the secondary prevention of stroke. J Neurol Sci 1996;143:1-13.

30. Farrell B, Godwin J, Richards S, Warlow C. The United Kingdom transient ischaemic attack (UK-TIA) aspirin trial: final results. J Neurol Neurosurg Psychiatry 1991;54:1044-54.

31. Taylor DW, Barnett HJ, Haynes RB, et al. Low-dose and high-dose acetylsalicylic acid for patients undergoing carotid endarterectomy: a randomised controlled trial. ASA and Carotid Endarterectomy (ACE) Trial Collaborators. Lancet 1999;353:2179-84.

32. Beneficial effect of carotid endarterectomy in symptomatic patients with high-grade carotid stenosis. North American Symptomatic Carotid Endarterectomy Trial Collaborators. N Engl J Med 1991;325:445-53.

33. Barnett HJ, Kaste M, Meldrum H, Eliasziw M. Aspirin dose in stroke prevention: beautiful hypotheses slain by ugly facts. Stroke 1996;27:588-92.

34. Bonaca MP, Bhatt DL, Cohen M, et al. Long-term use of ticagrelor in patients with prior myocardial infarction. N Engl J Med 2015;372:1791-800.

35. Gent M, Blakely JA, Easton JD, et al. The Canadian American Ticlopidine Study (CATS) in thromboembolic stroke. Lancet 1989;1:1215-20.

36. Balsano F, Rizzon P, Violi F, et al. Antiplatelet treatment with ticlopidine in unstable angina. A controlled multicenter clinical trial. The Studio della Ticlopidina nell'Angina Instabile Group. Circulation 1990;82:17-26.

37. Hass WK, Easton JD, Adams HP, et al. A randomized trial comparing ticlopidine hydrochloride with aspirin for the prevention of stroke in high-risk patients. Ticlopidine Aspirin Stroke Study Group. N Engl J Med 1989;321:501-7.

38. Leon MB, Baim DS, Popma JJ, et al. A clinical trial comparing three antithrombotic-drug regimens after coronary-artery stenting. Stent Anticoagulation Restenosis Study Investigators. N Engl J Med 1998;339:1665-71.

39. Quinn MJ, Fitzgerald DJ. Ticlopidine and clopidogrel. Circulation 1999;100:1667-72.

40. Yusuf S, Zhao F, Mehta SR, Chrolavicius S, Tognoni G, Fox KK. Effects of clopidogrel in addition to aspirin in patients with acute coronary syndromes without ST-segment elevation. N Engl J Med 2001;345:494-502.

41. Wiviott SD, Braunwald E, Angiolillo DJ, et al. Greater clinical benefit of more intensive oral antiplatelet therapy with prasugrel in patients with diabetes mellitus in the trial to assess improvement in therapeutic outcomes by optimizing platelet inhibition with prasugrel–Thrombolysis in Myocardial Infarction 38. Circulation 2008;118:1626-36.

42. Becker RC, Bassand JP, Budaj A, et al. Bleeding complications with the P2Y12 receptor antagonists clopidogrel and ticagrelor in the PLATelet inhibition and patient Outcomes (PLATO) trial. Eur Heart J 2011;32:2933-44.

43. U.S. Food and Drug Administration. FDA approves blood-thinning drug Brilinta to treat acute coronary syndromes - Boxed warning says daily aspirin doses above 100 miligrams decrease effectiveness. 2011. (Accessed October 3, 2016, at: http://www.fda.gov/NewsEvents/Newsroom/PressAnnouncements/ucm263964.htm.)

44. Levine GN, Bates ER, Bittl JA, et al. 2016 ACC/AHA Guideline Focused Update on Duration of Dual Antiplatelet Therapy in Patients With Coronary Artery Disease: A Report of the American College of Cardiology/American Heart Association Task Force on Clinical Practice Guidelines. J Am Coll Cardiol 2016;68:1082-115.

45. Tohgi H, Konno S, Tamura K, Kimura B, Kawano K. Effects of low-to-high doses of aspirin on platelet aggregability and metabolites of thromboxane A2 and prostacyclin. Stroke 1992;23:1400-3.

46. Patrono C, Coller B, Dalen JE, et al. Platelet-active drugs: the relationships among dose, effectiveness, and side effects. Chest 2001;119:39S-63S.

47. Voora D, Cyr D, Lucas J, et al. Aspirin exposure reveals novel genes associated with platelet function and cardiovascular events. J Am Coll Cardiol 2013;62:1267-76.

48. Gurbel PA, Bliden KP, DiChiara J, et al. Evaluation of dose-related effects of aspirin on platelet function: results from the Aspirin-Induced Platelet Effect (ASPECT) study. Circulation 2007;115:3156-64.

49. Savage MP, Goldberg S, Bove AA, et al. Effect of thromboxane A2 blockade on clinical outcome and restenosis after successful coronary angioplasty. Multi-Hospital Eastern Atlantic Restenosis Trial (M-HEART II). Circulation 1995;92:3194-200.

50. Serruys PW, Rutsch W, Heyndrickx GR, et al. Prevention of restenosis after percutaneous transluminal coronary angioplasty with thromboxane A2-receptor blockade. A randomized, double-blind, placebo-controlled trial. Coronary Artery Restenosis Prevention on Repeated Thromboxane-Antagonism Study (CARPORT). Circulation 1991;84:1568-80.

51. Frelinger AL, III, Li Y, Linden MD, et al. Association of cyclooxygenase-1-dependent and -independent platelet function assays with adverse clinical outcomes in aspirin-treated patients presenting for cardiac catheterization. Circulation 2009;120:2586-96.

52. Bhatt DL, Flather MD, Hacke W, et al. Patients with prior myocardial infarction, stroke, or symptomatic peripheral arterial disease in the CHARISMA Trial. J Am Coll Cardiol 2007;49:1982-8.

53. Tricoci P, Huang Z, Held C, et al. Thrombin-receptor antagonist vorapaxar in acute coronary syndromes. N Engl J Med 2012;366:20-33.

54. Morrow DA, Braunwald E, Bonaca MP, et al. Vorapaxar in the secondary prevention of atherothrombotic events. N Engl J Med 2012;366:1404-13.

55. Kopp E, Ghosh S. Inhibition of NF-kappa B by sodium salicylate and aspirin. Science 1994;265:956-9.

56. Frantz B, O'Neill EA, Ghosh S, Kopp E. The Effect of Sodium Salicylate and Aspirin on NF-κB. Science 1995;270:2017-9.

57. Pierce JW, Read MA, Ding H, Luscinskas FW, Collins T. Salicylates inhibit I kappa B-alpha phosphorylation, endothelial-leukocyte adhesion molecule expression, and neutrophil transmigration. J Immunol 1996;156:3961-9.

58. Yin M-J, Yamamoto Y, Gaynor RB. The anti-inflammatory agents aspirin and salicylate inhibit the activity of I[kappa]B kinase-[beta]. Nature 1998;396:77-80.

59. Stevenson MA, Zhao M-J, Asea A, Coleman CN, Calderwood SK. Salicylic acid and aspirin inhibit the activity of RSK2 kinase and repress RSK2-dependent transcription of cyclic AMP response element binding protein- and NF-κB-responsive genes. J Immunol 1999;163:5608-16.

60. Schwenger P, Bellosta P, Vietor I, Basilico C, Skolnik EY, Vilček J. Sodium salicylate induces apoptosis via p38 mitogen-activated protein kinase but inhibits tumor necrosis factor-induced c-Jun N-terminal kinase/stress-activated protein kinase activation. Proc Natl Acad Sci U S A 1997;94:2869-73.

61. Santos MT, Valles J, Aznar J, Marcus AJ, Broekman MJ, Safier LB. Prothrombotic effects of erythrocytes on platelet reactivity. Reduction by aspirin. Circulation 1997;95:63-8.

62. Weber C, Erl W, Pietsch A, Weber PC. Aspirin inhibits nuclear factor–κB mobilization and monocyte adhesion in stimulated human endothelial cells. Circulation 1995;91:1914-7.

63. Lou J, Povsic TJ, Allen JD, et al. The effect of aspirin on endothelial progenitor cell biology: preliminary investigation of novel properties. Thromb Res 2010;126:e175-9.

64. Steinhubl SR, Badimon JJ, Bhatt DL, Herbert J-M, Lüscher TF. Clinical evidence for anti-inflammatory effects of antiplatelet therapy in patients with atherothrombotic disease. Vasc Med 2007;12:113-22.

65. Marimuthu S, Chivukula RS, Alfonso LF, Moridani M, Hagen FK, Bhat GJ. Aspirin acetylates multiple cellular proteins in HCT-116 colon cancer cells: Identification of novel targets. Int J Oncol 2011;39:1273-83.

66. Lewis JP, Yerges-Armstrong LM, Ellero-Simatos S, Georgiades A, Kaddurah-Daouk R, Hankemeier T. Integration of pharmacometabolomic and pharmacogenomic approaches reveals novel insights into antiplatelet therapy. Clin Pharmacol Ther 2013;94:570-3.

67. Suchindran S, Himmel T, Ortel TL, et al. Abstract 16260: Aspirin-responsive platelet genes are associated with platelet function and cardiovascular events. Circulation 2014;130:A16260.

68. FitzGerald GA, Oates JA, Hawiger J, et al. Endogenous biosynthesis of prostacyclin and thromboxane and platelet function during chronic administration of aspirin in man. J Clin Invest 1983;71:676-88.

69. Voora D, Ortel T, Lucas J, Chi J-T, Becker R, Ginsburg G. Time-dependent changes in non-COX-1-dependent platelet function with daily aspirin therapy. J Thromb Thrombolysis 2012:1-12.

70. Mukherjee D, Nissen SE, Topol EJ. Risk of cardiovascular events associated with selective COX-2 inhibitors. JAMA 2001;286:954-9.

71. Szczeklik A. Adverse reactions to aspirin and nonsteroidal anti-inflammatory drugs. Ann Allergy 1987;59:113-8.

72. Allen LaPointe NM, Kramer JM, DeLong ER, et al. Patient-reported frequency of taking aspirin in a population with coronary artery disease. Am J Cardiol 2002;89:1042-6.

73. Final report on the aspirin component of the ongoing Physicians' Health Study. Steering Committee of the Physicians' Health Study Research Group. N Engl J Med 1989;321:129-35.

74. Garcia-Rodriguez LA, Cattaruzzi C, Troncon MG, Agostinis L. Risk of hospitalization for upper gastrointestinal tract bleeding associated with ketorolac, other nonsteroidal anti-inflammatory drugs, calcium antagonists, and other antihypertensive drugs. Arch Intern Med 1998;158:33-9.

75. Roderick PJ, Wilkes HC, Meade TW. The gastrointestinal toxicity of aspirin: an overview of randomised controlled trials. Br J Clin Pharmacol 1993;35:219-26.

76. Randomised double-blind trial of fixed low-dose warfarin with aspirin after myocardial infarction. Coumadin Aspirin Reinfarction Study (CARS) Investigators. Lancet 1997;350:389-96.

77. Comparison of sibrafiban with aspirin for prevention of cardiovascular events after acute coronary syndromes: a randomised trial. The SYMPHONY Investigators. Sibrafiban versus Aspirin to Yield Maximum Protection from Ischemic Heart Events Post-acute Coronary Syndromes. Lancet 2000;355:337-45.

78. Slattery J, Warlow CP, Shorrock CJ, Langman MJ. Risks of gastrointestinal bleeding during secondary prevention of vascular events with aspirin--analysis of gastrointestinal bleeding during the UK-TIA trial. Gut 1995;37:509-11.

79. Derry S, Loke YK. Risk of gastrointestinal haemorrhage with long term use of aspirin: meta-analysis. BMJ 2000;321:1183-7.

80. Laine L, Peterson WL. Bleeding peptic ulcer. N Engl J Med 1994;331:717-27.

81. Campbell CL, Smyth S, Montalescot G, Steinhubl SR. Aspirin dose for the prevention of cardiovascular disease: a systematic review. JAMA 2007;297:2018-24.

82. Manson JE, Stampfer MJ, Colditz GA, et al. A prospective study of aspirin use and primary prevention of cardiovascular disease in women. JAMA 1991;266:521-7.

83. Grosser T, Fries S, Lawson JA, Kapoor SC, Grant GR, FitzGerald GA. Drug resistance and pseudoresistance: an unintended consequence of enteric coating aspirin. Circulation 2013;127:377-85.

84. Peace A, McCall M, Tedesco T, et al. The role of weight and enteric coating on aspirin response in cardiovascular patients. J Thromb Haemost 2010;8:2323-5.

85. Williams MA, Fleg JL, Ades PA, et al. Secondary prevention of coronary heart disease in the elderly (with emphasis on patients > or =75 years of age): an American Heart Association scientific statement from the Council on Clinical Cardiology Subcommittee on Exercise, Cardiac Rehabilitation, and Prevention. Circulation 2002;105:1735-43.

86. Roger VL, Go AS, Lloyd-Jones DM, et al. Heart disease and stroke statistics—2012 update: a report from the American Heart Association. Circulation 2012;125:e2-e200.

87. Prevalence of Selected Chronic Conditions: United States, 1990-92. National Center for Health Statistics. Hyattsville, MD1993.

88. PCORnet. Data Standards. Security, and Network Infrastructure. 2014. (Accessed February 10, 2015, at http://www.pcornet.org/task-forces/data-standards-security-and-network-infrastructure/.)

89. Seniors as Web Users. 2013. (Accessed January 23, 2015, 2015, at http://www.nngroup.com/articles/usability-for-senior-citizens/.)

90. Keiding N. Logrank Test. Encyclopedia of Biostatistics: John Wiley & Sons, Ltd; 2005.

91. Hlatky MA, Ray RM, Burwen DR, et al. Use of Medicare data to identify coronary heart disease outcomes in the Women's Health Initiative. Circ Cardiovasc Qual Outcomes 2014;7:157-62.

92. U.S. Food and Drug Administration. Welcome to Mini-Sentinel. 2014. (Accessed February 10, 2015, at http://mini-sentinel.org/.)

93. Mini-Sentinel systematic validation of health outcome of interest - Acute myocardial infarction cases report. 2011. (Accessed October 3, 2016, at: http://mini-sentinel.org/work_products/Validation_HealthOutcomes/Mini-Sentinel-Validation-of-AMI-Cases.pdf.)

94. Kokotailo RA, Hill MD. Coding of stroke and stroke risk factors using international classification of diseases, revisions 9 and 10. Stroke 2005;36:1776-81.

95. Arnason T, Wells PS, van Walraven C, Forster AJ. Accuracy of coding for possible warfarin complications in hospital discharge abstracts. Thromb Res 2006;118:253-62.

96. Sasieni P. Cox Regression Model. Encyclopedia of Biostatistics: John Wiley & Sons, Ltd; 2005.

97. Hintze JL. PASS User's Guide II. 2008. (Accessed October 3, 2016 at: http://ncss.wpengine.netdna-cdn.com/wp-content/uploads/2012/09/PASSUG2.pdf.)

98. Little RJ, D'Agostino R, Cohen ML, et al. The prevention and treatment of missing data in clinical trials. N Engl J Med 2012;367:1355-60.

99. Little RJA, Rubin DB. Statistical Analysis with Missing Data. 2nd ed. Hoboken, N.J: Wiley-Interscience; 2002.

100. Juran JM. Juran on Quality by Design: The New Steps for Planning Quality into Goods and Services. Revised ed. New York: Free Press; 1992.

101. Haybittle JL. Repeated assessment of results in clinical trials of cancer treatment. Br J Radiol 1971;44:793-7.

Aspirin Dosing: A Patient-Centric Trial Assessing Benefits and Long-term Effectiveness (ADAPTABLE) Protocol Amendment – Justification of Changes

This document will detail specific justification of changes to the ADAPTABLE protocol as follows.

Section I.A.1. ADAPTABLE Study Design

A new study design slide has been inserted.

Section II.C.3. Aspirin and Platelet $P2Y_{12}$ Inhibitors

The ADAPTABLE leadership committees discussed the new recommendation from the American College of Cardiology/American Heart Association Dual Anti-Platelet Therapy Guidelines published earlier in 2016 (J Am Coll Cardiol 2016;68:1082-115), which is a Class IB-NR recommendation for the use of low-dose aspirin (< 100 mg/day) when aspirin is used together with any $P2Y_{12}$ inhibitor. However, as we detail in the revised wording for this section, the data supplement in this guidelines document underlying this specific recommendation cites numerous nonrandomized, post-hoc analyses that have investigated different aspirin doses both with and without concomitant use of $P2Y_{12}$ inhibitors, except for the CURRENT-OASIS-7 trial that only evaluated low- vs. high-dose aspirin for patients for 30 days after the index ACS event. Furthermore, the guidelines committee specifically endorsed the goals and objectives of the ADAPTABLE trial to determine the optimal dose of aspirin with concomitant DAPT treatment. Therefore, based upon the lack of long-term randomized trial data on the risks and benefits of low- vs. high dose aspirin when used together with a $P2Y_{12}$ inhibitor, it was decided to continue to allow patients treated with concomitant prasugrel or clopidogrel to be included in the ADAPTABLE trial (as patients on ticagrelor were already excluded in the prior version of the protocol, as detailed).

Section III.A.1. Enrollment and Eligibility

Based upon the initial experiences across CDRNs with implementation of the computable phenotype, it was determined that smaller than expected numbers of potentially eligible patients were identified due to a number of reasons. First, many prior MI events and/or prior revascularization procedures occurred at non-CDRN institutions and thus could not be confirmed with CDM data. Second, specific angiographic data (for confirming the degree of coronary stenoses) were found to be difficult to confirm with CDM data. Finally, it was determined that many patients with confirmed ASCVD could be identified with codes that recognized chronic coronary disease, ischemic heart disease, or atherosclerosis that are typically used for billing for outpatient encounters but were not recognized in the prior inclusion criteria. Thus, these outpatient codes were added to the inclusion criteria.

Additionally, it was determined that the prior enrichment criteria for the trial may have limited the number of potentially eligible patients identified with implementation of computable phenotype. Additionally, enrichment criteria have been added to recognize patients with chronic heart failure (who were not identified with the single enrichment criterion of LVEF <50%, since ejection fraction data were found to be difficult to confirm with CDM data) and those with significant hypertension and/or hyperlipidemia (cardiovascular disease risk factors that are known to be associated with an increased risk of long-term cardiovascular events).

The potential for the additional inclusion and enrichment criteria to identify a larger number of confirmed ASCVD patients with an increased risk of a major cardiovascular event is demonstrated by the preliminary data from the REACH Net CDRN shown in Table 1.

Table 1. Implementation of the Computable Phenotype at the REACH Net CDRN

	Current Protocol	Protocol Amendment
Total CDM population	637,361	637,361
Qualifying event		
MI	23,153 (3,785)	23,153 (3,785)
PCI	20,840 (627)	20,840 (627)
CABG	13,870 (1,319)	13,870 (1,319)
Chronic CAD	—	55,327 (23,379)
Total individuals with qualifying event	38,498	61,877
Enrichment criteria		
Age ≥ 65 years	41,699	41,699
Diabetes mellitus	29,254	29,254
Cerebrovascular disease	19,757	19,757
Peripheral artery disease	14,349	14,349
LVEF ≤ 50%	10,389	10,389
Smoker	9,445	9,445
Serum creatinine ≥1.5 mg/dL	Not included*	Not included*
Congestive heart failure	—	22,624
Hyperlipidemia (diagnosis code)	—	48,402
Hypertension (diagnosis code)	—	56,492
Total eligible	8,208	60,988

CABG, coronary artery bypass graft; CAD, coronary artery disease; CDM, common data model; CDRN, clinical data research network; LVEF, left ventricular ejection fraction; MI, myocardial infarction; PCI, percutaneous coronary intervention
()= Individuals with only that qualifying event
*Technical issues with the CDM (that are being worked out) precluded use of this variable

Section III.A.3.c. Enrollment, Randomization, and Drug Allocation

Based upon input from the ADAPTOR patient representatives, it was decided to limit the amount of information collected from enrolled patients after they completed the randomization process on the ADAPTABLE web portal. Therefore, it was clarified in this section

that the enrolling site will be responsible for assuring that randomized patients complete their early first web portal contact (1-3 weeks after randomization). It is felt that this step will improve patient retention in the trial.

III.A.3.f. Data Collection and Follow-up

Details regarding the protected health information (PHI) collected in the CDM have been clarified with additional language within this section. Furthermore, this section clarifies that patients will be asked to voluntarily provide a portion of their Social Security number and all of their health plan identification numbers via the ADAPTABLE web portal to facilitate the linkage to data from Medicare, large national health plans, and the National Death Index for trial follow-up and endpoint ascertainment.

III.A.5.d. Identifying Endpoints

Further clarification is provided how queries will be applied to the CDM and to other data sources (to evaluate potential “out-of-network” hospitalizations). Given the introduction of ICD-10 codes into clinical practice within the last year, this section was revised to reflect that both ICD-9 and ICD-10 codes will be used to confirm specified trial endpoints. The combined ICD-9 and ICD-10 codes that will be used to confirm the trial endpoints will be listed in the final trial statistical analysis plan and were thus removed from this section.

Aspirin Dosing: A Patient-Centric Trial Assessing Benefits and Long-term Effectiveness (ADAPTABLE) Protocol Amendment 3 – Justification of Changes

This document will detail specific justification of changes to the ADAPTABLE protocol with Amendment 3 as follows.

Section I.B. Study Aims

Details about the Health Plan Research Network (HPRN) that is participating as an enrolling site have been added. Revisions to the total trial sample size, the estimated accrual timeline, and the maximum follow-up timeline have been incorporated into this section, and are detailed at the end of this document.

Section III.A.4. Patient Engagement

The section on the ADAPTABLE Co-Learning Community (ACLC) has been modified to reflect the numerous other communication and information sharing mechanisms that have been developed to promote patient engagement across all trial activities.

Section III.B. Enrollment and Eligibility

Since patients without internet access have been enrolled and included in the trial since the last protocol amendment was finalized, the conditional provision for potentially enrolling non-internet patients in the trial was removed.

Section III.C.2. Consent, Randomization, and Drug Allocation

It has been clarified in this section informed consent via the ADAPTABLE patient portal will be obtained for patients without internet access during a process facilitated by site research staff during clinic encounters and telephone contact with the DCRI Call Center will be the method utilized for follow-up.

Section III.C.5. Data Collection and Follow-up

The supplemental linkages to Medicare and private health plan data sources (and authorizations that underlie these data linkages) to capture out-of-network hospitalizations and death events not captured in the CDM are referenced in this section and it is noted that further details will be provided in a separate Linkage Plan document.

Sections III.C.7. ADAPTABLE Patient Portal and III.D.1. Medidata Data Security for the Participant Portal

Mytrus, the company that originally built and has maintained the patient web portal, has been acquired by Medidata since the last protocol amendment, so the name was changed in these sections.

Section III.D.3. Duke Clinical Research Institute Data Security

Since the last protocol amendment, the Duke Clinical Research Institute has merged data infrastructure and data security systems with Duke Health Technology Solutions (DHTS) so this data partner is now acknowledged.

Section III.E.4. Identifying Endpoints

Further clarification is provided how queries will be applied to the CDM and to other listed data sources to ascertain endpoints, confirm patient-reported hospitalizations as non-fatal endpoints, and evaluate patient-reported hospitalizations that may be “out-of-network” hospitalizations not captured in other data sources, including the CDM. Further details are also provided regarding the ascertainment and confirmation of death events through a variety of mechanisms. Additionally, given the introduction of ICD-10 codes into clinical practice within the last year, this section was revised to reflect that primarily ICD-10 codes will be utilized in the coding algorithms to confirm the specified non-fatal trial endpoints. The ICD-10 codes that will be used to confirm the trial endpoints will be listed in the final trial statistical analysis plan.

Section III.F.5. Power

Given the observed enrollment rates, the estimated total accrual period and maximum follow-up have been extended resulting in a longer trial timeline. Additionally, the estimated event rates for the primary effectiveness composite endpoint were revised. As a result, the original power calculations were updated with these new assumptions resulting in a revised total trial sample size of 15,000 patients, as described.

Aspirin Dosing: A Patient-Centric Trial Assessing Benefits and Long-term Effectiveness (ADAPTABLE) Protocol Amendment 4 – Justification of Changes

This document will detail specific justification of changes to the ADAPTABLE protocol with Amendment 4 as follows.

Section I.A. Study Rationale

Revisions to the maximum follow-up timeline (44 months extended to 50 months) have been incorporated into this section.

Section III.E.4. Identifying Endpoints

Revisions to the type of data reviewed and acceptable for endpoint reconciliation particularly as they apply to confirmation of participant-reported hospitalizations have been added into this section. The majority of the records that have been submitted by clinical sites is electronic health records (i.e. clinical records) rather than billing codes or billing records.

Section III.F.5. Power

Given the observed enrollment rates, the estimated total accrual period and maximum follow-up have been extended resulting in a longer trial timeline. Additionally, the estimated event rates for the primary effectiveness composite endpoint were revised. As a result, the original power calculations were updated with these new assumptions resulting in a revised total trial sample size of 15,000 patients with maximum follow-up of 50 months, as described.

Statistical Analysis Plan

Aspirin Dosing: A Patient-Centric Trial Assessing Benefits and Long-Term Effectiveness

"ADAPTABLE"

Protocol History

Version 1.0 May 1, 2020

Prepared by
Hillary Mulder, MS
Biostatistician III
Duke Clinical Research Institute

Lisa Wruck, PhD
Director, Center for Predictive Medicine
Duke Clinical Research Institute

Michael Pencina, PhD
Professor of Biostatistics
Duke Clinical Research Institute

1 Synopsis 3
2 Study Aims 3
2.1.1 Aim 1 3
2.1.2 Aim 2 3
2.1.3 Aim 3 3
3 Data Sources 3
4 Randomization and Drug Allocation 4
5 Study Endpoints 5
5.1 Primary Endpoint 5
5.2 Secondary Endpoints 5
5.3 Primary Safety Endpoint 5
6 Identification of Endpoints 5
7 Summary of Statistical Methods 6
7.1 Baseline Demographics and Medical History 6
7.2 Populations for Analysis 6
7.3 Primary Effectiveness Comparison 6
7.4 Primary Safety Comparison 7
7.5 Censoring Rules 7
7.6 Power 8
7.7 Secondary Endpoints 8
7.8 Prior Treatment Effect 9
7.9 Handling of Missing Data 9
7.9.1 Outcome Dates 9
7.9.2 Event-free Survival Outcomes 10
7.9.3 Longitudinal Outcome Data 10
7.10 Planned Sensitivity Analyses 10
7.11 Subgroup Analyses (Heterogeneity of Treatment Effect) 13
8 Measures of Trial Conduct - Aim 3 13
9 Tables and Figures 14
10 References 15

1 Synopsis

To identify the optimal dose of aspirin for secondary prevention in patients with atherosclerotic cardiovascular disease (ASCVD), we conducted a pragmatic clinical trial in which 15,000 patients who are at high risk for ischemic events were randomly assigned in a 1:1 ratio to receive an aspirin dose of 81 mg/day vs. 325 mg/day. Study participants were enrolled over 38 months. Maximum follow-up was 50 months (extended from originally specified 44 months). The primary endpoint is a composite of all-cause death, hospitalization for MI, or hospitalization for stroke. The primary safety endpoint is hospitalization for major bleeding with an associated blood product transfusion.

Additional details about the inclusion and exclusion criterion are contained in the study protocol. Many of the following details are also contained in the study protocol, with some differences in proposed statistical methodology.

2 Study Aims

The major aims of the trial are to test the effect of an aspirin policy on efficacy and safety outcomes, as well as to identify if events captured through electronic health record (EHR) data can be used appropriately in future trials. More specifically, the aims are as follows:

2.1.1 Aim 1

To compare the effectiveness of two daily doses of aspirin (81 mg and 325 mg) in reducing a composite endpoint of all-cause death, hospitalization for nonfatal MI, or hospitalization for nonfatal stroke in high-risk ASCVD patients. Secondary endpoints will be the components of the composite primary endpoint as well as coronary revascularization procedures (percutaneous coronary intervention [PCI] or coronary artery bypass grafting [CABG]) performed during study follow-up. The primary safety endpoint will be hospitalization for major bleeding complications with an associated blood product transfusion.

2.1.2 Aim 2

To compare the effects of aspirin in selected, pre-specified subgroups of patients, including women vs men, older vs younger patients, racial minority patients vs white patients, patients with vs. without diabetes mellitus, patients with vs. without chronic kidney disease (CKD), and patients with vs. without treatment with a concomitant P2Y12 inhibitor at baseline.

2.1.3 Aim 3

To develop, refine, and evaluate the infrastructure for PCORnet to conduct multiple comparative-effectiveness trials in the future. This aim will be accomplished with a "phased-in" approach that will allow for an initial testing of the PCORnet infrastructure followed by adjustments to the trial operational plan to most efficiently accomplish Aims 1 and 2. Also, we will carefully monitor the recruitment and enrollment patterns within and across participating Clinical Data Research Networks (CDRNs) and a Health Plan Research Network (HPRN) and will provide regular feedback reports to each CDRN and HPRN to promote consistent recruitment practices.

Note that the statistical analysis plan for Aim 3 will be described in a separate document.

3 Data Sources

Data sources include the following: electronic health record (EHR) data organized according to the PCORnet Common Data Model (CDM) format, Medicare claims data (CMS), private health insurance claims and patient reported outcomes (PRO) and patient reported data collected via the study web portal. Patient portal data was collected on a continual basis via the web portal.

CDM data was extracted through queries issued by the DCRI data coordinating center for a pre-specified number of times throughout and at the end of the trial. Claims data were collected periodically at a few specified time intervals.

4 Randomization and Drug Allocation

Patients were randomized via a patient portal in a 1:1 ratio to receive 81 mg vs. 325 mg of aspirin in an open-label fashion. Patients were asked to obtain their randomized aspirin dose at their local pharmacies. The randomization scheme was established before the inception of enrollment. Randomized treatment assignment has been obtained from the patient portal data. Compliance with the randomized aspirin dose assignment has been obtained from patient reported data.

Additionally, patients have been randomly assigned to follow-up intervals of every 3 vs 6 months. By embedding a secondary randomization for follow-up time intervals, the best methods for optimizing participant adherence, compliance, and retention in this pragmatic trial will be investigated.

5 Study Endpoints

5.1 Primary Endpoint

The primary endpoint of this study is the composite rate of all-cause mortality, hospitalization for nonfatal MI, or hospitalization for nonfatal stroke. Traditional reporting of potential endpoints by study sites with independent adjudication by a Clinical Events Committee has not done in this trial given the pragmatic nature of the ADAPTABLE study.

We have conducted an endpoint validation plan to ensure the accuracy of the current endpoint classification methodology when compared with clinical endpoint adjudication processes used in traditional clinical trials. See *ADAPTABLE Validation Plan* for the description of the validation plan, as it has been approved by PCORI. Validation will be summarized in the final report as a separate topic and will not be part of the main trial SAP. The results of the validation will not be incorporated into the primary analyses.

5.2 Secondary Endpoints

Secondary endpoints include the components of the primary endpoint, coronary revascularization procedures (PCI and CABG), and quality of life and functional status.

5.3 Primary Safety Endpoint

The primary safety endpoint is hospitalization for major bleeding. Bleeding events include intracranial hemorrhage, GI bleeding, bleeding in other locations, or a control of bleeding procedure. To qualify as a major bleeding event, there also must be documented evidence of a blood product transfusion within +/- 7 days of the bleed.

6 Identification of Endpoints

The method for identification of clinical endpoints is documented in the protocol. Endpoints were identified through electronic sources (including EHR [PCORnet CDM], CMS claims or private insurance claims data) or patient reported via the patient portal. Confirmation by electronic data is required for patient reported clinical endpoints. Some clinical endpoints, based on patient-reported events that could not be confirmed or refuted by the data sources listed above, were confirmed through medical record review.

For endpoints based on a hospitalization for a specific reason (MI, stroke, bleeding), the primary analysis will include endpoints identified by diagnostic codes both (a) in the principal position, and (b) for which the position is unknown. In the CMS and private insurance claims data, diagnostic code position is always known, and we will always rely on codes in the principal position. In the PCORnet EHR data, most, but not all, sites consistently use the principal/secondary designation for diagnoses. When diagnosis position is unavailable for a specific code, its position is recorded as “Other/Unknown” in the PCORnet CDM. Therefore, in the PCORnet data, when diagnosis position is available, only codes in the principal position will be used; but when diagnosis position is unavailable, all codes will be used. This requirement will ensure that we do not exclude events from sites that do not record this information. We will use diagnosis codes known to be (and designated as) secondary in sensitivity analyses only. More details on endpoint extraction are contained in the Query Technical Specifications.

Sensitivity analyses, including analyses addressing potential misclassification in endpoint definitions, are described in Section 7.10.

7 Summary of Statistical Methods

7.1 Baseline Demographics and Medical History

Descriptive summaries of baseline demographic and clinical variables will be generated for each randomized treatment arm of the study. Continuous baseline variables will be presented as medians with 25th and 75th percentiles, and discrete variables will be summarized using frequencies and percentages.

Patient age, sex, current smoking status, race and ethnicity are reported in the patient portal data as well as in CDM. These characteristics will be summarized from the patient portal data.

Medical history data obtained from CDM includes: atrial fibrillation, prior coronary revascularization, hypertension, coronary artery disease, congestive heart failure, prior MI, diabetes, COPD/Asthma, chronic kidney disease, peripheral artery disease, prior smoking, significant bleeding disorder, cerebrovascular disease, significant GI bleed, intracranial hemorrhage, and peptic ulcer. Comorbidities are identified through ICD 9 and ICD 10 diagnosis and procedure codes and CPT codes in any care setting at any time during the lookback period. The lookback period is 5 years prior to trial enrollment. Codes can be used from any diagnosis position (principal, secondary or other/unknown).

Medical history information obtained from the CDM is limited to diagnosis and procedures codes associated with encounters within the lookback period that occurred in the recruiting site's healthcare system. There is no expectation that codes associated with older or out-of-system encounters will be captured. This is a standard limitation of EHR data.

7.2 Populations for Analysis

The Intent-To-Treat (ITT) Population will consist of all patients randomized to a treatment group in the study regardless of their compliance with the study medication. For all ITT analyses, participants will be analyzed as randomized.

Patients who were inappropriately randomized due to administrative errors (excluding those taking prohibited medications) were withdrawn from the study prior to initiating study drug. These patients were included in the randomization scheme, but will have no study data beyond that and will not be included in the ITT population.

Given the pragmatic nature of the trial and the expected gaps in data on patient compliance with randomized dose, we will not define the per-protocol (PP) population. Sensitivity analyses are described in section 7.10 that address treatment compliance.

7.3 Primary Effectiveness Comparison

The primary endpoint of this study will be event-free survival from the first event of a composite of all-cause death, hospitalization for nonfatal myocardial infarction, or hospitalization for nonfatal stroke. The primary effectiveness analysis will be performed in the ITT population based on randomized treatment assignment. Event-free survival rates will be compared using Cox proportional-hazards models, equivalent to the log-rank test, using censoring rules described in Section 7.5. The test will be two-tailed and will be performed at an overall α of 0.05. The proportional hazards assumption will be checked for the randomized treatment assignment using weighted Schoenfeld residuals. If there is evidence of non-proportionality, a cautious

interpretation of the Cox model hazard ratio will be encouraged, and non-parametric event rate estimates will be emphasized (see below).

In addition to Cox regression, cumulative endpoint event rates will be estimated as a function of follow-up time in each treatment group using Kalbfleisch & Prentice's nonparametric estimator of the cumulative incidence function (CIF). The Kalbfleisch & Prentice CIF estimator is equivalent to the Kaplan-Meier estimator when applied to endpoints that are not subject to competing risks. Cumulative endpoint event rates and differences in cumulative endpoint event rates will be estimated and presented with 95% confidence intervals. These non-parametric analyses are important for descriptive purposes and will be a focus of interpretation if the event rate curves cross. They may also be used to construct summary measures of treatment effect which are interpretable when the proportional hazards assumption is violated.

Sensitivity analyses for potential misclassification and under-reporting of endpoints and for non-adherence to randomized aspirin dose are described in Section 7.10. An additional sensitivity analysis will be performed to take clustering of patients within sites into account, using the robust sandwich covariance estimator.

7.4 Primary Safety Comparison

The primary safety endpoint of this study will be the first occurrence of hospitalization for major bleeding. The primary safety analysis will be performed by the ITT principle based on randomized treatment assignment. Cumulative endpoint event rates will be estimated using Kalbfleisch & Prentice's nonparametric estimator of the CIF, taking into account the competing risk of all-cause death. Event-free survival rates will be compared using the Fine and Gray method, equivalent to the Cox proportional-hazards model when competing risks are present, using censoring rules defined in Section 7.5. Grey's test is the competing risk equivalent to the log-rank test for comparing the equality of event curves. The test will be two-tailed and will be performed at an overall α of 0.05.

7.5 Censoring Rules

In the event that a patient does not experience an efficacy or safety endpoint, the patient will be censored at the earliest of study end date or maximum follow-up time point determined from any of the following data sources: CDM censor date, CMS censor date, private health insurance censor date, or the web portal censor date (last point of contact).We expect the study end date to occur on or before all follow-up time points; however, for completeness, censoring dates are defined below. Patients who withdraw consent for trial follow-up will be censored at the time consent is withdrawn. Patients who consent to limited participation follow-up will be censored at the earliest follow-up time point as delineated.

The censoring date for CDM data will be at the site-specific censoring date, which is determined by the PCORnet Distributed Research Network Operations Center based on data curation query results. The censoring date for CMS will be the minimum of the end of enrollment in fee-for-service or the last date claims data are available. The censoring date for private health insurance claims will be the minimum of the end of enrollment or the last date claims data are available.

Time to event modeling will include the first occurrence of an event where the time to event is calculated as the event date – randomization date +1.

7.6 Power

Calculations were performed using PASS software. For the primary effectiveness endpoint, power calculations were based on an estimated primary event rate of 5.5% per year (in the higher risk arm), annualized rate of loss to follow-up of 5%, two-sided significance level α of 0.05, 7,500 patients in each treatment arm, enrollment of 38 months and a maximum follow-up period of 44 months. The power of the chosen testing strategy to detect a statistically significant difference under these assumptions is 85% if the relative risk reduction is 15%, corresponding to a total of 1246 primary effectiveness events.

In November 2019, the DSMB approved extended follow-up in response to updated power calculations based on observed primary event rates. Assuming 7,500 patients in each treatment arm, annualized rate of loss to follow-up of 5%, two-sided significance level α of 0.05, enrollment of 38 months and maximum follow-up period extended to 50 months, the power to detect a relative risk reduction of 15% is 88% when the overall primary event rate is 4.5% in the higher risk arm, corresponding to a total of 1322 primary effectiveness events.

For the primary safety endpoint, formal power calculations were not performed given uncertainties regarding the frequency of this pragmatic, novelly-defined bleeding endpoint.

7.7 Secondary Endpoints

Secondary endpoints include:

- All cause death
- Hospitalization for nonfatal MI
- Hospitalization for nonfatal stroke
- Coronary revascularization (PCI or CABG)
- Quality of life and functional status – components include:
    - Current Health
    - Physical function
    - Depression
    - Fatigue
    - Sleep disturbance
    - Social roles and activities
    - Pain Interference

Patient reported coronary revascularization events that do not match to one of the electronic sources will not be further verified. As such, these PROs will not be counted as events in the secondary event analyses.

Time-to-event outcomes will be analyzed using Cox proportional-hazards models, equivalent to the log-rank test. The test will be two-tailed and will be performed at an overall α of 0.05. The proportional hazards assumption will be checked for randomized treatment arm using weighted Schoenfeld residuals. If there is evidence of non-proportionality, a cautious interpretation of the Cox model hazard ratio will be encouraged, and non-parametric event rate estimates will be emphasized, as described for the primary effectiveness analysis.

Quality of life and functional status data are collected on numerical scales (1:excellent health-5:worst health). They will be analyzed as continuous variables. Mixed models using restricted maximum likelihood estimation (REML) will be employed to model trajectory of measures over time by treatment group. Mixed models account for the correlation structure imposed by repeated measures within participants while using all available data, from baseline to the end of the study, regardless of exact follow-up time. The intercept and slope will be modeled as random effects. The covariance structure for random effects will be modeled using an

unstructured form. Time from baseline measurement and randomized treatment arm will be included in the model as fixed effect. Time will be tested for linearity using natural cubic splines. An interaction between randomized treatment arm and time will be assessed, as will the overall effect of randomized treatment arm.

Since these measures are discrete scores, it is expected that normality assumptions will not hold. Natural log and other appropriate transformations will be considered.

No formal adjustments for multiple testing will be performed. All main and sensitivity analysis results will be presented and left to the interpretation of the reader.

7.8 Prior Treatment Effect

We expect that most patients recruited into the ADAPTABLE trial will already be treated with pre-randomization aspirin therapy, given the nature of the inclusion criteria and the known high utilization of aspirin for the secondary prevention of coronary artery disease in the United States. Therefore, a sensitivity analysis for the primary efficacy and safety endpoints with a 10-day landmark will be performed that excludes events occurring in the first 10 days following randomization to account for the expected time period of washout from the pre-randomization aspirin dose.

7.9 Handling of Missing Data

Concerted effort will be made to eliminate or minimize the occurrence of missing data. Participants will grant access to their electronic medical records at enrollment and during study follow-up as well as to have their information searched in national databases. During the course of the trial, missing data has been monitored by the operations team via aggregate reports. The DCRI call center has been responsible for locating participants with 2 consecutive missed study visits who have not been confirmed dead through electronic data queries or through contacts with the site research teams. This process has changed since study initiation so that participants are now contacted if there is no completed visit in the prior 6 months.

If, despite these efforts, missing data occur, we will employ statistical techniques appropriate to the type of data, as described below.

Reasons for missing data will be collected and described, including withdrawal of consent for any follow-up and loss to follow-up for portal visits. Descriptive statistics for key baseline characteristics and clinical events prior to study withdrawal or completion will be presented by subgroups defined by availability of follow-up data and by treatment group. All participants in the defined study population will be accounted for in all analyses and presentations.

7.9.1 Outcome Dates

Any partial or completely missing date for a confirmed primary effectiveness or safety outcome at the time of database lock will be imputed as follows:

- If the day is missing, 15th of the month, or the randomization date (if patient randomized after 15th of the same month and same year) will be used;
- If the month is missing, June, or the randomization month (if patient randomized after June and year of the event is same as randomization year) will be used;
- If the complete date is missing, the midpoint between the date of last known event-free visit and end of follow-up will be used.

7.9.2 Event-free Survival Outcomes

For the primary and secondary analyses based on event-free survival, most participants are expected to have at least one source of data for these endpoints (patient portal with confirmation of reported events, CDM, CMS or private claims). Issues with potential incomplete data will be addressed through sensitivity analyses described in Section 7.10. Participants discontinuing the study prematurely and withdrawing consent for trial follow-up will be considered truly missing and will be censored at the time of discontinuation. This approach might lead to biased results if the mechanism of discontinuation is non-ignorable, i.e. the hazard for a censored participant is not the same as that of uncensored participants, conditional on observed data. If more than 5% of participants withdraw consent for trial follow-up, then a tipping point analysis similar to that described by Little et al (2016) will be conducted to assess impact of potential non-ignorable censoring on inference for the primary analysis. A Weibull model of the primary endpoint will be fit to the entire ITT population, with independent variables including an indicator of withdrawal of consent for electronic follow-up and selected baseline characteristics. Other independent variables may be considered. The Weibull model will yield an estimated hazard at the time of withdrawal of consent for each participant adjusted for selected covariates. We then assume that the hazard for a participant who withdraws consent is different from those who do not and allow that difference to vary between treatment groups. An inflation factor will be applied to the hazards, with different inflation factors applied to each treatment group. The resulting hazards will be used to impute events to the end of trial follow-up assuming a Weibull distribution. A Cox model as specified for the primary analysis will be fit to the resulting dataset and treatment effect hazard ratio will be estimated, with standard errors adjusted using standard multiple imputation combining rules. The resulting inference will be examined across a range of clinically plausible inflation factors; if inference from primary analysis is maintained then the results will be considered robust. To aid in interpretation, the mean number of imputed events will be reported for inflation factors.

7.9.3 Longitudinal Outcome Data

Quality of life and functional status secondary endpoints will be measured longitudinally by patient self-report through the portal or call center. Missing data may occur with missed contacts, participant withdrawal, or refusal to answer questions. The mixed model approach planned for analysis of these endpoints yields unbiased inference in the presence of a missing at random (MAR) missingness mechanism, meaning that the distribution of missing data is the same as that of non-missing data, conditional on observed covariates. To assess plausibility of the MAR assumption, we will examine patterns of missingness and characterize participants by missingness pattern and treatment group. If concerns are noted, then a pattern mixture model approach will be considered. If there are no concerns regarding the MAR assumption, but there is significant missing data (more than 10% of participants with more than two missed measures), an inverse probability weighting approach will be considered.

7.10 Planned Sensitivity Analyses

Sensitivity analyses will be conducted to assess robustness of trial conclusions to 1) non-adherence to randomized aspirin dose and 2) under-reporting and misclassification of endpoint data. Sensitivity analyses will be focused on primary analysis of the primary efficacy and safety endpoints, but the methods may be generalized to key secondary endpoints.

Non-adherence. A sensitivity analysis will be conducted in the ITT population in which participant-reported aspirin dose is added to the primary analysis Cox model as a time-varying covariate.

COVID-19. A sensitivity analysis will be conducted in the ITT population in which censoring for the primary efficacy endpoint will occur on January 19, 2020, the day before the first confirmed US case of COVID-19. Although we do not expect a differential impact of randomized aspirin dose, COVID-19 could drastically increase the observed primary efficacy endpoint.

Bayesian Analysis. A sensitivity analysis for the primary efficacy endpoint will be performed using a Bayesian proportional hazards model or a Bayesian piece-wise exponential non-proportional hazards model, pending assessment of the proportional hazards assumption. Using the Bayesian posterior distribution, we will calculate the posterior probability that the unknown treatment specific hazard ratio exceeds thresholds of 1.0, 1.05, 1.10, 1.15 and 1.20 and reciprocals of those numbers. Because Bayesian inferences may be sensitive to the choice of prior distribution, further sensitivity analyses will be performed and reported for a range of possible prior distributions.

Proportional hazards assumption holds.
Bayesian analysis requires the specification of a prior probability distribution representing prior information about the set of unknown model parameters before observing the study data. Because prior information about the treatment effect is limited, we will pre-specify a flat (uniform) prior for the Cox model regression coefficient. The selection of a uniform prior reflects the subjective assessment that prior information about the direction and magnitude of the treatment effect is neutral. It also allows posterior inferences to be dominated by the current study data as opposed to the prior.

Bayesian estimation of the Cox model will be based on the method of Kalbfleisch (1978) which uses the Cox partial likelihood function in place of the full data likelihood. Using the Cox partial likelihood is advantageous because it lends itself to efficient Bayesian MCMC computation via Gibbs sampling and avoids the requirement to specify a prior distribution for the baseline hazard function. Using the partial likelihood in place of the full likelihood has been justified on the grounds that it closely approximates a Bayesian analysis of the full data likelihood when the prior distribution for the baseline hazard function is a highly diffuse gamma process prior (Sinha, Singh, Ibrahim, 2003).

Posterior means and other summaries of the posterior distribution will be calculated using Markov Chain Monte Carlo (MCMC) simulations as implemented in the SAS PHREG procedure. To reduce Monte Carlo error and ensure convergence, we will generate 50,000 sets of simulated parameter values after an initial burn-in period of 2,000 iterations.

Proportional hazards assumption is violated.
The piece-wise exponential model lends itself to simple and efficient Bayesian MCMC computation, allowing the model to be flexible enough to accommodate a variety of shapes for the unknown treatment-specific hazard function. To implement this approach, follow-up time will be divided into discrete time intervals. The treatment-specific hazard function for the primary endpoint will be approximated as a constant function within each treatment group and time interval. A summary measure of treatment effect will then be computed by estimating the ratio of the cumulative average hazard ratio over follow-up. The cumulative average hazard ratio reduces to the hazard ratio statistic when the proportional hazards assumption holds, but does

not rely on the PH assumption for its validity or interpretability. A flat prior will be chosen for all model parameters requiring a prior distribution.

Under-reporting and misclassification of endpoints. The primary and key secondary endpoints are derived from multiple sources reflecting varying levels of data completeness and sensitivity/specificity of electronic phenotype definitions for true diagnosis. It is therefore critical to clarify the assumptions underlying inference around the treatment effect and perform sensitivity analyses to assess robustness of this inference to potential violations of the assumptions.

For the primary analysis (and related secondary analyses), we make the following assumptions:

- Death is reported without error.
- Other components of the primary endpoint may be subject to under-reporting and misclassification but these errors are non-differential, i.e. probability of an error is consistent across randomized treatment groups.
- Patient self-report with confirmation is assumed to have nearly perfect specificity since incorrect self-reports will be excluded by examination of medical record. There may be some under-reporting since identification of the event depends on self-report. Some self-reported events may not be confirmed or contradicted if participant did not consent to confirmation process.
- CDM endpoints may be under-reported since participants may be treated for events outside the CDM health systems or events within the health system may be missed. Misclassification may occur due to imperfect electronic phenotype definitions.
- Claims data (CMS and private) is assumed to be complete for enrolled participants and changes in enrollment status are independent of trial outcome. Misclassification may occur due to imperfect electronic phenotype definitions.

We will perform two sets of sensitivity analyses: 1) to assess impact of under-reporting and 2) to assess impact of misclassification. Because available data sources vary across participants, we summarize the potential for error due to under-reporting and misclassification according to each potential combination of available data. To simplify classification of participants, we will not attempt to account for changes in data sources over time, but rather classify participants according to data sources available for more than half of follow-up time.

	Data sources available				Potential error	
**Scenario**	**Self-report**	**Confirmation**	**CDM**	**Claims (CMS or private)**	**Under-reporting**	**Misclassification**
1	X				Yes	Yes
2	X	X			Yes	No
3	X		X		Yes	Yes
4	X			X	No	Yes
5	X		X	X	No	Yes
6			X		Yes	Yes
7				X	No	Yes
8			X	X	No	Yes

To assess impact of under-reporting due to participants moving or seeking care outside of the CDM health system, we will repeat the primary analysis modifying the censoring rules for CDM data such that the censoring date will be the last date the patient encountered the health system. This will include inpatient and outpatient visits, labs, vitals and prescription medication fills.

Under the assumption that under-reporting is non-differential between treatment groups, the impact on primary analysis inference would be a loss of power. If the primary result is non-significant then we will assess overall impact of under-reporting, by performaing a tipping point analysis to identify how many events would have to be missed to achieve significance. We will take an approach that is adapted and simplified from that described by Little et al (2016) for a sensitivity analysis for missing data. A Weibull model of the primary endpoint will be fit to the entire ITT population, with independent variables including an indicator that participant is at risk for under-reporting (Scenarios 1, 2, 3 and 6 in the table) and selected baseline characteristics. Other independent variables may be considered. Events will be sequentially added for those participants at risk of under-reporting across treatment groups. For each increment, *i* events will be imputed from the Weibull model 1000 times. A Cox model as specified for the primary analysis will be fit to the resulting dataset and treatment effect hazard ratio will be estimated, with standard errors adjusted using standard multiple imputation combining rules. The increment will be increased until the upper limit of the 95% CI for the HR of the treatment effect crosses 1.0.

To assess impact of misclassification of electronic phenotype definitions, we will repeat the primary analysis modifying the code-based definitions of primary endpoints to include codes in 1) principal position only and 2) any position.

To further assess impact of misclassification, we will perform a tipping point analysis expanding on methods developed by Liublinska and Rubin (2014) for missing data in clinical trials. Positive predictive value and negative predictive value will be varied from 0 to 1 to reclassify events or non-events for those participants at risk of misclassification (Scenarios 1 and 3-8). For each combination of PPV and NPV, the corresponding number of reclassified events will be imputed 1000 times. Reclassified events will randomly selected and set to non-events. Reclassified non-events will be generated from the Weibull model described in tipping analysis for under-reporting. A Cox model as specified for the primary analysis will be fit to the resulting dataset and treatment effect hazard ratio will be estimated, with standard errors adjusted using standard multiple imputation combining rules. A 95% confidence interval will be calculated for the HR of the treatment effect. Resulting inference based on comparing confidence limits to 1.0 will be presented in a heat map with positive predictive value and negative predictive value reported on the x- and y-axes. Note that this analysis does not relax the assumption of consistency of misclassification across treatment groups.

7.11 Subgroup Analyses (Heterogeneity of Treatment Effect)

Subgroup analyses for the primary effectiveness and safety endpoints will be performed on the ITT population in order to explore whether the treatment effect is consistent across subgroups. Subgroup analyses to evaluate variation in treatment effect will be performed on the basis of tests for interaction using the Cox proportional-hazards model with terms for treatment group, the subgroup variable and treatment by subgroup variable interaction. Additionally, treatment effects within each categorical subgroup will be examined separately using Cox proportional-

hazards models. Event rates by treatment and HRs with 95% confidence intervals will be reported for each subgroup. Forest plots will be generated displaying the estimated hazard ratios and 95% confidence intervals for each subgroup. For continuous variables, the linearity assumption will be checked for violations using natural cubic splines. In the event that major violations are found, natural cubic splines will be used in the final model. This analysis will be considered primary, but for display purposes, these variables will also be categorized.

The following variables determined at baseline will be examined:

- Age (continuous)
- Race categories (White, Black, and Asian; Hispanic ethnicity)
- Diabetes mellitus
- Chronic kidney disease (serum creatinine > 1.5 mg/dL)
- P2Y12 inhibitor use
- Female sex

We expect homogeneity of treatment effect across subgroups following the results observed in the full sample. Thus, testing for differences between treatment arms within subgroups will be considered exploratory and no claims of heterogeneity will be made based on tests within subgroups.

As a sensitivity analysis, the interaction between randomized treatment arm and internet vs non-internet enrollment will be assessed for the primary efficacy and safety outcomes.

8 Tables and Figures

See appendices for Table Shells.

9 References

Little R.J., D. R. (2012). The Prevention and Treatment of Missing Data in Clinical Trials. *N Engl J Med*, 367:1355-1360.

RJ Little et al. The treatment of missing data in a large cardiovascular clinical outcomes study. Clinical Trials 2016

Liublinska V, and Rubin DB (2014). Sensitivity Analysis for a Partially Missing Binary Outcome in a Two-Arm Randomized Clinical Trial. *Stat Med*, 33(24):4170-4185.

Kalbfleisch, J.D. and R.L. Prentice, The statistical analysis of failure time data, second edition. Vol. 360. 2011: John Wiley & Sons.

Kaplan, E. and P. Meier, Nonparametric estimation from incomplete observations. Journal of the American Statistical Association, 1958. 53: p. 457-481.

Hernan, M.A., The Hazards of Hazard Ratios. Epidemiology, 2010. 21(1): p. 13-15.

Royston, P. and M.K. Parmar, The use of restricted mean survival time to estimate the treatment effect in randomized clinical trials when the proportional hazards assumption is in doubt. Statistics in Medicine, 2011. 30(19): p. 2409-2421.

Péron J., P. Roy, B. Ozenne, L. Roche, M. Buyse. The net chance of a longer survival as a patient-oriented measure of treatment benefit in randomized clinical trials. JAMA oncology. 2016 Jul 1;2(7):901-5.

Statistical Analysis Plan

Aspirin Dosing: A Patient-Centric Trial Assessing Benefits and Long-Term Effectiveness

"ADAPTABLE"

Protocol History

Version 1.0	May 1, 2020
Version 1.1	December 7, 2020
Version 1.2	February 9, 2021

Prepared by
Hillary Mulder, MS
Biostatistician III
Duke Clinical Research Institute

Lisa Wruck, PhD
Director, Center for Predictive Medicine
Duke Clinical Research Institute

Michael Pencina, PhD
Professor of Biostatistics
Duke Clinical Research Institute

1 Synopsis 3
2 Study Aims 3
2.1.1 Aim 1 3
2.1.2 Aim 2 3
2.1.3 Aim 3 3
3 Data Sources 3
4 Randomization and Drug Allocation 4
5 Study Endpoints 5
5.1 Primary Endpoint 5
5.2 Secondary Endpoints 5
5.3 Primary Safety Endpoint 5
6 Identification of Endpoints 5
7 Summary of Statistical Methods 6
7.1 Baseline Demographics and Medical History 6
7.2 Populations for Analysis 7
7.3 Primary Effectiveness Comparison 7
7.4 Primary Safety Comparison 8
7.5 Censoring Rules 8
7.6 Power 8
7.7 Secondary Endpoints 9
7.8 Prior Treatment Effect 10
7.9 Handling of Missing Data 10
7.9.1 Outcome Dates 10
7.9.2 Event-free Survival Outcomes 11
7.9.3 Longitudinal Outcome Data 11
7.10 Planned Sensitivity Analyses 11
7.11 Subgroup Analyses (Heterogeneity of Treatment Effect) 15
8 Tables and Figures 15
9 References 16

1 Synopsis

To identify the optimal dose of aspirin for secondary prevention in patients with atherosclerotic cardiovascular disease (ASCVD), we conducted a pragmatic clinical trial in which 15,000 patients who are at high risk for ischemic events were randomly assigned in a 1:1 ratio to receive an aspirin dose of 81 mg/day vs. 325 mg/day. Study participants were enrolled over 38 months. Maximum follow-up was 50 months (extended from originally specified 44 months). The primary endpoint is a composite of all-cause death, hospitalization for MI, or hospitalization for stroke. The primary safety endpoint is hospitalization for major bleeding with an associated blood product transfusion.

Additional details about the inclusion and exclusion criterion are contained in the study protocol. Many of the following details are also contained in the study protocol, with some differences in proposed statistical methodology.

2 Study Aims

The major aims of the trial are to test the effect of an aspirin policy on efficacy and safety outcomes, as well as to identify if events captured through electronic health record (EHR) data can be used appropriately in future trials. More specifically, the aims are as follows:

2.1.1 Aim 1

To compare the effectiveness of two daily doses of aspirin (81 mg and 325 mg) in reducing a composite endpoint of all-cause death, hospitalization for nonfatal MI, or hospitalization for nonfatal stroke in high-risk ASCVD patients. Secondary endpoints will be the components of the composite primary endpoint as well as coronary revascularization procedures (percutaneous coronary intervention [PCI] or coronary artery bypass grafting [CABG]) performed during study follow-up. The primary safety endpoint will be hospitalization for major bleeding complications with an associated blood product transfusion.

2.1.2 Aim 2

To compare the effects of aspirin in selected, pre-specified subgroups of patients, including women vs men, older vs younger patients, racial minority patients vs white patients, patients with vs. without diabetes mellitus, patients with vs. without chronic kidney disease (CKD), and patients with vs. without treatment with a concomitant P2Y12 inhibitor at baseline.

2.1.3 Aim 3

To develop, refine, and evaluate the infrastructure for PCORnet to conduct multiple comparative-effectiveness trials in the future. This aim will be accomplished with a “phased-in” approach that will allow for an initial testing of the PCORnet infrastructure followed by adjustments to the trial operational plan to most efficiently accomplish Aims 1 and 2. Also, we will carefully monitor the recruitment and enrollment patterns within and across participating Clinical Data Research Networks (CDRNs) and a Health Plan Research Network (HPRN) and will provide regular feedback reports to each CDRN and HPRN to promote consistent recruitment practices.

Note that the statistical analysis plan for Aim 3 will be described in a separate document.

3 Data Sources

Data sources include the following: electronic health record (EHR) data organized according to the PCORnet Common Data Model (CDM) format, Medicare claims data (CMS), private health insurance claims and patient reported outcomes (PRO) and patient reported data collected via the study web portal. Patient portal data was collected on a continual basis via the web portal.

CDM data was extracted through queries issued by the DCRI data coordinating center for a pre-specified number of times throughout and at the end of the trial. Claims data were collected periodically at a few specified time intervals.

4 Randomization and Drug Allocation

Patients were randomized via a patient portal in a 1:1 ratio to receive 81 mg vs. 325 mg of aspirin in an open-label fashion. Patients were asked to obtain their randomized aspirin dose at their local pharmacies. The randomization scheme was established before the inception of enrollment. Randomized treatment assignment has been obtained from the patient portal data. Compliance with the randomized aspirin dose assignment has been obtained from patient reported data.

Additionally, patients have been randomly assigned to follow-up intervals of every 3 vs 6 months. By embedding a secondary randomization for follow-up time intervals, the best methods for optimizing participant adherence, compliance, and retention in this pragmatic trial will be investigated.

5 Study Endpoints

5.1 Primary Endpoint

The primary endpoint of this study is the composite rate of all-cause mortality, hospitalization for nonfatal MI, or hospitalization for nonfatal stroke. Traditional reporting of potential endpoints by study sites with independent adjudication by a Clinical Events Committee has not been done in this trial given the pragmatic nature of the ADAPTABLE study.

We have conducted an endpoint validation plan to ensure the accuracy of the current endpoint classification methodology when compared with clinical endpoint adjudication processes used in traditional clinical trials. See *ADAPTABLE Validation Plan* for the description of the validation plan, as it has been approved by PCORI. Validation will be summarized in the final report as a separate topic and will not be part of the main trial SAP. The results of the validation will not be incorporated into the primary analyses.

5.2 Secondary Endpoints

Secondary endpoints include the components of the primary endpoint, coronary revascularization procedures (PCI and CABG), and quality of life and functional status.

5.3 Primary Safety Endpoint

The primary safety endpoint is hospitalization for major bleeding. Bleeding events include intracranial hemorrhage, GI bleeding, bleeding in other locations, or a control of bleeding procedure. To qualify as a major bleeding event, there also must be documented evidence of a blood product transfusion within +/- 7 days of the bleed.

6 Identification of Endpoints

The method for identification of clinical endpoints is documented in the protocol. Endpoints were identified through electronic sources (including EHR [PCORnet CDM], CMS claims or private insurance claims data) or patient reported via the patient portal (see figure below). Confirmation by electronic data is required for patient reported clinical endpoints. Some clinical endpoints, based on patient-reported events that could not be confirmed or refuted by the data sources listed above, were confirmed through medical record review.

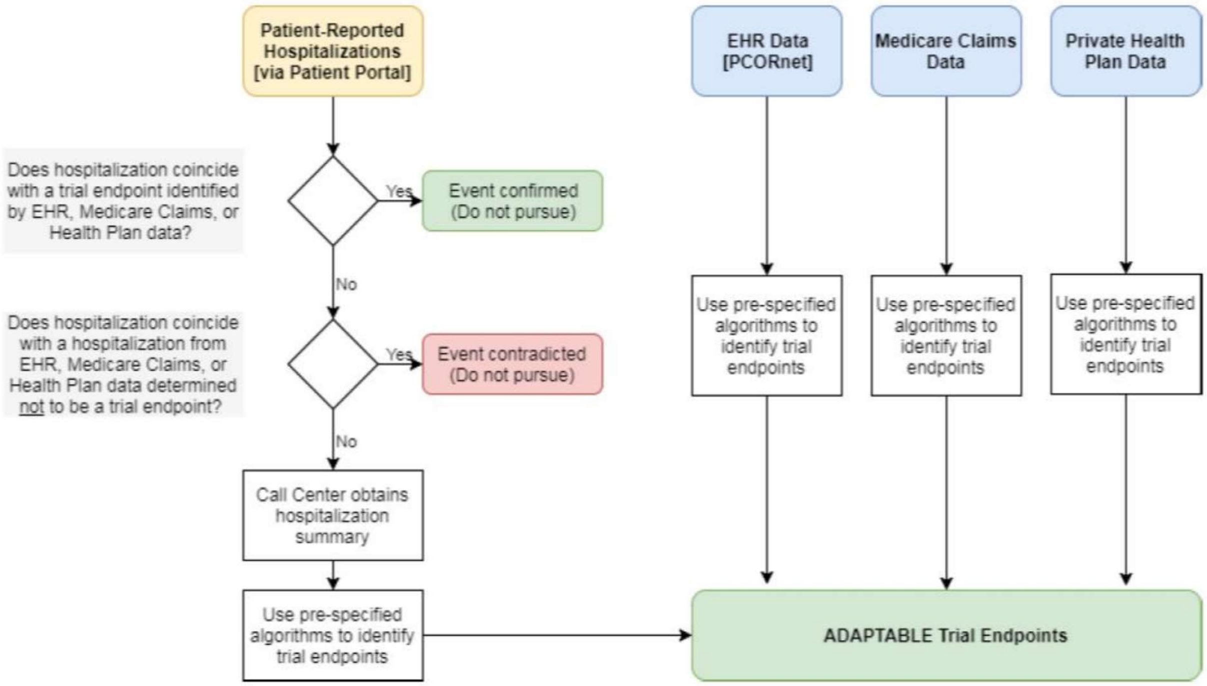

For endpoints based on a hospitalization for a specific reason (MI, stroke, bleeding), the primary analysis will include endpoints identified by diagnostic codes both (a) in the principal position, and (b) for which the position is unknown. In the CMS and private insurance claims data, diagnostic code position for inpatient encounters is usually known, and we will use all codes reported. In the PCORnet EHR data, most, but not all, sites consistently use the principal/secondary designation for diagnoses. When diagnosis position is unavailable for a specific code, its position is recorded as “Other/Unknown” in the PCORnet CDM. Therefore, in the PCORnet data, when diagnosis position is available, only codes in the principal position will be used; but when diagnosis position is unavailable, all codes will be used. This requirement will ensure that we do not exclude events from sites that do not record this information. We will use diagnosis codes known to be (and designated as) secondary in sensitivity analyses only. More details on endpoint extraction are contained in the Query Technical Specifications.

Sensitivity analyses, including analyses addressing potential misclassification in endpoint definitions, are described in Section 7.10.

7 Summary of Statistical Methods

7.1 Baseline Demographics and Medical History

Descriptive summaries of baseline demographic and clinical variables will be generated for each randomized treatment arm of the study. Continuous baseline variables will be presented as medians with 25th and 75th percentiles, and discrete variables will be summarized using frequencies and percentages.

Patient age, sex, current smoking status, race and ethnicity are reported in the patient portal data as well as in CDM. These characteristics will be summarized from the patient portal data.

Medical history data obtained from CDM includes: atrial fibrillation, prior coronary revascularization, hypertension, coronary artery disease, congestive heart failure, prior MI, diabetes, COPD/Asthma, chronic kidney disease, peripheral artery disease, prior smoking, significant bleeding disorder, cerebrovascular disease, significant GI bleed, intracranial hemorrhage, and peptic ulcer. Comorbidities are identified through ICD 9 and ICD 10 diagnosis and procedure codes and CPT codes in any care setting at any time during the lookback period. The lookback period is 5 years prior to trial enrollment. Codes can be used from any diagnosis position (principal, secondary or other/unknown).

Medical history information obtained from the CDM is limited to diagnosis and procedures codes associated with encounters within the lookback period that occurred in the recruiting site's healthcare system. There is no expectation that codes associated with older or out-of-system encounters will be captured. This is a standard limitation of EHR data.

7.2 Populations for Analysis

The Intent-To-Treat (ITT) Population will consist of all patients randomized to a treatment group in the study regardless of their compliance with the study medication. For all ITT analyses, participants will be analyzed as randomized.

Patients who were inappropriately randomized due to administrative errors (excluding those taking prohibited medications) were withdrawn from the study prior to initiating study drug. These patients were included in the randomization scheme, but will have no study data beyond that and will not be included in the ITT population.

Given the pragmatic nature of the trial and the expected gaps in data on patient compliance with randomized dose, we will not define the per-protocol (PP) population. Sensitivity analyses are described in section 7.10 that address treatment compliance.

7.3 Primary Effectiveness Comparison

The primary endpoint of this study will be event-free survival from the first event of a composite of all-cause death, hospitalization for nonfatal myocardial infarction, or hospitalization for nonfatal stroke. The primary effectiveness analysis will be performed in the ITT population based on randomized treatment assignment. Event-free survival rates will be compared using Cox proportional-hazards models, equivalent to the log-rank test, using censoring rules described in Section 7.5. The test will be two-tailed and will be performed at an overall α of 0.05. The proportional hazards assumption will be checked for the randomized treatment assignment using weighted Schoenfeld residuals. If there is evidence of non-proportionality, a cautious interpretation of the Cox model hazard ratio will be encouraged, and non-parametric event rate estimates will be emphasized (see below).

In addition to Cox regression, cumulative endpoint event rates will be estimated as a function of follow-up time in each treatment group using Kalbfleisch & Prentice's nonparametric estimator of the cumulative incidence function (CIF). The Kalbfleisch & Prentice CIF estimator is equivalent to the Kaplan-Meier estimator when applied to endpoints that are not subject to competing risks. Cumulative endpoint event rates and differences in cumulative endpoint event rates will be

estimated and presented with 95% confidence intervals. These non-parametric analyses are important for descriptive purposes and will be a focus of interpretation if the event rate curves cross. They may also be used to construct summary measures of treatment effect which are interpretable when the proportional hazards assumption is violated.

Sensitivity analyses for potential misclassification and under-reporting of endpoints and for non-adherence to randomized aspirin dose are described in Section 7.10. An additional sensitivity analysis will be performed to take clustering of patients within sites into account, using the robust sandwich covariance estimator.

7.4 Primary Safety Comparison

The primary safety endpoint of this study will be the first occurrence of hospitalization for major bleeding. The primary safety analysis will be performed by the ITT principle based on randomized treatment assignment. Cumulative endpoint event rates will be estimated using Kalbfleisch & Prentice's nonparametric estimator of the CIF, taking into account the competing risk of all-cause death. Event-free survival rates will be compared using the Fine and Gray method, equivalent to the Cox proportional-hazards model when competing risks are present, using censoring rules defined in Section 7.5. Grey's test is the competing risk equivalent to the log-rank test for comparing the equality of event curves. The test will be two-tailed and will be performed at an overall α of 0.05.

7.5 Censoring Rules

In the event that a patient does not experience an efficacy or safety endpoint, the patient will be censored at the earliest of study end date or maximum follow-up time point at which the patient can be reasonably assumed to be event free. The maximum follow-up time point will be determined from any of the following data sources: CDM censor date, CMS censor date, private health insurance censor date, or the web portal censor date (last point of contact).We expect the study end date to occur on or before all follow-up time points; however, for completeness, censoring dates are defined below. Patients who withdraw consent for trial follow-up will be censored at the time consent is withdrawn. Patients who consent to limited participation follow-up will be censored at the earliest follow-up time point as delineated.

The censoring date for CDM data will be at the site-specific censoring date, which is determined by the PCORnet Distributed Research Network Operations Center based on data curation query results. The censoring date for CMS will be the minimum of the end of enrollment in fee-for-service or the last date claims data are available. The censoring date for private health insurance claims will be the minimum of the end of claims enrollment or the last date claims data are available.

Time to event modeling will include the first occurrence of an event where the time to event is calculated as the event date – randomization date +1.

7.6 Power

Calculations were performed using PASS software. For the primary effectiveness endpoint, power calculations were based on an estimated primary event rate of 5.5% per year (in the higher risk arm), annualized rate of loss to follow-up of 5%, two-sided significance level α of 0.05, 7,500 patients in each treatment arm, enrollment of 38 months and a maximum follow-up period of 44 months. The power of the chosen testing strategy to detect a statistically significant

difference under these assumptions is 85% if the relative risk reduction is 15%, corresponding to a total of 1246 primary effectiveness events.

In November 2019, the DSMB approved extended follow-up in response to updated power calculations based on observed primary event rates. Assuming 7,500 patients in each treatment arm, annualized rate of loss to follow-up of 5%, two-sided significance level α of 0.05, enrollment of 38 months and maximum follow-up period extended to 50 months, the power to detect a relative risk reduction of 15% is 88% when the overall primary event rate is 4.5% in the higher risk arm, corresponding to a total of 1322 primary effectiveness events.

For the primary safety endpoint, power calculations were based on an estimated primary event rate of 2.5% per year (in the higher risk arm), annualized rate of loss to follow-up of 5%, two-sided significance level α of 0.05, 7,500 patients in each treatment arm, enrollment of 38 months and a maximum follow-up period of 44 months. The power of the chosen testing strategy to detect a statistically significant difference under these assumptions is 81% if the relative risk reduction is 20%, corresponding to a total of 642 primary safety events.

7.7 Secondary Endpoints

Secondary endpoints include:

- All cause death
- Hospitalization for nonfatal MI
- Hospitalization for nonfatal stroke
- Coronary revascularization (PCI or CABG)
- Quality of life and functional status – components include:
    - Current Health
    - Physical function
    - Depression
    - Fatigue
    - Sleep disturbance
    - Social roles and activities
    - Pain Interference

Patient reported coronary revascularization events that do not match to one of the electronic sources will not be further verified. As such, these PROs will not be counted as events in the secondary event analyses.

All-cause death will be analyzed using a Cox proportional hazards model. All other time-to-event outcomes will be analyzed using the Fine and Gray method, equivalent to the Cox proportional-hazards model when competing risks are present, with all-cause death as the competing risk. The test will be two-tailed and will be performed at an overall α of 0.05. The proportional hazards assumption will be checked for randomized treatment arm using weighted Schoenfeld residuals. If there is evidence of non-proportionality, a cautious interpretation of the model hazard ratio will be encouraged, and non-parametric event rate estimates will be emphasized, as described for the primary effectiveness analysis.

Quality of life and functional status data are collected on numerical scales (1:excellent health-5:worst health). They will be analyzed as continuous variables. Mixed models using restricted maximum likelihood estimation (REML) will be employed to model trajectory of measures over time by treatment group. Mixed models account for the correlation structure imposed by repeated measures within participants while using all available data, from baseline to the end of the study, regardless of exact follow-up time. The intercept and slope will be modeled as random effects. The covariance structure for random effects will be modeled using an

unstructured form. Time from baseline measurement and randomized treatment arm will be included in the model as fixed effect. Time will be tested for linearity using natural cubic splines. An interaction between randomized treatment arm and time will be assessed, as will the overall effect of randomized treatment arm.

Since these measures are discrete scores, it is expected that normality assumptions will not hold. Natural log and other appropriate transformations will be considered, as well as several distributions and link functions.

No formal adjustments for multiple testing will be performed. All main and sensitivity analysis results will be presented and left to the interpretation of the reader.

7.8 Prior Treatment Effect

We expect that most patients recruited into the ADAPTABLE trial will already be treated with pre-randomization aspirin therapy, given the nature of the inclusion criteria and the known high utilization of aspirin for the secondary prevention of coronary artery disease in the United States. Therefore, a sensitivity analysis for the primary efficacy and safety endpoints with a 10-day landmark will be performed that excludes events occurring in the first 10 days following randomization to account for the expected time period of washout from the pre-randomization aspirin dose.

7.9 Handling of Missing Data

Concerted effort will be made to eliminate or minimize the occurrence of missing data. Participants will grant access to their electronic medical records at enrollment and during study follow-up as well as to have their information searched in national databases. During the course of the trial, missing data has been monitored by the operations team via aggregate reports. The DCRI call center has been responsible for locating participants with 2 consecutive missed study visits who have not been confirmed dead through electronic data queries or through contacts with the site research teams. This process has changed since study initiation so that participants are now contacted if there is no completed visit in the prior 6 months.

If, despite these efforts, missing data occur, we will employ statistical techniques appropriate to the type of data, as described below.

Reasons for missing data will be collected and described, including withdrawal of consent for any follow-up and loss to follow-up for portal visits. Descriptive statistics for key baseline characteristics and clinical events prior to study withdrawal or completion will be presented by subgroups defined by availability of follow-up data and by treatment group. All participants in the defined study population will be accounted for in all analyses and presentations.

7.9.1 Outcome Dates

Any partial or completely missing date for a confirmed primary effectiveness or safety outcome at the time of database lock will be imputed as follows:

- If the day is missing, 15th of the month, or the randomization date (if patient randomized after 15th of the same month and same year) will be used;
- If the month is missing, June, or the randomization month (if patient randomized after June and year of the event is same as randomization year) will be used;
- If the complete date is missing, the midpoint between the date of last known event-free visit and end of follow-up will be used.

7.9.2 Event-free Survival Outcomes

For the primary and secondary analyses based on event-free survival, most participants are expected to have at least one source of data for these endpoints (patient portal with confirmation of reported events, CDM, CMS or private claims). Issues with potential incomplete data will be addressed through sensitivity analyses described in Section 7.10. Participants discontinuing the study prematurely and withdrawing consent for trial follow-up will be considered truly missing and will be censored at the time of discontinuation. This approach might lead to biased results if the mechanism of discontinuation is non-ignorable, i.e. the hazard for a censored participant is not the same as that of uncensored participants, conditional on observed data. If more than 5% of participants withdraw consent for trial follow-up, then a tipping point analysis similar to that described by Little et al (2016) will be conducted to assess impact of potential non-ignorable censoring on inference for the primary analysis. A Weibull model of the primary endpoint will be fit to the entire ITT population, with independent variables including an indicator of withdrawal of consent for electronic follow-up and selected baseline characteristics. Other independent variables may be considered. The Weibull model will yield an estimated hazard at the time of withdrawal of consent for each participant adjusted for selected covariates. We then assume that the hazard for a participant who withdraws consent is different from those who do not and allow that difference to vary between treatment groups. An inflation factor will be applied to the hazards, with different inflation factors applied to each treatment group. The resulting hazards will be used to impute events to the end of trial follow-up assuming a Weibull distribution. A Cox model as specified for the primary analysis will be fit to the resulting dataset and treatment effect hazard ratio will be estimated, with standard errors adjusted using standard multiple imputation combining rules. The resulting inference will be examined across a range of clinically plausible inflation factors; if inference from primary analysis is maintained then the results will be considered robust. To aid in interpretation, the mean number of imputed events will be reported for inflation factors.

7.9.3 Longitudinal Outcome Data

Quality of life and functional status secondary endpoints will be measured longitudinally by patient self-report through the portal or call center. Missing data may occur with missed contacts, participant withdrawal, or refusal to answer questions. The mixed model approach planned for analysis of these endpoints yields unbiased inference in the presence of a missing at random (MAR) missingness mechanism, meaning that the distribution of missing data is the same as that of non-missing data, conditional on observed covariates. As part of planned secondary analyses following the primary final report, we will examine patterns of missingness and characterize participants by missingness pattern and treatment group to assess plausibility of the MAR assumption. If concerns are noted, then a pattern mixture model approach will be considered. If there are no concerns regarding the MAR assumption, but there is significant missing data (more than 10% of participants with more than two missed measures), an inverse probability weighting approach will be considered.

7.10 Planned Sensitivity Analyses

Sensitivity analyses will be conducted to assess robustness of trial conclusions to 1) non-adherence to randomized aspirin dose and 2) under-reporting and misclassification of endpoint data. Sensitivity analyses will be focused on primary analysis of the primary efficacy and safety endpoints, but the methods may be generalized to key secondary endpoints.

Non-adherence. A sensitivity analysis will be conducted in the ITT population in which participant-reported aspirin dose is added to the primary analysis Cox model as a time-varying covariate.

COVID-19. A sensitivity analysis will be conducted in the ITT population in which censoring for the primary efficacy endpoint will occur on December 31, 2019 the original study end date and prior to COVID entering the United States. Although we do not expect a differential impact of randomized aspirin dose, COVID-19 could drastically increase the observed primary efficacy endpoint.

Bayesian Analysis. As part of planned secondary analyses following the primary final report, a sensitivity analysis for the primary efficacy endpoint will be performed using a Bayesian proportional hazards model or a Bayesian piece-wise exponential non-proportional hazards model, pending assessment of the proportional hazards assumption. Using the Bayesian posterior distribution, we will calculate the posterior probability that the unknown treatment specific hazard ratio exceeds thresholds of 1.0, 1.05, 1.10, 1.15 and 1.20 and reciprocals of those numbers. Because Bayesian inferences may be sensitive to the choice of prior distribution, further sensitivity analyses will be performed and reported for a range of possible prior distributions.

Proportional hazards assumption holds.
Bayesian analysis requires the specification of a prior probability distribution representing prior information about the set of unknown model parameters before observing the study data. Because prior information about the treatment effect is limited, we will pre-specify a flat (uniform) prior for the Cox model regression coefficient. The selection of a uniform prior reflects the subjective assessment that prior information about the direction and magnitude of the treatment effect is neutral. It also allows posterior inferences to be dominated by the current study data as opposed to the prior.

Bayesian estimation of the Cox model will be based on the method of Kalbfleisch (1978) which uses the Cox partial likelihood function in place of the full data likelihood. Using the Cox partial likelihood is advantageous because it lends itself to efficient Bayesian MCMC computation via Gibbs sampling and avoids the requirement to specify a prior distribution for the baseline hazard function. Using the partial likelihood in place of the full likelihood has been justified on the grounds that it closely approximates a Bayesian analysis of the full data likelihood when the prior distribution for the baseline hazard function is a highly diffuse gamma process prior (Sinha, Singh, Ibrahim, 2003).

Posterior means and other summaries of the posterior distribution will be calculated using Markov Chain Monte Carlo (MCMC) simulations as implemented in the SAS PHREG procedure. To reduce Monte Carlo error and ensure convergence, we will generate 50,000 sets of simulated parameter values after an initial burn-in period of 2,000 iterations.

Proportional hazards assumption is violated.
The piece-wise exponential model lends itself to simple and efficient Bayesian MCMC computation, allowing the model to be flexible enough to accommodate a variety of shapes for the unknown treatment-specific hazard function. To implement this approach, follow-up time will be divided into discrete time intervals. The treatment-specific hazard function for the primary endpoint will be approximated as a constant function within each treatment group and time interval. A summary measure of treatment effect will then be computed by estimating the ratio of

the cumulative average hazard ratio over follow-up. The cumulative average hazard ratio reduces to the hazard ratio statistic when the proportional hazards assumption holds, but does not rely on the PH assumption for its validity or interpretability. A flat prior will be chosen for all model parameters requiring a prior distribution.

Under-reporting and misclassification of endpoints. The primary and key secondary endpoints are derived from multiple sources reflecting varying levels of data completeness and sensitivity/specificity of electronic phenotype definitions for true diagnosis. It is therefore critical to clarify the assumptions underlying inference around the treatment effect and perform sensitivity analyses to assess robustness of this inference to potential violations of the assumptions.

For the primary analysis (and related secondary analyses), we make the following assumptions:

- Death is reported without error.
- Other components of the primary endpoint may be subject to under-reporting and misclassification but these errors are non-differential, i.e. probability of an error is consistent across randomized treatment groups.
- Patient self-report with confirmation is assumed to have nearly perfect specificity since incorrect self-reports will be excluded by examination of medical record. There may be some under-reporting since identification of the event depends on self-report. Some self-reported events may not be confirmed or contradicted if participant did not consent to confirmation process.
- CDM endpoints may be under-reported since participants may be treated for events outside the CDM health systems or events within the health system may be missed. Misclassification may occur due to imperfect electronic phenotype definitions.
- Claims data (CMS and private) is assumed to be complete for enrolled participants and changes in enrollment status are independent of trial outcome. Misclassification may occur due to imperfect electronic phenotype definitions.

We will perform two sets of sensitivity analyses: 1) to assess impact of under-reporting on the primary analysis and 2) to assess impact of misclassification on the primary analysis. Because available data sources vary across participants, we summarize the potential for error due to under-reporting and misclassification according to each potential combination of available data. To simplify classification of participants, we will not attempt to account for changes in data sources over time, but rather classify participants according to data sources available for more than half of follow-up time.

	Data sources available				Potential error	
**Scenario**	**Self-report**	**Out of Network Confirmation**	**CDM**	**Claims (CMS or private)**	**Under-reporting**	**Misclassification**
1	X				Yes	Yes
2	X	X			Yes	No
3	X		X		Yes	Yes
4	X			X	No	Yes
5	X		X	X	No	Yes
6			X		Yes	Yes
7				X	No	Yes

8			X	X	No	Yes
9*					Yes	No

*Only information is vital status check at end of trial.

To assess impact of under-reporting due to participants moving or seeking care outside of the CDM health system, we will repeat the primary analysis modifying the censoring rules for CDM data such that the censoring date will be the last date the patient encountered the health system. This will include inpatient and outpatient visits, labs, vitals and prescription medication fills. All other censoring dates will remain the same.

Under the assumption that under-reporting is non-differential between treatment groups, the impact on primary analysis inference would be a loss of power. If the primary result is non-significant then we will assess overall impact of under-reporting, by performing a tipping point analysis to identify how many events would have to be missed to achieve significance. We will take an approach that is adapted and simplified from that described by Little et al (2016) for a sensitivity analysis for missing data. A Weibull model of the primary endpoint will be fit to the entire ITT population, with independent variables including an indicator that participant is at risk for under-reporting (Scenarios 1, 2, 3 and 6 in the table) and selected baseline characteristics. Other independent variables may be considered. Events will be sequentially added for those participants at risk of under-reporting across treatment groups. For each increment, *i* events will be imputed from the Weibull model 1000 times. A Cox model as specified for the primary analysis will be fit to the resulting dataset and treatment effect hazard ratio will be estimated, with standard errors adjusted using standard multiple imputation combining rules. The increment will be increased until the upper limit of the 95% CI for the HR of the treatment effect crosses 1.0.

To assess impact of misclassification of electronic phenotype definitions, we will repeat the primary analysis modifying the code-based definitions of primary endpoints to include codes in 1) principal position only and 2) any position.

To further assess impact of misclassification, we will perform a tipping point analysis expanding on methods developed by Liublinska and Rubin (2014) for missing data in clinical trials. Positive predictive value and negative predictive value will be varied from 0 to 1 to reclassify events or non-events for those participants at risk of misclassification (Scenarios 1 and 3-8). For each combination of PPV and NPV, the corresponding number of reclassified events will be imputed 1000 times. Reclassified events will randomly selected and set to non-events. Reclassified non-events will be generated from the Weibull model described in tipping analysis for under-reporting. A Cox model as specified for the primary analysis will be fit to the resulting dataset and treatment effect hazard ratio will be estimated, with standard errors adjusted using standard multiple imputation combining rules. A 95% confidence interval will be calculated for the HR of the treatment effect. Resulting inference based on comparing confidence limits to 1.0 will be presented in a heat map with positive predictive value and negative predictive value reported on the x- and y-axes. Note that this analysis does not relax the assumption of consistency of misclassification across treatment groups.

7.11 Subgroup Analyses (Heterogeneity of Treatment Effect)

Subgroup analyses for the primary effectiveness and safety endpoints will be performed on the ITT population in order to explore whether the treatment effect is consistent across subgroups. Subgroup analyses to evaluate variation in treatment effect will be performed on the basis of tests for interaction using the Cox proportional-hazards model with terms for treatment group, the subgroup variable and treatment by subgroup variable interaction. Additionally, treatment effects within each categorical subgroup will be examined separately using Cox proportional-hazards models. Event rates by treatment and HRs with 95% confidence intervals will be reported for each subgroup. Forest plots will be generated displaying the estimated hazard ratios and 95% confidence intervals for each subgroup. For continuous variables, the linearity assumption will be checked for violations using natural cubic splines. In the event that major violations are found, natural cubic splines will be used in the final model. This analysis will be considered primary, but for display purposes, these variables will also be categorized.

The following variables determined at baseline will be examined:

- Age (continuous)
- Race categories (White, Black, and Asian; Hispanic ethnicity)
- Diabetes mellitus
- Chronic kidney disease (serum creatinine > 1.5 mg/dL)
- P2Y12 inhibitor use
- Female sex

We expect homogeneity of treatment effect across subgroups following the results observed in the full sample. Thus, testing for differences between treatment arms within subgroups will be considered exploratory and no claims of heterogeneity will be made based on tests within subgroups.

As a sensitivity analysis, the interaction between randomized treatment arm and internet vs non-internet enrollment will be assessed for the primary efficacy and safety outcomes.

8 Tables and Figures

See appendices for Table Shells.

9 References

Little R.J., D. R. (2012). The Prevention and Treatment of Missing Data in Clinical Trials. *N Engl J Med*, 367:1355-1360.

RJ Little et al. The treatment of missing data in a large cardiovascular clinical outcomes study. Clinical Trials 2016

Liublinska V, and Rubin DB (2014). Sensitivity Analysis for a Partially Missing Binary Outcome in a Two-Arm Randomized Clinical Trial. *Stat Med*, 33(24):4170-4185.

Kalbfleisch, J.D. and R.L. Prentice, The statistical analysis of failure time data, second edition. Vol. 360. 2011: John Wiley & Sons.

Kaplan, E. and P. Meier, Nonparametric estimation from incomplete observations. Journal of the American Statistical Association, 1958. 53: p. 457-481.

Hernan, M.A., The Hazards of Hazard Ratios. Epidemiology, 2010. 21(1): p. 13-15.

Royston, P. and M.K. Parmar, The use of restricted mean survival time to estimate the treatment effect in randomized clinical trials when the proportional hazards assumption is in doubt. Statistics in Medicine, 2011. 30(19): p. 2409-2421.

Péron J., P. Roy, B. Ozenne, L. Roche, M. Buyse. The net chance of a longer survival as a patient-oriented measure of treatment benefit in randomized clinical trials. JAMA oncology. 2016 Jul 1;2(7):901-5.

Aspirin Dosing: A Patient-Centric Trial Assessing Benefits and Long-term Effectiveness (ADAPTABLE) Statistical Analysis Plan

Justification of Changes from Version 1.0 to Version 1.2

This document will detail specific justification of changes to the ADAPTABLE Statistical Analysis Plan as follows.

The Statistical Analysis Plan (SAP) was developed in a collaborative fashion by the ADAPTABLE trial leadership (which included the primary statisticians for the study). The initial SAP was created prior to the end of study participation for the ADAPTABLE participants, and necessary changes were made prior to database lock and the analytic phase.

Section 6 Identification of Endpoints

A figure was added to clarify how clinical endpoints were determined in ADAPTABLE (a process that was called Endpoint Reconciliation).

Section 7.5 Censoring Rules

A point of clarification was added to annotate the maximum follow-up time point at which the patient can be reasonably assumed to be event free.

Section 7.6 Power

The pre-specified assumptions about study power for the primary safety endpoint were added from the original protocol. The prior version of the SAP did not include the power calculations for the primary safety endpoint.

Section 7.7 Secondary Endpoints

More details were added for analytic methods for all-cause death and all other time-to-event outcomes. The final version accounted for the competing risk of death, whereas the initial version did not.

Section 7.9.3 Longitudinal Outcomes

More details were added for analytic methods to examine patterns of data missingness. This data will be reported in the Summative Evaluation Plan to be submitted to PCORI with the Final Study Report.

Section 7.10 Planned Sensitivity Analyses

In the sub-section on COVID-19, the date used (December 31, 2019) was changed due to more information on COVID-19 being available at the time of the final SAP.

In the sub-section on “Under-reporting and misclassification of endpoints,” a point of clarification was added to state that the sensitivity analyses would be performed to “assess the impact of under-reporting on the primary analysis.”

ADAPTABLE Endpoint Validation Plan

Background and Rationale:
The ADAPTABLE trial is a pragmatic, randomized controlled trial that will be performed in a less costly and more expedient manner than traditional clinical trials. In carrying out this trial, some common clinical trial processes, such as formal clinical endpoint classification (CEC) or endpoint adjudication will need to be modified in order to ensure efficiency and lower costs. As previously proposed, endpoints will be ascertained by applying published and validated algorithms designed to ensure comprehensive surveillance for all potential primary and secondary endpoints. These data surveillance processes and coding algorithms have been described in detail in the ADAPTABLE protocol. There are concerns that event ascertainment via coding algorithms of administrative claims data needs to demonstrate acceptable levels of validity and be accepted by physicians and patients alike. Multiple publications have measured the accuracy of administrative claims data for classifying non-fatal endpoints when compared with results from traditional endpoint adjudication in clinical trials and registries, and conflicting data exist regarding the agreement between administrative claims data and clinical study adjudication.[1-5] The following sections will outline the ADAPTABLE plan to validate non-fatal clinical endpoints during the course of the study.

Endpoints to Review:
We plan to review non-fatal endpoints including hospitalizations for (1) myocardial infarction (MI), (2) stroke and (3) major bleeding. We do not plan to review death since the primary composite endpoint includes all-cause death, and studies have consistently shown that the standard methods to ascertain vital status (i.e. all-cause death) that will be used in ADAPTABLE are valid and accurate. We will also not review the occurrence of percutaneous coronary intervention (PCI) and coronary artery bypass grafting (CABG) surgery – these are also secondary endpoints in the trial. A prior study comparing the use of administrative claims data with clinical trial data showed a high agreement rate for the confirmation of coronary revascularization procedures ($\kappa = 0.88–0.91$).[5]

Expected Number of Endpoints to Review:
We plan to review a sample of approximately 20% of the MI, stroke, and major bleeding endpoints captured and classified with the coding algorithms described in the ADAPTABLE protocol. Based upon the distribution of death vs. non-fatal events (MI and stroke) in the REACH registry, we estimate that 60% of the primary composite endpoints for ADAPTABLE will be non-fatal MI or non-fatal stroke events (roughly 785 of the 1308 primary endpoint events expected based upon sample size calculations).[6] In order to review each of the primary (non-fatal) efficacy and safety events, we plan to select the first 10 MI events, 10 stroke events, and 5 bleeding events that occur within each CDRN (total=25 events per CDRN). Therefore, we plan to sample approximately 200 overall endpoints to review during the course of the study.

Timing of Validation:
Validation will be performed consistently throughout the conduct of the study. The rationale to perform this over the entire conduct of the study is that changes in administrative claims have been implemented in October 2015 and variable enrollment and accumulation of endpoints in patients at each CDRN will occur from year-to-year throughout the study.

Methods:

Endpoint Ascertainment
The primary goal of this validation plan is to confirm that events that are observed and classified as primary efficacy and safety endpoints in ADAPTABLE with established coding algorithms are comparable to clinical endpoints confirmed with adjudication using methods that are commonly utilized in traditional trials. The primary methods of endpoint identification for ADAPTABLE will occur via routine queries in

the PCORnet CDM. Since all patient-reported events require confirmation, we will not include these events in the validation plan.

Source Document Collection

Once the sample of endpoints is identified, the DCRI Coordinating Center will collect source documents via electronic means, fax, and/or mail for all specified endpoints. Potential source documents include hospital discharge summaries for each endpoint to be validated, brain imaging reports (e.g. CT scans), laboratory values (e.g. hemoglobin levels for bleeding endpoints, cardiac marker values for MI endpoints), and electrocardiograms (for MI endpoints). These source documents will be collated and private health information and treatment assignment (i.e. aspirin dosing) information will be redacted through standard processes before case dossiers are sent to independent physicians for adjudication.

Adjudication

Each selected case will be reviewed in a blinded manner by a disease-specific, expert adjudicator (a single cardiologist will review MI and major bleeding endpoints; a single neurologist will review stroke endpoints). Standard endpoint definitions for MI, stroke, and major bleeding will be used to adjudicate these endpoints using standardized adjudication forms.[7-9]

Analysis:

Once all cases have been reviewed, formal statistical analyses will be performed to measure the percentages of true positive results and false positive results and the positive predictive value for each endpoint that will be validated (non-fatal stroke, non-fatal MI, and major bleeding).

Although events will be adjudicated for this validation plan, these results (if different) will not change the primary analysis of ADAPTABLE which will count those events identified via coding algorithms as described in the protocol.

Potential Pitfalls and Implications:

As with all large clinical trials, it is imperative to systematically identify and classify suspected endpoints through a multi-layered process that includes reporting of potential endpoints by site investigators and routine data queries of the trial clinical and safety databases for potential triggers for unreported endpoints. While this validation plan will allow the study team to determine the agreement rate between administrative claims coding algorithms for endpoint confirmation and independent physician adjudication of endpoints (through review of medical record source documents), it will not re-create the clinical trial process by creating queries and triggers for possible endpoints.

References

1. Kumamaru H, Judd SE, Curtis JR, et al. Validity of claims-based stroke algorithms in contemporary Medicare data: reasons for geographic and racial differences in stroke (REGARDS) study linked with medicare claims. Circulation Cardiovascular quality and outcomes 2014;7:611-9.
2. Thigpen JL, Dillon C, Forster KB, et al. Validity of international classification of disease codes to identify ischemic stroke and intracranial hemorrhage among individuals with associated diagnosis of atrial fibrillation. Circulation Cardiovascular quality and outcomes 2015;8:8-14.
3. Lakshminarayan K, Larson JC, Virnig B, et al. Comparison of Medicare claims versus physician adjudication for identifying stroke outcomes in the Women's Health Initiative. Stroke; a journal of cerebral circulation 2014;45:815-21.
4. Jones SA, Gottesman RF, Shahar E, Wruck L, Rosamond WD. Validity of hospital discharge diagnosis codes for stroke: the Atherosclerosis Risk in Communities Study. Stroke; a journal of cerebral circulation 2014;45:3219-25.
5. Hlatky MA, Ray RM, Burwen DR, et al. Use of Medicare data to identify coronary heart disease outcomes in the Women's Health Initiative. Circulation Cardiovascular quality and outcomes 2014;7:157-62.
6. Wilson PW, D'Agostino R, Sr., Bhatt DL, et al. An international model to predict recurrent cardiovascular disease. The American journal of medicine 2012;125:695-703 e1.
7. Hicks KA, Tcheng JE, Bozkurt B, et al. 2014 ACC/AHA Key Data Elements and Definitions for Cardiovascular Endpoint Events in Clinical Trials: A Report of the American College of Cardiology/American Heart Association Task Force on Clinical Data Standards (Writing Committee to Develop Cardiovascular Endpoints Data Standards). Circulation 2015;132:302-61.
8. An international randomized trial comparing four thrombolytic strategies for acute myocardial infarction. The GUSTO investigators. The New England journal of medicine 1993;329:673-82.
9. Schulman S, Kearon C, Subcommittee on Control of Anticoagulation of the S, Standardization Committee of the International Society on T, Haemostasis. Definition of major bleeding in clinical investigations of antihemostatic medicinal products in non-surgical patients. Journal of thrombosis and haemostasis : JTH 2005;3:692-4.
