# Supplementary material for: Effectiveness and Safety of Enteric-Coated vs Uncoated Aspirin in Patients With Cardiovascular Disease: A Secondary Analysis of the ADAPTABLE Randomized Clinical Trial
Source: JAMA Cardiol. 2023 Oct 4;8(11):1061–9. doi: 10.1001/jamacardio.2023.3364 (PMC10551818; doi:10.1001/jamacardio.2023.3364)
Supplement: Supplement 2. — Trial Protocol. Enteric-Coated Aspirin Portion of ADAPTABLE [file jamacardiol-e233364-s002.pdf]

Enteric Coated Aspirin in the ADAPTABLE Trial

Date: November 17th 2021

Title: **Enteric Coated Effect on Outcomes in Aspirin Dosing and Patient-Reported Outcomes from ADAPTABLE: A Patient-Centric Trial Assessing Benefits and Long-Term Effectiveness**

Principle Investigator: Mark B. Effron,

Corresponding Author: Mark B. Effron (mark.effron@ochsner.org)

Co-Investigator(s): Amber Sleem, Schuler Jones

Principle Statistician: Amanda Stebbins

Statistical Advisor: Lisa Wruck

Target journal: JAMA, JAMA-Cardiology,JACC or JAHA

EPM Code: 6516-XX

Background

Many physicians advise patients with cardiovascular disease to use enteric coated aspirin (EC-ASA) preparations in order to decrease GI bleeding complications. Data from pharmacodynamic studies suggest there is decreaseed platelet inhibition with EC-ASA compared with ASA without EC. This study will evaluate EC-ASA is as clinically effective as uncoated ASA.

Data is mixed on whether enteric coated aspirin reduces the protective effect of ASA in patients with cardiovascular disease. Pharmacodynamic studies suggest that enteric coating on ASA decreases the antiplatelet effect of ASA and results in an increased rate of "ASA resistance", but there are no clear clinical outcomes data that support the relevance of the pharmacodynamic studies.

Given that over 10,000 subjects in ADAPTABLE reported the type of ASA they were using, insight into the benefits and/or detriments of enteric coated ASA in cardiovascular patients can be obained.

Primary Objectives/Aims

Primary Hypothesis: To assess the effectiveness of Enteric coated aspirin compared to plain ASA in preventing cardiovascular events
Hypothesis: *The effectiveness of enteric coated aspirin will be the same as plain aspirin.*

Aim 1: To examine whether the effectiveness and safety of low (81 mg daily) vs. high-dose (325 mg daily) aspirin is consistent between participants taking enteric coated and plain aspirin.
Hypothesis: *The effectiveness and safety of low vs. high-dose aspirin in patients with established ASCVD is consistent between participants administered enteric and plain coated aspirin.*

Aim 2: Describe differences in baseline clinical characteristics and CV risk factors by enteric coated and not plain aspirin.

Aim 2: To examine whether enteric coated aspirin will have the same bleeding rate as plain aspirin.
Hypothesis: *The safety events of enteric coated aspirin will be comparable for patients administered enteric coated and plain aspirin.*

Endpoints

- Efficacy Endpoints
  - Primary
    - Death/MI/Stroke
  - Secondary
    - All-cause mortality
    - Hospitalization for Myocardial Infarction
    - Hospitalization for Stroke
    - Coronary revascularization
    - TIA

- Safety Endpoints
  - Primary
    - Hospitalization for major bleeding with associated blood product transfusion
  - Secondary
    - Hospitalization for GI Bleed
    - Any GI bleed (if reported)

Data

Population:

- All patients randomized in the ADAPTABLE Trial.

Inclusion Criteria:

- Participants reported taking enteric coated or plain asprin.

Exclusion Criteria:

- Participants missing type of aspirin coating.

General Analysis Conventions:

- Unless otherwise noted, all hypothesis tests will be 2-sided. P-values < 0.05 will be interpreted as statistically significant.
- Analyses for this manuscript will be interpreted as exploratory. No formal adjustment for multiple hypothesis testing will be made.
- Missing values will be excluded from descriptive statistical summaries. For multivariable analyses (e.g., multivariable models) the extent and patterns of missing data will be assessed, and a suitable imputation approach will be implemented.
- Analysis will be performed using SAS 9.4 (or higher version). Other software may be used if required and determined to be appropriate.

Quality Assurance Measures:

- To reduce the risk of error, all tables and figures will be generated in an automated fashion. In the rare circumstance where results are “cut and pasted” visual checks will be performed to ensure errors were not made.
- The reviewing statistician will provide code review as needed before results are finalized, particularly for objectives that involve complex statistical modelling.

Analysis Objectives

1. **Objective**: Compare baseline characteristics of patients by enteric coated and plain aspirin.

   Analysis: Continuous variables will be presented as median and $25^{th}/75^{th}$ percentiles, mean, standard deviation and minimum and maximum values. Categorical variables will be presented as counts and proportions. P-values will be reported from the Chi-square test for discrete measures and the T test/Wilcoxon Rank Sum test for continuous variables.

   See Appendix: Table 1 Characteristics of participants at baseline

2. **Objective**: Describe the clinical outcomes at median follow-up time by treatment and type of aspirin coating.

   Analysis: Unadjusted and adjusted Cox proportional hazards models with an interaction between treatment arm and aspirin coating will be fit to the data. An interaction term will be included in the primary endpoint model and all cause mortality model, to assess if the enteric coating modifies the association between treatment and these pre-specified outcomes. Hazard ratios and 95% CI will be computed.

   Adjustment measures include age, sex, ethnicity, strata, race, prior aspirin use, P2Y12 use at baseline, smoking status, no internet access, history of afib, bleeding, cad, chf, cvd, diabetes, hypertension, hyperlipidemia, prior MI, and PAD.

   See Appendix:

   Table 3**:** Interaction between enteric coating or plain aspirin and treatment for primary composite outcome and all cause mortality.

   Table 4: Event details by aspirin coating at median all cause mortality follow-up time for primary composite outcome, key secondary effectiveness outcomes and primary safety outcomes.

   Table 5: Event details by Aspirin dose and type of coating at median follow-up time for primary composite outcome, key secondary effectiveness outcomes and primary safety outcomes.

   Figure 1. Cumulative incidence of the primary effectiveness endpoint in for participants taking enteric coated or plain aspirin (cumulative incidence graph with log-rank p-value).

Results and Potential Conclusions: Event rates, counts and adjusted hazard ratios (95% CI) and the interaction p-value from the adjusted model will be included, when an interaction term is included in the models. If the p-value for the interaction is significant ($p<0.05$), then the results can be interpreted such that there is a differential treatment effect of aspirin doses (81 mg and 325 mg) between type of aspirin coating on the clinical outcome.

If the p-value for the interaction is not significant ($p>0.5$) then it will be removed.

Appendix Table 1 Characteristics of participants at baseline

**Characteristics**	**Enteric coated**	**Plain coated Aspirin**	**P Value**
Aspirin Group: 81mg	XX.X	XX.X	
Aspirin Group: 325 mg	XX.X	XX.X	
Age yrs, mean st. dev, median, (25th, 75th)	XX (XX, XX)	XX (XX, XX)	
Weight kg, mean st. dev, median (25th, 75th)	XX (XX, XX)	XX (XX, XX)	
Height cm, mean st. dev, median (25th, 75th)	XX (XX, XX)	XX (XX, XX)	
BMI kg/ m^2, mean st. dev, median (25th, 75th)	XX (XX, XX)	XX (XX, XX)	
Female, no. (%)			
Race, no. (%)			
White	XX.X	XX.X	
Black or African American	XX.X	XX.X	
Asian	XX.X	XX.X	
Hispanic ethnicity	XX.X	XX.X	
**Medical history, no. (%)**			
Prior myocardial infarction	XX.X	XX.X	
Prior coronary revascularization	XX.X	XX.X	
Prior percutaneous coronary intervention	XX.X	XX.X	
Prior coronary artery bypass graft surgery	XX.X	XX.X	
Hypertension	XX.X	XX.X	
Dyslipidemia	XX.X	XX.X	
Diabetes mellitus	XX.X	XX.X	
Atrial fibrillation	XX.X	XX.X	
Cerebrovascular disease	XX.X	XX.X	
PAD	XX.X	XX.X	
Congestive heart failure	XX.X	XX.X	
Chronic obstructive pulmonary disease/Asthma	XX.X	XX.X	
Chronic kidney disease	XX.X	XX.X	
Peripheral artery disease	XX.X	XX.X	
Prior significant gastrointestinal bleed	XX.X	XX.X	

Appendix Table 1 Characteristics of participants at baseline

Characteristics	Enteric coated	Plain coated Aspirin	P Value
Prior intracranial hemorrhage	XX.X	XX.X	
Peptic ulcer disease	XX.X	XX.X	
Current smoker	XX.X	XX.X	
P2Y12	XX.X	XX.X	
Medication for upset stomach	XX.X	XX.X	
Medications at the time of randomization, no. (%)			
Aspirin use before study			
Missing	XX.X	XX.X	
No	XX.X	XX.X	
Yes	XX.X	XX.X	
Prior daily dosage			
81 mg	XX.X	XX.X	
162 mg	XX.X	XX.X	
325 mg	XX.X	XX.X	
Missing	XX.X	XX.X	
Clopidogrel	XX.X	XX.X	
Prasugrel	XX.X	XX.X	
Other antiplatelet medication (Ticlopidine, Vorapaxar, Cilostazol)	XX.X	XX.X	

Appendix Table 2. Pre-specified adjustment measures for primary composite outcome and key secondary effectiveness outcomes

Note: **X** indicates the measure will be included in the multivariable model

Baseline Measures	Number Missing	Percent Missing (%)	Primary Endpoint	Nonfatal MI	Nonfatal Stroke	PCI CABG	All Cause Mortality	Primary Safety
Age	0	0	X	X	X	X	X	X
Sex	0	0	X	X	X	X	X	X
Current Smoker	0	0	X	X	X	X	X	
Year of Randomization	0	0						
Randomized treatment	0	0	X	X	X	X	X	X
Randomization follow-up strata	0	0	X	X		X	X	
Non-internet user at randomization	0	0	X	X		X	X	
Data mart	0	0						
Current health	0	0						
Physical function	0	0						
How invited to join	1	0						
Ethnicity	270	1.8	X	X	X	X	X	
Medical History, Coronary Artery Disease	414	2.7	X	X	X	X	X	
Medical History, MI	414	2.7	X	X		X	X	
History of CABG	414	2.7	X	X		X	X	
History of PCI	414	2.7	X	X		X	X	
Medical History, Cerebrovascular Disease	414	2.7	X	X	X	X	X	
Medical History, Hypertension	414	2.7	X	X	X	X	X	X
Medical History, Hyperlidemia	414	2.7	X	X	X	X	X	
Medical History, Atrial Fibrillation	414	2.7	X	X	X	X	X	
Medical History, Congestive Heart Failure	414	2.7	X	X	X	X	X	
Medical History, Peripheral Arterial Disease	414	2.7	X	X	X	X	X	
Medical History, Diabetes Mellitus	414	2.7	X	X	X	X	X	
History of bleeding [GI Bleed, ICH, Bleeding disorder]	414	2.7	X					
Prior Aspirin experience/dose	973	6.5	X	X	X	X	X	
Race group	980	6.5	X	X	X	X	X	
Anti-inflammatory	1260	8.4						
Medication for stomach upset	1260	8.4						
Baseline P2Y12	1261	8.4	X		X			X
Weight (kg)	2008	13.3						
Body Mass Index (kg/m2)	2352	15.6	X	X	X	X	X	X
LDL-c	7088	47						
HbA1c	9510	63.1						

Appendix Table 3. Interaction between type of aspirin coating and treatment for primary composite outcome and key secondary effectiveness outcomes

**Outcome**	**Enteric coated**			**Plain coated Aspirin**			**Adjusted Model Interaction p value**
	**81 Mg Rate**	**325 mg Rate**	**Adjusted Hazard Ratio (95% CI)**	**81 Mg Rate**	**325 mg Rate**	**Adjusted Hazard Ratio (95% CI)**	
**Primary composite endpoint:** All-cause death, MI, or stroke							XXX
All-cause death							
Nonfatal MI							
Nonfatal Stroke							
Revasc							
TIA							
Safety Major Bleeding							
Hospitalization for GI Bleed							

Appendix Table 4. Event details by type of aspirin coating at median follow-up time for primary composite outcome, key secondary effectiveness outcomes and primary safety outcomes.

Outcome	Enteric Coated<br>Events (rate)	Plain Aspirin<br>Events (rate)	Unadjusted Hazard Ratio (95% CI)	p value
**Primary composite endpoint:**				
All-cause death, MI, or stroke	XXX(YY%)	XXX(YY%)		XXX
All-cause death	XXX(YY%)	XXX(YY%)		
Hospitalization for MI	XXX(YY%)	XXX(YY%)		
Hospitalization for stroke	XXX(YY%)	XXX(YY%)		
Hospitalization for TIA	XXX(YY%)	XXX(YY%)		
Hospitalization for major bleeding with associated blood product transfusion	XXX(YY%)	XXX(YY%)		
Hospitalization for GI Bleed	XXX(YY%)	XXX(YY%)		

CI, confidence interval; MI, myocardial infarction;
Rates are calculated at median follow-up (26.2 months) using the Kalbfleisch & Prentice cumulative incidence function estimator. Events include data from electronic health record data, CMS claims, private insurance claims and confirmed participant reported outcomes (PROs).

Appendix Table 5. Event details by coated asprin type at median follow-up time for all cause mortality, key secondary effectiveness outcomes, primary safety outcomes and limb endpoints.

	**Enteric Coated**		**Plain Coated**	
	**81 Mg Rate**	**325 mg Rate**	**81 Mg Rate**	**325 mg Rate**
**Primary composite endpoint:** All-cause death, MI, or stroke	XXX(YY%)	XXX(YY%)	XXX(YY%)	XXX(YY%)
All-cause death	XXX(YY%)	XXX(YY%)	XXX(YY%)	XXX(YY%)
Hospitalization for MI	XXX(YY%)	XXX(YY%)	XXX(YY%)	XXX(YY%)
Hospitalization for stroke	XXX(YY%)	XXX(YY%)	XXX(YY%)	XXX(YY%)
Hospitalization for TIA	XXX(YY%)	XXX(YY%)	XXX(YY%)	XXX(YY%)
Hospitalization for major bleeding with associated blood product transfusion	XXX(YY%)	XXX(YY%)	XXX(YY%)	XXX(YY%)
Hospitalization for GI Bleed	XXX(YY%)	XXX(YY%)	XXX(YY%)	XXX(YY%)
Hospitalization for Revascularization	XXX(YY%)	XXX(YY%)	XXX(YY%)	XXX(YY%)

MI, myocardial infarction;
Rates are calculated at median follow-up (26.2 months) using the Kalbfleisch & Prentice cumulative incidence function estimator. Events include data from electronic health record data, CMS claims, private insurance claims and confirmed participant reported outcomes (PROs).

Appendix Figure 1. Time-to-event curves for the primary effectiveness outcome (death/MI/stroke) by enteric coated aspirin or plain aspirin.

Caption: Cumulative incidence of the primary effectiveness outcome of all-cause death, hospitalization for myocardial infarction, or hospitalization for stroke reported by Type of Aspirin Coating.
