# Supplementary material for: Effectiveness and Safety of Enteric-Coated vs Uncoated Aspirin in Patients With Cardiovascular Disease: A Secondary Analysis of the ADAPTABLE Randomized Clinical Trial
Source: JAMA Cardiol. 2023 Oct 4;8(11):1061–9. doi: 10.1001/jamacardio.2023.3364 (PMC10551818; doi:10.1001/jamacardio.2023.3364)
Supplement: Supplement 3. — eTable 1. Demographic and Presenting Characteristics, Medical History, and Medications by Aspirin Formulation Regardless of Dose eTable 2. Clinical End Points by Aspirin Formulation [Adjusted] Regardless of Dose eTable 3. Interaction of Acid Reducing Medication and Aspirin Formulation Type Regardless of Aspirin Dose on the Cumulative Incidence of Study End Points eTable 4. Event Rates of Study End Points by Formulation Type and Time-Dependent Reported Aspirin Use eFigure 1. Cumulative Incidence of the Primary End Point (All-Cause Death, Myocardial Infarction, or Stroke) by Enteric-Coated Aspirin and Uncoated (Plain Coated) Aspirin Regardless of Aspirin Dose eFigure 2. Cumulative Incidence Function of Major Bleeding by Enteric-Coated Aspirin and Uncoated (Plain Coated) Aspirin Regardless of Aspirin Dose [file jamacardiol-e233364-s003.pdf]

Supplementary Online Content

Sleem A, Effron MB, Stebbins A, et al. Effectiveness and safety of enteric coated vs uncoated aspirin in patients with cardiovascular disease: a secondary analysis of the ADAPTABLE randomized clinical trial. Published online October 4, 2023. *JAMA Cardiol*. doi:10.1001/jamacardio.2023.3364

eTable 1. Demographic and Presenting Characteristics, Medical History, and Medications by Aspirin Formulation Regardless of Dose

eTable 2. Clinical End Points by Aspirin Formulation [Adjusted] Regardless of Dose

eTable 3. Interaction of Acid Reducing Medication and Aspirin Formulation Type Regardless of Aspirin Dose on the Cumulative Incidence of Study End Points

eTable 4. Event Rates of Study End Points by Formulation Type and Time-Dependent Reported Aspirin Use

eFigure 1. Cumulative Incidence of the Primary End Point (All-Cause Death, Myocardial Infarction, or Stroke) by EC-ASA and Uncoated (Plain Coated) ASA Regardless of Aspirin Dose

eFigure 2. Cumulative Incidence Function of Major Bleeding by EC-ASA and Uncoated (Plain Coated) ASA Regardless of Aspirin Dose

This supplementary material has been provided by the authors to give readers additional information about their work.

© 2023 Sleem A et al. *JAMA Cardiology*.

eTable 1. Demographic and Presenting Characteristics, Medical History, and Medications by Aspirin Formulation Regardless of Dose				
Characteristic[a]	All participants reporting formulation of ASA used (N=10,678)	Enteric coated ASA (N=7366)	Uncoated ASA (N=3312)	P value
Age (yrs)				<.0001
n	10678	7366	3312	
Median (25th, 75th)	68.0 (61.3, 73.7)	68.3 (61.8, 73.8)	67.2 (60.4, 73.3)	
Female	3393 (31.8%)	2304 (31.3%)	1089 (32.9%)	0.10
Randomized Dose				<.0001
81-mg	5374 (50.3%)	4074 (55.3%)	1300 (39.3%)	
325mg	5304 (49.7%)	3292 (44.7%)	2012 (60.7%)	
History of PAD				0.14
No	7980 (76.6%)	5539 (77.0%)	2441 (75.7%)	
Yes	2436 (23.4%)	1652 (23.0%)	784 (24.3%)	
Race				0.49
White	8965 (84.0%)	6218 (84.4%)	2747 (82.9%)	
Black or African American	900 (8.4%)	592 (8.0%)	308 (9.3%)	

© 2023 Sleem A et al. *JAMA Cardiology*.

eTable 1. Demographic and Presenting Characteristics, Medical History, and Medications by Aspirin Formulation Regardless of Dose				
Characteristic[a]	All participants reporting formulation of ASA used (N=10,678)	Enteric coated ASA (N=7366)	Uncoated ASA (N=3312)	P value
Other	482 (4.5%)	331 (4.5%)	151 (4.6%)	
Prefer not to say or missing	331 (3.1%)	225 (3.1%)	106 (3.2%)	
Ethnicity				0.23
Hispanic	270 (2.5%)	181 (2.5%)	89 (2.7%)	
Non-hispanic	10051 (94.2%)	6954 (94.4%)	3097 (93.6%)	
Did not respond	354 (3.3%)	231 (3.1%)	123 (3.7%)	
Current Smoker	1006 (9.4%)	640 (8.7%)	366 (11.1%)	0.0001
Baseline $P2Y_{12}$ inhibitor use	2300(22.0%)	1587(22.0%)	713(22.0%)	0.99
EHR and Claims Information				
Weight (kg) (N)	9313	6451	2862	
Median (IQR)	90.0 (78.2, 103.6)	90.0 (78.2, 103.6)	90.9 (78.6, 104.5)	0.09
BMI (N)	9074	6293	2781	
Median (IQR)	30.0 (26.7, 34.2)	30.0 (26.6, 34.1)	30.2 (26.9, 34.5)	0.03
Atrial Fibrillation/Flutter	884 (8.5%)	611 (8.5%)	273 (8.5%)	0.96

© 2023 Sleem A et al. *JAMA Cardiology*.

eTable 1. Demographic and Presenting Characteristics, Medical History, and Medications by Aspirin Formulation Regardless of Dose				
Characteristic[a]	All participants reporting formulation of ASA used (N=10,678)	Enteric coated ASA (N=7366)	Uncoated ASA (N=3312)	P value
History of Bleeding[†]	894 (8.6%)	595 (8.3%)	299 (9.3%)	0.09
Significant GI Bleed	661 (6.3%)	446 (6.2%)	215 (6.7%)	0.37
History of ICH	155 (1.5%)	105 (1.5%)	50 (1.6%)	0.73
History of bleeding disorder	126 (1.2%)	73 (1.0%)	53 (1.6%)	0.007
Prior CABG	2525 (24.2%)	1763 (24.5%)	762 (23.6%)	0.33
CHF	2392 (23.0%)	1646 (22.9%)	746 (23.1%)	0.79
Chronic Kidney Disease	1835 (17.6%)	1265 (17.6%)	570 (17.7%)	0.92
COPD	1938 (18.6%)	1329 (18.5%)	609 (18.9%)	0.63
Diabetes	3988 (38.3%)	2696 (37.5%)	1292 (40.1%)	0.01
Hypertension	8873 (85.2%)	6147 (85.5%)	2726 (84.5%)	0.20
Hyperlipidemia	9232 (88.6%)	6426 (89.4%)	2806 (87.0%)	<0.001
Prior MI	3739(35.9%)	2547 (35.4%)	1192 (37.0%)	0.13
Peptic Ulcer Disease	312 (3.0%)	211 (2.9%)	101 (3.1%)	0.58
PCI/CABG	5648 (54.2%)	3911 (54.4%)	1737 (53.9%)	0.62

© 2023 Sleem A et al. *JAMA Cardiology*.

eTable 1. Demographic and Presenting Characteristics, Medical History, and Medications by Aspirin Formulation Regardless of Dose

Characteristic[a]	All participants reporting formulation of ASA used (N=10,678)	Enteric coated ASA (N=7366)	Uncoated ASA (N=3312)	P value
Prior Aspirin Use	10275 (96.2%)	7127 (96.8%)	3148 (95.0%)	<.0001
Prior Aspirin Dose				<.0001
81-mg	8714 (84.9%)	6125 (86.0%)	2589 (82.5%)	
162 mg	250 (2.4%)	179 (2.5%)	71 (2.3%)	
325 mg	1295 (12.6%)	815 (11.4%)	480 (15.3%)	
Other	16 (0.2%)	8 (0.1%)	8 (0.3%)	
ARM	2767(38.4%)	2767(38.4%)	1137(35.1%)	<0.002

[a] All values n (CIR%) except as noted

[b] History of bleeding includes GI Bleed, ICH and Bleeding disorder

ARM – acid reducing medication, ASA – aspirin, BMI - body mass index, CABG – coronary artery bypass graft surgery, CHF – congestive heart failure, COPD – chronic obstructive pulmonary disease, EHR – electronic health record, GI – gastrointestinal, ICH – intracranial hemorrhage, MI – myocardial infarction, PAD – peripheral arterial disease, PCI – percutaneous coronary intervention

© 2023 Sleem A et al. *JAMA Cardiology*.

eTable 2. Clinical End Points by Aspirin Formulation [Adjusted] Regardless of Dose

	Uncoated Coated ASA	Enteric Coated ASA	Adjusted Cox Model	
	Event (CI rate %)	Event (CI rate %)	HR (95% CI)	P Value
Death/MI/Stroke	266 (7.9%)	543 (6.8%)	0.94 (0.80, 1.09)	0.40
All-cause mortality	155 (4.3%)	301 (3.7%)	0.91 (0.75, 1.11)	0.35
MI	101 (3.4%)	219 (2.9%)	0.99 (0.78, 1.26)	0.94
Stroke	43 (1.2%)	90 (1.2%)	0.93 (0.64, 1.35)	0.69
Major Bleeding	24 (0.7%)	45 (0.6%)	0.82 (0.49, 1.37)	0.46
PCI or CABG	195 (6.4%)	477 (6.4%)	1.10 (0.92, 1.30)	0.29
TIA	4 (0.1%)	24 (0.3%)	2.61 (0.90, 7.54)	0.08
GI Bleed	54 (1.5%)	88 (1.2%)	0.72 (0.51, 1.02)	0.06

Cumulative Incidence is reported at 26.2 months from randomization [the median follow up time for all-cause mortality in Adaptable].

Primary endpoint, nonfatal MI, all-cause mortality and revascularization adjustment measures are randomized treatment, age, current smoking, randomized follow up strata, no internet use at randomization, race, ethnicity, history of CAD, MI, CABG, PCI, CVD, hypertension, hyperlipidemia, atrial fibrillation, CHF, PAD, prior aspirin use, diabetes, history of bleeding, BMI, and baseline $P2Y_{12}$ inhibitor use.
Stroke, TIA and GI bleeding adjustment measures are randomized treatment, age, current smoking, ethnicity, race, history of CAD, CVD, hypertension, hyperlipidemia, atrial fibrillation, CHF, PAD, diabetes, prior aspirin use, baseline $P2Y_{12}$, diabetes, history of bleeding, and BMI.

ASA - aspirin, BMI – body mass index, CAD – coronary artery disease, CABG - coronary artery bypass grafting, CVD – cardiovascular disease, GI – gastrointestinal, MI – myocardial infarction, PAD - peripheral arterial disease, TIA – transient ischemic attack

© 2023 Sleem A et al. *JAMA Cardiology*.

eTable 3. Interaction of Acid Reducing Medication and Aspirin Formulation Type Regardless of Aspirin Dose on the Cumulative Incidence of Study End Points

	Enteric coated Aspirin	Uncoated Aspirin	Adjusted Cox model
	Event (CI rate %)	Event (CI rate %)	Interaction P Value
Death/MI/stroke	543 (6.8%)	266 (7.9%)	0.55
All-cause mortality	301 (3.7%)	155 (4.3%)	0.75
MI	219 (2.9%)	101 (3.4%)	0.49
Stroke	90 (1.2%)	43 (1.2%)	0.10
Major bleeding	45 (0.6%)	24 (0.7%)	0.52
PCI or CABG	477 (6.4%)	195 (6.4%)	0.45
TIA	24 (0.3%)	4 (0.1%)	0.91
GI bleed	88 (1.2%)	54 (1.5%)	0.64

CI reported at 26.2 months from randomization (median follow-up time for all-cause mortality in ADAPTABLE)

Primary endpoint, MI, all-cause mortality, and revascularization adjustment measures are randomized treatment, age, current smoking, randomized follow-up strata, no internet use at randomization, race, ethnicity, history of CAD, MI, CABG, PCI, CVD, hypertension, hyperlipidemia, atrial fibrillation, CHF, PAD, prior aspirin use, BMI, and baseline $P2Y_{12}$ inhibitor.

© 2023 Sleem A et al. *JAMA Cardiology*.

Major bleeding adjustment measures are randomized treatment, diabetes, age, sex, hypertension, baseline $P2Y_{12}$ inhibitor, and BMI.

Stroke, TIA, and GI bleeding adjustment measures are randomized treatment, age, current smoking, ethnicity, race, history of CAD, CVD, hypertension, hyperlipidemia, atrial fibrillation, CHF, PAD, diabetes, prior aspirin use, baseline $P2Y_{12}$ inhibitor, and BMI.

ASA – aspirin, BMI - body mass index, CAD - coronary artery disease, CABG – coronary artery bypass graft surgery, CHF – congestive heart failure, CI – cumulative incidence, COPD – chronic obstructive pulmonary disease, CVD – cardiovascular disease, EHR – electronic health record, GI – gastrointestinal, ICH – intracranial hemorrhage, MI – myocardial infarction, PAD – peripheral arterial disease, PCI – percutaneous coronary intervention

© 2023 Sleem A et al. *JAMA Cardiology*.

eTable 4. Event Rates of Study End Points by Formulation Type and Time-Dependent Reported Aspirin Use						
			Unadjusted Model		Adjusted Model	
	Taking 81 mg rate (N)	Taking 325 mg rate (N)	HR (95% CI)	P Value	HR (95% CI)	P Value
Primary Endpoint [Death,MI,Stroke] Overall interaction p value				0.85		0.87
Enteric coated	3.7 (340)	2.8 (129)	1.30 (1.06, 1.60)	0.01	1.27 (1.03, 1.55)	0.02
Uncoated	3.8 (141)	3.2 (66)	1.16 (0.87, 1.56)	0.32	1.12 (0.83, 1.50)	0.45
All Cause Death: Overall interaction p value				0.96		0.95
Enteric coated	1.9 (183)	1.4 (67)	1.28 (0.97, 1.70)	0.08	1.22 (0.92, 1.61)	0.17
Uncoated	2.3 (88)	1.5 (31)	1.47 (0.98, 2.22)	0.06	1.38 (0.91, 2.08)	0.12
Primary Safety Endpoint [Major bleeding]: Overall interaction p value				0.86		0.88
Enteric coated	0.28 (26)	0.3 (13)	1.02 (0.52, 1.99)	0.95	1.04 (0.53, 2.02)	0.92
Uncoated	0.35 (13)	0.2 (4)	1.83 (0.60, 5.63)	0.29	1.79 (0.58, 548)	0.32

Rates and HR reflect the effect of the time-varying reported dose on the end point. Rates are calculated as annualized event rates (events per 100 patient-years).

Adjustment model includes age, race, ethnicity, prior aspirin dose, prior MI, prior PCI, history of atrial fibrillation, non-internet user at randomization, history of bleeds, and baseline $P2Y_{12}$ use.

CI – confidence interval, HR- hazard ratio, MI – myocardial infarction, N - number

© 2023 Sleem A et al. *JAMA Cardiology*.

eFigure 1. Cumulative Incidence of the Primary End Point (All-Cause Death, Myocardial Infarction, or Stroke) by EC-ASA and Uncoated (Plain Coated) ASA Regardless of Aspirin Dose

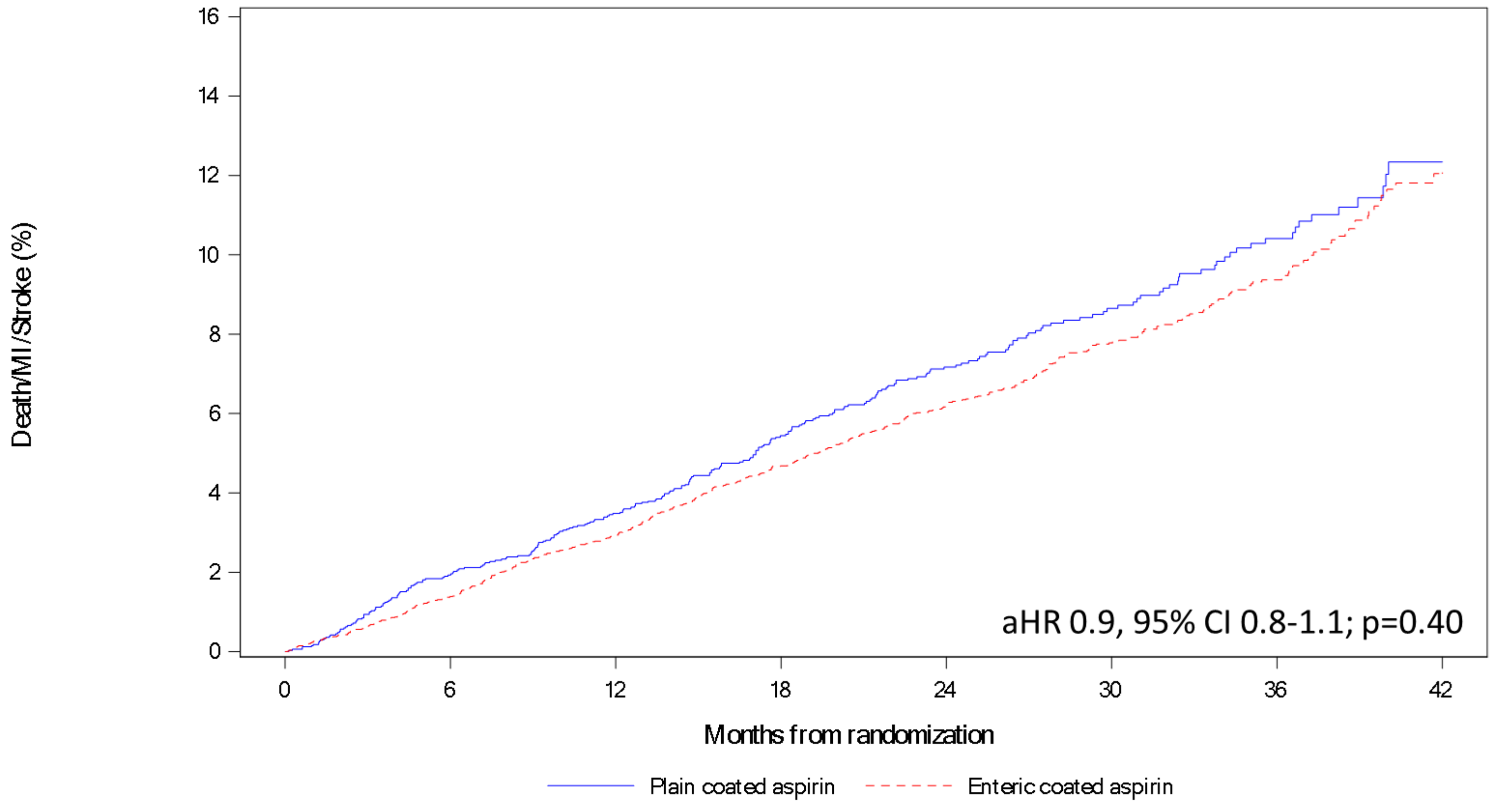

At Risk								
Plain coated aspirin	3312	3244	3174	2517	1852	1164	683	296
Enteric coated aspirin	7366	7242	7102	5725	4320	2818	1613	648

© 2023 Sleem A et al. *JAMA Cardiology*.

eFigure 2. Cumulative Incidence Function of Major Bleeding by EC-ASA and Uncoated (Plain Coated) ASA Regardless of Aspirin Dose

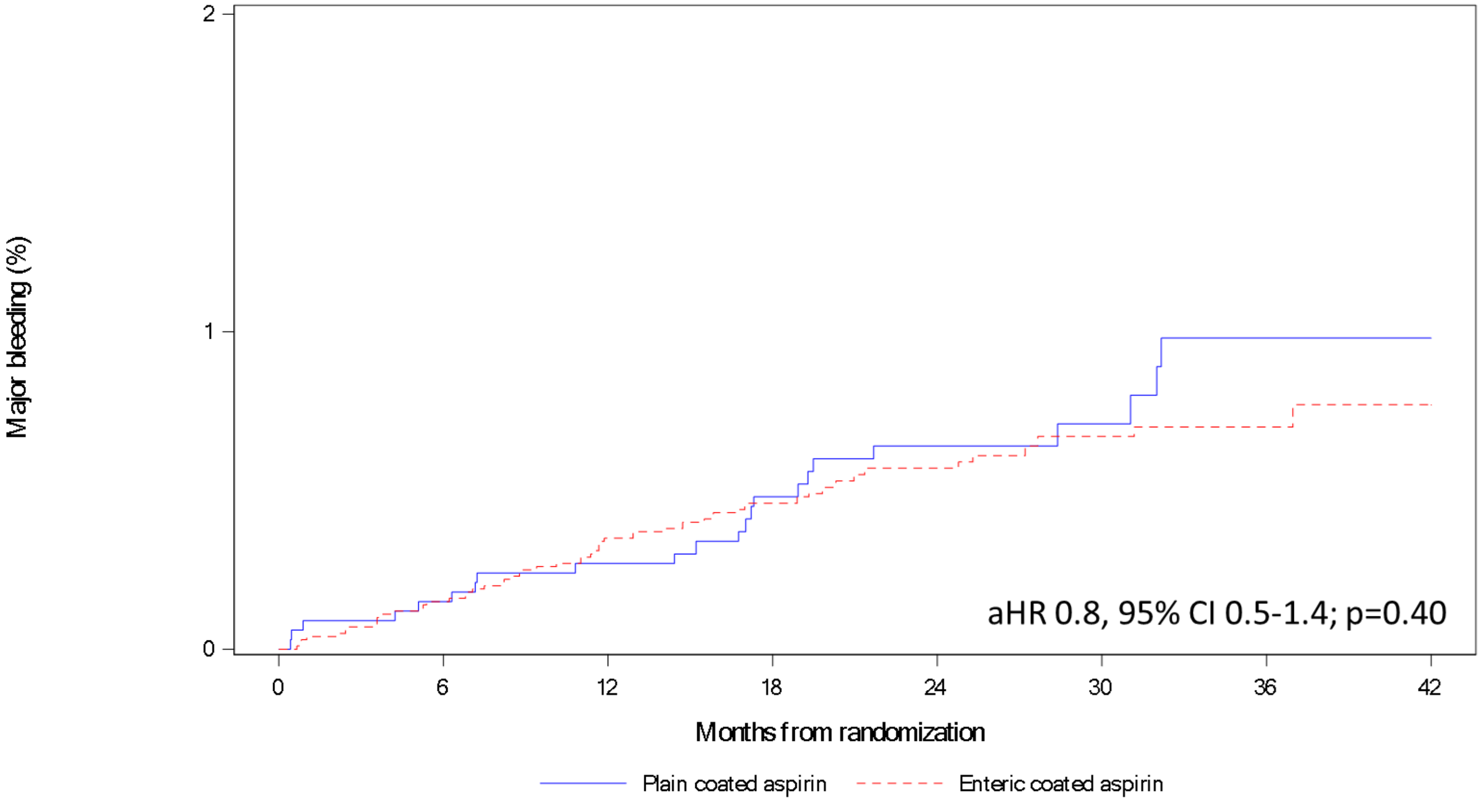

At Risk

	0	6	12	18	24	30	36	42
Plain coated aspirin	3312	3274	3231	2578	1912	1214	710	320
Enteric coated aspirin	7366	7298	7200	5862	4443	2911	1672	676

© 2023 Sleem A et al. *JAMA Cardiology*.
