# Supplementary material for: Effectiveness and Safety of Enteric-Coated vs Uncoated Aspirin in Patients With Cardiovascular Disease: A Secondary Analysis of the ADAPTABLE Randomized Clinical Trial
Source: JAMA Cardiol. 2023 Oct 4;8(11):1061–9. doi: 10.1001/jamacardio.2023.3364 (PMC10551818; doi:10.1001/jamacardio.2023.3364)
Supplement: Supplement 4. — Data Sharing Statement [file jamacardiol-e233364-s004.pdf]

Data Sharing Statement

Data
Data available: Yes
Data types: Deidentified participant data
How to access data: Data from ADAPTABLE will be shared publicly according to PCORI requirements. Final steps are being completed to deposit the study data in the Inter-university Consortium for Political and Social Research (ICPSR) at the University of Michigan.
When available: With publication

Supporting Documents
Document types: None

Additional Information
Who can access the data: Data from ADAPTABLE will be shared publicly according to PCORI requirements. Final steps are being completed to deposit the study data in the Inter-university Consortium for Political and Social Research (ICPSR) at the University of Michigan.
Types of analyses: Data from ADAPTABLE will be shared publicly according to PCORI requirements. Final steps are being completed to deposit the study data in the Inter-university Consortium for Political and Social Research (ICPSR) at the University of Michigan.
Mechanisms of data availability: Data from ADAPTABLE will be shared publicly according to PCORI requirements. Final steps are being completed to deposit the study data in the Inter-university Consortium for Political and Social Research (ICPSR) at the University of Michigan.
